# Quantifying risks and interventions that have affected the burden of diarrhoea among children younger than 5 years: an analysis of the Global Burden of Disease Study 2017

**DOI:** 10.1016/S1473-3099(19)30401-3

**Published:** 2020-01

**Authors:** GBD 2017 Diarrhoeal Disease Collaborators, Christopher E Troeger, Ibrahim A. Khalil, Brigette F. Blacker, Molly H. Biehl, Samuel B. Albertson, Stephanie R M Zimsen, Puja C Rao, Degu Abate, Alireza Ahmadi, Mohamed Lemine Cheikh brahim Ahmed, Chalachew Genet Akal, Fares Alahdab, Noore Alam, Kefyalew Addis Alene, Vahid Alipour, Syed Mohamed Aljunid, Rajaa M Al-Raddadi, Nelson Alvis-Guzman, Saeed Amini, Nahla Hamed Anber, Mina Anjomshoa, Carl Abelardo T. Antonio, Jalal Arabloo, Olatunde Aremu, Hagos Tasew Atalay, Suleman Atique, Euripide F G A Avokpaho, Samah Awad, Ashish Awasthi, Alaa Badawi, Kalpana Balakrishnan, Joseph Adel Mattar Banoub, Aleksandra Barac, Quique Bassat, Neeraj Bedi, Derrick A. Bennett, Krittika Bhattacharyya, Zulfiqar A Bhutta, Ali Bijani, Josip Car, Félix Carvalho, Carlos A Castañeda-Orjuela, Devasahayam J Christopher, Lalit Dandona, Rakhi Dandona, Ahmad Daryani, Feleke Mekonnen Demeke, Aniruddha Deshpande, Shirin Djalalinia, Manisha Dubey, Eleonora Dubljanin, Eyasu Ejeta Duken, Maysaa El Sayed Zaki, Aman Yesuf Endries, Eduarda Fernandes, Florian Fischer, Nancy Fullman, William M. Gardner, Birhanu Geta, Keyghobad Ghadiri, Giuseppe Gorini, Alessandra C Goulart, Yuming Guo, Gessessew Bugssa Hailu, Arvin Haj-Mirzaian, Arya Haj-Mirzaian, Samer Hamidi, Hamid Yimam Hassen, Chi Linh Hoang, Mihaela Hostiuc, Zakir Hussain, Seyed Sina Naghibi Irvani, Spencer L. James, Ravi Prakash Jha, Jost B. Jonas, André Karch, Amir Kasaeian, Tesfaye Dessale Kassa, Nicholas J Kassebaum, Adane Teshome Kefale, Yousef Saleh Khader, Ejaz Ahmad Khan, Md Nuruzzaman Khan, Young-Ho Khang, Abdullah T Khoja, Ruth W Kimokoti, Adnan Kisa, Sezer Kisa, Niranjan Kissoon, Sonali Kochhar, Soewarta Kosen, Ai Koyanagi, Barthelemy Kuate Defo, G Anil Kumar, Dharmesh Kumar Lal, Cheru Tesema Leshargie, Shanshan Li, Rakesh Lodha, Erlyn Rachelle King Macarayan, Marek Majdan, Abdullah A. Mamun, Helena Manguerra, Addisu Melese, Ziad A Memish, Desalegn Tadese Mengistu, Tuomo J Meretoja, Tomislav Mestrovic, Bartosz Miazgowski, Erkin M Mirrakhimov, Babak Moazen, Karzan Abdulmuhsin Mohammad, Shafiu Mohammed, Lorenzo Monasta, Catrin E Moore, Jonathan F. Mosser, Seyyed Meysam Mousavi, Srinivas Murthy, Ghulam Mustafa, Javad Nazari, Cuong Tat Nguyen, Long Hoang Nguyen, Muhammad Imran Nisar, Molly R Nixon, Felix Akpojene Ogbo, Anselm Okoro, Andrew T Olagunju, Tinuke O Olagunju, Mahesh P A, Smita Pakhale, Maarten J Postma, Mostafa Qorbani, Reginald Quansah, Alireza Rafiei, Fakher Rahim, Vafa Rahimi-Movaghar, Rajesh Kumar Rai, Mohammad Sadegh Rezai, Aziz Rezapour, Maria Jesus Rios-Blancas, Luca Ronfani, Katherine Rosettie, Dietrich Rothenbacher, Saeed Safari, Zikria Saleem, Evanson Zondani Sambala, Abdallah M. Samy, Milena M Santric Milicevic, Benn Sartorius, Monika Sawhney, Seyedmojtaba Seyedmousavi, Masood Ali Shaikh, Aziz Sheikh, Mika Shigematsu, David L Smith, Joan B Soriano, Chandrashekhar T Sreeramareddy, Jeffrey D Stanaway, Mu'awiyyah Babale Sufiyan, Teklay G E Teklu, Mohamad-Hani Temsah, Belay Tessema, Bach Xuan Tran, Khanh Bao Tran, Irfan Ullah, Rachel L Updike, Tommi Juhani Vasankari, Yousef Veisani, Fiseha Wadilo Wada, Yasir Waheed, Marcia Weaver, Kirsten E Wiens, Charles Shey Wiysonge, Ebrahim M Yimer, Naohiro Yonemoto, Zoubida Zaidi, Heather J Zar, Afshin Zarghi, Stephen S Lim, Theo Vos, Ali H Mokdad, Christopher J L Murray, Hmwe Hmwe Kyu, Simon I. Hay, Robert C Reiner

## Abstract

**Background:**

Many countries have shown marked declines in diarrhoeal disease mortality among children younger than 5 years. With this analysis, we provide updated results on diarrhoeal disease mortality among children younger than 5 years from the Global Burden of Diseases, Injuries, and Risk Factors Study 2017 (GBD 2017) and use the study's comparative risk assessment to quantify trends and effects of risk factors, interventions, and broader sociodemographic development on mortality changes in 195 countries and territories from 1990 to 2017.

**Methods:**

This analysis for GBD 2017 had three main components. Diarrhoea mortality was modelled using vital registration data, demographic surveillance data, and verbal autopsy data in a predictive, Bayesian, ensemble modelling tool; and the attribution of risk factors and interventions for diarrhoea were modelled in a counterfactual framework that combines modelled population-level prevalence of the exposure to each risk or intervention with the relative risk of diarrhoea given exposure to that factor. We assessed the relative and absolute change in diarrhoea mortality rate between 1990 and 2017, and used the change in risk factor exposure and sociodemographic status to explain differences in the trends of diarrhoea mortality among children younger than 5 years.

**Findings:**

Diarrhoea was responsible for an estimated 533 768 deaths (95% uncertainty interval 477 162–593 145) among children younger than 5 years globally in 2017, a rate of 78·4 deaths (70·1–87·1) per 100 000 children. The diarrhoea mortality rate ranged between countries by over 685 deaths per 100 000 children. Diarrhoea mortality per 100 000 globally decreased by 69·6% (63·1–74·6) between 1990 and 2017. Among the risk factors considered in this study, those responsible for the largest declines in the diarrhoea mortality rate were reduction in exposure to unsafe sanitation (13·3% decrease, 11·2–15·5), childhood wasting (9·9% decrease, 9·6–10·2), and low use of oral rehydration solution (6·9% decrease, 4·8–8·4).

**Interpretation:**

Diarrhoea mortality has declined substantially since 1990, although there are variations by country. Improvements in sociodemographic indicators might explain some of these trends, but changes in exposure to risk factors—particularly unsafe sanitation, childhood growth failure, and low use of oral rehydration solution—appear to be related to the relative and absolute rates of decline in diarrhoea mortality. Although the most effective interventions might vary by country or region, identifying and scaling up the interventions aimed at preventing and protecting against diarrhoea that have already reduced diarrhoea mortality could further avert many thousands of deaths due to this illness.

**Funding:**

Bill & Melinda Gates Foundation.

## Introduction

Diarrhoeal diseases are the second leading infectious cause of mortality globally, after lower respiratory infections, among children younger than 5 years, although childhood mortality due to diarrhoea has declined since the 1990s.^1^, ^2^, ^3^, ^4^, ^5^ Accelerating and maintaining these declines is essential to meeting Sustainable Development Goals for under-5 childhood mortality and ensuring that children everywhere have the opportunity of a full, healthy life.

Many countries have shown marked declines in diarrhoea mortality among children younger than 5 years, and much of this progress has been associated with programmes addressing key environmental risks for diarrhoea and scaling up interventions to prevent or treat acute diarrhoea.^3^ Several global initiatives have offered guidance on efficient and recommended interventions to avert illness and mortality due to diarrhoea, including the Global Action Plan for Pneumonia and Diarrhea.^6^ These programmes have typically categorised interventions into groups that are defined by the stage in the morbidity pathway at which they occur, including prevention of infection (such as provision of safe water and sanitation) and treatment of disease (such as administration of oral rehydration solution).^7^ Studies have shown that rotavirus vaccine,^8^ safe water and sanitation,^9^, ^10^ nutrition supplementation,^10^ and use of oral rehydration solution^11^ protect children younger than 5 years against diarrhoea illness and mortality. However, no study to date has assessed the contribution of a range of risk factors and interventions on diarrhoea mortality across settings and over time.

**Research in context**

**Evidence before this study**

The Global Burden of Diseases Study (GBD) has produced a series of updates to estimates of health loss due to over 300 causes of death and disability, including diarrhoea. There are several other groups that also estimate diarrhoea mortality, particularly among children younger than 5 years, including the partnership between WHO and Maternal and Child Epidemiology Estimation. Although the estimates vary slightly between GBD iterations and compared with other groups' estimates, one thing that is generally agreed on is that diarrhoea mortality among children younger than 5 years is decreasing over time. We searched PubMed from Jan 1, 2005, to April 29, 2019, with the search terms “diarrhea AND mortality AND global AND risk AND trend*” and found 36 results, from which at least seven were using GBD results. This search found a few attempts to quantify the burden of individual risk factors, such as vitamin deficiency or breastfeeding, in a cross-sectional way; however, to our knowledge, no other study has attempted to evaluate trends in under-5 diarrhoea mortality over time due to demographic changes and changes in exposure to multiple risk factors.

**Added value of this study**

Here we report findings from GBD 2017, which builds on previous iterations of GBD with additional data and modelling improvements. We used estimates of 12 risk factors or interventions for diarrhoea mortality (handwashing, low rotavirus vaccine coverage, unsafe sanitation, unsafe water, zinc deficiency, childhood stunting, childhood underweight, childhood wasting, low use of oral rehydration solution, low birthweight and short gestation, suboptimal breastfeeding, and vitamin A deficiency), produced for the GBD study, to evaluate changes in diarrhoea mortality among children younger than 5 years. A major component of the GBD study is producing internally consistent and externally comparable estimates for all locations and over time, which allows us to identify countries where the diarrhoea mortality rate has changed the most and to evaluate the contributing risk factors or interventions that affect the mortality rate. We provide cross-sectional and longitudinal estimates of the reasons for which children are dying from diarrhoea, how these reasons vary, and where specific interventions might have the greatest impact.

**Implications of all the available evidence**

Diarrhoea mortality among children younger than 5 years has declined in many parts of the world, particularly because of improvements in safe sanitation, childhood nutrition, and use of oral rehydration solution. However, there is variation by country, suggesting that there is no single solution to reduce diarrhoea mortality. Every country must consider their specific context to identify strategies to reduce diarrhoea mortality. Our estimates can help provide evidence to do so.

We have previously shown a strong relationship between sociodemographic level and diarrhoea mortality^2^, ^3^ but that does not necessarily translate to actionable evidence of interventions to prioritise to reduce health loss associated with diarrhoea. Understanding why some countries have seen more progress than others would provide such evidence and give a targeted roadmap for accelerating declines in diarrhoea mortality. The Global Burden of Diseases, Injuries, and Risk Factors Study 2017 (GBD 2017) is a systematic, scientific effort to quantify morbidity and mortality, including for diarrhoea and its risk factors.^1^, ^12^, ^13^ With this analysis, we provide updated results of estimates of diarrhoea mortality among children younger than 5 years and use GBD's comparative risk assessment to quantify trends and effects of risk factors, interventions, and broader sociodemographic development on mortality changes from 1990 to 2017. We use results from the GBD 2017 to assess which countries have performed best in reducing under-5 diarrhoea mortality and compare countries on the basis of mortality rates, exposure to risk factors and interventions, and the contribution of changes in risk factor exposure to diarrhoea mortality.

## Methods

### Overview

Detailed methods for GBD and diarrhoea estimation in GBD have been previously published.^1^, ^2^, ^3^, ^12^, ^13^ Herein, we describe these methods briefly, focusing on a high-level description of modelling strategy. Further information about diarrhoea mortality modelling in GBD 2017 is provided in the appendix (p 2). Uncertainty in the diarrhoea estimates is maintained through the modelling process using 1000 draws and is reflected as 2·5th and 97·5th percentiles of the posterior distribution. In compliance with the Guidelines for Accurate and Transparent Health Estimates Reporting,^14^ data and code for the GBD 2017 cycle are publicly available.

This analysis had three main components: diarrhoea mortality estimation, diarrhoea risk factor and intervention estimation, and the application of these results to evaluate changes in diarrhoea mortality between 1990 and 2017. Although the GBD 2017 study also produced estimates of diarrhoea incidence, results are not presented here but can be found on the GBD-Compare website.

### Mortality

Most causes of death in GBD 2017, including diarrhoea, are modelled with the Cause of Death Ensemble model (CODEm).^1^, ^15^ This statistical tool is designed to create a wide variety of models using a covariate selection algorithm and then to weight these models on the basis of their out-of-sample predictive validity. These models are combined into an ensemble that predicts diarrhoea mortality by age, sex, year, and location from 1980 to 2017. The model for diarrhoea uses vital registration data, demographic surveillance data, and verbal autopsy data. Covariates include childhood undernutrition, safe water and sanitation, the Socio-demographic Index (SDI), and maternal education, among others.^1^ SDI is a composite measure of development that accounts for fertility, education, and income and is associated with many population health indicators, including diarrhoea mortality.^2^

### Risk factors

Risk factors in GBD 2017 are causally related to diarrhoea incidence or mortality.^13^ There are 12 risk factors or interventions for diarrhoea estimated in GBD 2017 (no handwashing with soap, low rotavirus vaccine coverage, unsafe sanitation, unsafe water, zinc deficiency, childhood stunting, childhood underweight, childhood wasting, low use of oral rehydration solution, low birthweight and short gestation, suboptimal breastfeeding, and vitamin A deficiency; details for their estimation are included in the appendix pp 7–57). In general, risk factors are part of a comparative risk assessment framework that involves a counterfactual approach to quantify the level of exposure to the risk factor in a population and the relative risk of diarrhoea given exposure. Typically, the exposure in a population is modelled on the basis of surveys and scientific literature and the risk of diarrhoea is from published meta-analyses or from systematic reviews done for GBD.^13^ For some risk factors in this analysis, notably unsafe water and sanitation, evidence of the risk of diarrhoea morbidity is assumed to represent the risk of diarrhoea mortality, because little or no data are available for mortality as the primary study outcome. More information on the modelling strategy for the risk factors can be found in the appendix (pp 10–53). We also used existing aggregate risk factors for childhood growth failure (ie, stunting, underweight, and wasting) and unsafe water, unsafe sanitation, and lack of access to handwashing with soap.^13^

Risk factors are counterfactual and estimated independently. For these reasons, the sum of risk-factor attributable fractions is not equal to 100% in a given population and could be more or less, depending on the population-level exposure to each factor. In other words, in this counterfactual approach, there are multiple potential ways to avert an episode of diarrhoea or prevent a death due to diarrhoea. For example, a child who dies from diarrhoea might have lacked adequate nutrition or safe water but access to either might have saved their life, even without access to the other.

### Analysis of temporal trends

The last component of our analysis was the application of the results for diarrhoea-related mortality and risk factors to space and time patterns. The primary outcome of interest was the diarrhoea mortality rate per 100 000 children younger than 5 years in 1990 and 2017. We calculated the absolute change in mortality rate as the difference between rates in 1990 and those in 2017. We calculated the relative percent change in mortality rate in 2017 compared with 1990. We fitted a log-normal regression using SDI to predict the expected change in diarrhoeal mortality rate per unit increase in SDI. This rate was considered the baseline change in diarrhoeal mortality that is explained by changes in SDI.

To assess the effect of changes for each risk factor among children younger than 5 years, we took advantage of the counterfactual definition of risk factor burden such that the diarrhoea mortality rate due to each risk factor was equivalent to the reduction expected given complete absence of the risk factor. We categorised risk factors for diarrhoea mortality into two groups based on their biological mechanism of risk: prevention and protection. Prevention-associated risks are those that increase the probability of having diarrhoeal disease and include lack of rotavirus vaccine, no handwashing with soap, unsafe water, unsafe sanitation, and dietary zinc deficiency. Protection-associated risks are those that increase the likelihood of death among children with diarrhoea and include suboptimal breastfeeding, not receiving oral rehydration solution, low birthweight and short gestation, childhood stunting, childhood underweight, childhood wasting, and vitamin A deficiency (appendix pp 7–9). We decomposed the effect of the change in exposure to each risk factor on the diarrhoea mortality rate between 1990 and 2017, accounting for the independent effects of population growth, population ageing, and other drivers of diarrhoea mortality. This process has been described in detail elsewhere.^3^, ^13^

### Role of the funding source

The funder of the study played no role in study design, data collection, data analysis, data interpretation, or writing of the report. All collaborators had full access to all the data in the study and the corresponding author had final responsibility for the decision to submit for publication.

## Results

**Figure 1:**
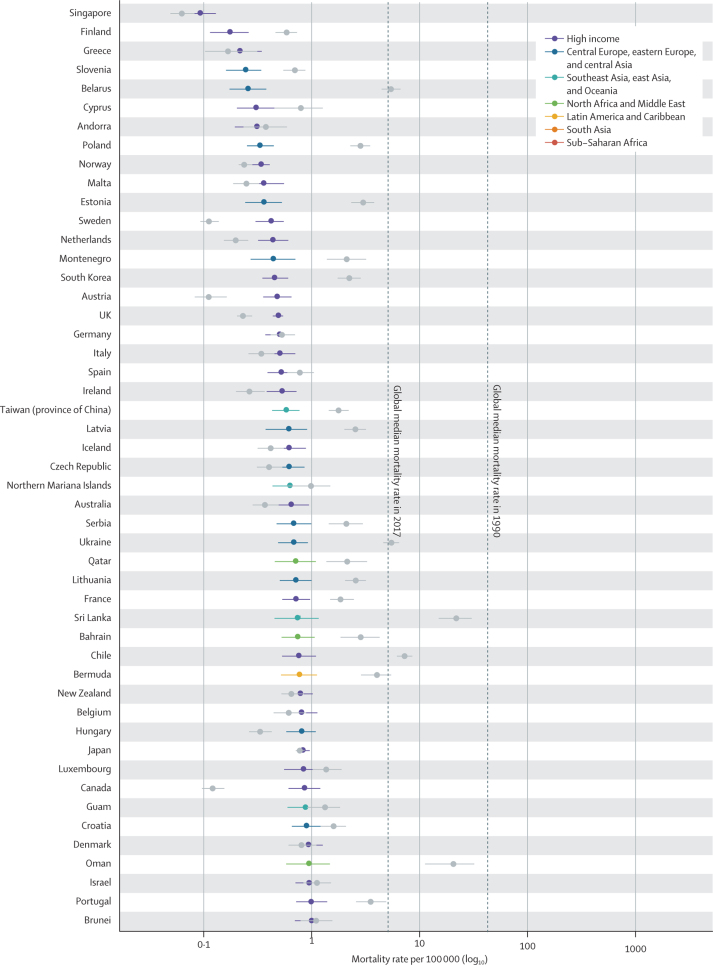

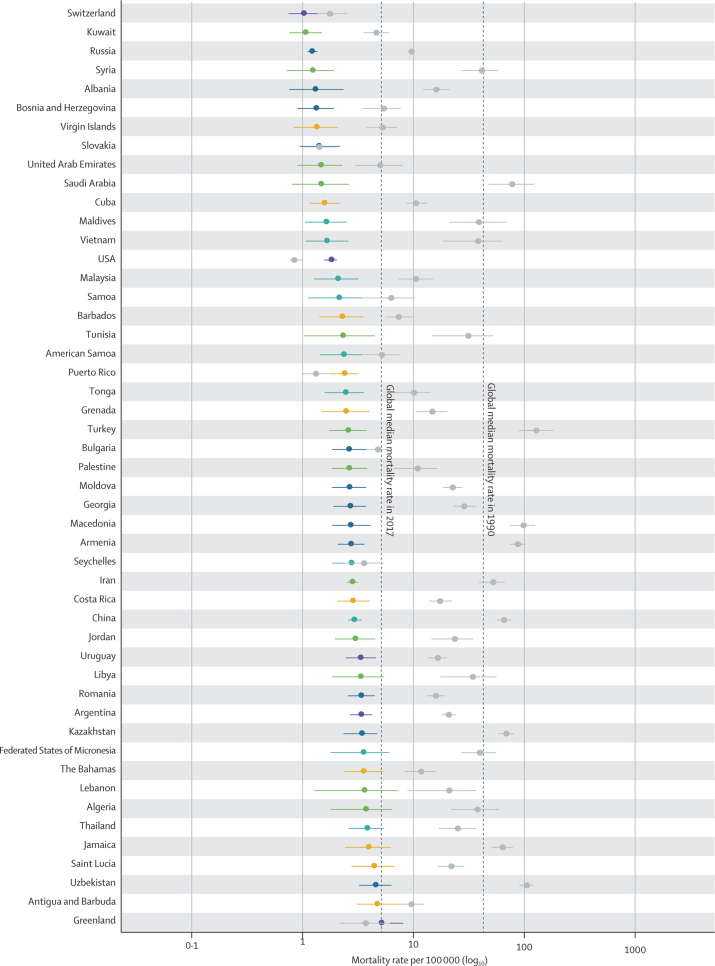

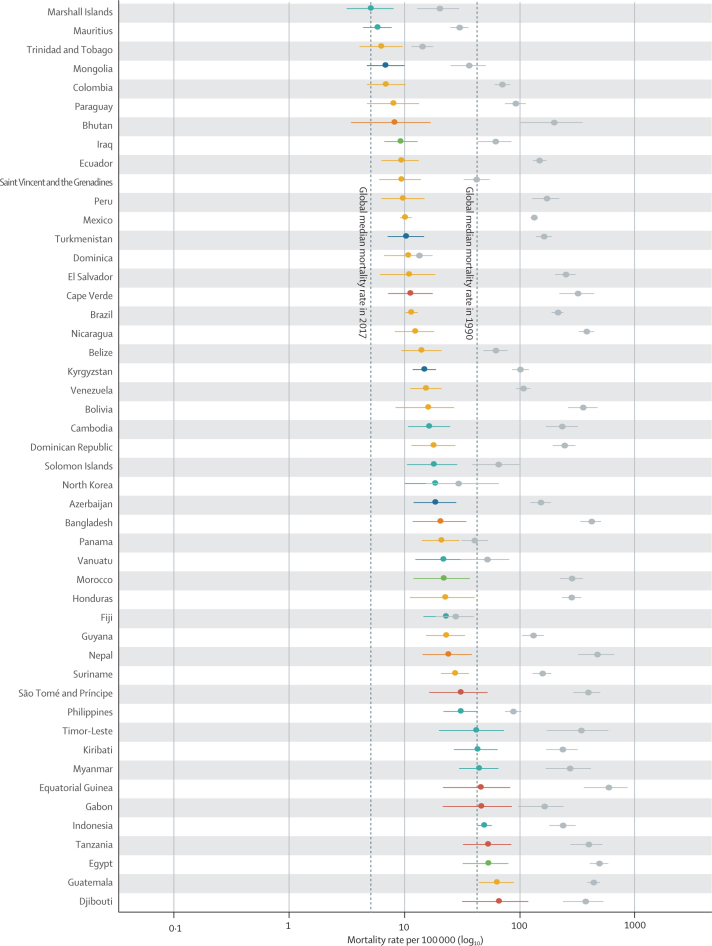

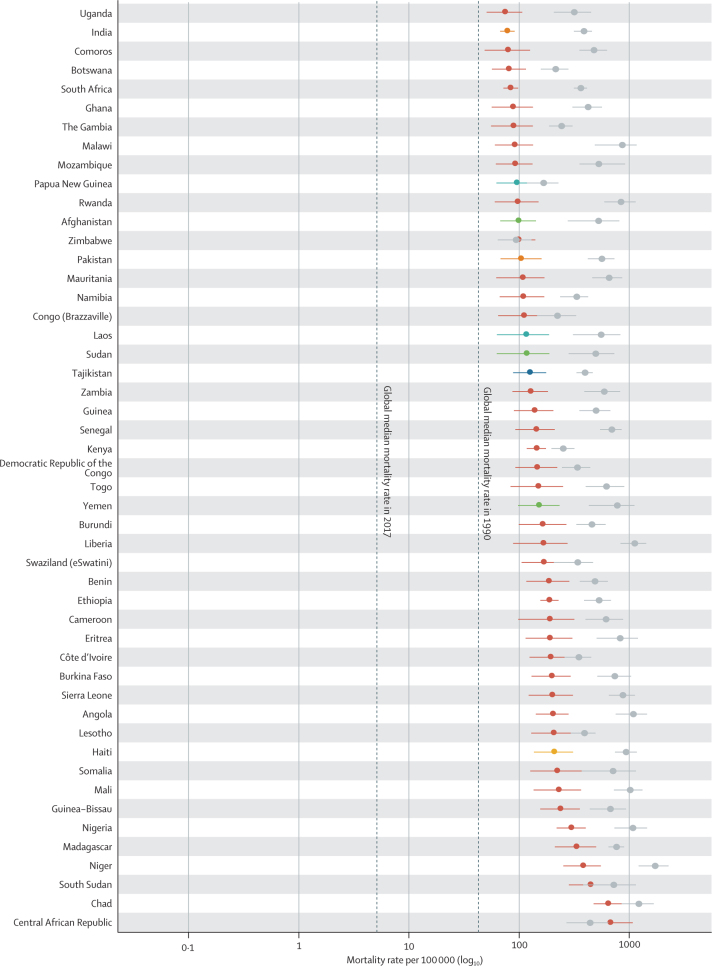
The diarrhoea mortality rate among children younger than 5 years by country, 1990 and 2017 Data are under-5 diarrhoea mortality rate (95% uncertainty interval) in 1990 (gray points) and in 2017 (coloured points). The colours indicate the Global Burden of Diseases, Injuries, and Risk Factors Study super region. Countries are ordered by increasing mortality rate in 2017.

**Figure 2:**
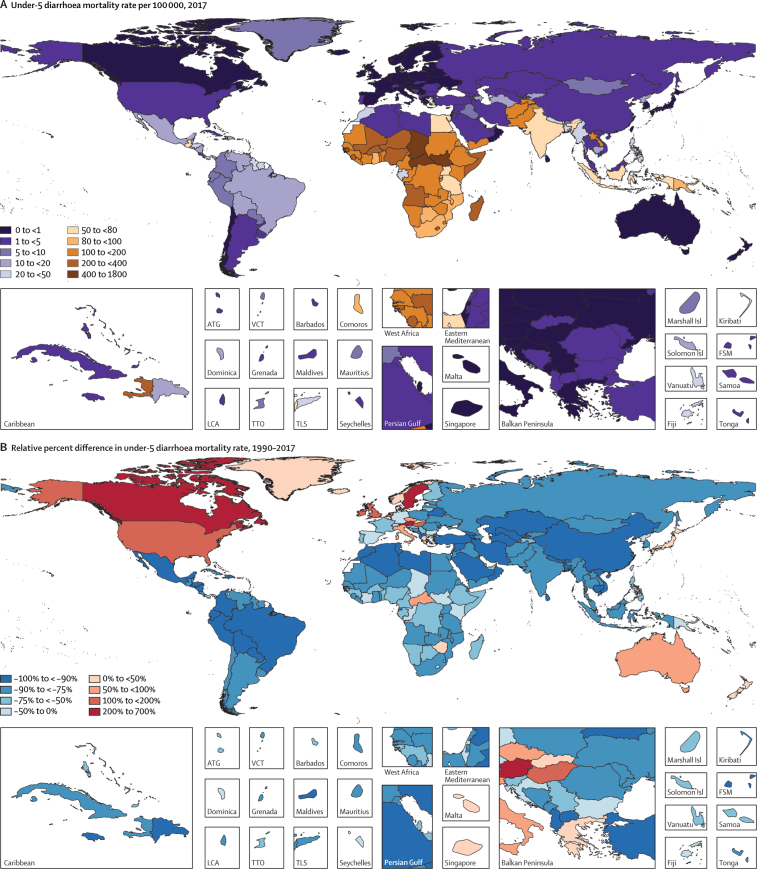

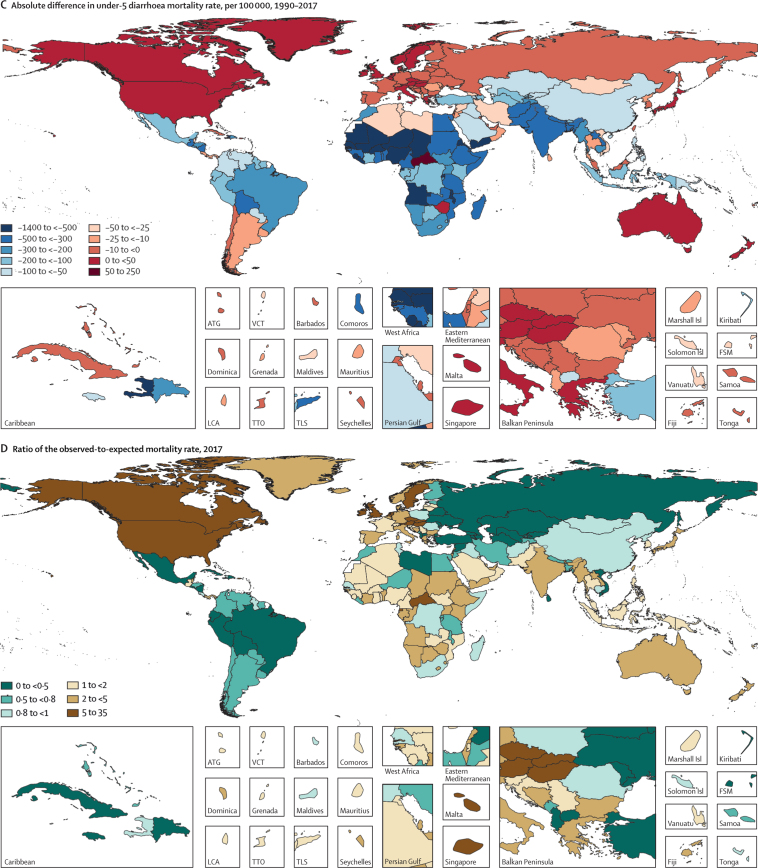
Maps of diarrhoea mortality rate per 100 000 among children younger than 5 years, 1990–2017 (A) Diarrhoea mortality rate per 100 000 children younger than 5 years in 2017. (B) Relative percent difference and (C) absolute difference in diarrhoea mortality rate among children younger than 5 years between 2017 and 1990. (D) Ratio of observed-to-predicted diarrhoea mortality rate per 100 000 (predicted on the basis of the observed change in SDI between 1990 and 2017) in 2017. ATG=Antigua and Barbuda. FSM=Federated States of Micronesia. Isl=Islands. LCA=Saint Lucia. SDI=Socio-demographic Index. TLS=Timor-Leste. TTO=Trinidad and Tobago. VCT=Saint Vincent and the Grenadines.

In 2017, diarrhoea was responsible for an estimated 533 768 deaths (95% uncertainty interval [UI] 477 162–593 145) among children younger than 5 years, globally (table), accounting for 9·9% (8·9–10·9) of all under-5 childhood deaths. Together, India (102 678 deaths, 87 608–118 510) and Nigeria (104 267 deaths, 75 975–139 594) accounted for more than a third of all diarrhoea deaths (appendix pp 58–75). To increase the comparability of results across locations with radically different population sizes, the remaining results focus on mortality rate. Diarrhoea mortality rate among children younger than 5 years was 78·4 deaths (70·1–87·1) per 100 000 children, globally, and ranged from 0·1 deaths (0·1–0·1) per 100 000 children in Singapore to 685·8 (385·7–1082·0) per 100 000 children in the Central African Republic (Figure 1, Figure 2; appendix pp 58–75). The regions with the highest diarrhoea mortality rates were western sub-Saharan Africa (269·3 deaths, 219·1–328·9, per 100 000 children) and central sub-Saharan Africa (176·1 deaths, 130·6–233·9, per 100 000 children; table; figure 2A).

**Table:** Mortality from diarrhoeal diseases and associated risk factors by GBD super-region and region, 2017

	**Deaths (95% UI)**	**Mortality rate per 100 000 (95% UI)**	**Percent change in mortality rate (95% UI), 1990 to 2017**	**Absolute difference in mortality rate per 100 000 (95% UI), 1990 to 2017**	**Deaths per 100 000 episodes (95% UI)**	**Attributable fraction for nutrition-associated risks (95% UI)**	**Attributable fraction for low rotavirus vaccine coverage (95% UI)**	**Attributable fraction for unsafe WASH (95% UI)**	**Attributable fraction low ORS coverage (95% UI)**	**Attributable fraction for all risks (95% UI)**
**Global**	**533 768 (477 162 to 593 145)**	**78·4 (70·1 to 87·1)**	**–69·6% (−74·6 to −63·1)**	**–179·5 (−207·1 to −149·2)**	**48·2 (44·5 to 51·5)**	**88·5% (81·3 to 92·4)**	**22·0% (16·7 to 27·9)**	**94·0% (85·9 to 98·1)**	**57·7% (39·9 to 70·8)**	**99·4% (98·7 to 99·8)**
**Central Europe, eastern Europe, and central Asia**	**2395 (1907 to 3032)**	**8·6 (6·8 to 10·8)**	**–78·6% (−83·2 to −72·8)**	**–31·4 (−31·8 to −30·5)**	**6·5 (6·5 to 6·6)**	**85·3% (75·4 to 90·8)**	**8·0% (5·0 to 12·2)**	**82·5% (66·8 to 93·3)**	**51·1% (33·7 to 65·0)**	**97·7% (95·4 to 99·1)**
Central Asia	2187 (1703 to 2812)	22·8 (17·8 to 29·3)	–82·2% (−86·4 to −76·8)	–105·1 (−108·4 to −100·5)	24·3 (23·2 to 25·4)	86·2% (76·8 to 91·5)	6·5% (4·0 to 10·3)	85·0% (69·3 to 94·8)	50·5% (33·1 to 64·6)	98·3% (96·3 to 99·4)
Central Europe	70 (61 to 82)	1·2 (1·1 to 1·4)	–83·4% (−86·4 to −79·4)	–6·2 (−7·0 to −5·6)	0·7 (0·7 to 0·8)	79·1% (65·2 to 86·6)	28·6% (18·8 to 40·0)	47·0% (26·6 to 68·9)	58·7% (40·9 to 71·9)	90·4% (84·0 to 95·0)
Eastern Europe	138 (122 to 154)	1·1 (1·0 to 1·2)	–87·3% (−88·8 to −85·8)	–7·4 (−7·9 to −7·0)	0·8 (0·7 to 0·8)	74·5% (60·7 to 81·8)	31·4% (20·2 to 44·5)	62·2% (43·7 to 79·4)	56·3% (38·4 to 69·7)	92·2% (86·3 to 96·2)
**High-income**	**706 (618 to 769)**	**1·2 (1·1 to 1·3)**	**–44·0% (−52·9 to −35·7)**	**–1·0 (−1·1 to −0·9)**	**1·4 (1·2 to 1·7)**	**69·3% (53·4 to 79·0)**	**10·6% (6·0 to 16·5)**	**19·6% (11·6 to 30·0)**	**57·1% (39·6 to 70·2)**	**77·0% (67·2 to 83·6)**
Australasia	12 (8 to 17)	0·7 (0·5 to 0·9)	61·1% (−4·1 to 143·1)	0·3 (0·1 to 0·4)	1·4 (1·3 to 1·5)	66·8% (47·3 to 78·6)	4·1% (1·8 to 8·2)	22·3% (10·3 to 40·8)	56·9% (39·0 to 70·1)	75·6% (63·8 to 83·8)
High-income Asia Pacific	53 (45 to 61)	0·7 (0·6 to 0·8)	–43·5% (−56·0 to −28·5)	–0·5 (−0·7 to −0·5)	0·9 (0·8 to 1·0)	72·0% (55·8 to 81·0)	16·1% (10·3 to 23·2)	21·7% (10·3 to 40·3)	56·6% (38·5 to 69·8)	81·1% (70·4 to 87·9)
High-income North America	371 (314 to 410)	1·7 (1·5 to 1·9)	125·3% (76·4 to 164·1)	1·0 (0·8 to 1·0)	3·3 (2·7 to 3·8)	66·9% (50·4 to 77·7)	7·9% (3·8 to 13·6)	11·6% (7·1 to 17·9)	56·9% (39·1 to 70·2)	72·4% (61·5 to 79·8)
Southern Latin America	141 (114 to 170)	2·8 (2·2 to 3·3)	–83·4% (−87·1 to −79·0)	–13·9 (−15·3 to −12·6)	0·9 (0·9 to 0·9)	76·4% (61·2 to 85·2)	10·1% (5·2 to 16·7)	49·6% (26·5 to 74·4)	57·8% (40·2 to 71·6)	89·6% (82·3 to 95·2)
Western Europe	129 (114 to 146)	0·6 (0·5 to 0·7)	–25·0% (−44·2 to −6·8)	–0·2 (−0·3 to −0·2)	0·9 (0·8 to 1·0)	69·1% (52·5 to 79·1)	18·5% (11·9 to 26·8)	8·9% (5·1 to 15·0)	56·7% (39·0 to 70·0)	74·9% (63·5 to 81·6)
**Latin America and Caribbean**	**9904 (8527 to 11 490)**	**19·5 (16·7 to 22·6)**	**–90·0% (−91·5 to −88·1)**	**–175·6 (−185·4 to −165·7)**	**10·4 (9·8 to 10·9)**	**79·4% (65·6 to 87·3)**	**8·0% (4·9 to 12·1)**	**82·0% (67·5 to 92·1)**	**55·7% (37·6 to 69·1)**	**96·2% (92·6 to 98·5)**
Andean Latin America	754 (560 to 1 003)	11·2 (8·4 to 15·0)	–94·5% (−96·0 to −92·4)	–193·7 (−227·1 to −163·8)	5·6 (4·8 to 6·5)	70·4% (53·3 to 81·4)	5·1% (2·7 to 8·7)	77·0% (54·8 to 91·3)	60·3% (42·0 to 73·0)	93·5% (86·7 to 97·7)
Caribbean	3513 (2358 to 5044)	89·8 (60·3 to 128·9)	–72·4% (−82·1 to −57·9)	–236·1 (−262·3 to −212·3)	48·1 (40·2 to 55·6)	83·9% (70·9 to 91·2)	6·9% (4·4 to 10·0)	95·8% (88·9 to 98·9)	53·7% (35·3 to 67·7)	99·5% (98·6 to 99·9)
Central Latin America	3781 (3269 to 4428)	15·6 (13·5 to 18·3)	–89·9% (−91·4 to −88·0)	–138·4 (−143·5 to −132·7)	7·1 (6·8 to 7·5)	76·3% (62·5 to 84·9)	10·5% (6·3 to 16·3)	74·4% (55·8 to 88·3)	58·7% (41·1 to 71·5)	94·6% (89·7 to 97·8)
Tropical Latin America	1856 (1647 to 2087)	11·5 (10·2 to 13·0)	–94·6% (−95·4 to −93·6)	–202·7 (−225·0 to −178·9)	8·7 (7·7 to 9·8)	79·8% (64·7 to 88·2)	2·2% (0·8 to 4·9)	73·6% (56·7 to 87·7)	51·7% (33·6 to 66·5)	94·5% (89·9 to 97·6)
**North Africa and Middle East**	**28 962 (23 106 to 35 611)**	**45·0 (35·9 to 55·3)**	**–80·7% (−84·8 to −75·3)**	**–188·5 (−227·8 to −149·5)**	**17·9 (16·7 to 18·7)**	**88·0% (78·6 to 92·8)**	**17·2% (11·0 to 25·1)**	**87·0% (74·9 to 94·9)**	**61·2% (43·6 to 73·6)**	**98·3% (96·5 to 99·4)**
**South Asia**	**135 390 (115 688 to 156 734)**	**77·6 (66·3 to 89·9)**	**–81·4% (−85·8 to −76·1)**	**–339·4 (−397·7 to −282·5)**	**74·8 (71·6 to 77·1)**	**89·6% (84·1 to 92·9)**	**16·9% (10·9 to 24·5)**	**92·9% (82·9 to 97·9)**	**53·5% (36·1 to 67·7)**	**99·6% (99·1 to 99·9)**
**Southeast Asia, east Asia, and Oceania**	**22 105 (19 881 to 24 679)**	**15·6 (14·0 to 17·4)**	**–83·1% (−85·5 to −79·9)**	**–76·7 (−88·6 to −65·6)**	**10·2 (9·1 to 11·3)**	**88·1% (79·8 to 92·1)**	**29·5% (20·7 to 39·8)**	**85·7% (66·4 to 95·6)**	**54·8% (37·1 to 68·1)**	**98·6% (96·8 to 99·6)**
East Asia	2645 (2309 to 3198)	3·1 (2·7 to 3·8)	–95·1% (−95·9 to −94·1)	–60·5 (−70·9 to −52·3)	2·4 (2·2 to 2·6)	77·3% (61·4 to 86·2)	9·9% (4·5 to 19·7)	77·4% (58·2 to 91·2)	55·5% (37·8 to 68·7)	95·1% (89·8 to 98·2)
Oceania	1450 (961 to 2127)	81·5 (54·0 to 119·6)	–37·3% (−60·7 to 0·0)	–48·5 (−52·2 to −41·3)	27·4 (22·9 to 31·9)	88·1% (80·5 to 93·0)	21·5% (13·9 to 31·0)	94·8% (86·7 to 98·6)	61·7% (44·0 to 74·5)	99·5% (98·8 to 99·9)
Southeast Asia	18 009 (15 924 to 20 389)	32·4 (28·6 to 36·7)	–79·3% (−83·3 to −73·7)	–123·9 (−152·3 to −96·8)	18·0 (17·0 to 19·1)	89·5% (82·3 to 93·0)	33·3% (23·8 to 44·5)	86·2% (66·0 to 95·9)	54·1% (36·4 to 67·5)	99·0% (97·4 to 99·7)
**Sub-Saharan Africa**	**334 306 (285 351 to 388 790)**	**204·6 (174·7 to 238·0)**	**–68·4% (−74·2 to −60·3)**	**–443·0 (−523·8 to −352·9)**	**91·3 (91·0 to 91·4)**	**88·5% (80·6 to 92·6)**	**24·4% (19·2 to 30·0)**	**96·2% (90·1 to 99·0)**	**59·5% (41·7 to 72·4)**	**99·7% (99·1 to 99·9)**
Central sub-Saharan Africa	34 800 (25 798 to 46 206)	176·1 (130·6 to 233·9)	–63·5% (−73·9 to −49·3)	–306·9 (−364·9 to −246·9)	59·5 (52·6 to 67·7)	88·6% (79·2 to 93·4)	39·8% (32·0 to 47·2)	96·5% (91·2 to 99·1)	60·5% (42·2 to 73·4)	99·7% (99·2 to 99·9)
Eastern sub-Saharan Africa	98 175 (84 620 to 114 013)	155·1 (133·7 to 180·1)	–70·3% (−76·4 to −62·3)	–367·1 (−446·7 to −287·5)	72·2 (69·7 to 75·4)	86·7% (78·5 to 91·2)	13·4% (8·7 to 19·0)	96·7% (91·2 to 99·2)	58·9% (41·1 to 71·5)	99·7% (99·1 to 99·9)
Southern sub-Saharan Africa	8 070 (6 972 to 9 314)	94·5 (81·7 to 109·1)	–67·9% (−73·3 to −61·4)	–200·1 (−223·6 to −173·6)	85·4 (85·4 to 86·6)	81·5% (68·3 to 88·6)	7·1% (4·1 to 11·1)	89·0% (78·3 to 96·0)	53·2% (35·2 to 66·7)	98·3% (96·2 to 99·4)
Western sub-Saharan Africa	193 260 (157 286 to 236 075)	269·3 (219·1 to 328·9)	–70·1% (−76·9 to −60·2)	–630·4 (−785·7 to −480·1)	119·0 (111·2 to 126·9)	89·8% (82·2 to 93·6)	29·2% (23·7 to 35·0)	96·1% (89·9 to 99·0)	59·9% (41·0 to 72·9)	99·7% (99·2 to 99·9)

WASH=water, sanitation, and hygiene. ORS=oral rehydration solution. GBD=Global Burden of Diseases, Injuries, and Risk Factors Study. UI=uncertainty interval.

Diarrhoea mortality rate per 100 000 children decreased by 69·6% (95% UI 63·1–74·6) between 1990 and 2017, globally (table; figure 1). The greatest relative decline in diarrhoea mortality rate occurred in Saudi Arabia (98·1% decrease [95·9–99·1]; from 77·1 deaths [47·1–122·1] per 100 000 children to 1·5 deaths [0·8–2·6] per 100 000 children; figure 1; appendix pp 58–75). Diarrhoea mortality rate increased in 25 countries (figure 2) but its absolute change in these locations was small during this time (table; figure 1). The greatest absolute decline in diarrhoea mortality rate occurred in Niger (1344·2 fewer deaths [969·9–1735·1] per 100 000 children between 1990 and 2017; from 1731·2 deaths [1223·8–2290·9] per 100 000 children in 1990, to 387·8 deaths [253·9–555·9] per 100 000 children in 2017; Figure 1, Figure 2; appendix pp 58–75).

Nine countries (Armenia, China, Iran, Jamaica, Kazakhstan, Macedonia, Saudi Arabia, Turkey, and Uzbekistan) had mortality rates above the global median rate in 1990 and below the global median rate in 2017. These countries had steep increases in SDI during this time (appendix pp 91–93). Diarrhoea mortality rate decreased by 98·1% (95% UI 95·9–99·1) in Saudi Arabia, as reported above, by 98·0% (96·5–98·8) in Turkey (from 127·5 deaths [88·1–181·5] per 100 000 children to 2·6 deaths [1·7–3·7] per 100 000 children), and by 95·5% (94·6–96·4) in China (from 65·0 deaths [56·3–75·9] per 100 000 children to 2·9 deaths [2·5–3·4] per 100 000 children). Some of these countries had diarrhoea mortality rates much lower than expected on the basis of SDI (appendix pp 58–75). For example, the ratio of observed-to-expected mortality rates in Armenia (0·17), Georgia (0·16), and Macedonia (0·12) were the three lowest globally, suggesting the mortality rate in these countries is much lower than expected based on SDI (figure 2D). By contrast, the ratios of observed-to-expected mortality rate in China (0·83) and Saudi Arabia (1·03) were closer to 1, suggesting a correlation between mortality rate and SDI in these locations. The observed mortality rate was much greater than expected based on SDI in some high-income locations (eg, Canada [18·2] and Austria [12·4], which were the countries with the highest ratios, globally) as well as in low-income locations such as some countries in sub-Saharan Africa (eg, Equatorial Guinea [11·0], Central African Republic [5·8], and Sudan [4·7]; figure 2D).

**Figure 3:**
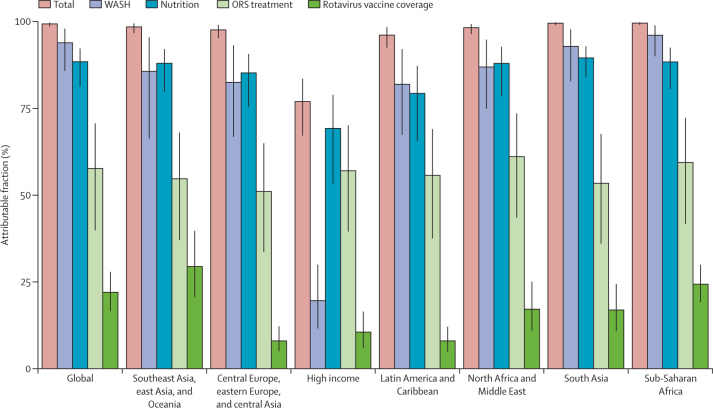
Aggregated attributable fractions for diarrhoeal risk factors among children younger than 5 years by GBD super-region, 2017 In the comparative risk factors framework used in GBD 2017, risk factors are counterfactual and can overlap such that a single risk might be sufficient, but is not necessary, to cause a diarrhoea death. Therefore, although the total risk attribution cannot exceed 1, there could be overlap between the risk factors associated with rotavirus vaccine coverage, ORS treatment, nutrition, or WASH at the population level, such that eliminating exposure to one would avert a diarrhoea death. ORS=oral rehydration solution. WASH=water, sanitation, and hygiene.

The risk factors included in GBD 2017 for diarrhoea among children younger than 5 years accounted for 99·4% (95% UI 98·7–99·8) of diarrhoea deaths in 2017 (table; figure 3). Full use of the rotavirus vaccine could have prevented an estimated 22·0% (16·7–27·9) of diarrhoea deaths and providing safe water, sanitation, and hygiene (WASH) could have averted 94·0% (85·9–98·1) of deaths, globally (table; figure 3). Risk factors related to poor childhood nutrition, including growth failure and zinc and vitamin A deficiencies were responsible for 88·5% (81·3–92·4) of diarrhoea deaths in 2017, and full coverage of use of oral rehydration solution could have prevented 57·7% (39·9–70·8) of diarrhoea deaths in 2017 (table; figure 3). Poor childhood nutrition and unsafe WASH were attributed to a similar fraction of deaths (>75%) in most GBD super-regions, with the exception of the high-income super-region, where poor nutrition was responsible for 69·3% (53·4 to 79·0) and unsafe WASH for only 19·6% (11·6 to 30·0) of deaths (table; figure 3). When grouped into quintiles, countries with the lowest SDI in 2017 had much greater risk attribution than countries in the highest SDI, particularly for unsafe WASH (96·5% [90·9–99·1] for countries in the lowest SDI *vs* 13·0% [7·2–22·0] for countries in the highest SDI; appendix p 94).

**Figure 4:**
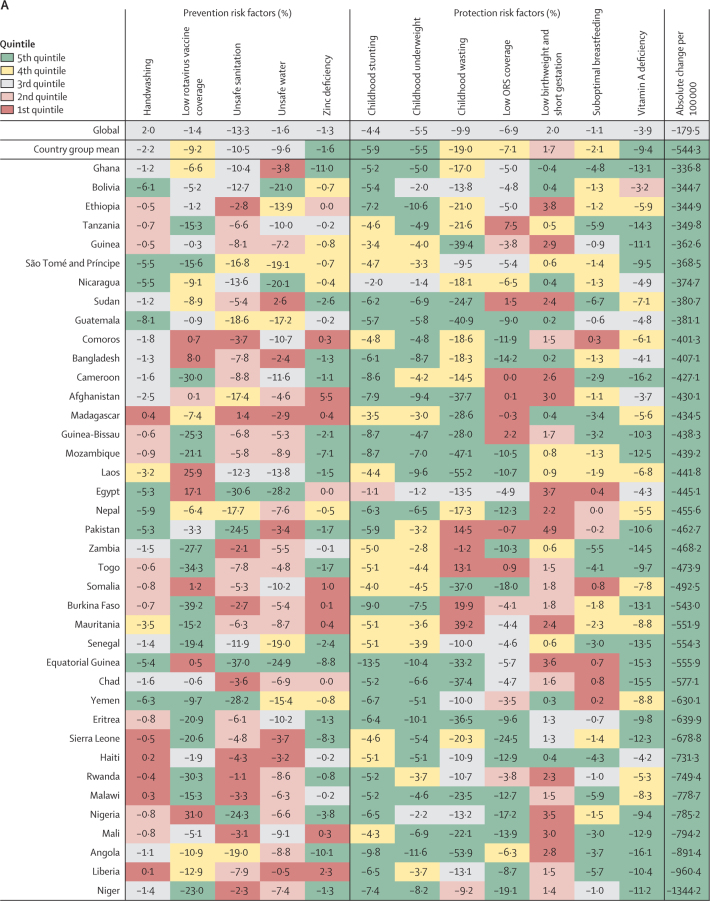

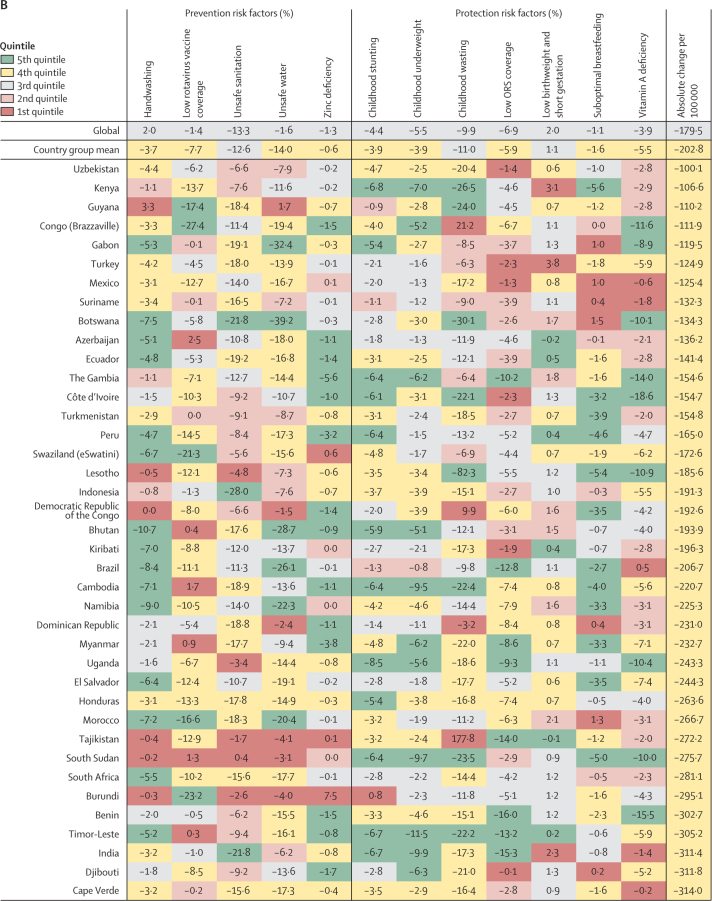

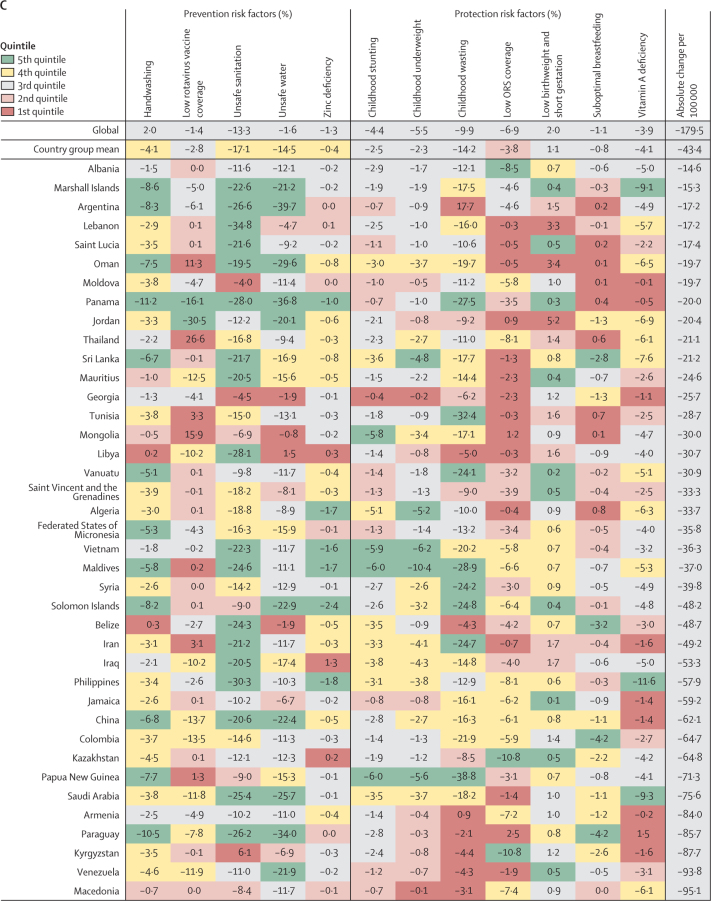

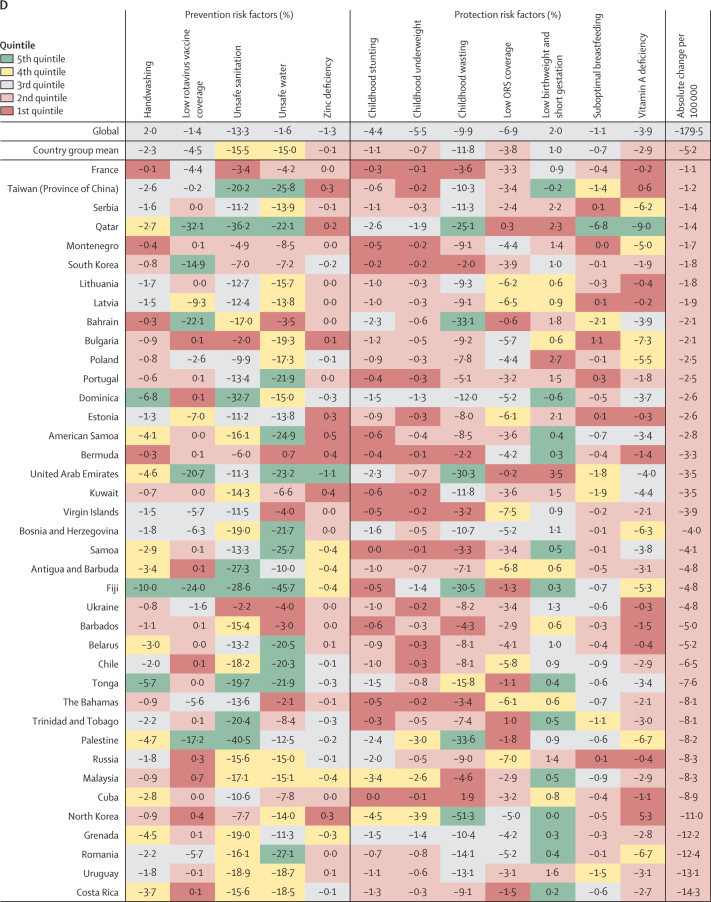

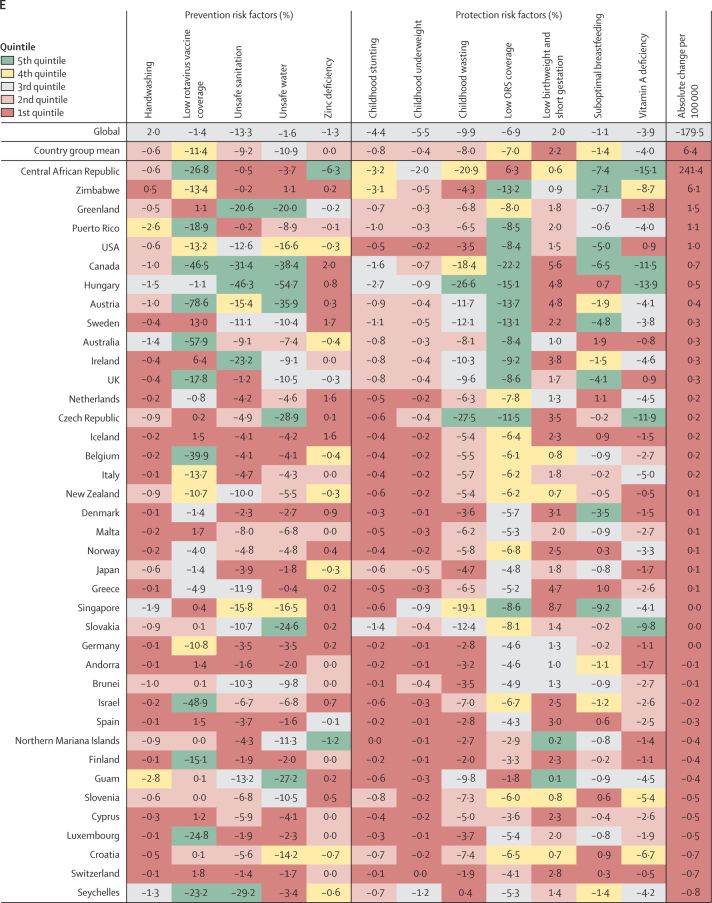
Percent change in the diarrhoea mortality rate attributable to changes in risk factor exposure by country, grouped in quintiles of the absolute attributable change in mortality rate, 1990–2017 Data are percent reduction in diarrhoea mortality rate between 1990 and 2017 attributable to changes in exposure to each risk factor. The first row represents the value among all countries and the second row represents the mean value among countries in each country group: (A) 5th, (B) 4th, (C) 3rd, (D) 2nd, and (E) 1st quintile of the absolute attributable change in mortality rate between 1990 and 2017. The colours of the tiles indicate the quintile for the attributable change in mortality due to each risk factor among all countries. ORS=oral rehydration solution.

A decomposition analysis of the percent change in diarrhoea mortality between 1990 and 2017 due to changes in risk factor attribution is shown in figure 4; countries are grouped by quintile of absolute decrease in diarrhoea mortality rate (from the largest decrease [5th quintile] to the smallest decrease [1st quintile]). Globally, factors responsible for the greatest reductions in the diarrhoea mortality rate were decreases in unsafe sanitation (13·3% decrease, 95% UI 11·2–15·5), childhood wasting (9·9% decrease, 9·6–10·2), and low use of oral rehydration solution (6·9% decrease, 4·8–8·4; figure 4; appendix pp 76–90).

Among the countries grouped in the 5th quintile (figure 4A), improvements in risk factors associated with undernutrition were correlated with the largest declines in diarrhoea mortality rate, including improvements in childhood wasting (19·0% mean decrease, −15·0 to 47·8), childhood stunting (5·9% mean decrease, 3·3 to 9·1), childhood underweight (5·5% mean decrease, 1·9 to 10·4), vitamin A deficiency (9·4% mean decrease, 4·1 to 15·6), and zinc deficiency (1·6% decreased attribution, −1·1 to 9·0). Expanded use of oral rehydration solution reduced diarrhoea mortality by 7·1% (−1·6 to 18·1) on average in these countries (figure 4A). The greatest improvements in childhood wasting were seen in Laos (55·2% decrease, 50·2–52·4) and Angola (53·9% decrease, 45·5–59·1; figure 4A). Many countries in this group greatly reduced diarrhoea mortality by expanding use of oral rehydration solution (eg, the greatest reduction was in Sierra Leone [24·5% decrease, 16·9–29·8]; figure 4A). Increased coverage of rotavirus vaccine reduced diarrhoea mortality rate by substantial amounts among some countries that have introduced the vaccine, such as by 39·2% (22·4–73·7) in Burkina Faso and by 34·3% (19·3–63·2) in Togo (figure 4A). Among countries in this group, the decrease in diarrhoea mortality rate was not explained by large decreases in unsafe WASH exposures except for in a few countries, such as Equatorial Guinea and Egypt, where reductions in these risk factors substantially reduced diarrhoea mortality rate (figure 4A; appendix pp 76–90).

Countries in the fourth quintile (second fastest) of the absolute change in diarrhoea mortality rate tended to have the decline mostly explained by WASH risks. For example, diarrhoea mortality rate decreased by 28·0% (95% UI 25·7–29·7%) in Indonesia and by 21·8% (18·9–23·8) in India because of sanitation, by 39·2% (27·8–50·2) in Botswana because of increased access to safe water, and by 10·7% (4·8–16·2) in Bhutan because of handwashing (figure 4B; appendix pp 76–90). The mean change in diarrhoea mortality rate in these countries was more evenly explained by childhood growth failure, micronutrition, and WASH risk factors than for countries in the third (figure 4C) and second (figure 4D) quintiles for reduction in diarrhoea mortality rate. China and Saudi Arabia, two of three countries with the largest relative decline in diarrhoea mortality rate, were in the third quintile in absolute decline. A substantial amount of the decline in China was driven by improvements in handwashing (6·8%, 3·0–10·9), unsafe sanitation (20·6%, 19·4–21·0), and unsafe water (22·4%, 14·2–29·8; figure 4C). Countries in the first quintile, with the smallest absolute change in diarrhoea mortality rate, were mainly countries in the high-income GBD super-region. The mortality rate increased because of increased prevalence of low birthweight and short gestational age in this quintile and by as much as 8·7% (8·3–10·3) in Singapore (figure 4E). Although improvements in rotavirus vaccine coverage, suboptimal breastfeeding, vitamin A deficiency, and moderate declines in stunting, underweight, and wasting contributed to reducing diarrhoea mortality in the Central African Republic and Zimbabwe, the net mortality rate increased in these two locations, potentially because of other unmeasured factors (241·4 additional deaths [114·6–430·5] per 100 000 children in the Central African Republic and 6·1 additional deaths [1·8–11·1] per 100 000 children in Zimbabwe; appendix pp 76–90). Overall, the diarrhoea mortality rate increased in 23 countries, most of which were in the GBD high-income super region (figure 4E; appendix pp 58–75).^16^

## Discussion

Diarrhoea mortality among children younger than 5 years is largely preventable with existing interventions that reduce exposure to pathogens and reduce the risk of mortality. Our estimates from this and previous iterations of GBD have consistently shown that diarrhoea is one of the leading causes of death in this age group.^2^, ^3^ There is reason to be optimistic about the observed decline in mortality. But this trend should not lull public health officials, policy makers, and funding agencies into a sense that persistence of such declines is inevitable; rather, it should press all actors into asking why the pace of decline has been unequal across countries. The analyses presented here attempt to answer some of this most important question.

The SDI increased in every country between 1990 and 2017.^1^ The greatest increase in SDI between 1990 and 2017 occurred in Equatorial Guinea, a country where diarrhoea mortality decreased by more than 550 deaths per 100 000 children during that time period. However, the two countries with the greatest absolute decline in diarrhoea mortality rates, Niger and Liberia, had increases in SDI that were smaller than the global average.^1^ The SDI indicator—a composite of income, education, and fertility—appears to be highly correlated with health outcomes but might struggle to capture rapid changes in living conditions within a country, such as the start or end of internal conflict. We have previously shown that the SDI is a strong predictor of diarrhoea mortality, but there are locations where difference between the mortality rate and the rate predicted on the basis of changes in SDI is large.^2^ An important contrast becomes apparent since the countries with the greatest relative decline in diarrhoea mortality rate (such as China, Turkey, and Saudi Arabia) appear to be correlated with increases in SDI, whereas the countries with the greatest absolute decline in diarrhoea mortality rate have declines that exceed those predicted by SDI. Increasing development is related to greater ability for countries to build and maintain integrative surveillance and treatment programmes. For example, starting in the early 1990s, China implemented a series of national programmes to reduce under-5 mortality, including the Program for Control of Diarrheal Diseases that focused on surveillance, health education, training, and access to health care for all counties, cities, and provinces with emphasis on rural areas.^17^

Regardless of distinguishing between relative and absolute change, countries with the largest declines in diarrhoea mortality rate tended to have large reductions in childhood underweight and stunting attribution. There have been substantial reductions in childhood growth failure from 1990 to 2017, but with persistent subnational variation.^16^ Our results show that improving the nutritional status of children is the single most important intervention to prevent infectious disease mortality. To a smaller extent, micronutrient deficiencies such as vitamin A and zinc deficiencies have improved in countries with either the fastest relative or absolute change. Childhood growth failure, including stunting, underweight, and wasting, appear to have a nearly linear relationship with SDI.^13^ The dietary zinc-deficiency risk factor reported in this Article is distinct from zinc therapy, which has been shown to substantially reduce diarrhoea duration and possibly mortality among children.^18^ Diarrhoeal episodes among children are associated with impaired growth, and accounting for growth failure could increase the overall burden of diarrhoea by 40%.^19^, ^20^ Overall, given the range of additional health outcomes,^21^ the efficacy,^10^ and the cost effectiveness of nutritional interventions, they should not be overlooked when considering strategies to reduce child vulnerability to diarrhoeal diseases.^22^

In the past two decades, researchers, policy makers, and clinicians have espoused the biologically plausible idea that reducing environmental contamination through WASH might be key to tackling the persistent challenge of childhood diarrhoea and stunting. However, at least three large trials^10^, ^23^, ^24^ have reported no effect of WASH combined interventions on child linear growth and showed little benefit due to safe water and mixed protective benefits for sanitation and handwashing for childhood diarrhoea. Although these studies focused on family-level or neighbourhood-level interventions, some evidence points to community-level contamination of water sources even when some members of the community are using safe water and sanitation or that household point-of-use water purification interventions might be effective in such communities with high prevalence of unsafe sanitation.^25^ Provision of universal safe water and sanitation requires substantial infrastructural, which might not be feasible in some locations.^26^, ^27^ A 2018 report^28^ concluded that many countries are not on pace to meet targets for universal access to safe water and sanitation introduced in the Sustainable Development Goals. Poor access to safe water and sanitation persists particularly among rural and poor populations.^28^ Sanitation had the biggest effect on diarrhoea mortality among all risk factors (13·3% decrease, 95% UI 11·2–15·5), but this change appeared to be greatest in locations with middle values of SDI (0·4–0·6).^13^ Countries with the greatest relative change in diarrhoea mortality rate (such as China, Turkey, and Saudi Arabia) had steep reductions due to decreases in exposure to unsafe WASH. This trend was not observed among most of the countries with the fastest absolute reduction in diarrhoea mortality rate, where the change in attribution for the WASH risks were often in the lowest two quintiles of change among all countries.

Many countries have an SDI that is far from the range at which water and sanitation are likely to improve rapidly. Countries at the lower range of SDI that had the greatest declines in absolute mortality rates tended to succeed at reducing attribution to risks that act at the individual level. For example, some of the countries with the largest absolute declines in diarrhoea mortality rates (eg, Niger, Nigeria, Sierra Leone, and Somalia) had more than 15% reduction in diarrhoea mortality due to increased use of oral rehydration solution. Treatment with oral rehydration solution or with recommended home fluids is inexpensive and effective, perhaps explaining why oral rehydration solution decreased mortality in locations with a quite small change in SDI. Treatment with oral rehydration solution could prevent 93% of diarrhoea deaths,^11^ but appropriate use remains low in many locations, perhaps because in some of these locations oral rehydration solutions are perceived as medication that must be prescribed by health-care workers (and hence might appear difficult to access), adherence to treatment is low, and alternative treatments (including antibiotics) are considered western medicine and thus are perceived as superior and preferentially used.^29^

Globally, 34·7% of under-5 diarrhoea deaths were caused by rotavirus.^1^ Existing rotavirus vaccines prevented about 30 000 deaths in 2016.^30^ Some of the poorest countries are eligible for support from Gavi, the Vaccine Alliance in funding rotavirus vaccine use but have not introduced the vaccine, such as the Democratic Republic of the Congo, Nigeria, Chad, and South Sudan.^31^ These countries also had slower reductions in diarrhoea mortality than neighbouring countries that did introduce the vaccine, such as Rwanda, Niger, and Sudan. Countries in Latin America and the Caribbean were early adopters of the rotavirus vaccine and several countries have substantially reduced diarrhoea mortality due to rotavirus. There remains some uncertainty about the future of the rotavirus vaccine in low-to-middle SDI countries that have or will soon transition from being eligible for Gavi support. Introduction and coverage of the rotavirus vaccine, particularly among high-mortality countries, can avert a substantial amount of the diarrhoea deaths attributable to rotavirus.

There are several limitations in this study. Our estimates are dependent on data availability, which tends to be scarce, particularly among countries with high mortality in sub-Saharan Africa. Detailed, reliable, and timely data on disease burden are needed for countries to make informed decisions about health policy and interventions. Global burden estimates attempt to fill some of these gaps but have substantial uncertainty (which we have attempted to report consistently) and uncertainty is propagated through our modelling process. Previous criticism of the GBD diarrhoea estimates highlights both insufficient and conflicting data, including different requirements for using data on mortality and aetiologies. A principle of the GBD is to use all available, representative, and reliable data, which sometimes leads to global burden estimates based on different underlying data, a source of some differences between such estimates.^32^, ^33^, ^34^ For some risk factors, we assumed that the relative risk of diarrhoea episodes and diarrhoea mortality is the same. This assumption was necessary because of little or no data describing the risk of mortality but it is possible that such an assumption might have biased our estimates. Many of the covariates used in the mortality modelling are also inputs into the risk factor attribution as estimates of population-level exposure to those risks. The risks were included as covariates precisely because they affect diarrhoea mortality. The calculations for the relative risks are independent and do not influence the effect of the covariates in the diarrhoea mortality model.

Although we have quantified treatment with oral rehydration solution for diarrhoea, we did not explicitly account for health-care seeking behaviours, primary health-care availability, and other potential treatments like therapeutic zinc and antibiotic use. The comparative risk assessment framework used for risk factors in GBD estimates risk factors independently. Although this is essential for understanding the counterfactual disease burden associated with a risk, it means that we were unable to assess the potential of combined intervention strategies, such as those that target reductions in childhood underweight through breastfeeding promotion, and such intervention packages are likely to be very effective at reducing disease burden.^35^ We also know that there can be substantial overlap in exposure to risk factors such that an individual child might be both exposed to unsafe water and unvaccinated against rotavirus. Although we did not account for covariance in all risk factors, our approach does allow for the intuitive, actionable interpretation that eliminating exposure to any attributable risk is sufficient to avert a diarrhoea death.

Evaluating trends and disease burden on the national level can mask subnational variation that exists because of different risk factor exposure, access to health care, or other factors. We have shown that childhood growth failure,^16^ diarrhoea mortality,^5^ and under-5 mortality^4^ are all geographically heterogeneous between and within countries in Africa. This analysis was done at a population level, which might hide individual exposures and behaviours. Future work evaluating risks and trends at fine geographical or individual levels and including correlations between risks at this level could better inform targeting of resources for interventions.

This study provides a clear and actionable path for reducing diarrhoea mortality among children younger than 5 years, but this path is likely to differ depending on location (including at a subnational level). Provision of oral rehydration solution for diarrhoeal episodes and the rotavirus vaccine have contributed to declines in even the poorest locations, whereas the provision of universal access to safe water and sanitation might require substantial infrastructural developments. Although less beneficial than some other interventions, vitamin A and zinc supplementation should be considered an essential part of routine childhood health care. Continuing to reduce diarrhoea mortality among children younger than 5 years requires focused effort and intention. Assuming instead that these declines are inevitable, relying on the improvements expected with economic development is not enough; children are dying from diarrhoea now.

## Data sharing

In compliance with the Guidelines for Accurate and Transparent Health Estimates Reporting, data and code for GBD 2017 are publicly available.
