# Correction to *Lancet Infect Dis* 2020; 20: 37–59

**DOI:** 10.1016/S1473-3099(26)00375-0

**Published:** 2026-09

*GBD 2017 Diarrhoeal Disease Collaborators. Quantifying risks and interventions that have affected the burden of diarrhoea among children younger than 5 years: an analysis of the Global Burden of Disease Study 2017.* Lancet Infect Dis *2020;* **20:** 37–59—In this Article, the number of countries and territories where mortality increased should have been 25 throughout. This correction has been made as of July 6, 2026.

