# Supplementary material for: Quantifying risks and interventions that have affected the burden of diarrhoea among children younger than 5 years: an analysis of the Global Burden of Disease Study 2017
Source: Lancet Infect Dis. 2020 Jan;20(1):37–59. doi: 10.1016/S1473-3099(19)30401-3 (PMC7340495; doi:10.1016/S1473-3099(19)30401-3)

THE LANCET Infectious Diseases

Supplementary webappendix

This webappendix formed part of the original submission and has been peer reviewed. We post it as supplied by the authors.

Supplement to: GBD 2017 Diarrhoeal Disease Collaborators. Quantifying risks and interventions that have affected the burden of diarrhoea among children younger than 5 years: an analysis of the Global Burden of Disease Study 2017. *Lancet Infect Dis* 2019; published online Oct 30. https://doi.org/10.1016/S1473-3099(19)30401-3.

Appendix for: *Quantifying risks and interventions to reduce mortality due to diarrhea among children younger than 5 years: An analysis of the Global Burden of Disease study 2017*

This appendix is made up of several parts. The first is a detailed description of diarrhea mortality modeling in the Global Burden of Disease study 2017 (GBD 2017). Diarrhea modeling details are provided on pages 2-6. Following that, a description of input data and modeling strategy for each risk factor or intervention used in the analysis is provided (pages 10-54). Lastly, we have supplementary results for every country.

Diarrhea mortality methodology	2
Risk factor summary descriptive table	7
Risk factor methodology summaries	
Child growth failure	10
Unsafe hygiene	16
Unsafe sanitation	18
Unsafe water	21
Oral rehydration solution	25
Rotavirus vaccine	28
Zinc deficiency	32
Vitamin A deficiency	33
Low birth weight and short gestation	38
Suboptimal breastfeeding	51
References	54
Supplementary country results tables	58
Maps of Socio-demographic Index	76
Bar chart of risk factors by SDI quintile	79

Diarrhea mortality modeling

Modeling diarrhea mortality methods

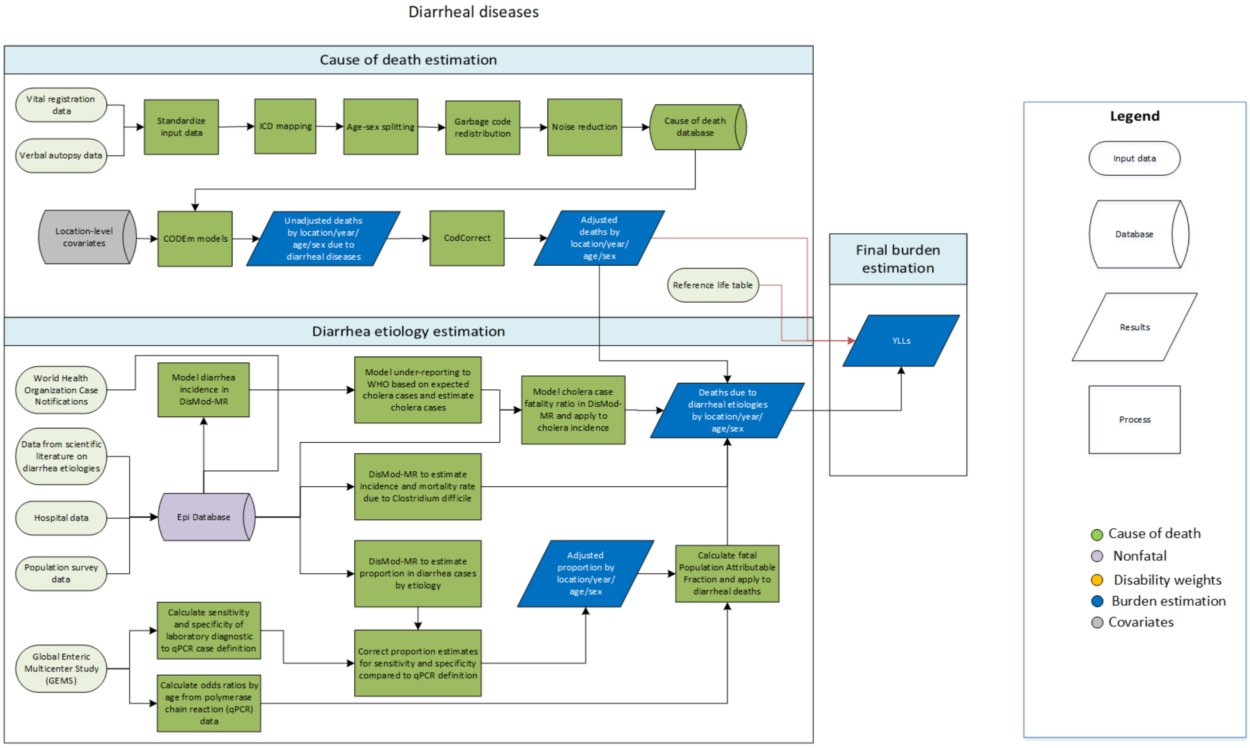

Input data

We used all available data from vital registration systems, surveillance systems and verbal autopsy (**Table 1 and Figure 1**). The International Classification of Diseases (ICD) 9 codes that are used in diarrhea are 001-001.9, 003-006.9, 007.4-007.8, 008.01-008.02, 008.04, 008.2-009.9, and 787.91and ICD 10 codes are A00-A00.9, A02-A04.1, A04.3, A04.5-A07, A07.2-A07.4, A08-A09.9, and R19.7. We checked for and excluded outliers from our data by country or region. We also excluded early neonatal mortality data in the Philippines (1994–1998) and India Civil Registration System data and medically certified cause of death (MCCD) data in all states (1986–2013).

There are three main aspects of cause of death data preparation.[1] The first is age-sex splitting. This is necessary because many data are only available in age ranges or for both sexes and all COD data must be sex-age specific. This occurs after using all data to produce a global age/sex pattern which is then used to proportionally split those data. The next step is garbage code redistribution. This takes deaths that are coded to causes of death that are not part of the GBD hierarchy and assigns them to an appropriate cause of death. A relevant example would be deaths coded to sepsis. This is a non-specific cause of death. A series of regressions determines the proportion of these garbage codes that should be reassigned to each cause. The last step is noise reduction

which is essentially a way to move data points up or down in magnitude based on regional trends. The amount of change is dependent on the data variance. Those points with greater variance are subject to be moved more than those with lesser variance. Data points from verbal autopsy studies and data points that are not nationally representative have variance that is inflated to account for these issues.

Table 1. Cause-specific mortality input data.

**Input data**	**GBD 2017**
**Total data sources**	**19,665 geography-years**
Vital registration data	17,734 geography-years
Sample registration data	740 geography-years
Verbal autopsy data	1,042 geography-years
Surveillance data	509 geography-years

Figure 1. Number of geography-years of mortality data used in diarrhoea mortality modelling.

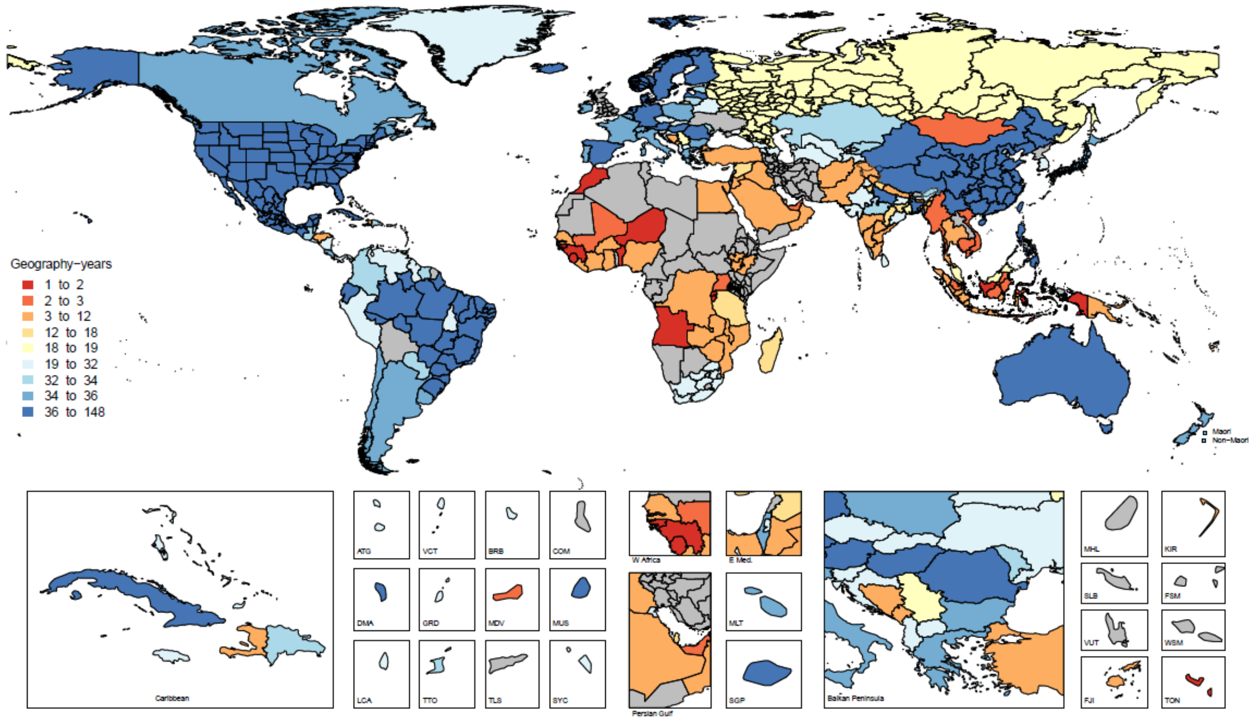

Modeling strategy

Diarrheal disease mortality was estimated in the Cause of Death Ensemble modeling platform (CODEm). We estimated diarrhea mortality separately for males and females and for children under 5 years and older than 5 years. We used country-level covariates to inform our CODEm models (**Table 2**). We evaluated our diarrheal disease cause of death models using in and out of sample predictive performance.

CODEm is a Bayesian statistical model and uses spatial priors from a hierarchical structure to inform the mortality models. CODEm produces a large suite of models based on either cause fraction or mortality rate, uses mixed-effects linear and space-time Gaussian process regression models, and a covariate selection process. Each sub-model is evaluated using out-of-sample predictive validity. Thirty percent of the data are excluded from the initial model fits and 15% are used to evaluate component models and 15% used to build the ensembles. The sub-models are ranked using 15% of the data based on their out-of-sample predictive validity. The proportion weighting of the ensemble sub-models is evaluated using the remaining 15% of the hold-out data. This weighting scheme evaluates ensemble models that are built with ranked sub-models contributing proportionally more or fewer draws to the final ensemble. The final ensemble model is evaluated against other ensemble models using the same fit statistics (in-sample, out-of-sample root mean squared error and data coverage). Detailed information on this process can be found in Foreman et al 2012[2] and in the GBD 2017 Mortality and Causes of Death manuscript.[1]

Diarrhoea mortality is estimated for 23 age groups, 774 locations, both sexes, and every year from 1980-2017. We estimated diarrhoea mortality separately for males and females and for children under 5 years and older than 5 years due to expected underlying differences in the risk of mortality between these age groups. Data-rich and data-poor geographic locations were modelled separately and these models were then hybridised for a global model. This was to maintain proper uncertainty in the models where trusted data on causes of death exist. For a detailed description of the input data coverage, completeness, and reliability of the cause of death data in GBD 2017, please refer to the scoring system introduced in the GBD 2016 Mortality Collaborators manuscript.[3]

Like all models of mortality in GBD, diarrhoea mortality models are single-cause, requiring in effect that the sum of all mortality models must be equal to the all-cause mortality envelope. We correct diarrhoea mortality, and other causes of mortality, by re-scaling them according to the uncertainty around the cause-specific mortality rate. This process is called CoDCorrect and is essential to ensure internal consistency among causes of death.

In CODEm, the “level” of a covariate should reflect its position in a causal pathway where 1 is most proximally related to diarrhea mortality (causal) and 3 is distally

related or a proxy for diarrhea mortality. Details on CODEm covariate and sub-model selection can be found elsewhere[4] but the core idea is that submodels are built first using level 1 covariates and by adding covariates until the coefficient is either not significant or changes *direction.* The “direction” of a covariate is the sign of the coefficient. For covariates where the direction is positive, the covariate should be associated with diarrhea mortality such that greater exposure to the covariate is related to greater diarrhea mortality. The inverse is true for covariates where the direction is negative.

Table 2. The covariates used in diarrhoea mortality modeling. The *Level* represents the strength of the association between the covariate and diarrhoea mortality from 1 (proximally related) to 3 (distally related). The *Direction* indicates the positive or negative association between the covariate and diarrhoea mortality.

Level	Covariate	Direction
1	Diarrhea SEV	+
	Height for age < 2 SD	+
	Sanitation SEV	+
	Water SEV	+
	Weight for age < 2 SD	+
	Weight for height < 2 SD	+
	Oral rehydration solution treatment	-
	Safe sanitation	-
	Safe water	-
2	Vitamin A deficiency	+
	Zinc deficiency	+
	Healthcare access and quality index	-
	Rotavirus vaccine	-
	Zinc treatment for diarrhea	-
3	Breastfeeding SEV	+
	Handwashing	-
	LDI per capita	-
	Maternal education years	-
	Socio-demographic Index	-
	Population density < 150/km2	0
	Population density > 1000/km2	0

Comparison to other global health estimates

Our estimates of diarrhea mortality in children under 5 in 2016 (549,233, 95% UI 491,332-606,205) differ from those produced by the WHO Department of Evidence, Information and Research and the Maternal and Child Epidemiology Estimation (MCEE) group (477,293).[5] The main differences are in Nigeria. For Nigeria, the differences may arise from the input data used. GBD 2017 accepted a pair of verbal

autopsy studies representative at the subnational level for Nigeria because of the GBD principle of incorporating as many data as possible in the analysis.

Number of deaths due to diarrhea among children under-5 in the year 2016 is shown for the WHO-MCEE group as well as for GBD 2016 and GBD 2017

	WHO-MCEE	GBD 2017	GBD 2016
**Global**	**477,293**	**549,233**	**485,800**
Indonesia	8,551	11,190	8,700
Bangladesh	7,189	3,159	3,375
India	117,285	110,115	77,684
Pakistan	39,484	30,108	35,030
DR Congo	32,047	20,654	19,414
Ethiopia	15,535	31,326	16,361
Nigeria	76,980	106,294	106,357

This scatterplot shows the number of deaths estimated by GBD 2017 and the WHO/MCEE group in 2016.

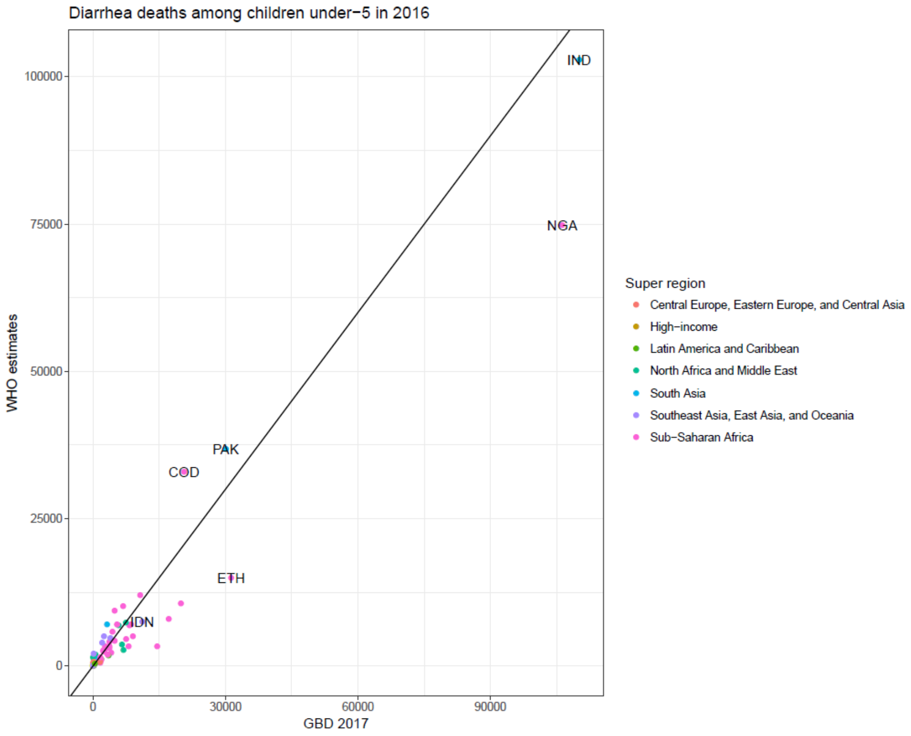

Description of risk factors used in this analysis

Risk factor description table. Definitions for risk factors and interventions used in this analysis. More information on each risk factor or intervention is provided in the Appendix, including input data and modeling strategy.

Type	Risk Factor	Definition	Modeled Values	Interpretation
Prevention	Handwashing	The proportion of the population that does not have access to a handwashing station with available soap and water.	The prevalence of the availability of a handwashing station with soap and water.	The provision of a handwashing station with soap reduces the exposure to fecal material.
Prevention	Low Rotavirus Vaccine Coverage	The proportion of children that are not vaccinated against rotavirus	The prevalence of children receiving a full course of rotavirus vaccine.	Not receiving the rotavirus vaccine puts children younger than 5 at an increased risk of dying from rotavirus diarrhea
Prevention	Unsafe sanitation	The proportion of the population that does not have access to a sanitation facility with a sewer connection.	The prevalence of unimproved sanitation use, the prevalence of improved sanitation facilities without a sewer connection or septic tank, the prevalence of toilet facilities with a sewer connection or septic tank.	Unsafe sanitation has three definitions based on the type of facilities in a population. Improved and unimproved facilities are based on the Joint Monitoring Programme definitions for sanitation.
Prevention	Unsafe water	The proportion of the population that does not use high quality piped water that has been boiled or filtered before drinking.	The prevalence of 1) unimproved water use without household water treatment (HWT), 2) unimproved water source with chlorine or solar purification, 3) unimproved water source with boiling or filtration, 4) Improved, non-piped water source without HWT, 5) Improved, non-piped water source with chlorine or solar purification, 6) Improved, non-piped water source with boiling or filtration, 7) Piped water source without HWT, 8) Piped water source with chlorine or solar purification, 9) Piped water source with boiling or filtration, 10) High quality piped water.	Unsafe water has several categorical definitions that have different relative risks for diarrhea. The definitions for improved and unimproved are from the Joint Monitoring Programme for water and sanitation. For example, unimproved and untreated water has the highest risk of diarrhea while improved, untreated water has a lower, but still existent, risk for diarrhea. The prevalence of each of these types of water are modeled for every population.
Prevention	Zinc deficiency	Consumption of less than 2.5 milligrams of dietary zinc per day	The prevalence of children who do not receive sufficient dietary zinc.	The low consumption of zinc puts children at elevated risks for mortality due to diarrhea.
Protection	Childhood stunting	Proportion of children younger than 5 years that are less than the WHO 2006 growth standard for height-for-age based on z-scores from that standard. The prevalence of mild (<-1 z score), moderate (-1 to -2 z scores), and severe	Prevalence of mild, moderate, and severe stunting.	Children who are short for their age, based on international growth standards, have a greater risk of dying from diarrhea than children who are not. Relative risks for diarrhea mortality by mild, moderate, and severe stunting are used in this analysis as

		(>-3 z-scores) were estimated for each population.		are modeled prevalence estimates for each of the stunting categories.
Protection	Childhood underweight	Proportion of children younger than 5 years that are less than the WHO 2006 growth standard for weight-for-age based on z-scores from that standard. The prevalence of mild (<-1 z score), moderate (-1 to -2 z scores), and severe (>-3 z-scores) were estimated for each population.	Prevalence of mild, moderate, and severe underweight.	Children who are low body weight for their age, based on international growth standards, have a greater risk of dying from diarrhea than children who are not. Relative risks for diarrhea mortality by mild, moderate, and severe underweight are used in this analysis as are modeled prevalence estimates for each of the underweight categories.
Protection	Childhood wasting	Proportion of children younger than 5 years that are less than the WHO 2006 growth standard for weight-for-height based on z-scores from that standard. The prevalence of mild (<-1 z score), moderate (-1 to -2 z scores), and severe (>-3 z-scores) were estimated for each population.	Prevalence of mild, moderate, and severe wasting.	Children who are low weight for their height, based on international growth standards, have a greater risk of dying from diarrhea than children who are not. Relative risks for diarrhea mortality by mild, moderate, and severe wasting are used in this analysis as are modeled prevalence estimates for each of the wasting categories.
Protection	Low birth weight and short gestation	A joint estimation of the prevalence of birth weight and gestational age below the minimum risk exposure level for birth weight and gestational age (<4000 grams and <38 weeks of gestation).	Jointly estimated prevalence of low weight and short gestational period, measured in a matrix of 500-gram birth weight and 2-week gestational periods.	The prevalence of low birth weight and short gestation period are modeled jointly. The prevalence for each category of birth weight, in 500 gram bins, and short gestation, in 2 week bins, represents the proportion of children in a population that were born prematurely.
Protection	Low Oral Rehydration Solution Coverage	The proportion of children with diarrhea that did not receive oral rehydration solution or recommended home fluids	The prevalence of children with diarrhea that were treated with oral rehydration solution or with recommended home fluids.	Not receiving oral rehydration solution or recommended home fluids puts children with diarrhea at an increased risk of death.
Protection	Suboptimal Breastfeeding	Suboptimal breastfeeding is either non-exclusive breastfeeding or discontinued breastfeeding. Non-exclusive breastfeeding is the proportion of children younger than 6 months that are not exclusively breastfed (predominant, partial, and none). Discontinued breastfeeding is the proportion of children 6 to 23 months who receive no breast milk.	Prevalence of predominant, partial, and no breastfeeding among children younger than 6 months, prevalence of children 6-23 months who receive no breastmilk.	Suboptimal breastfeeding is either non-exclusive breastfeeding for infants under 6 months or discontinued breastfeeding for children 6-23 months.

Protection	Vitamin A deficiency	Serum retinol less than 70 µmol/L	Prevalence of Vitamin A deficiency.	The prevalence of Vitamin A deficiency among children younger than 5 is a risk factor for diarrhea mortality.

Child Growth Failure

Flowchart

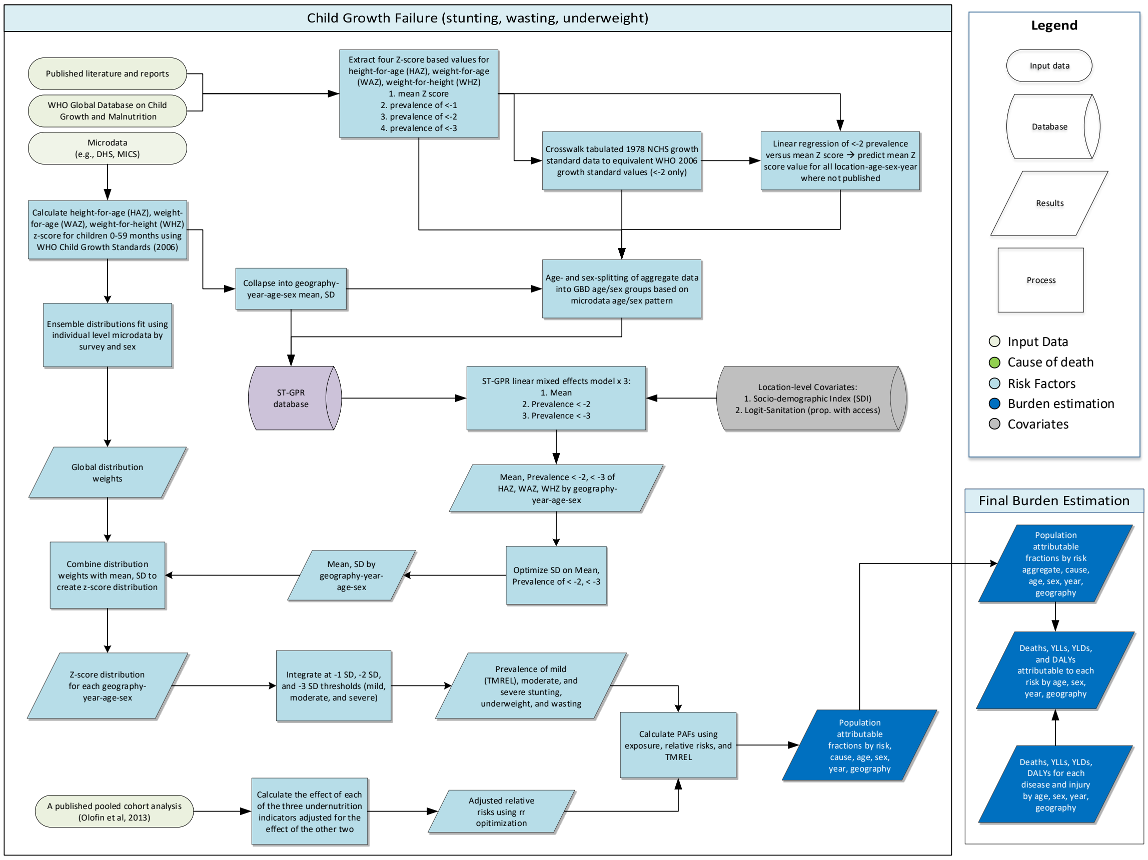

Input data & methodological summary

Exposure

Case definition

Child growth failure is estimated using three indicators, stunting, wasting, and underweight, all of which all of which are based on categorical definitions using the WHO 2006 growth standards for children 0-59 months. Definitions are based on Z scores from the growth standards, which were derived from an international reference population. Mild, moderate, and severe categorical prevalences were estimated for each of the three indicators.

Input data

There are three main inputs for the GBD child growth failure models: microdata from population surveys and tabulated data from reports, published literature, and the WHO Global Database on Child Growth and Malnutrition.[6] The primary data additions in GBD 2017 for child growth failure were from population surveys that include anthropometry. Population surveys include a variety of multi-country and country-specific survey series such as Multiple Indicator Cluster Surveys (MICS), Demographic

and Health Surveys (DHS), Living Standards Measurement Surveys (LSMS), and the China Health and Nutrition Survey (CHNS), as well as other one time country specific surveys such as the Indonesia Family Life Survey and the Brazil National Demographic and Health Survey of Children and Women. These microdata contain information about each individual child's age (from which age in weeks and age in months are calculated), as well as height and/or weight. From that information, a height-for-age z-score (HAZ), weight-for-age z-score (WAZ), and weight-for-height z-score (WHZ) are calculated using the WHO 2006 Child Growth Standards and the LMS method.[7]

All available data from the WHO Global Database on Child Growth and Malnutrition was extracted for GBD 2016 – much of which is from published studies. Exclusions included examination date prior to 1985, non-population representative studies, and those based on self-report. A systematic literature review was last completed in GBD 2010. We looked for four metrics from all sources with tabulated data: mean Z score, prevalence <-1 Z score (mild), prevalence <-2 Z score (moderate), and prevalence <-3 Z score (severe). All data for each metric was extracted for each of stunting (height-for-age Z score; HAZ), wasting (weight-for-height Z score; WHZ), and underweight (weight-for-age Z score; WAZ).

To maximise internal-consistency and comprehensiveness of the modelling dataset, we performed three data transformations. First, any data that were reported using the National Center for Health Statistics (NCHS) 1978 growth standards were crosswalked to corresponding values on the WHO 2006 Growth Standards curves based on a study that evaluated growth standard concordance.[8] Crosswalks from 1978 to 2006 growth standards were performed only on <-2 (i.e. moderate) prevalence data as that is where the concordance was most consistent. Second, for any study that lacked a measure of mean Z score for any of stunting, wasting, or underweight, we predicted a mean value for that study based on an ordinary-least squares regression of mean Z score versus <-2 prevalence for that metric from all sources where both were available. Third, any data that was presented as both sexes combined or for 0-59 months combined, we used the age and sex pattern from all data sources that included that detail to split into corresponding and age- and sex-specific data. All data was uploaded to a database and all inputs are catalogued in the Global Health Data Exchange (http://ghdx.healthdata.org). A representative dataset coverage map for moderate stunting is shown below.

Figure 1: Number of data points in moderate stunting (<-2 HAZ) in males, 1990 to 2017

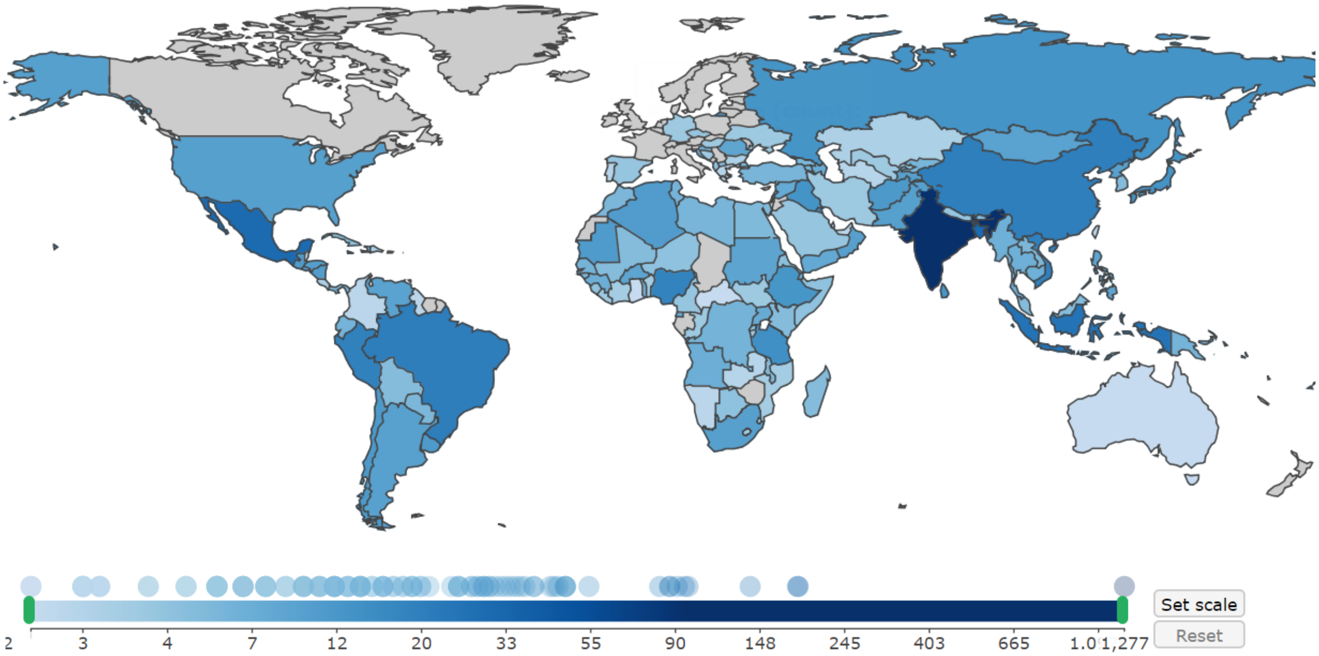

Modelling strategy

Exposure estimation

The following three-step modelling process was applied to each of stunting, wasting, and underweight.

First, all microdata was fit using an ensemble modelling process, a modelling framework developed for GBD 2016 that is described elsewhere in this appendix. A series of 12 individual distributions (normal, log normal, log logistic, exponential, gamma, mirror gamma, inverse gamma, gumbel, mirror gumbel, Weibull, inverse Weibull, and beta) were fit to the entire set of microdata (approximately 2.5 million individual z-scores) at the individual survey level. A weighting algorithm combined each distribution to find the optimal combination of these distributions for each survey, minimising the absolute prediction error across the entire distribution. Ensemble weights for each survey were then averaged across all surveys to produce a single set of global weights of the ensemble distributions. Weights were different for each sex, but invariant across geography, time, and age group. All component distributions that were used to derive weights were parameterised using "method of moments," meaning that each corresponding probability density function (PDF) could be described as a function of the mean and variance of the quantity of interest.

Second, models were developed for mean Z scores and prevalence of moderate and severe growth failure. Individual level microdata were collapsed to calculate three metrics: mean z-score, moderate prevalence, and severe prevalence. These data were combined with that derived from literature, GHDx review, and the WHO Global

Database on Child Growth and Malnutrition. Each of the three metrics was then modelled using spatiotemporal Gaussian process regression (ST-GPR), a common modelling framework used across GBD, generating estimates for each age-group, sex, year, and location. Location-level covariates used in all models included Socio-demographic Index (SDI) and logit-transformed proportion of households with improved sanitation.

Third, we combined estimates of mean, prevalence (moderate and severe) with ensemble weights in an optimisation framework in order to derive the variance that would best correspond to the predicted mean and prevalence. This variance was then paired with the mean and, using the method of moments equation for each of the component distributions of the ensemble, PDF of the distribution of Z-scores were calculated for each location, year, age-group, and sex. PDFs were integrated to determine the prevalence between -1 and -2 Z scores (mild), between -2 and -3 Z scores (moderate), and below -3 Z scores (severe). These were categorical exposures used for subsequent attributable risk analysis.

Ad-hoc data exclusions were limited. In some cases, we identified surveys with evidence of data entry issues (e.g. weights entered in a mixture of pounds and kilograms) that could not be corrected and these data were outliered. We initially ran all models with the complete dataset. Data plausibility inspection began with examination of time trends in stunting. If a given datum was judged to have led to a change in the prevalence of moderate stunting in 1-4 year olds of 50% or greater in 5 years or fewer, and was inconsistent with data prior to and after that year (a change considered implausible), we outliered the offending datum and reran the model. We then further visually-inspected the results of moderate stunting, wasting, and underweight in parallel to look for location-age-sex-years where the results were not internally-consistent (e.g. stunting and wasting decreasing, underweight rapidly increasing). This inspection revealed very few inconsistent data.

Improvements from GBD 2015 to GBD 2016/ 2017

In GBD 2017, the primary changes from GBD 2016 were the 1) addition of a significant volume of new survey data, 2) crosswalking instead of down-weighting data based on NCHS 1978 growth standard, 3) utilisation of updated versions of location-level covariates, and 4) utilisation of an updated version of the ST-GPR modelling framework that empirically derives many of the modelling parameters.

There are several important differences from the GBD 2015 analysis. First, our systematic data searching efforts led to an approximately 30% increase in the number of data sources since GBD 2015, including a significant increase in data sources for Oceania, Latin America, and South Asia. Most notable was the increase in data for India through our collaboration with the India Council for Medical Research (ICMR) and

Public Health Foundation of India (PHFI). Second, while GBD 2015 also used ST-GPR to model growth failure, models were completed for a single 0-5 age group, followed by application of a pooled uniform age-sex split which resulted in the implicit assumption that the age pattern of growth failure is invariant over time and geography. GBD 2016 estimates, owing to smaller sample sizes in younger age groups, do have wider uncertainty in those age groups. Third, GBD 2015, like all analyses of growth failure before it, assumed that high-income countries had zero prevalence of child growth failure. We suspended this assumption in GBD 2016 as it is not accurate and instead made explicit estimates of growth failure in all locations. Fourth, GBD 2015 did not use an ensemble approach or estimate the entire distribution of Z scores. Fifth, we changed the name of this risk factor category changed from childhood undernutrition to child growth failure to more explicitly identify the specific aspects of childhood undernutrition that are covered by the three component indicators.

Theoretical minimum-risk exposure level

Theoretical minimum risk exposure level (TMREL) for underweight, stunting, and wasting was assigned to be greater than or equal to -1 SD of the WHO 2006 standard weight-for-age, height-for-age, and weight-for-height curves respectively. This has not changed since GBD 2010.

Relative risks

The final list of outcomes paired with child growth failure risks included lower respiratory infections (LRI), diarrhea, measles, and protein energy malnutrition (PEM) as shown in Table 1. These were derived from a pooled cohort analysis by Olofin and colleagues.[5]

There is a high degree of correlation between stunting, wasting, and underweight. Failing to account for their covariance and assuming independence would overestimate the total burden significantly. This is the main reason that GBD 2010 only included childhood underweight. In GBD 2013, a method was developed to adjust observed RRs of Olofin and colleagues by simulating the joint distribution of the three indicators using the distribution of each indicator and covariance between indicators in the countries included in the meta-analysis (extracted from Demographic and Health Survey (DHS) micro-data).[9] Based on the analysis done by McDonald and colleagues, we assumed there is an interaction between the three indicators, and extracted the interaction terms from the corresponding analysis. We calculated the adjusted RRs by minimising the error between observed crude RRs (from meta-analysis) and expected crude RRs derived from adjusted RRs.

Of historical note, URI and otitis media were included as outcomes in the GBD 2013 risk analysis, based on the "analogy" causal criterion, assuming there is similar pathway as LRI outcome. However, closer review for GBD 2015 did not find sufficient evidence to support their inclusion and they were excluded, a decision that was carried forward into GBD 2016. We also attributed 100% of PEM to childhood wasting and underweight

but not stunting. To build on the existing literature base for GBD on risk-outcome pairs, a literature search was conducted for GBD 2017 searching for case-control studies published after January 1st, 1985; this search did not return any sources that were appropriate for this work.

Table 1: Adjusted RRs for each risk-outcome pair for child growth failure

Outcome	**Stunting**	**Wasting**	**Underweight**
Diarrhea	<-1: 1.111 (1.023-1.273)<br><-2: 1.222 (1.067-1.5)<br><-3: 1.851 (1.28-2.699)	<-1: 6.601 (2.158-11.243)<br><-2: 23.261 (9.02-35.845)<br><-3: 105.759 (42.198-157.813)	<-1: 1.088 (1.046-1.134)<br><-2: 1.23 (1.163-1.314)<br><-3: 2.332 (2.076-2.802)
Lower respiratory infections (LRI)	<-1: 1.125 (0.998-1.655)<br><-2: 1.318 (1.014-2.165)<br><-3: 2.355 (1.15-5.114)	<-1: 5.941 (1.972-11.992)<br><-2: 20.455 (70.84-37.929)<br><-3: 47.67 (15.923-94.874)	<-1: 1.145 (1.044-1.364)<br><-2: 1.365 (1.215-1.755)<br><-3: 2.593 (1.908-4.39)
Measles	<-1: 1.103 (0.861-1.719)<br><-2: 1.54 (1.029-3.222)<br><-3: 2.487 (1.129-6.528)	<-1: 1.833 (0.569-8.965)<br><-2: 8.477 (1.33-42.777)<br><-3: 37.936 (5.088-199.126)	<-1: 0.995 (0.5-1.726)<br><-2: 2.458 (1.26-5.118)<br><-3: 5.668 (1.767-12.414)
Protein-energy malnutrition	0% PAF	100% PAF	100% PAF

Unsafe Hygiene

Flowchart

Unsafe Handwashing

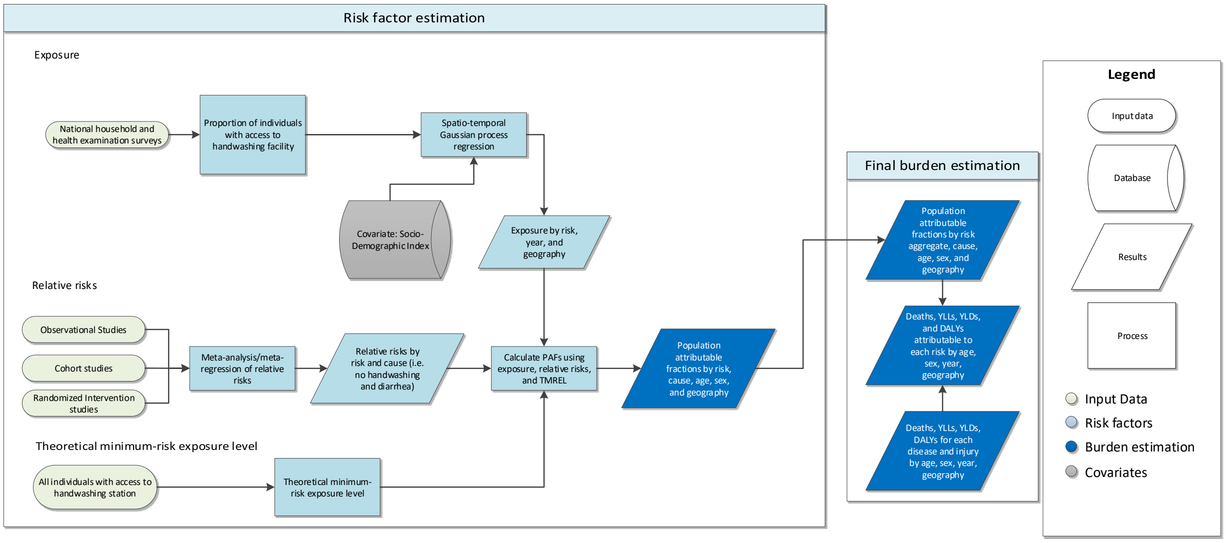

Input data & methodological summary

Exposure

Case definition

Unsafe hygiene is defined as lack of access to a handwashing station with available soap and water. We estimated the burden of unsafe hygiene in both developed and developing settings.

Input data

Since water and soap availability data are very limited, only country-specific Demographic Health Surveys (DHS) and Malaria Indicator Survey Series (MICS) conducted after 2006 were included as input data.

Modelling strategy

By year and location, proportion of households with handwashing facility is modelled using a 3-step modelling scheme of mixed effect linear regression followed by spatio-temporal Gaussian process regression (ST-GPR), which outputs full time series estimates for each GBD 2017 location. Socio-demographic index (SDI), a composite index that include income per capita, education, and fertility, was set as a fixed effect in the linear regression since it proved to have significant coefficient. Random effects were set at GBD 2017 region and super-region levels to fit the model but were not used in the predictions.

The process of vetting and validating models was accomplished primarily through an examination of ST-GPR scatter plots by GBD 2016 location from 1990-2016. Any data points lacking face validity were re-inspected for error at the level of extraction and survey implementation, and subsequently excluded from analysis if deemed appropriate. In addition to SDI, a number of different potential fixed effects were considered, including lag-distributed income and urbanicity. However, SDI proved to be the strongest predictor.

A considerable limitation for when estimating handwashing practices for over 190 independent locations around the world was data sparseness. Even when data were published on handwashing prevalence, the definition was often altered from the GBD 2017 standard definition or it may only have pertained to certain populations (such as hospital patients) and lacked representativeness at the geographic scale we required. The incorporation of questions about soap and water availability in DHS and MICS added much-needed information but there remains a large data gap to be filled if we are to become more certain in handwashing access estimates.

Theoretical minimum-risk exposure level

The theoretical minimum-risk exposure level for unsafe hygiene is defined as all individuals with access to handwashing facility after any contact with excreta, including children's excreta.

Relative risks

A meta-analysis by Cairncross et al.[11] provide relative risk values describing the relationship between lack of facility access and diarrheal diseases (Relative risk for diarrhea was 1.91 among populations without access to handwashing facility with soap, 95% CI 1.32 to 2.67).

Unsafe Sanitation

Flowchart

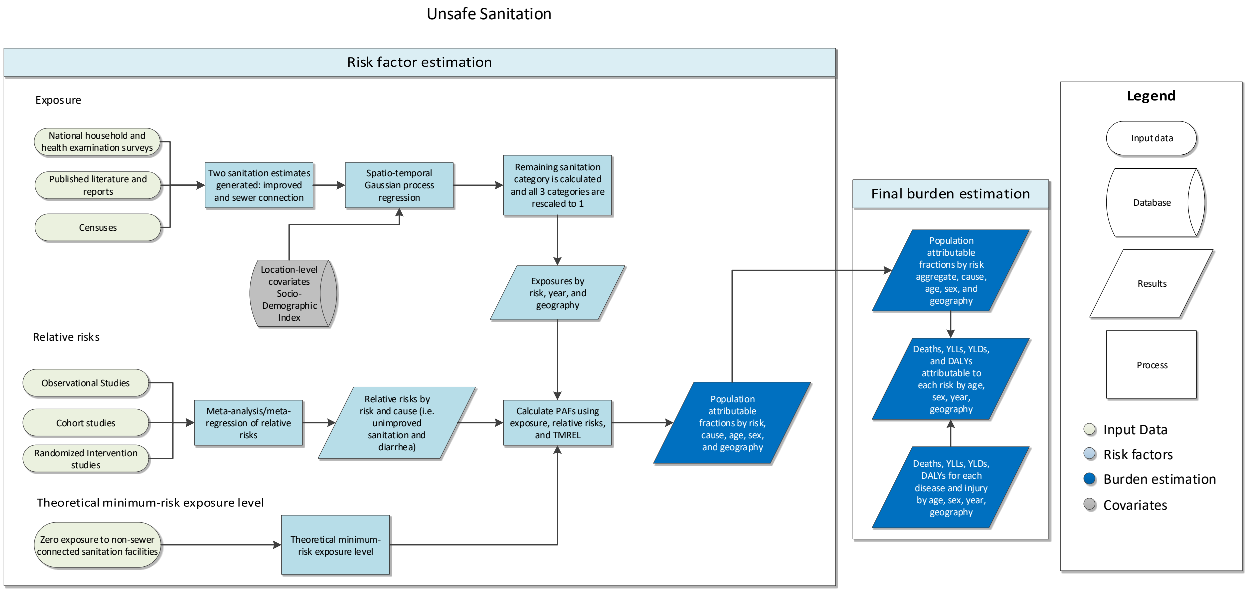

Input data & methodological summary

Exposure

Case definition

Exposure to unsafe sanitation is defined based on the primary toilet type used by households. Improved facilities are defined as such based on JMP designation (WHO).[12] Sewer connection toilets included flush toilets or any toilet with connection to the sewer or septic tank.

Input data

The search for usable household surveys and censuses was conducted using the Global Health Data Exchange (GHDx) database. For each survey, household sample weights were multiplied by the number of household members to produce a weighting scheme that estimates proportion of individuals, not proportion of households, exposed to a given indicator. Surveys and censuses were then tabulated to two sanitation categories, sewer connection and improved sanitation, for each location. Data in tabulated form was lower priority to add to models and was only updated when time permitted.

Modeling

A change made for GBD 2017 was to model sanitation categories in an ordinal framework instead of independent models. Two distinct indicators were estimated: the prevalence of individuals using sewer connection or septic tank facilities and the proportion of individuals with improved sanitation within the population not connected to sewer or septic tank. This ordinal framework allows us to estimate the category with the most data (sewer connection/septic tank prevalence) and leverage that estimate to anchor the estimates for improved and unimproved sanitation categories. The results of the improved proportion model are multiplied by the sewer connection/septic tank prevalence to calculate improved sanitation prevalence. The sum of improved and sewer connection/septic tank prevalence are subtracted from 1 to yield unimproved sanitation prevalence.

The two indicators were modeled using a 3-step modeling scheme of mixed effect linear regression followed by spatio-temporal Gaussian process regression (ST-GPR), which produced full time series estimates for each GBD 2017 location. Socio-demographic index (SDI), a composite metric combining education per capita, income per capita, and fertility, was set as a fixed effect in the linear regression since it proved to be a significant predictor. Random effects were set at GBD 2017 region and super-region levels to fit the models but were not used in the predictions.

The process of vetting and validating models was accomplished primarily through an examination of ST-GPR scatter plots by GBD 2017 location from 1990-2017. Any unfitting data points were re-inspected for error at the level of extraction and survey implementation, and subsequently excluded from analysis if deemed appropriate. In addition to SDI, a number of different potential fixed effects were considered, including lag-distributed income and urbanicity, but SDI proved to be the strongest predictor of unsafe sanitation in terms of magnitude of the coefficient. Uncertainty in the estimates was initially constructed based on standard deviation around each survey mean, then propagated through ST-GPR modeling by incorporating the variance of each data point in the Gaussian process regression step. A data point with high variance, for example, would contribute relatively less influence to the model than a data point with lower variance.

Once models are vetted, full time series outputs from ST-GPR modeling are then converted from proportion to prevalence by year and geography and then rescaled to form three mutually exclusive categories that sum up to 1. The table below provides the final result of this rescaling.

Category	*Definition*	*Relative risk for diarrhea given exposure*
Unimproved sanitation	Proportion of individuals that use	3.242<br>(2.528 to 4.067)

	unimproved sanitation facilities.	
Improved sanitation	Proportion of individuals with access to improved sanitation facilities, excluding sewer connection or septic tank.	2.595<br>(2.044 to 3.285)
Sanitation facilities with sewer connection or septic tank	Proportion of individuals with access to toilet facilities with sewer connection or septic tank.	1.0<br>(1.0 to 1.0)

Theoretical minimum-risk exposure level

The theoretical minimum-risk exposure level for unsafe sanitation was defined as all individuals have access to a sanitation facility with sewer connection.

Relative risks

For GBD 2017, unsafe sanitation was only paired with one outcome, diarrheal diseases. A meta-analysis by Wolf et al. 2014[13] provides the bulk of the relative risk evidence for the relationship between unsafe sanitation and diarrheal diseases. This meta-analysis was updated through a literature review that searched for related intervention studies post-2014 conducted in PubMed. Search terms used were identical to those provided by Wolf et al. 2014. Please refer to appendix tables for more information on relative risk values and citations.

Unsafe Water

Flowchart

Unsafe Drinking Water

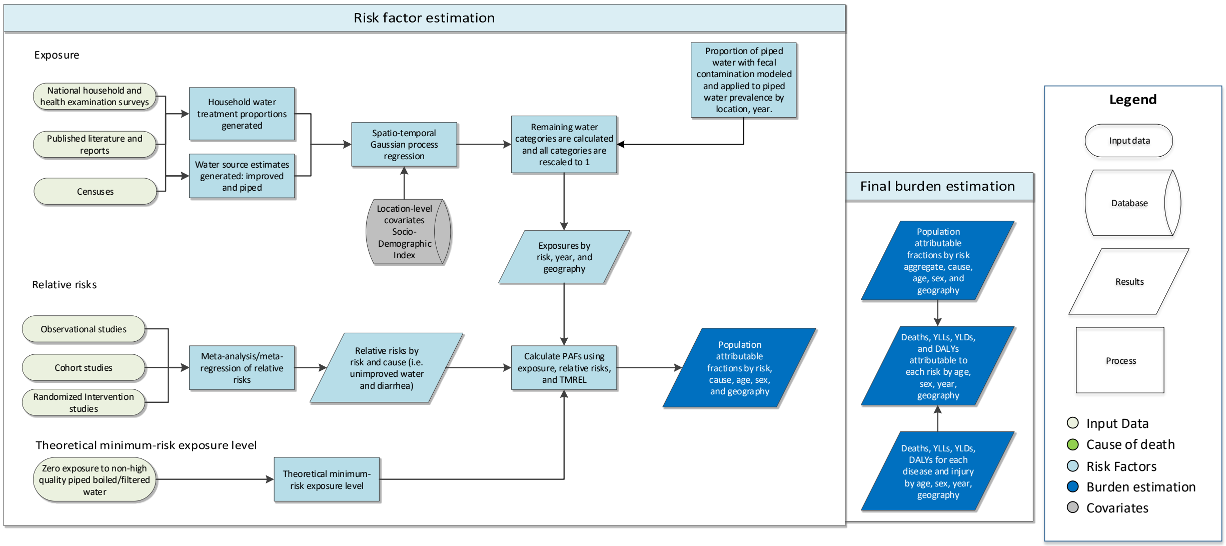

Input data & methodological summary

Exposure

Case definition

For GBD 2017, exposure to unsafe water was defined based on reported primary water source used by the household and use of household water treatment (HWT) to improve the quality of drinking water before consumption. Water sources were defined as "improved" based on the JMP designation,[12] which includes piped water as improved water, and households with access to piped water connection to the house, yard, or plot were defined as having access to piped water supply. Solar treatment, chlorine treatment, boiling, or the use of filters were all established as effective point-of-use household water treatments based on effect sizes calculated from network meta-analysis.

Input data

The search for usable household surveys and censuses was conducted using the Global Health Data Exchange (GHDx) database. HWT input data is primarily limited to two large survey series (DHS and MICS) due to data availability. Water source data includes censuses and nationally representative surveys such as DHS, MICS, AIS, and WHS. For each survey, household sample weights were multiplied by the number of household members to produce a weighting scheme that estimates proportion of individuals, not proportion of households, exposed to a given indicator. Surveys and censuses were then tabulated to the two water source and two water treatment categories of interest for each location.

Modelling

Water source data is modelled using an ordinal framework, with two distinct models: prevalence of piped water and proportion of improved water (excluding piped) within the non-piped population. Both models produce results for each unique location, year combination. This ordinal framework allows us to estimate the category with the most data (piped water prevalence) and leverage that estimate to anchor the estimates for improved and unimproved water categories. The results of the improved proportion model are multiplied by the piped water prevalence to calculate improved water prevalence. The sum of improved and piped water prevalence are subtracted from 1 to yield unimproved water prevalence.

HWT categories are estimated in a similar ordinal framework, by modelling prevalence of individuals using no water treatment methods and proportions of households that boil/filter water within the population of households that engage in treatment methods. The prevalence of individuals that boil/filter drinking water is calculated by multiplying the proportion that boil/filter modelled previously times prevalence of any water treatment (estimated by subtracting prevalence of no treatment from 1). The prevalence of individuals that treat their water using solar/chlorine methods was estimated by subtracting the sum of prevalence of no treatment estimates and prevalence of filter/boil treatment from 1. By year and location, each of the above categories are modelled using a 3-step modelling scheme of mixed effect linear regression followed by spatio-temporal Gaussian process regression (ST-GPR), which produces full time series estimates for each GBD 2017 location. Socio-demographic index (SDI), a composite metric combining education per capita, income per capita, and fertility, was set as a fixed effect in the linear regression since it proved to be a significant predictor. Random effects were set at GBD 2017 region and super-region levels to fit the models but were not used in the predictions.

The process of vetting and validating models was accomplished primarily through an examination of ST-GPR scatter plots by GBD 2017 location from 1990-2017. Any unfitting data points were re-inspected for error at the level of extraction and survey implementation, and subsequently excluded from analysis if deemed appropriate. In addition to SDI, a number of different potential fixed effects were considered, including lag-distributed income and urbanicity, but SDI proved to be the strongest predictor of the unsafe water categories. Uncertainty in the estimates was initially formed based on standard deviation by survey, then propagated through ST-GPR modelling by means of confidence intervals around each data point that reflect the point-estimate specific variance.

Once models are vetted, full time series outputs from ST-GPR modelling are then converted from proportion to prevalence by year and geography and then rescaled to form 9 mutually exclusive categories that sum up to 1. The table below provides the final result of this rescaling.

Category	Definition	Relative risk of diarrhea given exposure
Unimproved, no HWT	Proportion of individuals that primarily use unimproved source, and *do not* use any HWT to purify their drinking water.	11.501 (2.761 to 31.282)
Unimproved, chlorine/solar	Proportion of individuals that primarily use unimproved source, and solar or chlorine treatment to purify their drinking water.	7.914 (1.971 to 21.188)
Unimproved, boil/filter	Proportion of individuals that primarily use unimproved source, and boil or filter to purify their drinking water.	4.789 (1.204 to 12.752)
Improved water except piped, no HWT	Proportion of individuals that primarily use improved sources other than piped water supply, and *do not* use any HWT to purify their drinking water.	9.428 (2.782 to 23.028)
Improved water except piped, chlorine/solar	Proportion of individuals that primarily use improved sources other than piped water supply, and use solar or chlorine treatment to purify their drinking water.	6.488 (1.997 to 15.677)
Improved water except piped, boil/filter	Proportion of individuals that primarily use improved sources other than piped water supply, and boil/filter their drinking water.	3.926 (1.22 to 9.401)
Basic piped water, no HWT	Proportion of individuals that primarily use basic piped water supply, and *do not* use any HWT to purify their drinking water	8.431 (2.533 to 20.446)
Basic piped water, chlorine/solar	Proportion of individuals that primarily use basic piped water supply, and *use* solar or chlorine water treatment to purify their drinking water.	5.802 (1.807 to 13.843)
Basic piped water, boil/filter	Proportion of individuals that primarily use basic piped water supply, and boil or filter to purify their drinking water	3.511 (1.107 to 8.331)
High-quality (HQ) piped water, boil/filter	Proportion of individuals that primarily use high-quality piped water supply, and boil or filter to purify their drinking water	1 (1 to 1)

We modelled the microbiological quality of piped water sources primarily using data from a review by Bain et al.[14] that measured proportion of piped water sources contaminated with fecal indicators. We use the value generated from this model to split the prevalence of piped water into basic piped water and high quality piped water by location, year, age, and sex.

A substantial limitation in our analysis is the paucity of data on piped water quality. The inclusion of more location-specific data on water treatment utilisation at the household level can greatly improve our estimates in future iterations.

Theoretical minimum-risk exposure level

The theoretical minimum-risk exposure level for unsafe water is defined as all households have access to high quality piped water that has been boiled or filtered before drinking.

Relative risks

For GBD 2017, unsafe water was paired with one outcome-diarrheal diseases-given evidence provided by relative risk studies. A meta-analysis by Wolf et al.[13] provided the bulk of the relative risk evidence for the relationship between unsafe water and diarrheal diseases. This meta-analysis was updated through a literature review that searched for related intervention studies post-2014 conducted in PubMed. Search terms used were identical to those provided by Wolf et al.[13] Relative risk values for water-source interventions and point-of-use treatment interventions were calculated using network meta-analysis approach so as to include studies that differ in control groups within the same analysis. This analysis produced distinct relative risks for each water source and water treatment category. The combined effect of a source intervention and point-of-use intervention was assumed to be multiplicative in order to match GBD 2017 exposure definitions. Please refer to appendix table for more information on relative risk values and citations.

Oral rehydration solution

Treatment for Childhood Illness Flowchart

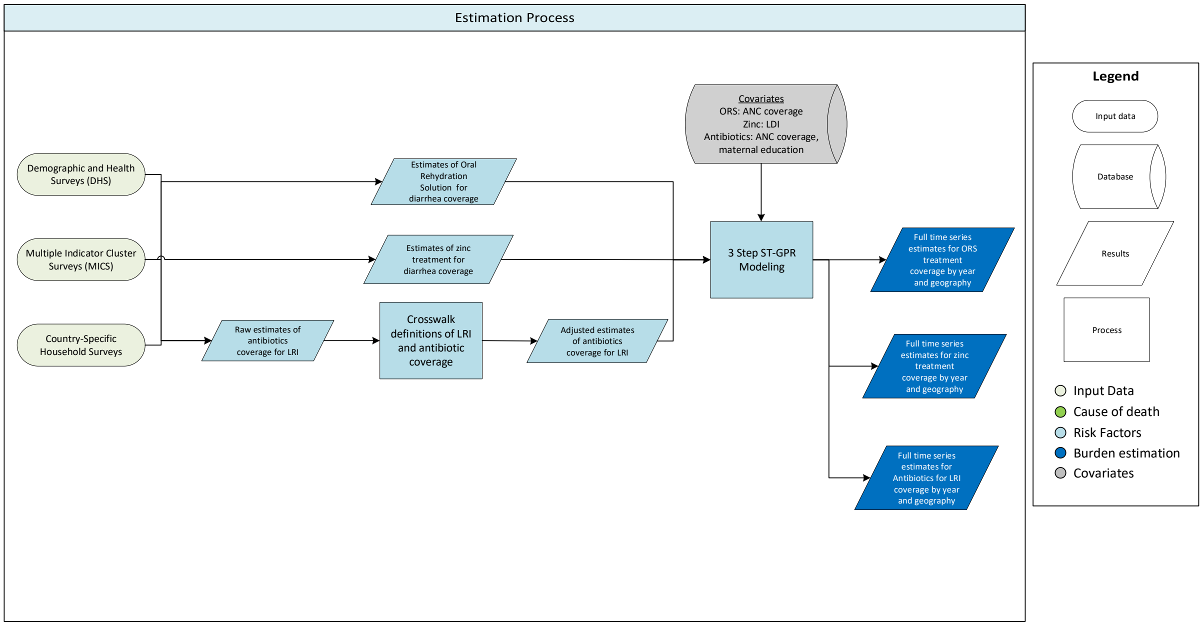

Oral rehydration solution (ORS) for diarrhea: Oral rehydration solution (ORS) for diarrhea is defined as the proportion of children ages 0-4 years who had diarrhea in the past two weeks and received ORS.

Input Data

Data Source Identification

Across all childhood illness care indicators, we use individual-level microdata from population health surveys. Individual-level data comes primarily from major multi-country surveys that survey women on recent illnesses of children in their household as well as any care received, including Demographic and Health Surveys (DHS),[15] Multiple Indicator Cluster Surveys (MICS),[16] Reproductive Health Surveys (RHS),[17] and Living Standards Measurement Study (LSMS) surveys.[18] We supplement these with microdata from individual country-specific surveys.

The last data update was conducted for GBD 2017, resulting in the following data and source counts:

Indicator	No. of Observations	No. of Studies	Year Range
ORS for diarrhea	1051	435	1986-2016

ORS for Diarrhea input data

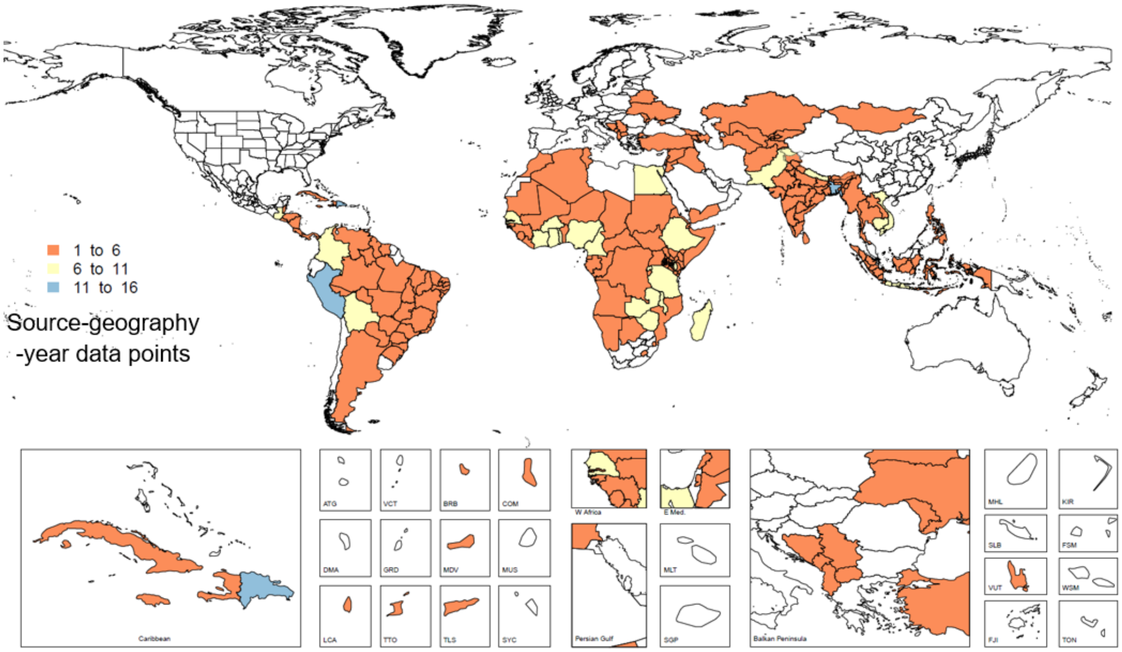

Indicator and Source Metadata

Many household surveys collect information on maternal and child health (MCH) indicators for children under 5 and/or mothers who gave birth within five years prior to the time of survey. We include surveys that are geographically representative and rely on maternal-reported care received among children with illness in the prior two weeks, including treatment received for diarrhea. We exclude all data sources that are not nationally representative (but include surveys that are only subnationally representative if they are from one of the subnational units estimated in GBD) as well as surveys with high levels of missingness.

ORS coverage data were extracted as care received for diarrhea among children who are reported to have had diarrhea in the two weeks prior to survey.

The table below includes the variables used in calculating numerator and denominator for each of the indicators:

Indicator	Numerator	Denominator	Definition
ORS for diarrhea	# of children with diarrhea in past 2 weeks who received ORS treatment	# of total children with diarrhea in the past 2 weeks	Proportion of all children 0-4 years who had diarrhea in the past 2 weeks who received ORS treatment

Modeling Strategy

ST-GPR

We estimate ORS coverage by location and year using Spatiotemporal Gaussian Process Regression (ST-GPR), a three-stage modeling tool used extensively in GBD. The first step is a mixed effects linear regression. We use the following location-level covariates along with standard nested random effects for super-regions, regions, and countries:

Indicator	Covariates
ORS for Diarrhea	ANC1+ coverage

The second step of ST-GPR takes the residuals of the linear regression and smooths them based on proximity in space and time. The third step is Gaussian process smoothing that takes into account the uncertainty in the data points and estimates. Random draws of 1,000 samples were obtained from the final distribution for every country and year. Ninety-five percent uncertainty intervals were calculated by taking the ordinal 25th and 975th draws from the sample distribution.

To produce coverage estimates of all child illness treatment indicators, we model in ST-GPR the proportion of children who had diarrhea in the past 2 weeks who received ORS treatment.

Relative Risks

Indicator	Outcome	Effect Size	Source	N Studies
ORS for diarrhea	Diarrhea mortality	0.31 (0.20-0.49)	Munos et al. 2010[19]	3 Quasi-Experimental

Rotavirus vaccine coverage

Case definition

This modeling strategy pertains to measures of vaccine coverage, the proportion of the target population covered by rotavirus vaccine (two or three doses).

Input data

The present study used data from household-level surveys as well as administrative reports of immunization coverage. Survey data which provided person-level information on immunization were identified and extracted. Major multi-country survey programs included in the analysis include the Demographic and Health Surveys (DHS),[20] Multiple Indicator Cluster Surveys (MICS),[21] Reproductive Health Surveys (RHS),[22] Living Standards Measurement Study (LSMS) surveys,[23] and World Health Surveys (WHS).[24] We also conducted a comprehensive search of the Global Health Data Exchange (GHDx),[25] as well as targeted internet searches and review of Ministry of Health websites, to identify national surveys and other multi-country survey programs.

Administrative estimates of immunization coverage were obtained from the Joint Reporting Form (JRF),[26] through which the World Health Organization (WHO) and UNICEF collate annual estimates of immunization coverage reported by UN member states. These immunization coverage estimates are separate from those synthesized by WHO, and are calculated by dividing the number of doses of a given vaccine delivered to the target population (i.e., children aged 12 to 23) by the number of individuals in that target population.

We excluded all data sources that were not nationally representative or had high levels of missingness. We applied survey weights based on survey sampling frames whenever they were available to generate weighted national estimates of vaccination coverage accompanied by estimates of standard error (SE). Estimates of SE, as well as sample sizes, were used to calculate uncertainty, as described below. Any point estimates with sample sizes less than 50 were reviewed to ensure that were not substantive outliers and would otherwise have an undue influence on our analysis.

Modeling strategy

Data processing

Age splitting

Most household surveys collect information on maternal and child health (MCH) indicators for children under 5 and/or mothers who gave birth within five years prior to the time of survey. To maximize data use for our model, we included immunization data for children aged 12 to 59 at the time of survey. Children younger than 12 months of age were excluded to minimize the influence of potentially censored observations.

For each vaccine, coverage estimates were assigned to birth-cohort years based on a child's age prior to the time of survey: we used responses recorded for children aged 12 to 23 months for immunization coverage for one year prior to the time of survey, children aged 24 to 35 months for coverage two years prior to the time of survey, and so forth.

Age-specific estimates are easily computed from individual-level microdata, but many published reports and survey summaries present data in broader age aggregates (e.g. coverage for children aged 12 to 35 months). To standardize these age groups, we applied an age-splitting model used in the GBD study[27] and other analyses that generated smoking and obesity prevalence by age group.[28,29]

Using surveys with microdata as the reference, we used the following model to generate standardized age group-specific estimates of immunization coverage:

$$\tilde{P}_{a,c,t,k} = P_{a,c,t,k}^{a+x} \frac{P_{a,c,t,j}}{P_{a,c,t,j}^{a+x}}$$

where $\tilde{P}_{a,c,k}$ is the adjusted estimate of coverage for target age group a in country c and year t of survey k; and $P_{a,c,k}^{a+x}$ is coverage reported from survey k, for country c in year t for the age group spanning age a to age $(a + x)$. The ratio of coverage between the target age group and broader age group from a survey j with microdata from the same country-year was used to split data from survey k. Surveys to be split were ideally matched with DHS or MICS surveys. If microdata were not available for the same year, ratios within five years of the survey that required age-splitting were applied.

Administrative bias adjustment

Intervention coverage estimates based on administrative sources can be biased. Such biases may arise for a number of reasons, including discrepancies in the accurate reporting of services or interventions provided (e.g. number of vaccine doses administered) and target population (e.g. number of children in need of vaccines), as well as capturing these data in a timely manner from both public and private-sector facilities and health care providers. We implemented a vaccine-specific bias adjustment process to account for bias in administrative reports of immunization coverage in the JRF for DTP3 vaccine coverage. Given that the magnitude, direction, and cause of such biases are heterogeneous across space, time, and antigen,[30,31] a vaccine-specific, time-varying, all-location bias correction factor was used.

For immunization coverage, we view individual-level data collected through population health surveys as the most accurate and least biased source of information of vaccination coverage, particularly for geographies with incomplete health information systems. We thus compute administrative bias as the ratio between estimates of coverage from surveys (where available) and matched administrative coverage. We model this bias in a

spatiotemporal Gaussian process regression (ST-GPR) framework, described further in the subsequent section of this appendix, using the Socio-demographic index (SDI) as a predictor. This method allows us to estimate antigen-specific administrative bias factors for all geographies and years since 1980, even in places without survey data, by borrowing strength in data across space and time. The GPR framework properly estimates prediction errors in the data synthesis procedure by for uncertainty in bias ratios when generating fitted values. In this framework, more weight is given to survey data with less uncertainty.

Antigen-specific modeled estimates of administrative bias are then used to adjust administrative coverage data for over- or under-reporting to reflect observed survey coverage. Adjusted administrative data are used as inputs into the trend estimation process.

Although we only directly perform an administrative bias adjustment on data for DTP3, our ratio-based modeling strategy for the other antigens (described further below) leverages the bias-corrected administrative data by multiplying the modeled ratio by the coverage of the denominator in the final step.

Trend estimation

We used a spatiotemporal Gaussian process regression (ST-GPR) to synthesize point estimates from multiple data sources and derive a complete time series for each vaccine. This method has been used extensively GBD and related studies, and accounts for uncertainty pertaining to each point estimate while borrowing strength across geographic space and time.[10, 11,15,16] Briefly, we assumed the Gaussian process was defined by a mean function $m(\bullet)$ and covariance function $Cov(\bullet)$.

We estimated the mean function using a two-step approach. Specifically, $m_c(t)$ can be expressed as:

$$m_c(t) = X\beta + h(r_{c,t})$$

where $X\beta$ is a linear model and $h(r_{c,t})$ is a smoothing function for the residuals; and $r_{c,t}$ is derived from the linear model. The following linear model was used to model DPT3 coverage:

$$logit(P_{c,t}) = \beta_0 + \beta_1 \mathrm{HAQ_{c,t}} + \alpha_c + \gamma_{R[c]} + \omega_{\mathrm{SR[c]}} + \varepsilon_{c,t}$$

where $P_{c,t}$ is vaccination coverage for country c year t; $HAQ_{c,t}$ is value of the Healthcare Access and Quality Index[33] for country c and year t; α_c, $\gamma_{R[c]}$, and $\omega_{\mathrm{SR[c]}}$ are country, region, and super-region random intercepts, respectively. These estimates were then modeled through ST-GPR.

Given their recent introduction, there are limited data on coverage Hib3, PCV3, and rotavirus vaccines. To leverage the relatively data-rich DTP3 estimates, we modeled the scale-up of each vaccine over time by modeling their ratio with the more data-rich DTP3 vaccine coverage. We first calculated the ratio of vaccine coverage data (including survey data and uncorrected administrative data) with DTP3 by survey-year. We then modeled the full time series of the ratios using ST-GPR. The following linear model was used as the mean function for the Hib, PCV, and Rota ratios with DPT3:

$$logit(P_{c,i}) = \beta_0 + \beta_1 \mathrm{HAQ}_{\mathrm{c,i}} + \alpha_c + \gamma_{R[c]} + \omega_{\mathrm{SR[c]}} + \varepsilon_{c,i}$$

where $P_{c,i}$ is the coverage ratio for country c time since introduction i; $HAQ_{c,i}$ is value of the Healthcare Access and Quality Index[33] for country c and time since introduction i; α_c, $\gamma_{R[c]}$, and $\omega_{\mathrm{SR[c]}}$ are country, region, and super-region random intercepts, respectively. We ultimately obtained estimates of coverage by multiplying the modeled ratio by the final estimated DTP3 coverage by location and year.

Random draws of 1,000 samples were obtained from the distributions above for every country for a given vaccine. Ninety-five percent uncertainty intervals were calculated by taking the ordinal 25th and 975th draws from the sample distribution.

To assess the accuracy of our modeled estimates, we performed cross-validation analyses using a knockout structure as previously described[34]. ST-GPR hyperparameters were selected on models that minimized the overall root-mean squared error (RMSE) of the model across a set of 10 knockouts.

Routine introduction

National vaccine schedules and vaccine introduction dates were used as reported from WHO[35] or from the country's Ministry of Health website where otherwise unavailable.

Vaccine efficacy

The vaccine efficacy for rotavirus vaccine was a single value for all locations and based on a meta-analysis performed using data from a review by Lamberti et al. (76.5%, 95% UI 70.7 to 82.2%).[36]

Zinc Deficiency Capstone Appendix

Exposure

Case definition

Exposure to zinc deficiency is defined as consumption of less than 2.5 milligrams of zinc per day among children between the ages of 1 and 4 years old.

Input data

We used dietary data from nationally and sub-nationally representative nutrition surveys and United Nations FAO Supply and Utilization Accounts to estimate the mean intake of zinc at the population level.

Modelling strategy

For GBD 2016, we first used a spatio-temporal Gaussian process regression (ST-GPR) framework to estimate the mean intake of zinc by age, sex, country, and year. To assist with estimation for locations and years without data, we used the lag-distributed income of that location-year as a covariate. We considered data from 24-hour diet recall as the gold standard, and adjusted data from other sources to the gold standard method. Using the method described in the dietary risks section, we characterised the distribution of zinc intake for children between ages of 1 and 4 years old and estimate the proportion of the children with intake of less than 2.5 milligrams of zinc per day.

Relative risk

Relative risks used for zinc deficiency is based on the results of randomised trials that measured the effect of zinc supplementation. The relative risk for diarrhea morbidity given zinc deficiency was 1.9 (95% CI 1.5 to 2.3) and the relative risk for diarrhea mortality given zinc deficiency was 1.95 (95% CI 0.91 to 3.91).

Theoretical minimum-risk exposure level

The theoretical minimum-risk exposure level for proportion zinc deficient is zero percent deficient.

Vitamin A Deficiency Capstone Appendix

Flowchart

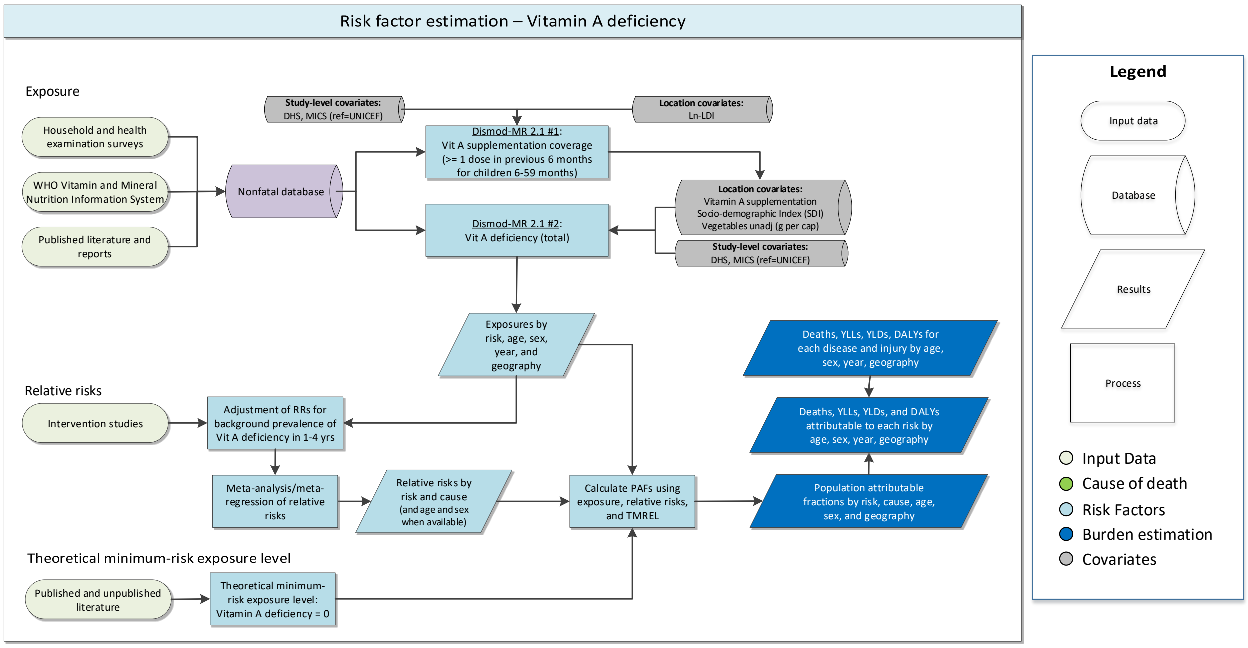

Input data & methodological summary

Exposure

Case definition

For GBD 2017, vitamin A deficiency is defined as serum retinol <70 µmol/L. We examined vitamin A deficiency as a risk factor in children aged 6 months to 5 years.

To ensure we were using as much information as possible, and therefore maximize the data basis of our estimates, we modeled Vitamin A deficiency sequentially. The first step was to estimate the coverage of Vitamin A supplementation. Although the typical metric on which supplementation is tracked is 2+ doses of Vitamin A in the previous 12 months for children under 5 years, most existing health surveys do not routinely provide sufficient information to calculate it. Our case definition for the supplementation model was therefore the proportion of children 6-59 months of age who received at least one dose of Vitamin A in the previous 6 months. Supplementation estimates were then used as a location-level covariate to guide exposure models of overall Vitamin A deficiency.

Input data

For GBD 2017, we used data from the WHO Vitamin and Mineral Nutrition Information System, health surveys such as DHS and MICS, and studies identified through literature review. This included updating the dataset to include all ages and all studies available in VMNIS as of April 2018. A separate systematic review was last conducted for GBD 2013. The PubMed search terms were: ((vitamin A deficiency[Title/Abstract]

AND prevalence[Title/Abstract]) AND ("2009"[Date – Publication] : "2013"[Date – Publication])). The table below shows the number of data points included in the final datasets. Exclusion criteria were:

1. Studies that were not population-based, e.g., hospital or clinic-based studies
2. Studies that did not provide primary data on epidemiological parameters, e.g., commentaries
3. Review articles
4. Case series
5. Self-reported cases

Table 1. Geographic representation of datasets used for three stages of Vitamin A deficiency risk factor burden estimation (number of data points per geography)

Geography	Supplementation (proportion)	Deficiency (prevalence)
**Global**	900	1540
**East Asia**	12	27
**Southeast Asia**	102	212
**Oceania**	24	54
**Central Asia**	51	66
**Central Europe**	2	13
**Eastern Europe**		3
**Australasia**		1
**Western Europe**		38
**Southern Latin America**		16
**High-income North America**		33
**Caribbean**	17	34
**Andean Latin America**	25	70
**Central Latin America**	33	212
**Tropical Latin America**	1	52
**North Africa and Middle East**	49	148
**South Asia**	61	96
**Central Sub-Saharan Africa**	60	8
**Eastern Sub-Saharan Africa**	182	220
**Southern Sub-Saharan Africa**	49	57
**Western Sub-Saharan Africa**	232	180

Modeling strategy

All Vitamin A deficiency estimates were made using DisMod-MR 2.1. As described above, we first estimated Vitamin A supplementation coverage. Although all data was from ages 6-59 months, we assumed no difference in age pattern of supplementation coverage and used the natural log of lag-distributed income per capita (LN-LDI) as a location-level covariate to inform estimates where data was absent. DHS and MICS data was cross-walked to the reference data source, which came from UNICEF (http://data.worldbank.org/indicator/SN.ITK.VITA.ZS).

Table 2: Covariate effects for Vitamin A supplementation model

Measure	Covariate	Type	Value	Exponentiated
**Prevalence**	MICS	Study-level	-0.6 (-0.76 — -0.45)	0.55 (0.47 — 0.64)
**Prevalence**	DHS	Study-level	-0.09 (-0.2 — 0.025)	0.91 (0.82 — 1.03)
**Prevalence**	LDI (I$ per capita)	Country-level	0.013 (0.00033 — 0.042)	1.01 (1.00 — 1.04)

Second, we estimated the age- and sex-specific prevalence of Vitamin A deficiency (serum retinol < 0.7 µmol/L). WHO VMNIS was the primary data source for this model and was supplemented with data from DHS and other health surveys where testing was performed. We assumed the following in our model: no excess mortality, birth prevalence is possible, incidence is decreasing after age 5 and remission is increasing after age 5. Data from subnational locations was crosswalked to the reference data sources of nationally-representative data. Females were found to have 1.09 times higher Vitamin A deficiency, although the uncertainty in that ratio ranged from 0.97 to 1.24. Location-level covariates were used for Vitamin A supplementation coverage from the above model as well as GBD 2016 Socio-demographic Index (SDI) numbers.

Table 3: Covariate effects for Vitamin A deficiency model

Measure	Covariate	Type	Value	Exponentiated
**Prevalence**	Sex	Study-level (x-cov)	-0.0091 (-0.088 — 0.065)	0.99 (0.92 — 1.07)
**Prevalence**	Subnational	Study-level (x-cov)	-0.28 (-0.44 — -0.1)	0.76 (0.64 — 0.90)
**Prevalence**	Vitamin A supplem. coverage rate	Country-level	-0.028 (-0.1 — -0.00071)	0.97 (0.90 — 1.00)
**Prevalence**	Socio-demographic Index	Country-level	-2.98 (-3 — -2.92)	0.051 (0.050 — 0.054)
**Prevalence**	vegetables unadjusted(g)	Country-level	-1.36 (-1.53 — -1.12)	0.26 (0.22 — 0.33)

Theoretical minimum-risk exposure level

The theoretical minimum risk exposure is that the prevalence of vitamin A deficiency is zero.

Relative risks

The relative risks were updated in GBD 2017 to reflect studies included in the most recently published systematic review by Imdad and colleagues.[1] The overall estimation strategy has not changed. For each trial identified by the systematic review, we adjusted the relative risk for the background prevalence of Vitamin A deficiency in 1-4 years from the GBD 2017 model described above. This adjustment assumes the effect of

supplementation is observed only in the fraction of the trial population that are Vitamin A deficient. Many studies evaluate either incidence or mortality. A subset of studies evaluated both incidence and cause-specific mortality as outcomes for the same cause. We found no statistical difference between the effect sizes of incidence and mortality in any of these studies so pooled all incidence and mortality observations as independent observations prior to meta-analysis. We then performed a fixed effects meta-analysis of all adjusted RRs to determine final outcomes to be included in GBD risk factor attribution estimates. Forest plots are shown in Figures 1-5; Final RRs are shown in Table 4. Three outcomes – diarrhea, lower respiratory infections (LRI), and measles – were found to be statistically significant after adjustment, pooling, and meta-analysis. Meningitis was non-significant. Malaria was significant, but only a single study was identified that evaluated this outcomes, which does not meet GBD causal criteria.

Table 4: Pooled relative risks for risk-outcome pairs included in GBD 2017

Cause	GBD 2016 RR	GBD 2017 RR
**Diarrhea**	1.6 (1.21 - 2.02)	**2.35 (2.17 - 2.54)**
**Measles**	2.4 (1.61 - 3.48)	**2.76 (2.01 - 3.78)**
**Lower Respiratory Infections (LRI)**		**1.23 (1.03 - 1.48)**
**Meningitis**		3.2 (0.69 - 14.75)
**Malaria**		3.65 (2.23 - 5.97)

Figure 1: Forest plot of RR of diarrhea in Vitamin A deficiency

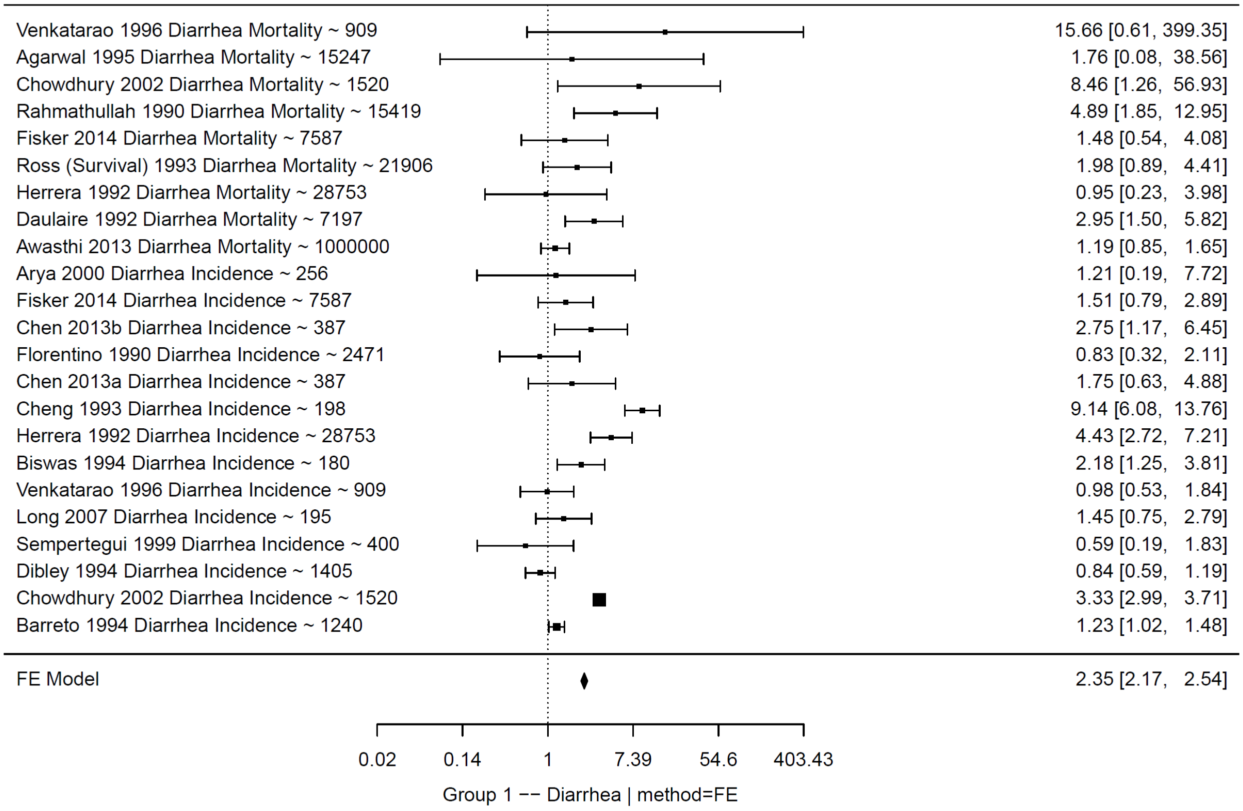
Venkatarao 1996 Diarrhea Mortality ~ 909
15.66 [0.61, 399.35]
Agarwal 1995 Diarrhea Mortality ~ 15247
1.76 [0.08, 38.56]
Chowdhury 2002 Diarrhea Mortality ~ 1520
8.46 [1.26, 56.93]
Rahmathullah 1990 Diarrhea Mortality ~ 15419
4.89 [1.85, 12.95]
Fisker 2014 Diarrhea Mortality ~ 7587
1.48 [0.54, 4.08]
Ross (Survival) 1993 Diarrhea Mortality ~ 21906
1.98 [0.89, 4.41]
Herrera 1992 Diarrhea Mortality ~ 28753
0.95 [0.23, 3.98]
Daulaire 1992 Diarrhea Mortality ~ 7197
2.95 [1.50, 5.82]
Awasthi 2013 Diarrhea Mortality ~ 1000000
1.19 [0.85, 1.65]
Arya 2000 Diarrhea Incidence ~ 256
1.21 [0.19, 7.72]
Fisker 2014 Diarrhea Incidence ~ 7587
1.51 [0.79, 2.89]
Chen 2013b Diarrhea Incidence ~ 387
2.75 [1.17, 6.45]
Florentino 1990 Diarrhea Incidence ~ 2471
0.83 [0.32, 2.11]
Chen 2013a Diarrhea Incidence ~ 387
1.75 [0.63, 4.88]
Cheng 1993 Diarrhea Incidence ~ 198
9.14 [6.08, 13.76]
Herrera 1992 Diarrhea Incidence ~ 28753
4.43 [2.72, 7.21]
Biswas 1994 Diarrhea Incidence ~ 180
2.18 [1.25, 3.81]
Venkatarao 1996 Diarrhea Incidence ~ 909
0.98 [0.53, 1.84]
Long 2007 Diarrhea Incidence ~ 195
1.45 [0.75, 2.79]
Sempertegui 1999 Diarrhea Incidence ~ 400
0.59 [0.19, 1.83]
Dibley 1994 Diarrhea Incidence ~ 1405
0.84 [0.59, 1.19]
Chowdhury 2002 Diarrhea Incidence ~ 1520
3.33 [2.99, 3.71]
Barreto 1994 Diarrhea Incidence ~ 1240
1.23 [1.02, 1.48]
FE Model
2.35 [2.17, 2.54]
0.02
0.14
1
7.39
54.6
403.43
Group 1 -- Diarrhea | method=FE

Low Birth Weight and Short Gestation Capstone Appendix

Flowchart

Low birth weight and Short gestation Risk Factors

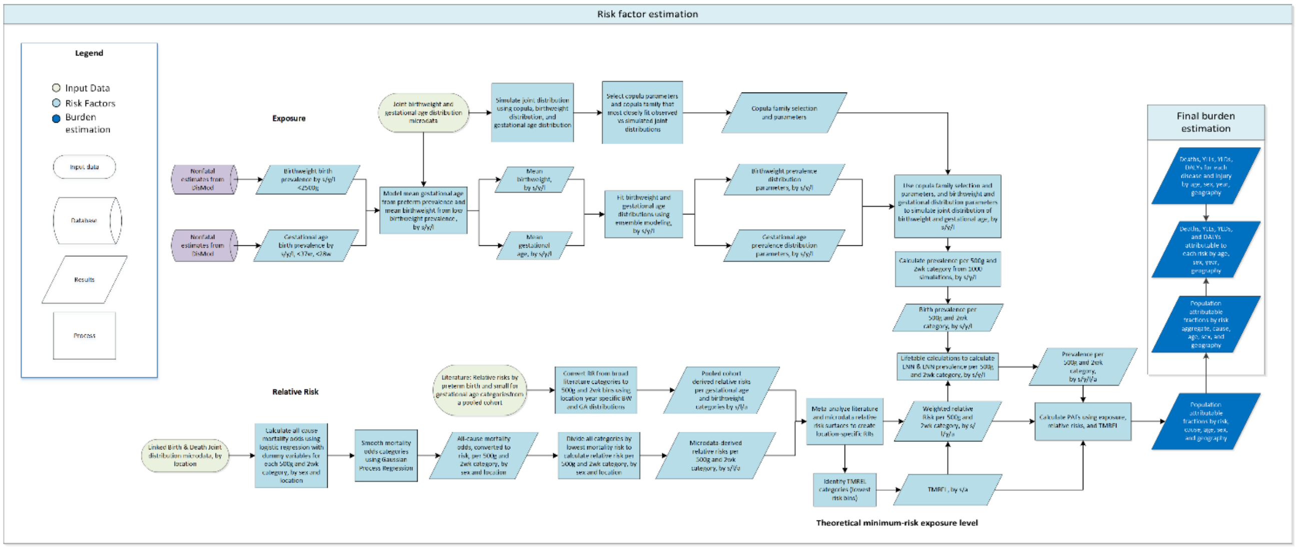

Input data and methodological summary

The "Low Birth Weight and Short Gestation" (LBWSG) risk factor and its child risks "Low Birth Weight for Gestation" and "Short Gestation for Birth Weight" first were included as risk factors in GBD 2016.

Although low birth weight for gestation and short gestation for birth weight are separate risk factors, the exposures and relative risks for both are estimated jointly through the low birth weight and short gestation parent risk factor. As of GBD 2017, LBWSG are the only risk factors estimated jointly.

Case definition

The meaning of the "low birth weight" and "short gestation" in GBD have subtle definitional differences compared to other usages of "low birth weight" and "short gestation" in literature. The term "low birth weight" has historically been used to refer to birth weight (BW) less than 2500 grams. However, because the goal of the GBD risk factors analysis is to quantify the entirety of attributable burden due to each risk factor, the GBD definition of "low birth weight" therefore refers to all birth weight below the Theoretical Minimum Risk Exposure Level (TMREL) for birth weight. Likewise, new-borns have been typically been classified into gestational age (GA) categories of "extremely preterm" (<28 weeks of gestation), "very preterm" (28-<32 weeks of gestation), and "moderate to late preterm" (32-<37 weeks of gestation). "Short gestation" in GBD refers to all gestational ages below the gestational age TMREL.

Exposures and relative risks for the GBD Low birth weight and short gestation risk factors are divided into joint 500-gram birth weight and 2-week gestational age combinations. The lowest risk overall 500-gram/2-week bin is the overall TMREL. The univariate TMRELs vary with GA and BW. The lowest risk GA varies by BW category and the lowest risk BWs vary with GA category. The latter are used to quantify univariate attributable risk. Under this framework, all attributable burden under the joint TMREL is referred to jointly as burden of LBWSG. All attributable burden to BWs under the TMREL for each GA category are, on aggregate, "low birth weight" and all attributable burden to GAs under the TMREL for each BW category are, on aggregate, "short gestation." Each combination of 500-grams and 2-wks is associated with a relative risk for mortality by neonatal period (early and late neonatal) and by the causes listed in Table 2 and described below, and relative to the joint TMREL.

Exposure

Input data

To model the joint distribution of exposure of low birth weight and short gestation for each location, year, and sex estimated in GBD 2017, three types of information are used:

- Distribution of gestational age for each location, year, and sex
- Distribution of birth weight for each location, year, and sex

- Copula family and parameters, specifying correlation between gestational age and birth weight distributions

Modelling strategy

Distributions of birth weight & gestational age

To model the joint distribution of birth weight and gestational age for every location-sex-year, ensemble model methods standard to GBD risk factors (described elsewhere in the methods appendix), are first used to create separate distributions of birth weight and gestational age for every location-sex-year.

Microdata is the most ideal data source for modelling distributions; however, microdata is not widely available for birth weight and is more scarce for gestational age. Categorical prevalence data is much more readily available, and from a wider range of locations and years, for low birth weight (<2500g), extremely preterm (<28 weeks of gestation), very preterm (28-32 weeks of gestation), moderate to late preterm (32-37 weeks of gestation), and preterm birth (<37 weeks of gestation). From GBD 2010 to GBD 2015, this categorical data has been used model birth prevalence of preterm birth by gestational age (<28 weeks, 28-<32 weeks, and 32-<37 weeks) and low birth weight (<2500g) for every location, sex, and year estimated in GBD. Starting in GBD 2016 with the introduction of the LBWSG risk factors, the full distributions at birth have been modelled for gestational age and birth weight for all GBD locations, estimation years, and both sexes. The gestational age and birth weight distributions are then aggregated into the categorical estimates of <28 weeks, 28-<32 weeks, 32-<37 weeks gestation, and <2500 g birth weight.

Ensemble model methods standard to GBD are used to model the distribution at birth of gestational age and birth weight. Gestational age ensemble distribution models use the prevalence of <37 weeks gestation, the prevalence of <28 weeks gestation, and mean gestational age per each location-year-sex as inputs into the model. Birth weight distribution models use the prevalence of <2500 grams birth weight and mean birth weight per each location-year-sex. Prevalence of <37 weeks gestation and of <2500 grams birth weight was estimated for all location-year-sexes using STGPR modelling processes standard to GBD.

Low birth weight (<2500 grams) data was extracted from literature, vital registration systems, and surveys. DHS survey data were observed to have high missingness; to correct for the missingness, birth weight was imputed using the Amelia package in R. Birth weight was predicted using standard Amelia imputation methods from the following variables also in the DHS surveys: urbanicity, sex, birthweight recorded on card, birth order, maternal education, paternal education, child age, child weight, child height, mother's age at birth, mother's weight, shared toilet facility, and household water treated. Data counts for categorical prevalence models are listed in Table 1.

Table 1: Data Counts for Categorical Prevalence Models

	<28 weeks	<37 weeks	<2500 grams
Site-years (total)	1872	2420	2980
Number of GBD regions with data (out of 21 regions)	14	21	21
Number of GBD super-regions with data (out of 7 super-regions)	6	7	7

Global ensemble weights for gestational age were derived by using a 3 million sample of all available microdata in Table 2 to select the ensemble weights. Of the exponential, gamma, inverse gamma, Weibull, log normal, and normal distributions, the three distribution families that received the highest weights were the Weibull (87%), normal (4%), and inverse gamma (4%) distributions. Global ensemble weights for birth weight were derived using a 3 million sample of all available microdata in Table 2, in addition to birth weight microdata available primarily through the DHS and MICS surveys. Of the exponential, gamma, inverse gamma, Weibull, log normal, and normal distributions, the three distribution families that received the highest weights were the log normal (38%), normal (32%), and Weibull (20%) distributions.

Ordinary least squares was used to model mean gestational age for all location-year-sexes by regressing mean gestational age on prevalence of <37 weeks gestation per location-year. All available microdata (Table 2) was used to fit the model. OLS was also used to model mean birth weight by regressing prevalence of <2500 g birth weight per location-year. All available joint microdata (Table 2), as well as additional birth weight microdata extracted primarily through DHS and MICS surveys, was used to fit the model. As estimates of prevalence of <37 weeks gestation and prevalence of <2500g birth weight are available for all location-year-sexes through STGPR models, mean gestational age and mean birth weight were predicted for all location-year-sexes.

Copula optimisation

In order to model the joint distribution of gestational age and birth weight from separate distributions, information is needed about the correlation between the two distributions. Distributions of gestational age and birth weight are not independent; the Spearman correlation for each country where joint microdata was available (Table 2), pooling across all years of data available, ranged from 0.25-0.49. The overall Spearman correlation was 0.38, pooling across all countries in the dataset.

Table 3: Summary of Data Inputs

Location	**Years of data**	**Total births***	**Format of data**	**Spearman correlation**	**Used in Ensemble**	**Used in Copula**	**Used in Relative**

					Weight Selection	Parameter Selection	Risk Models
BRA	2016	2,854,380	Microdata	0.37	Yes	Yes	No
ECU	2003-2015	2,473,039	Microdata	0.34	Yes	Yes	No
ESP	1990-2014	8,537,220	Microdata	0.42	Yes	Yes	No
JPN	1995-2015	23,644,506	Tabulations	0.41	No	No	Yes
MEX	2008-2012	10,256,117	Microdata	0.35	Yes	Yes	No
NOR	1990-2014	1,489,210	Microdata	0.44	Yes	Yes	Yes
NZL	1990-2016	1,600,501	Microdata	0.25	Yes	Yes	Yes
SGP	1993-2015	972,775	Tabulations	0.41	No	No	Yes
TWN	1998-2002	1,331,760	Tabulations	0.38	No	No	Yes
URY	1996-2014	698,622	Microdata	0.49	Yes	Yes	No
USA	1990-2014	81,929,879	Microdata	0.38	Yes	Yes	Yes

** Pooled across all year and sexes, excluding data missing year of birth, gestational age, or birth weight*

Copula modelling is used to model joint distributions between the birth weight and gestational age marginal distributions. The Copula and VineCopula packages in R were used to select the optimal copula family and copula parameters to model the joint distribution, using joint microdata from the country-years in Table 2. The copula family selected from the microdata was "Survival BB8", with theta parameter set to 1.75 and delta parameter set to 1.

The joint distribution of birth weight and gestational age per location-year-sex was modelled using the global copula family and parameters selected and the location-year-sex gestational age and birth weight distributions. The joint distribution was simulated 100 times to capture uncertainty. Each simulation consisted of 100,000 simulated joint birth weight and gestational age data points. Each joint distribution was divided into 500g by 2wk bins to match the categorical bins of the relative risk surface. Birth prevalence was then calculated for each 500g by 2wk bin.

Estimating Early Neonatal Prevalence & Late Neonatal Prevalence from Birth Cohorts

Early neonatal prevalence and late neonatal prevalence was estimated using life table approaches for each 500g & 2wk bin. Using the all-cause early neonatal mortality rate for each location-year-sex, births per location-year-sex-bin, and the relative risks for each location-year-sex-bin in the early neonatal period, the all-cause early neonatal mortality rate was calculated for each location-year-sex-*bin*. The early neonatal mortality rate per bin was used to calculate the number of survivors at 7 days and prevalence in the early neonatal period. Using the same process, the all-cause late neonatal mortality rate for each location-year-sex was paired with the number of survivors at 7 days and late neonatal relative risks per bin to calculate late neonatal prevalence and survivors at 28 days.

Relative risks & theoretical minimum-risk exposure level

Input data

In the Norway, New Zealand, and US Linked Birth/Death Cohort microdata datasets, live births are reported with gestational age, birth weight, and an indicator of death at 7 days and 28 days. For this analysis, gestational age was grouped into two-week categories, and birth weight was grouped into 500-gram categories. The Taiwan, Japan, and Singapore datasets were prepared in tabulations of joint 500-gram and two-week categories.

Modelling strategy

For each location, data was pooled across years, and the risk of all-cause mortality at the early neonatal period and late neonatal period at joint birth weight and gestational age combinations was calculated. In all datasets except for the United States, sex-specific data were combined to maximise sample size. The United States analyses were sex-specific. To calculate relative risk at each 500g and 2wk combination, logistic regression was first used to calculate mortality odds for each joint 2-week gestational age and 500-gram birth weight category. Mortality odds were smoothed with Gaussian Process Regression, with the independent distributions of mortality odds by birth weight and mortality odds by gestational age serving as priors in the regression.

A pooled country analysis[37] of mortality risk in the early neonatal period and late neonatal period by SGA category in developing countries in Asia and Sub-Saharan Africa were also converted into 500-gram and 2-week bin mortality odds surfaces. The relative risk surfaces produced from microdata and the Asia and Africa surfaces produced from the pooled country analysis were meta-analyzed, resulting in a meta-analysed mortality odds surface for each location. The meta-analysed mortality odds surface for each location was smoothed using Gaussian Process Regression and then converted into mortality risk. To calculate mortality relative risks, the risk of each joint 2-week gestational age and 500-gram birth weight category were divided by the risk of mortality in the joint gestational age and birth weight category with the lowest mortality risk.

For each of the country-derived relative risk surfaces, the 500 g and 2-week gestational age joint bin with the lowest risk was identified. This bin differed within each country dataset. To identify the universal 500 g and 2-week gestational age category that would serve as the universal TMREL for our analysis, we chose the bins that was identified to be the TMREL in each country dataset to contribute to the universal TMREL. Therefore, the joint categories that served as our universal TMREL for the LBWSG risk factor were “38-40 weeks of gestation and 3500-4000 grams", "38-40 weeks of gestation and 4000-4500 grams", and "40-42 weeks of gestation and 4000-4500 grams". As the joint TMREL, all three categories were assigned to a relative risk equal to 1.

PAF calculations

The total PAF for the low birth weight and short gestation joint risk factor is calculated by summing the PAF calculated from each 500g x 2wk category, with the lowest risk category among all the 500g x 2wk categories serving as the TMREL. The equation for calculating PAF for each 500g x 2wk category is:

$$PAF_{joasgt} = \frac{\sum_{x=1}^{u} RR_{joast}(x)P_{jasgt}(x) - RR_{joasg}(TMRE_{jas})}{\sum_{x=1}^{u} RR_{joas}(x)P_{jasgt}(x)}$$

To calculate the overall PAF for the short gestation for birth weight risk factor, PAF was once again calculated for each joint 500-gram and 2-week category. Unlike the joint PAF calculation, which used only one TMREL for all 500-gram and 2-week categories, the joint 500-gram and 2-week category with the lowest risk for each 500-gram birth weight grouping served as the TMREL for that 500-gram birth weight grouping. For example, the [3000, 3500) gram birth weight grouping contains five joint categories: [34, 36) weeks and [3000, 3500) grams; [36, 37) weeks and [3000, 3500) grams; [37, 38) weeks and [3000, 3500) grams; [38, 40) weeks and [3000, 3500) grams; and [40, 42) weeks and [3000, 3500) grams. The [40, 42) weeks and [3000, 3500) grams joint category has the lowest risk, and so it serves as the TMREL for the [3000, 3500) gram birth weight grouping. In the Relative Risk surface figures, a birth weight grouping is one "column" of the birth weight and gestational age matrix.

The overall PAF for the short gestation for birth weight risk factor was then calculated for all the joint 500-gram and 2-week categories using the formula below:

$$PAF_{1..i} = 1 - \prod_{i=1}^{n}(1 - PAF_i)$$

The same methodology was applied to calculate the total PAF for the low birth weight for gestation risk factor, using two-week gestational age categories (each "row" of the matrix) instead of 500-gram birth weight categories. For example, the [24, 26) weeks gestational age grouping contains three joint categories: [0, 500) grams and [24, 26) weeks; [500, 1000) grams and [24, 26) weeks; and [1000, 1500) grams and [24, 26) weeks. The [1000, 1500) grams and [24, 26) weeks joint category has the lowest risk, and so it serves as the TMREL for the [24, 26) weeks gestational age grouping.

After the short gestation for birth weight PAF and low birth weight for gestational age PAF were calculated, they were then scaled so that the sum of the short gestation for birth weight PAF and low birth weight for gestation PAF equal the low birth weight and short gestation parent PAF calculated for each location/year/sex/age group.

Table for the relative risks for diarrhea mortality given low birthweight and short gestation joint categories by sex and early neonatal and late neonatal age groups.

Category	Sex	Early neonatal (0-6 days)	Late neonatal (7-27 days)
Birth prevalence - [0, 24) wks, [0, 500) g	Males	1564.792 (1056.542 to 2116.062)	618.595 (458.842 to 812.921)
Birth prevalence - [0, 24) wks, [0, 500) g	Females	1600.122 (1050.664 to 2211.877)	713.571 (526.178 to 921.018)
Birth prevalence - [0, 24) wks, [500, 1000) g	Males	1155.815 (825.412 to 1506.837)	457.5 (352.552 to 573.483)
Birth prevalence - [0, 24) wks, [500, 1000) g	Females	1169.123 (802.003 to 1617.979)	515.406 (396.713 to 641.541)
Birth prevalence - [24, 26) wks, [500, 1000) g	Males	955.583 (723.748 to 1244.265)	443.357 (363.03 to 534.695)
Birth prevalence - [24, 26) wks, [500, 1000) g	Females	947.143 (702.662 to 1237.093)	487.549 (387.307 to 603.498)
Birth prevalence - [26, 28) wks, [500, 1000) g	Males	497.817 (377.617 to 648.547)	330.886 (261.438 to 401.709)
Birth prevalence - [26, 28) wks, [500, 1000) g	Females	483.682 (354.946 to 629.517)	344.618 (274.427 to 419.864)
Birth prevalence - [30, 32) wks, [500, 1000) g	Males	236.614 (163.821 to 324.502)	149.995 (117.866 to 188.368)
Birth prevalence - [30, 32) wks, [500, 1000) g	Females	229.197 (157.606 to 317.194)	152.117 (120.779 to 190.583)
Birth prevalence - [28, 30) wks, [500, 1000) g	Males	297.629 (214.953 to 396.586)	216.995 (173.321 to 271.466)
Birth prevalence - [28, 30) wks, [500, 1000) g	Females	281.056 (198.176 to 386.635)	219.884 (174.264 to 272.704)
Birth prevalence - [26, 28) wks, [1000, 1500) g	Males	267.91 (210.177 to 332.92)	164.167 (132.898 to 200.569)
Birth prevalence - [26, 28) wks, [1000, 1500) g	Females	266.509 (197.461 to 346.932)	174.222 (137.431 to 217.349)
Birth prevalence - [34, 36) wks, [1000, 1500) g	Males	142.056 (98.086 to 197.774)	52.86 (42.914 to 64.617)
Birth prevalence - [34, 36) wks, [1000, 1500) g	Females	141.899 (95.864 to 197.656)	57.421 (46.452 to 71.339)
Birth prevalence - [28, 30) wks, [1500, 2000) g	Males	127.966 (97.178 to 167.026)	50.018 (40.539 to 61.919)
Birth prevalence - [28, 30) wks, [1500, 2000) g	Females	130.924 (96.513 to 172.188)	57.275 (46.36 to 70.038)
Birth prevalence - [28, 30) wks, [1000, 1500) g	Males	158.563 (120.99 to 204.947)	103.32 (83.486 to 127.144)

Birth prevalence - [28, 30) wks, [1000, 1500) g	Females	153.905<br>(112.327 to 200.786)	107.529<br>(86.954 to 131.78)
Birth prevalence - [32, 34) wks, [1000, 1500) g	Males	117.142<br>(81.354 to 161.101)	53.185<br>(43.049 to 66.274)
Birth prevalence - [32, 34) wks, [1000, 1500) g	Females	115.171<br>(79.363 to 159.206)	56.034<br>(45.982 to 68.36)
Birth prevalence - [30, 32) wks, [1000, 1500) g	Males	119.308<br>(87.769 to 160.885)	67.163<br>(54.863 to 82.638)
Birth prevalence - [30, 32) wks, [1000, 1500) g	Females	115.448<br>(84.272 to 156.425)	69.14<br>(55.873 to 85.012)
Birth prevalence - [37, 38) wks, [1500, 2000) g	Males	62.972<br>(46.159 to 83.484)	24.148<br>(20.066 to 29.406)
Birth prevalence - [37, 38) wks, [1500, 2000) g	Females	59.988<br>(43.974 to 79.053)	26.719<br>(21.746 to 32.816)
Birth prevalence - [36, 37) wks, [1500, 2000) g	Males	60.218<br>(43.669 to 82.48)	23.031<br>(18.793 to 28.483)
Birth prevalence - [36, 37) wks, [1500, 2000) g	Females	58.527<br>(42.172 to 80.557)	25.143<br>(20.331 to 30.566)
Birth prevalence - [30, 32) wks, [2000, 2500) g	Males	67.971<br>(50.354 to 88.935)	18.03<br>(14.621 to 22.103)
Birth prevalence - [30, 32) wks, [2000, 2500) g	Females	69.383<br>(49.108 to 94.583)	22.069<br>(17.836 to 27.163)
Birth prevalence - [30, 32) wks, [1500, 2000) g	Males	77.369<br>(59.702 to 99.232)	31.079<br>(25.786 to 36.724)
Birth prevalence - [30, 32) wks, [1500, 2000) g	Females	76.134<br>(56.885 to 100.996)	34.756<br>(28.764 to 41.849)
Birth prevalence - [34, 36) wks, [1500, 2000) g	Males	55.555<br>(39.553 to 75.104)	21.346<br>(17.677 to 26.143)
Birth prevalence - [34, 36) wks, [1500, 2000) g	Females	54.335<br>(38.617 to 75.24)	23.046<br>(18.743 to 28.287)
Birth prevalence - [32, 34) wks, [1500, 2000) g	Males	57.155<br>(42.484 to 73.651)	23.114<br>(19.028 to 27.915)
Birth prevalence - [32, 34) wks, [1500, 2000) g	Females	56.101<br>(39.794 to 76.295)	25.149<br>(20.615 to 30.388)
Birth prevalence - [32, 34) wks, [2000, 2500) g	Males	37.444<br>(29.026 to 48.227)	12.233<br>(10.252 to 14.477)
Birth prevalence - [32, 34) wks, [2000, 2500) g	Females	36.874<br>(26.658 to 49.653)	14.384<br>(12.095 to 17.03)
Birth prevalence - [40, 42) wks, [2000, 2500) g	Males	18.092<br>(13.292 to 23.719)	9.23<br>(7.037 to 11.454)
Birth prevalence - [40, 42) wks, [2000, 2500) g	Females	15.574<br>(11.516 to 20.778)	9.975<br>(7.82 to 12.46)
Birth prevalence - [38, 40) wks, [2000, 2500) g	Males	13.104<br>(9.829 to 16.99)	8.198<br>(6.786 to 9.959)

Birth prevalence - [38, 40) wks, [2000, 2500) g	Females	11.308<br>(8.389 to 14.38)	8.577<br>(7.04 to 10.449)
Birth prevalence - [32, 34) wks, [2500, 3000) g	Males	33.063<br>(24.393 to 43.503)	8.441<br>(6.822 to 10.431)
Birth prevalence - [32, 34) wks, [2500, 3000) g	Females	32.812<br>(23.439 to 45.567)	10.398<br>(8.227 to 13.042)
Birth prevalence - [34, 36) wks, [2000, 2500) g	Males	21.925<br>(16.305 to 29.433)	9.367<br>(7.859 to 11.112)
Birth prevalence - [34, 36) wks, [2000, 2500) g	Females	21.297<br>(15.657 to 28.761)	10.295<br>(8.548 to 12.273)
Birth prevalence - [37, 38) wks, [2000, 2500) g	Males	13.0<br>(10.102 to 16.456)	8.096<br>(6.724 to 9.676)
Birth prevalence - [37, 38) wks, [2000, 2500) g	Females	11.563<br>(8.805 to 15.11)	8.467<br>(6.994 to 10.342)
Birth prevalence - [36, 37) wks, [2000, 2500) g	Males	14.401<br>(10.789 to 18.654)	8.221<br>(6.917 to 9.923)
Birth prevalence - [36, 37) wks, [2000, 2500) g	Females	13.513<br>(9.817 to 17.942)	8.654<br>(7.215 to 10.369)
Birth prevalence - [34, 36) wks, [2500, 3000) g	Males	13.419<br>(10.387 to 16.819)	5.562<br>(4.646 to 6.696)
Birth prevalence - [34, 36) wks, [2500, 3000) g	Females	13.266<br>(9.666 to 17.689)	6.395<br>(5.292 to 7.606)
Birth prevalence - [34, 36) wks, [4000, 4500) g	Males	23.096<br>(14.708 to 35.098)	2.895<br>(2.245 to 3.716)
Birth prevalence - [34, 36) wks, [4000, 4500) g	Females	25.038<br>(15.763 to 37.255)	3.778<br>(2.855 to 4.925)
Birth prevalence - [34, 36) wks, [3000, 3500) g	Males	14.006<br>(10.222 to 18.478)	4.322<br>(3.449 to 5.338)
Birth prevalence - [34, 36) wks, [3000, 3500) g	Females	14.375<br>(10.269 to 20.114)	5.265<br>(4.145 to 6.564)
Birth prevalence - [36, 37) wks, [2500, 3000) g	Males	4.874<br>(4.014 to 5.713)	3.699<br>(3.134 to 4.374)
Birth prevalence - [36, 37) wks, [2500, 3000) g	Females	4.609<br>(3.731 to 5.61)	3.898<br>(3.235 to 4.67)
Birth prevalence - [34, 36) wks, [3500, 4000) g	Males	18.024<br>(12.279 to 25.547)	3.657<br>(2.838 to 4.675)
Birth prevalence - [34, 36) wks, [3500, 4000) g	Females	19.263<br>(12.567 to 27.924)	4.634<br>(3.576 to 5.989)
Birth prevalence - [37, 38) wks, [2500, 3000) g	Males	3.306<br>(2.82 to 3.843)	3.194<br>(2.691 to 3.803)
Birth prevalence - [37, 38) wks, [2500, 3000) g	Females	2.991<br>(2.496 to 3.61)	3.242<br>(2.745 to 3.817)
Birth prevalence - [40, 42) wks, [2500, 3000) g	Males	3.771<br>(3.002 to 4.693)	3.175<br>(2.56 to 3.923)

Birth prevalence - [40, 42) wks, [2500, 3000) g	Females	3.244<br>(2.486 to 4.159)	3.228<br>(2.605 to 3.985)
Birth prevalence - [38, 40) wks, [2500, 3000) g	Males	2.755<br>(2.274 to 3.309)	2.944<br>(2.44 to 3.548)
Birth prevalence - [38, 40) wks, [2500, 3000) g	Females	2.376<br>(1.91 to 2.886)	2.938<br>(2.434 to 3.503)
Birth prevalence - [36, 37) wks, [3000, 3500) g	Males	3.774<br>(3.094 to 4.497)	2.466<br>(2.058 to 2.929)
Birth prevalence - [36, 37) wks, [3000, 3500) g	Females	3.73<br>(2.981 to 4.646)	2.715<br>(2.277 to 3.218)
Birth prevalence - [36, 37) wks, [4000, 4500) g	Males	6.826<br>(5.212 to 9.045)	1.77<br>(1.491 to 2.082)
Birth prevalence - [36, 37) wks, [4000, 4500) g	Females	7.269<br>(5.144 to 9.821)	2.177<br>(1.786 to 2.607)
Birth prevalence - [36, 37) wks, [3500, 4000) g	Males	4.544<br>(3.64 to 5.622)	2.057<br>(1.74 to 2.44)
Birth prevalence - [36, 37) wks, [3500, 4000) g	Females	4.662<br>(3.577 to 6.014)	2.398<br>(1.98 to 2.864)
Birth prevalence - [37, 38) wks, [3000, 3500) g	Males	2.007<br>(1.759 to 2.293)	1.888<br>(1.613 to 2.224)
Birth prevalence - [37, 38) wks, [3000, 3500) g	Females	1.925<br>(1.582 to 2.328)	1.972<br>(1.669 to 2.313)
Birth prevalence - [37, 38) wks, [4000, 4500) g	Males	3.28<br>(2.596 to 4.133)	1.335<br>(1.171 to 1.532)
Birth prevalence - [37, 38) wks, [4000, 4500) g	Females	3.521<br>(2.649 to 4.5)	1.559<br>(1.333 to 1.835)
Birth prevalence - [37, 38) wks, [3500, 4000) g	Males	2.128<br>(1.833 to 2.466)	1.505<br>(1.299 to 1.76)
Birth prevalence - [37, 38) wks, [3500, 4000) g	Females	2.142<br>(1.694 to 2.67)	1.661<br>(1.411 to 1.961)
Birth prevalence - [40, 42) wks, [3000, 3500) g	Males	1.436<br>(1.245 to 1.65)	1.47<br>(1.199 to 1.8)
Birth prevalence - [40, 42) wks, [3000, 3500) g	Females	1.326<br>(1.069 to 1.614)	1.465<br>(1.188 to 1.775)
Birth prevalence - [38, 40) wks, [3000, 3500) g	Males	1.33<br>(1.155 to 1.53)	1.559<br>(1.305 to 1.851)
Birth prevalence - [38, 40) wks, [3000, 3500) g	Females	1.224<br>(1.0 to 1.492)	1.564<br>(1.304 to 1.847)
Birth prevalence - [38, 40) wks, [4000, 4500) g	Males	1.787<br>(1.453 to 2.182)	1.175<br>(1.005 to 1.371)
Birth prevalence - [38, 40) wks, [4000, 4500) g	Females	1.877<br>(1.467 to 2.388)	1.224<br>(1.022 to 1.465)
Birth prevalence - [38, 40) wks, [3500, 4000) g	Males	1.785<br>(1.478 to 2.147)	1.173<br>(1.0 to 1.377)

Birth prevalence - [38, 40) wks, [3500, 4000) g	Females	1.892<br>(1.481 to 2.352)	1.23<br>(1.03 to 1.46)
Birth prevalence - [40, 42) wks, [3500, 4000) g	Males	1.0<br>(1.0 to 1.0)	1.003<br>(1.0 to 1.046)
Birth prevalence - [40, 42) wks, [3500, 4000) g	Females	1.002<br>(1.0 to 1.013)	1.001<br>(1.0 to 1.006)
Birth prevalence - [40, 42) wks, [4000, 4500) g	Males	1.0<br>(1.0 to 1.0)	1.0<br>(1.0 to 1.0)
Birth prevalence - [40, 42) wks, [4000, 4500) g	Females	1.0<br>(1.0 to 1.0)	1.0<br>(1.0 to 1.0)
Birth prevalence - [28, 30) wks, [2000, 2500) g	Males	117.172<br>(83.895 to 158.056)	27.726<br>(21.877 to 34.972)
Birth prevalence - [28, 30) wks, [2000, 2500) g	Females	121.682<br>(84.349 to 171.375)	33.983<br>(26.101 to 43.404)
Birth prevalence - [28, 30) wks, [2500, 3000) g	Males	77.948<br>(54.687 to 105.047)	16.608<br>(12.653 to 21.188)
Birth prevalence - [28, 30) wks, [2500, 3000) g	Females	79.193<br>(54.099 to 112.236)	20.387<br>(15.089 to 26.434)
Birth prevalence - [28, 30) wks, [3000, 3500) g	Males	42.199<br>(29.891 to 57.227)	10.082<br>(7.777 to 13.056)
Birth prevalence - [28, 30) wks, [3000, 3500) g	Females	42.551<br>(28.25 to 63.209)	11.989<br>(9.084 to 15.544)
Birth prevalence - [30, 32) wks, [2500, 3000) g	Males	58.722<br>(42.419 to 78.873)	12.115<br>(9.518 to 15.521)
Birth prevalence - [30, 32) wks, [2500, 3000) g	Females	59.522<br>(42.058 to 82.793)	15.364<br>(11.936 to 19.581)
Birth prevalence - [30, 32) wks, [3000, 3500) g	Males	45.67<br>(32.014 to 65.531)	8.381<br>(6.362 to 10.842)
Birth prevalence - [30, 32) wks, [3000, 3500) g	Females	46.104<br>(30.243 to 66.207)	10.506<br>(8.041 to 13.513)
Birth prevalence - [30, 32) wks, [3500, 4000) g	Males	36.334<br>(21.558 to 54.813)	5.698<br>(4.293 to 7.349)
Birth prevalence - [30, 32) wks, [3500, 4000) g	Females	37.931<br>(22.692 to 61.276)	6.892<br>(5.175 to 9.058)
Birth prevalence - [32, 34) wks, [3000, 3500) g	Males	34.016<br>(23.515 to 48.37)	6.577<br>(4.934 to 8.436)
Birth prevalence - [32, 34) wks, [3000, 3500) g	Females	34.585<br>(22.909 to 49.754)	8.314<br>(6.362 to 10.789)
Birth prevalence - [32, 34) wks, [3500, 4000) g	Males	36.248<br>(23.158 to 54.67)	5.068<br>(3.761 to 6.741)
Birth prevalence - [32, 34) wks, [3500, 4000) g	Females	38.098<br>(23.301 to 59.429)	6.476<br>(4.666 to 8.689)
Birth prevalence - [36, 37) wks, [1000, 1500) g	Males	166.686<br>(118.487 to 222.581)	57.535<br>(45.999 to 71.742)

Birth prevalence - [36, 37) wks, [1000, 1500) g	Females	169.725<br>(119.017 to 229.008)	63.564<br>(50.068 to 80.703)
Birth prevalence - [38, 40) wks, [1000, 1500) g	Males	174.066<br>(125.125 to 232.507)	57.966<br>(44.393 to 73.241)
Birth prevalence - [38, 40) wks, [1000, 1500) g	Females	171.557<br>(121.585 to 237.047)	65.208<br>(48.821 to 84.308)
Birth prevalence - [38, 40) wks, [1500, 2000) g	Males	67.302<br>(49.547 to 89.055)	25.206<br>(20.365 to 31.168)
Birth prevalence - [38, 40) wks, [1500, 2000) g	Females	62.19<br>(45.884 to 83.445)	28.05<br>(22.625 to 35.139)
Birth prevalence - [40, 42) wks, [1500, 2000) g	Males	76.673<br>(56.177 to 102.468)	25.785<br>(19.387 to 34.168)

Suboptimal Breastfeeding Capstone Appendix

Flowchart

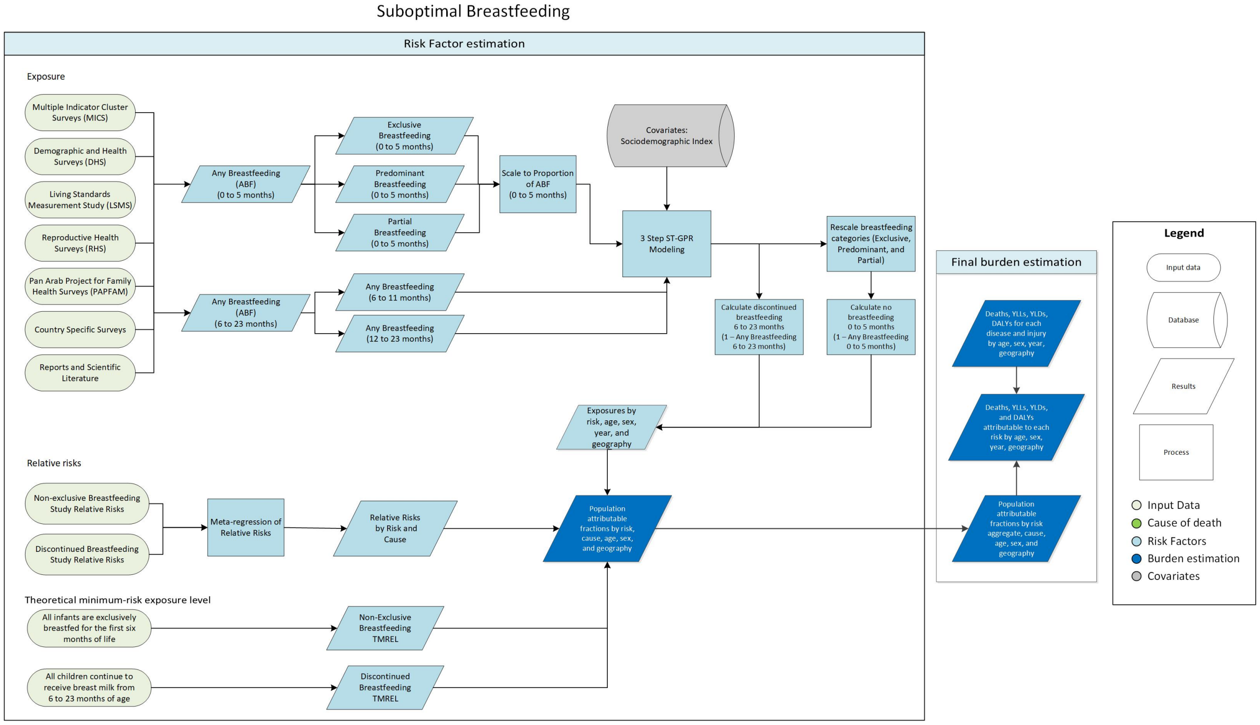

Definitions

Exposure to suboptimal breastfeeding is composed of 2 distinct categories: non-exclusive breastfeeding and discontinued breastfeeding.

Non-exclusive breastfeeding is defined as the proportion of children under 6 months of age who are not exclusively breastfed. We then parse those not exclusively breastfed into 3 categories – predominant, partial, and no breastfeeding. Exclusive breastfeeding is defined as the proportion of children who receive no other food or drink except breast milk (allowing for ORS, drops, or syrups containing vitamins, minerals, or medicines). Predominant breastfeeding is the proportion of children whose predominant source of nourishment is breastmilk but also receive other liquids. Partial breastfeeding refers to those infants who receive breastmilk as well as food and liquids, including non-human milk and formula. No breastfeeding refers to infants who do not receive breast milk as a source of nourishment.

Discontinued breastfeeding is defined as the proportion of children between 6 to 23 months who receive no breast milk as a source of nourishment.

Input Data

We made substantial exposure data updates for GBD 2017, including extracting identified surveys not included in previous rounds and re-extracting all surveys for new GBD 2017 subnational locations. We searched the Global Health Data Exchange

(GHDx) database for sources using the keyword "Breastfeeding." Of 2,026 potential sources identified, we extracted 1,081 unique country-years of data (2,262 unique geography-years, including subnational geographies) that met our inclusion criteria. The data used in the analysis consists mostly of processed individual-level microdata from surveys; in the cases where microdata was unavailable, we used reported tabulated data from survey reports and scientific literature. Data used to categorize type of non-exclusive breastfeeding (predominant, partial, and none) come from surveys with 24-hour dietary logs based on maternal recall.

Exposure Modelling

Using the processed microdata and tabulated data from reports, we generated a complete time series from 1980 to 2017 for the prevalence of breastfeeding patterns for children 0 to 5 months and 6 to 23 months using a three-step spatio-temporal Gaussian process regression modelling process.

First, we estimated a robust linear regression using each geography's sociodemographic index as a covariate. The following linear model was used for the estimation of breastfeeding indicators:

$$logit(P_{x,c,t}) = \beta_0 + \beta_1 SDI_{\mathrm{c,t}} + \alpha_c + \gamma_{R[c]} + \omega_{\mathrm{SR[c]}} + \varepsilon_{c,t}$$

where $P_{x,c,t}$ is prevalence for breastfeeding category x in country c and year t; $SDI_{c,t}$ is value of the Sociodemographic Index for country c and year t; α_c, $\gamma_{R[c]}$, and $\omega_{\mathrm{SR[c]}}$ are country, region, and super-region random intercepts, respectively.

We then followed this with a spatio-temporal regression that uses the residuals of the predictions from the linear regression to perform a locally-weighted regression that provides a greater weighting factor to those nearer in space and time. The predicted residuals from this step are then added to those created in the linear regression step.

Finally, we run a Gaussian process regression that incorporates the variance of the input data as well as the variance of the model predictions. It uses predictions from the spatio-temporal regression as the mean function and generates draws from a multinomial distribution (based on the data uncertainty in the prior) to generate the final prevalence estimates and their confidence intervals.

We estimated six models to produce each of our categories: the proportion of currently breastfeeding infants 0-5 months of age, the ratio of infants exclusively breastfed to breastfed infants 0-5 months of age, the ratio of infants predominantly breastfed to breastfed infants 0-5 months of age, the ratio of infants partially breastfed to breastfed infants 0-5 months of age, the proportion of currently breastfeeding infants 6-11 months of age, and the proportion of currently breastfeeding infants 12-23 months of age. We convert the ratios of exclusive, predominant, and partial breastfeeding to the total category prevalence proportions by multiplying each ratio by the estimates of any

breastfeeding among infants aged 0-5 months. This ensures that these categories sum correctly to the "any breastfeeding 0-5 months" envelope. We calculate the proportion of infants receiving no breastmilk 0-5 months of age by subtracting the estimates of current breastfeeding from 1. We perform the same operation to estimate discontinued breastfeeding in the 6-11 months and 12-23 month categories.

Estimating Attributable Burden

Assessment of risk-outcome pairs

We included outcomes based on the strength of available evidence supporting a causal relationship. Studies evaluating the causal evidence for our risk-outcome pairs came primarily from articles found in a review published by the World Health Organization.[1] Non-exclusive breastfeeding was paired with diarrhea and lower-respiratory infection as diseases outcomes. Discontinued breastfeeding was paired with diarrhea as an outcome.

Theoretical minimum-risk exposure level

For non-exclusive breastfeeding, those children that received no source of nourishment other than breastmilk ("exclusively breastfed") were considered to be at the lowest risk of any of the disease outcomes. For discontinued breastfeeding, we assumed that children aged 6 to 23 months who received any breastmilk as a source of nourishment to be at the lowest risk of disease outcome.

Relative Risks

We estimate relative risks for both non-exclusive and discontinued breastfeeding in a meta-analysis using relative risks from studies compiled in a published review by the World Health Organization.[38]

**Sub-optimal breastfeeding category**	**Relative risk**
None	3.605<br>(2.716 to 4.703)
Partial	2.633<br>(1.942 to 3.481)
Predominant	2.346<br>(1.667 to 3.234)
Discontinued	1.313<br>(1.111 to 1.549)
Exclusive	1.0<br>(1.0 to 1.0)

References

1 GBD 2017 Causes of Death Collaborators. Global, regional, and national age-sex-specific mortality for 282 causes of death in 195 countries and territories, 1980-2017: a systematic analysis for the Global Burden of Disease Study 2017. *The Lancet* 2018; **392**: 1736–88.

2 Foreman KJ, Lozano R, Lopez AD, Murray CJ. Modeling causes of death: an integrated approach using CODEm. *Popul Health Metr* 2012; **10**: 1.

3 GBD 2016 Mortality Collaborators. Global, regional, and national under-5 mortality, adult mortality, age-specific mortality, and life expectancy, 1970-2016: a systematic analysis for the Global Burden of Disease Study 2016. *Lancet Lond Engl* 2017; **390**: 1084–150.

4 Foreman KJ, Lozano R, Lopez AD, Murray CJ. Modeling causes of death: an integrated approach using CODEm. *Popul Health Metr* 2012; **10**: 1.

5 WHO. Estimates for 2000-2015. http://www.who.int/healthinfo/global_burden_disease/estimates_child_cod_2015/en/ (accessed Aug 25, 2016).

6 WHO | WHO Global Database on Child Growth and Malnutrition. WHO. http://www.who.int/nutgrowthdb/en/ (accessed July 30, 2018).

7 Wang Y, Chen H-J. Use of Percentiles and Z-Scores in Anthropometry. In: Preedy VR, ed. Handbook of Anthropometry. New York, NY: Springer New York, 2012: 29–48.

8 Uribe Á, Cecilia M, López Gaviria A, Estrada Restrepo A. Concordance between Z scores from WHO 2006 and the NCHS 1978 growth standards of children younger than five. Antioquia-Colombia. *Perspect En Nutr Humana* 2008; **10**: 177–87.

9 McDonald CM, Olofin I, Flaxman S, *et al.* The effect of multiple anthropometric deficits on child mortality: meta-analysis of individual data in 10 prospective studies from developing countries. *Am J Clin Nutr* 2013; **97**: 896–901.

10 Olofin I, McDonald CM, Ezzati M, *et al.* Associations of Suboptimal Growth with All-Cause and Cause-Specific Mortality in Children under Five Years: A Pooled Analysis of Ten Prospective Studies. *PLOS ONE* 2013; **8**: e64636.

11 Cairncross S, Hunt C, Boisson S, *et al.* Water, sanitation and hygiene for the prevention of diarrhoea. *Int J Epidemiol* 2010; **39 Suppl 1**: i193-205.

12 WHO/UNICEF Joint Monitoring Programme (JMP) for Water Supply and Sanitation. Improved and unimporved water sources and sanitation facilities. http://www.wssinfo.org/definitions-methods/watsan-categories/ (accessed Nov 18, 2016).

13 Wolf J, Prüss-Ustün A, Cumming O, *et al.* Assessing the impact of drinking water and sanitation on diarrhoeal disease in low- and middle-income settings: systematic review and meta-regression. *Trop Med Int Health TM IH* 2014; **19**: 928–42.

14 Bain R, Cronk R, Wright J, Yang H, Slaymaker T, Bartram J. Fecal contamination of drinking-water in low- and middle-income countries: a systematic review and meta-analysis. *PLoS Med* 2014; **11**: e1001644.

15 The DHS Program - Quality information to plan, monitor and improve population, health, and nutrition programs. https://dhsprogram.com/ (accessed Oct 25, 2018).

16 UNICEF Multiple Indicator Cluster Survey (MICS). http://mics.unicef.org/ (accessed Oct 25, 2018).

17 Centers for Disease Control and Prevention. Reproductive Health Surveys. 2018; published online April 11. https://www.cdc.gov/reproductivehealth/global/tools/surveys.htm (accessed Oct 25, 2018).

18 World Bank. Living Standards Measurement Study (LSMS). http://surveys.worldbank.org/lsms (accessed Oct 25, 2018).

19 Munos MK, Walker CLF, Black RE. The effect of oral rehydration solution and recommended home fluids on diarrhoea mortality. *Int J Epidemiol* 2010; **39 Suppl 1**: i75-87.

20 Measure DHS: Demographic and Health Surveys. http://www.measuredhs.com (accessed Aug 11, 2015).

21 UNICEF Stat. Monit. Multiple Indicator Cluster Survey (MICS). http://www.unicef.org/statistics/index_24302.html (accessed Aug 11, 2015).

22 Cent. Dis. Control Prev. Reproductive Health Surveys (RHS). http://www.cdc.gov/reproductivehealth/Global/surveys.htm (accessed Aug 11, 2015).

23 World Bank. Living Standard Measruement Studies (LSMS). http://go.worldbank.org/UK1ETMHBN0 (accessed Aug 11, 2015).

24 WHO Multi-Ctry. Stud. Data Arch. World Health Survey (WHS). http://apps.who.int/healthinfo/systems/surveydata/index.php/catalog/whs/about (accessed Aug 11, 2015).

25 IHME GHDx. Global Helath Data Exchange. http://ghdx.healthdata.org/ (accessed Aug 11, 2015).

26 WHO | WHO/UNICEF Joint Reporting Process. WHO. http://www.who.int/immunization/monitoring_surveillance/routine/reporting/reporting/en/ (accessed Aug 17, 2015).

27 Prospective Studies Collaboration. Age-specific relevance of usual blood pressure to vascular mortality: a meta-analysis of individual data for one million adults in 61 prospective studies. *The Lancet* 2002; **360**: 1903–13.

28 Ng M, Freeman MK, Fleming TD, *et al.* Smoking Prevalence and Cigarette Consumption in 187 Countries, 1980-2012. *JAMA* 2014; **311**: 183.

29 Ng M, Fleming T, Robinson M, *et al.* Global, regional, and national prevalence of overweight and obesity in children and adults during 1980–2013: a systematic analysis for the Global Burden of Disease Study 2013. *The Lancet* 2014; **384**: 766–81.

30 Murray CJ, Shengelia B, Gupta N, Moussavi S, Tandon A, Thieren M. Validity of reported vaccination coverage in 45 countries. *The Lancet* 2003; **362**: 1022–1027.

31 Haddad S, Bicaba A, Feletto M, Fournier P, Zunzunegui MV. Heterogeneity in the validity of administrative-based estimates of immunization coverage across health districts in Burkina Faso: implications for measurement, monitoring and planning. *Health Policy Plan* 2010; **25**: 393–405.

32 Wang H, Liddell CA, Coates MM, *et al.* Global, regional, and national levels of neonatal, infant, and under-5 mortality during 1990–2013: a systematic analysis for the Global Burden of Disease Study 2013. *The Lancet* 13; **384**: 957–79.

33 Barber RM, Fullman N, Sorensen RJD, *et al.* Healthcare Access and Quality Index based on mortality from causes amenable to personal health care in 195 countries and territories, 1990?2015: a novel analysis from the Global Burden of Disease Study 2015. *The Lancet* 2017; published online May. DOI:10.1016/S0140-6736(17)30818-8.

34 Foreman KJ, Lozano R, Lopez AD, Murray CJ. Modeling causes of death: an integrated approach using CODEm. *Popul Health Metr* 2012; **10**: 1.

35 WHO | Data, statistics and graphics. WHO. http://www.who.int/immunization/monitoring_surveillance/data/en/ (accessed June 12, 2017).

36 Lamberti LM, Ashraf S, Walker CLF, Black RE. A Systematic Review of the Effect of Rotavirus Vaccination on Diarrhea Outcomes Among Children Younger Than 5 Years. *Pediatr Infect Dis J* 2016; **35**: 992–8.

37 Katz J, Lee AC, Kozuki N, *et al.* Mortality risk in preterm and small-for-gestational-age infants in low-income and middle-income countries: a pooled country analysis. *Lancet Lond Engl* 2013; **382**: 417–25.

38 Horta BL, Victora CG. Short-term effects of breastfeeding: a systematic review on the benefits of breastfeeding on diarrhoea and pneumonia mortality. *World Health Organ* 2013.

Supplementary results

Supplementary results table 1. The deaths, mortality rate, percent and absolute change in mortality rate between 1990 and 2017, the deaths per 100,000 episodes, and the attributable fractions due to aggregate risk factor categories are shown for each GBD region and country.

Location Name	Deaths (95% UI)	Mortality per 100,000 (95% UI)	Ratio of observed mortality rate to expected	Percent change in mortality rate 1990-2017 (95% UI)	Absolute difference in mortality rate 1990-2017 (95% UI)	Deaths per 100,000 episodes (95% UI)	Nutrition-associated risks attributable fraction (95% UI)	Low rotavirus vaccine attributable fraction (95% UI)	Unsafe WASH attributable fraction (95% UI)	Low ORS coverage attributable fraction (95% UI)	Total risk attributable fraction (95% UI)
Global	533,768 (477,162-593,145)	78.4 (70.1-87.1)	1.4	-69.6% (-74.6 to -63.1%)	-179.5 (-149.2 to -207.1)	48.2 (44.5-51.5)	88.5% (81.3 to 92.4%)	22.0% (16.7 to 27.9%)	94.0% (85.9 to 98.1%)	57.7% (39.9 to 70.8%)	99.4% (98.7 to 99.8%)
Southeast Asia, East Asia, and Oceania	22,105 (19,881-24,679)	15.6 (14.0-17.4)	2.1	-83.1% (-85.5 to -79.9%)	-76.7 (-65.6 to -88.6)	10.2 (9.1-11.3)	88.1% (79.8 to 92.1%)	29.5% (20.7 to 39.8%)	85.7% (66.4 to 95.6%)	54.8% (37.1 to 68.1%)	98.6% (96.8 to 99.6%)
East Asia	2,645 (2,309-3,198)	3.1 (2.7-3.8)	0.9	-95.1% (-95.9 to -94.1%)	-60.5 (-52.3 to -70.9)	2.4 (2.2-2.6)	77.3% (61.4 to 86.2%)	9.9% (4.5 to 19.7%)	77.4% (58.2 to 91.2%)	55.5% (37.8 to 68.7%)	95.1% (89.8 to 98.2%)
China	2,343 (2,046-2,699)	2.9 (2.5-3.4)	0.8	-95.5% (-96.4 to -94.6%)	-62.1 (-53.7 to -72.6)	2.3 (2.0-2.5)	76.5% (60.4 to 85.5%)	7.8% (3.2 to 16.7%)	78.3% (59.0 to 91.8%)	55.5% (37.8 to 68.8%)	95.0% (89.6 to 98.2%)
North Korea	254 (137-887)	18.9 (10.2-66.1)	0.9	-36.9% (-66.7 to 15.2%)	-11.0 (-5.4 to -0.8)	5.6 (3.8-15.5)	86.2% (74.2 to 92.7%)	25.6% (16.2 to 36.4%)	70.1% (51.4 to 86.0%)	56.0% (37.6 to 70.7%)	96.3% (92.8 to 98.5%)
Taiwan	6 (4-8)	0.6 (0.4-0.8)	2.5	-66.9% (-77.5 to -53.4%)	-1.2 (-1.0 to -1.4)	0.3 (0.3-0.3)	69.4% (52.2 to 80.3%)	22.1% (13.9 to 31.6%)	37.0% (18.0 to 61.9%)	55.1% (36.3 to 68.9%)	83.5% (73.8 to 91.0%)
Southeast Asia	18,009 (15,924-20,389)	32.4 (28.6-36.7)	1.6	-79.3% (-83.3 to -73.7%)	-123.9 (-96.8 to -152.3)	18.0 (17.0-19.1)	89.5% (82.3 to 93.0%)	33.3% (23.8 to 44.5%)	86.2% (66.0 to 95.9%)	54.1% (36.4 to 67.5%)	99.0% (97.4 to 99.7%)
Cambodia	300 (195-451)	16.8 (10.9-25.2)	0.9	-92.9% (-95.9 to -87.7%)	-220.7 (-160.4 to -299.0)	8.7 (6.9-11.1)	85.4% (75.3 to 91.8%)	36.9% (31.8 to 41.6%)	89.7% (74.6 to 97.1%)	58.1% (40.0 to 71.8%)	99.0% (97.4 to 99.8%)
Indonesia	10,568 (9,121-12,150)	50.3 (43.4-57.9)	1.8	-79.2% (-83.9 to -71.9%)	-191.3 (-139.5 to -252.7)	22.2 (21.0-23.2)	90.8% (85.1 to 93.7%)	38.3% (24.6 to 53.6%)	84.6% (61.4 to 95.6%)	55.4% (37.8 to 68.6%)	99.2% (97.7 to 99.8%)

Location Name	Deaths (95% UI)	Mortality per 100,000 (95% UI)	Ratio of observed mortality rate to expected	Percent change in mortality rate 1990-2017 (95% UI)	Absolute difference in mortality rate 1990-2017 (95% UI)	Deaths per 100,000 episodes (95% UI)	Nutrition-associated risks attributable fraction (95% UI)	Low rotavirus vaccine attributable fraction (95% UI)	Unsafe WASH attributable fraction (95% UI)	Low ORS coverage attributable fraction (95% UI)	Total risk attributable fraction (95% UI)
Laos	949 (510-1,513)	117.9 (63.3-187.9)	2.5	-78.9% (-87.8 to -65.4%)	-441.8 (-247.4 to -648.2)	66.6 (41.2-93.1)	86.3% (75.3 to 92.3%)	41.8% (35.6 to 47.9%)	93.4% (83.2 to 98.2%)	54.6% (35.8 to 68.9%)	99.3% (98.1 to 99.8%)
Malaysia	54 (32-82)	2.1 (1.2-3.1)	1.8	-80.0% (-88.9 to -64.5%)	-8.3 (-6.0 to -11.6)	1.0 (0.8-1.1)	85.7% (75.0 to 92.0%)	16.8% (10.8 to 24.4%)	55.4% (24.5 to 80.9%)	55.5% (36.7 to 69.9%)	95.8% (91.6 to 98.4%)
Maldives	1 (0-1)	1.6 (1.0-2.5)	1	-95.8% (-97.9 to -91.1%)	-37.0 (-19.8 to -66.1)	1.5 (1.3-1.7)	85.5% (73.4 to 92.2%)	30.6% (20.0 to 43.6%)	87.8% (70.6 to 96.4%)	49.9% (31.2 to 65.5%)	98.7% (96.7 to 99.7%)
Mauritius	4 (3-5)	6.0 (4.4-7.8)	1.4	-80.4% (-85.9 to -72.2%)	-24.6 (-21.0 to -28.5)	3.1 (2.9-3.2)	85.2% (74.1 to 90.9%)	10.5% (5.0 to 18.9%)	28.1% (13.8 to 48.1%)	55.7% (37.0 to 69.8%)	93.0% (87.0 to 96.0%)
Myanmar	2,003 (1,330-2,921)	45.4 (30.2-66.3)	2.3	-83.7% (-90.7 to -71.2%)	-232.7 (-140.6 to -354.5)	25.3 (20.0-31.3)	86.3% (74.0 to 92.6%)	38.1% (32.9 to 43.2%)	93.0% (81.7 to 98.1%)	49.2% (31.2 to 64.1%)	99.2% (98.0 to 99.8%)
Philippines	3,763 (2,638-5,239)	31.5 (22.1-43.9)	1.2	-64.8% (-76.8 to -49.4%)	-57.9 (-53.5 to -60.9)	23.2 (19.9-26.8)	89.0% (78.4 to 94.1%)	16.6% (10.3 to 25.0%)	85.5% (64.6 to 95.6%)	53.0% (34.8 to 67.3%)	98.5% (96.2 to 99.6%)
Sri Lanka	12 (7-19)	0.7 (0.5-1.2)	0.3	-96.6% (-98.2 to -94.1%)	-21.2 (-14.6 to -29.3)	0.6 (0.5-0.7)	85.6% (75.0 to 92.0%)	19.3% (12.4 to 28.0%)	84.5% (64.0 to 95.1%)	59.2% (41.3 to 72.5%)	98.6% (96.5 to 99.6%)
Seychelles	0 (0-0)	2.7 (1.8-3.9)	4.1	-22.5% (-55.0 to 29.2%)	-0.8 (-0.5 to -1.4)	1.8 (1.6-2.0)	81.8% (66.7 to 89.8%)	7.6% (3.1 to 15.2%)	78.2% (52.1 to 93.0%)	55.8% (36.1 to 70.5%)	96.8% (92.3 to 99.1%)
Thailand	129 (87-179)	3.8 (2.6-5.3)	1.3	-84.6% (-90.4 to -75.0%)	-21.1 (-14.2 to -30.9)	3.4 (2.8-3.9)	84.3% (70.1 to 91.5%)	43.5% (35.6 to 51.8%)	83.7% (59.8 to 95.2%)	48.3% (30.6 to 63.7%)	98.0% (94.7 to 99.5%)
Timor-Leste	75 (35-129)	42.9 (20.2-74.0)	1.8	-87.7% (-93.6 to -77.9%)	-305.2 (-154.4 to -522.4)	21.4 (13.1-28.0)	93.5% (87.3 to 96.6%)	30.3% (19.6 to 43.5%)	92.7% (81.9 to 98.0%)	42.2% (24.6 to 58.4%)	99.7% (99.0 to 99.9%)
Vietnam	128 (81-197)	1.6 (1.0-2.6)	0.4	-95.6% (-97.8 to -89.1%)	-36.3 (-17.0 to -60.4)	1.1 (0.9-1.4)	83.7% (71.5 to 91.0%)	37.5% (30.2 to 46.2%)	86.3% (66.3 to 95.9%)	56.1% (38.8 to 70.2%)	98.4% (95.7 to 99.6%)
Oceania	1,450 (961-2,127)	81.5 (54.0-119.6)	1.3	-37.3% (-60.7 to 0.0%)	-48.5 (-41.3 to -52.2)	27.4 (22.9-31.9)	88.1% (80.5 to 93.0%)	21.5% (13.9 to 31.0%)	94.8% (86.7 to 98.6%)	61.7% (44.0 to 74.5%)	99.5% (98.8 to 99.9%)
American Samoa	0 (0-0)	2.3 (1.4-3.7)	1.3	-54.2% (-76.2 to -15.5%)	-2.8 (-2.0 to -3.7)	0.9 (0.7-1.1)	78.2% (59.8 to 87.8%)	26.1% (17.0 to 37.6%)	69.1% (39.3 to 88.5%)	61.0% (43.6 to 73.8%)	94.1% (87.4 to 98.1%)
Federated States of Micronesia	0 (0-1)	3.5 (1.8-6.0)	0.3	-91.0% (-95.7 to -84.3%)	-35.8 (-25.0 to -49.1)	1.4 (0.9-1.8)	83.7% (68.7 to 92.3%)	18.8% (11.5 to 28.6%)	83.1% (62.6 to 94.6%)	60.0% (42.2 to 72.8%)	97.6% (94.1 to 99.3%)

Location Name	Deaths (95% UI)	Mortality per 100,000 (95% UI)	Ratio of observed mortality rate to expected	Percent change in mortality rate 1990-2017 (95% UI)	Absolute difference in mortality rate 1990-2017 (95% UI)	Deaths per 100,000 episodes (95% UI)	Nutrition-associated risks attributable fraction (95% UI)	Low rotavirus vaccine attributable fraction (95% UI)	Unsafe WASH attributable fraction (95% UI)	Low ORS coverage attributable fraction (95% UI)	Total risk attributable fraction (95% UI)
Fiji	22 (14-33)	23.4 (14.8-35.5)	2.9	-17.0% (-54.3 to 49.8%)	-4.8 (-4.0 to -5.2)	8.2 (6.6-9.6)	82.6% (66.1 to 90.7%)	8.4% (3.9 to 15.7%)	76.5% (51.9 to 91.8%)	59.1% (40.7 to 72.5%)	96.5% (92.0 to 98.9%)
Guam	0 (0-0)	0.9 (0.6-1.3)	2	-33.5% (-60.1 to 6.8%)	-0.4 (-0.3 to -0.6)	0.2 (0.2-0.2)	76.2% (57.9 to 86.6%)	26.6% (17.6 to 37.8%)	59.8% (27.1 to 83.7%)	59.7% (41.7 to 73.2%)	91.7% (83.0 to 97.0%)
Kiribati	7 (4-11)	44.1 (27.2-65.1)	0.4	-81.7% (-89.5 to -69.5%)	-196.3 (-145.2 to -257.6)	15.0 (11.9-17.2)	88.7% (78.2 to 94.1%)	14.5% (8.2 to 23.0%)	92.1% (80.4 to 97.8%)	59.0% (39.6 to 72.7%)	99.3% (98.1 to 99.8%)
Marshall Islands	0 (0-1)	5.2 (3.2-8.1)	1.3	-74.6% (-85.3 to -56.4%)	-15.3 (-9.9 to -22.1)	2.2 (1.7-2.7)	83.6% (68.7 to 91.3%)	18.9% (11.5 to 28.5%)	85.4% (65.8 to 95.4%)	61.9% (44.1 to 74.9%)	97.9% (94.9 to 99.4%)
Northern Mariana Islands	0 (0-0)	0.6 (0.4-0.9)	0.8	-35.5% (-62.2 to 13.6%)	-0.4 (-0.2 to -0.6)	0.2 (0.2-0.2)	74.6% (55.8 to 85.5%)	25.5% (17.0 to 36.9%)	62.9% (31.7 to 85.0%)	59.5% (41.0 to 72.8%)	92.1% (83.6 to 97.3%)
Papua New Guinea	1,312 (848-1,955)	96.8 (62.6-144.2)	1.9	-42.4% (-65.2 to -4.9%)	-71.3 (-56.0 to -84.8)	32.2 (26.3-37.7)	88.3% (80.9 to 93.2%)	21.7% (13.9 to 31.3%)	95.2% (87.4 to 98.8%)	61.8% (44.0 to 74.7%)	99.6% (98.9 to 99.9%)
Samoa	1 (0-1)	2.1 (1.1-3.6)	0.5	-65.7% (-80.9 to -37.7%)	-4.1 (-2.3 to -6.7)	0.8 (0.5-1.0)	75.2% (55.4 to 86.5%)	26.1% (17.0 to 37.6%)	61.1% (31.6 to 83.6%)	59.3% (40.9 to 72.4%)	91.3% (82.5 to 96.8%)
Solomon Islands	17 (10-27)	18.4 (10.7-29.0)	1	-72.4% (-84.3 to -52.5%)	-48.2 (-28.7 to -71.8)	6.7 (4.9-8.2)	82.6% (67.2 to 91.1%)	25.5% (16.6 to 37.4%)	91.8% (80.6 to 97.5%)	60.0% (41.8 to 73.2%)	98.7% (96.7 to 99.6%)
Tonga	0 (0-0)	2.4 (1.6-3.5)	0.8	-75.6% (-85.6 to -59.2%)	-7.6 (-5.1 to -10.7)	1.0 (0.8-1.1)	81.7% (65.9 to 89.7%)	25.0% (16.6 to 36.3%)	78.1% (51.4 to 92.5%)	58.9% (40.7 to 72.2%)	96.5% (91.5 to 99.0%)
Vanuatu	9 (5-14)	22.3 (12.6-35.7)	1.3	-58.1% (-76.8 to -25.4%)	-30.9 (-18.5 to -46.7)	8.8 (6.3-11.0)	84.5% (71.4 to 91.9%)	25.7% (16.8 to 36.9%)	94.2% (85.4 to 98.4%)	61.5% (43.7 to 74.3%)	99.2% (97.9 to 99.8%)
Central Europe, Eastern Europe, and Central Asia	2,395 (1,907-3,032)	8.6 (6.8-10.8)	0.8	-78.6% (-83.2 to -72.8%)	-31.4 (-30.5 to -31.8)	6.5 (6.5-6.6)	85.3% (75.4 to 90.8%)	8.0% (5.0 to 12.2%)	82.5% (66.8 to 93.3%)	51.1% (33.7 to 65.0%)	97.7% (95.4 to 99.1%)
Central Asia	2,187 (1,703-2,812)	22.8 (17.8-29.3)	0.6	-82.2% (-86.4 to -76.8%)	-105.1 (-100.5 to -108.4)	24.3 (23.2-25.4)	86.2% (76.8 to 91.5%)	6.5% (4.0 to 10.3%)	85.0% (69.3 to 94.8%)	50.5% (33.1 to 64.6%)	98.3% (96.3 to 99.4%)
Armenia	6 (4-7)	2.7 (2.0-3.6)	0.2	-96.9% (-97.7 to -95.6%)	-84.0 (-71.3 to -97.9)	2.2 (2.2-2.2)	71.2% (54.0 to 82.0%)	2.0% (0.6 to 4.9%)	53.6% (39.8 to 71.0%)	52.1% (32.2 to 67.3%)	88.7% (81.2 to 94.0%)

Location Name	Deaths (95% UI)	Mortality per 100,000 (95% UI)	Ratio of observed mortality rate to expected	Percent change in mortality rate 1990-2017 (95% UI)	Absolute difference in mortality rate 1990-2017 (95% UI)	Deaths per 100,000 episodes (95% UI)	Nutrition-associated risks attributable fraction (95% UI)	Low rotavirus vaccine attributable fraction (95% UI)	Unsafe WASH attributable fraction (95% UI)	Low ORS coverage attributable fraction (95% UI)	Total risk attributable fraction (95% UI)
Azerbaijan	161 (103-242)	19.0 (12.1-28.5)	0.3	-87.8% (-92.6 to -80.2%)	-136.2 (-113.5 to -160.7)	16.8 (13.7-19.8)	79.3% (64.4 to 88.1%)	10.2% (8.1 to 12.5%)	72.7% (54.0 to 88.6%)	61.8% (44.5 to 74.9%)	95.6% (91.5 to 98.3%)
Georgia	7 (5-9)	2.7 (1.9-3.6)	0.2	-90.6% (-93.7 to -86.6%)	-25.7 (-20.8 to -32.5)	2.3 (2.1-2.5)	73.7% (57.3 to 84.2%)	4.8% (2.2 to 8.8%)	71.3% (51.0 to 87.9%)	56.2% (37.0 to 70.4%)	93.6% (87.6 to 97.5%)
Kazakhstan	64 (43-88)	3.4 (2.3-4.6)	0.2	-95.0% (-96.7 to -92.9%)	-64.8 (-55.1 to -75.5)	4.0 (3.4-4.1)	75.5% (58.7 to 85.3%)	15.4% (9.2 to 23.2%)	72.9% (53.8 to 88.6%)	48.2% (29.3 to 64.2%)	94.1% (88.6 to 97.8%)
Kyrgyzstan	118 (92-147)	15.2 (11.9-19.0)	0.2	-85.2% (-89.2 to -80.3%)	-87.7 (-75.0 to -102.7)	19.4 (17.6-21.2)	71.3% (53.8 to 82.4%)	12.1% (7.1 to 18.3%)	84.9% (70.9 to 94.6%)	48.0% (29.6 to 63.5%)	96.0% (91.5 to 98.6%)
Mongolia	27 (18-39)	7.0 (4.8-10.1)	0.8	-81.1% (-89.2 to -67.6%)	-30.0 (-20.7 to -41.5)	5.1 (4.2-6.2)	68.4% (51.7 to 80.8%)	35.5% (30.1 to 40.5%)	90.0% (76.0 to 97.1%)	54.8% (36.7 to 69.0%)	97.5% (93.7 to 99.3%)
Tajikistan	1,590 (1,108-2,208)	127.5 (88.8-177.1)	0.6	-68.1% (-78.2 to -54.3%)	-272.2 (-245.8 to -292.0)	79.2 (66.4-93.1)	90.1% (83.1 to 94.4%)	4.1% (1.7 to 8.1%)	87.5% (71.9 to 96.1%)	48.5% (31.4 to 63.3%)	99.2% (98.0 to 99.8%)
Turkmenistan	58 (40-83)	10.6 (7.2-15.0)	0.2	-93.6% (-95.7 to -90.6%)	-154.8 (-133.3 to -178.0)	17.2 (14.5-19.6)	74.0% (56.8 to 84.0%)	13.0% (7.9 to 19.3%)	81.8% (62.8 to 93.5%)	54.7% (36.5 to 69.0%)	96.0% (91.2 to 98.7%)
Uzbekistan	156 (110-215)	4.5 (3.2-6.3)	0.2	-95.7% (-97.0 to -93.8%)	-100.1 (-86.2 to -114.2)	6.5 (5.8-7.0)	78.3% (63.8 to 86.9%)	1.5% (0.4 to 4.0%)	80.2% (63.0 to 92.4%)	59.2% (41.2 to 72.5%)	96.7% (93.1 to 98.8%)
Central Europe	70 (61-82)	1.2 (1.1-1.4)	0.9	-83.4% (-86.4 to -79.4%)	-6.2 (-5.6 to -7.0)	0.7 (0.7-0.8)	79.1% (65.2 to 86.6%)	28.6% (18.8 to 40.0%)	47.0% (26.6 to 68.9%)	58.7% (40.9 to 71.9%)	90.4% (84.0 to 95.0%)
Albania	2 (1-4)	1.3 (0.7-2.3)	0.4	-91.8% (-95.8 to -84.8%)	-14.6 (-11.4 to -18.6)	0.9 (0.7-1.3)	86.3% (74.3 to 93.1%)	18.8% (11.4 to 27.4%)	69.8% (44.3 to 88.2%)	56.8% (38.6 to 71.5%)	97.0% (94.0 to 99.0%)
Bosnia and Herzegovina	2 (1-3)	1.3 (0.9-1.9)	3	-75.2% (-85.7 to -56.1%)	-4.0 (-2.6 to -5.7)	1.0 (0.8-1.2)	78.5% (63.0 to 89.0%)	25.0% (15.6 to 35.7%)	47.6% (24.0 to 72.3%)	59.1% (40.3 to 73.1%)	91.4% (85.7 to 95.9%)
Bulgaria	8 (6-12)	2.6 (1.8-3.7)	2.6	-44.7% (-65.1 to -14.9%)	-2.1 (-2.0 to -2.2)	1.6 (1.5-1.8)	79.9% (64.0 to 88.6%)	26.0% (16.6 to 37.3%)	38.1% (14.0 to 66.5%)	58.7% (40.3 to 73.2%)	88.8% (80.5 to 94.7%)
Croatia	2 (1-2)	0.9 (0.7-1.2)	1.8	-43.4% (-62.5 to -11.7%)	-0.7 (-0.6 to -0.9)	0.6 (0.6-0.7)	77.1% (60.1 to 86.7%)	25.7% (16.0 to 36.9%)	34.3% (13.9 to 60.4%)	58.3% (39.2 to 73.2%)	86.5% (77.3 to 92.7%)

Location Name	Deaths (95% UI)	Mortality per 100,000 (95% UI)	Ratio of observed mortality rate to expected	Percent change in mortality rate 1990-2017 (95% UI)	Absolute difference in mortality rate 1990-2017 (95% UI)	Deaths per 100,000 episodes (95% UI)	Nutrition-associated risks attributable fraction (95% UI)	Low rotavirus vaccine attributable fraction (95% UI)	Unsafe WASH attributable fraction (95% UI)	Low ORS coverage attributable fraction (95% UI)	Total risk attributable fraction (95% UI)
Czech Republic	3 (2-5)	0.6 (0.4-0.9)	7.9	54.7% (-6.4 to 140.0%)	0.2 (0.1 to 0.3)	0.4 (0.4-0.4)	78.8% (62.7 to 87.9%)	25.9% (16.2 to 37.3%)	23.3% (8.2 to 47.6%)	58.6% (39.3 to 72.7%)	85.9% (76.7 to 91.5%)
Hungary	4 (3-5)	0.8 (0.6-1.1)	12.3	145.1% (59.6 to 252.9%)	0.5 (0.3 to 0.7)	0.4 (0.3-0.4)	76.2% (59.2 to 86.2%)	14.6% (9.1 to 21.3%)	33.5% (17.4 to 56.9%)	58.5% (40.5 to 73.3%)	86.5% (78.0 to 92.2%)
Macedonia	3 (2-5)	2.7 (1.8-4.1)	0.1	-97.2% (-98.3 to -95.5%)	-95.1 (-71.3 to -120.3)	1.8 (1.5-2.0)	78.1% (61.1 to 87.8%)	25.9% (16.2 to 37.7%)	44.4% (22.8 to 69.0%)	53.1% (33.3 to 68.6%)	89.3% (81.5 to 94.7%)
Montenegro	0 (0-0)	0.4 (0.3-0.7)	0.6	-78.9% (-89.6 to -58.7%)	-1.7 (-1.1 to -2.5)	0.5 (0.4-0.6)	79.8% (63.6 to 89.3%)	26.1% (16.3 to 37.5%)	56.1% (28.7 to 80.4%)	61.2% (42.9 to 74.6%)	92.7% (86.5 to 97.1%)
Poland	6 (5-8)	0.3 (0.3-0.4)	1	-88.2% (-92.1 to -82.8%)	-2.5 (-2.0 to -3.0)	0.2 (0.2-0.2)	72.6% (57.2 to 82.8%)	37.5% (23.9 to 53.7%)	31.8% (13.1 to 57.0%)	59.0% (39.6 to 73.4%)	85.6% (77.1 to 91.9%)
Romania	32 (24-42)	3.4 (2.5-4.4)	1	-78.7% (-84.9 to -69.9%)	-12.4 (-10.6 to -14.5)	1.7 (1.6-1.7)	80.6% (65.7 to 88.6%)	31.4% (21.1 to 44.6%)	59.1% (37.8 to 79.5%)	59.0% (39.5 to 73.6%)	93.3% (87.6 to 97.0%)
Serbia	3 (2-5)	0.7 (0.5-1.0)	1.3	-67.2% (-80.9 to -44.2%)	-1.4 (-1.0 to -2.0)	0.5 (0.5-0.6)	80.2% (64.9 to 89.1%)	25.8% (16.4 to 37.1%)	41.2% (16.6 to 67.6%)	62.0% (44.3 to 75.0%)	90.8% (83.6 to 95.4%)
Slovakia	4 (3-6)	1.4 (0.9-2.1)	6.3	0.2% (-38.8 to 74.2%)	0.0 (-0.1 to 0.2)	0.8 (0.7-0.9)	75.3% (57.3 to 85.9%)	25.6% (15.9 to 37.0%)	26.6% (10.3 to 51.1%)	58.6% (39.0 to 73.3%)	84.4% (74.9 to 91.0%)
Slovenia	0 (0-0)	0.2 (0.2-0.3)	1.4	-64.7% (-77.4 to -48.1%)	-0.5 (-0.4 to -0.5)	0.1 (0.1-0.2)	73.0% (55.5 to 83.7%)	25.5% (16.2 to 36.6%)	24.5% (10.5 to 46.0%)	58.6% (39.1 to 73.2%)	83.0% (73.7 to 89.5%)
Eastern Europe	138 (122-154)	1.1 (1.0-1.2)	0.4	-87.3% (-88.8 to -85.8%)	-7.4 (-7.0 to -7.9)	0.8 (0.7-0.8)	74.5% (60.7 to 81.8%)	31.4% (20.2 to 44.5%)	62.2% (43.7 to 79.4%)	56.3% (38.4 to 69.7%)	92.2% (86.3 to 96.2%)
Belarus	1 (1-2)	0.3 (0.2-0.4)	0.3	-95.2% (-96.9 to -92.7%)	-5.2 (-4.3 to -6.3)	0.3 (0.2-0.3)	71.2% (54.4 to 81.8%)	23.9% (15.1 to 34.2%)	53.5% (34.4 to 74.5%)	55.9% (36.7 to 70.7%)	88.4% (80.2 to 94.2%)
Estonia	0 (0-0)	0.4 (0.2-0.5)	0.7	-87.8% (-92.4 to -81.3%)	-2.6 (-2.1 to -3.3)	0.2 (0.2-0.2)	70.3% (51.7 to 81.9%)	2.8% (1.1 to 5.7%)	41.3% (21.3 to 65.3%)	56.8% (37.4 to 72.3%)	85.1% (75.6 to 92.3%)
Latvia	1 (0-1)	0.6 (0.4-0.9)	1.1	-75.6% (-86.4 to -60.0%)	-1.9 (-1.6 to -2.3)	0.3 (0.3-0.4)	71.3% (52.5 to 82.2%)	6.6% (2.8 to 12.5%)	49.8% (28.3 to 73.1%)	56.6% (37.1 to 72.5%)	87.0% (77.8 to 93.7%)
Lithuania	1 (1-1)	0.7 (0.5-1.0)	1.3	-71.9% (-82.0 to -58.1%)	-1.8 (-1.5 to -2.2)	0.4 (0.4-0.4)	72.0% (53.0 to 82.6%)	23.5% (14.7 to 34.0%)	50.0% (28.4 to 73.4%)	57.0% (37.0 to 71.8%)	87.4% (78.6 to 93.9%)

Location Name	Deaths (95% UI)	Mortality per 100,000 (95% UI)	Ratio of observed mortality rate to expected	Percent change in mortality rate 1990-2017 (95% UI)	Absolute difference in mortality rate 1990-2017 (95% UI)	Deaths per 100,000 episodes (95% UI)	Nutrition-associated risks attributable fraction (95% UI)	Low rotavirus vaccine attributable fraction (95% UI)	Unsafe WASH attributable fraction (95% UI)	Low ORS coverage attributable fraction (95% UI)	Total risk attributable fraction (95% UI)
Moldova	5 (3-7)	2.6 (1.8-3.7)	0.4	-88.2% (-92.1 to -83.1%)	-19.7 (-16.6 to -23.4)	2.1 (1.7-2.4)	71.5% (56.6 to 81.9%)	14.2% (8.1 to 22.1%)	85.5% (70.8 to 94.9%)	57.9% (39.8 to 72.4%)	96.7% (92.9 to 98.9%)
Russian Federation	113 (102-126)	1.2 (1.1-1.3)	0.5	-87.2% (-88.6 to -85.9%)	-8.3 (-7.7 to -8.8)	0.7 (0.6-0.8)	74.3% (60.7 to 81.6%)	33.6% (21.6 to 47.7%)	59.9% (41.1 to 78.1%)	57.2% (39.3 to 70.3%)	91.7% (85.5 to 96.0%)
Ukraine	16 (11-22)	0.7 (0.5-0.9)	0.3	-87.4% (-91.3 to -82.6%)	-4.8 (-4.1 to -5.6)	1.0 (0.9-1.0)	77.9% (61.7 to 86.9%)	25.8% (16.2 to 37.2%)	73.8% (53.9 to 89.1%)	49.5% (29.0 to 67.6%)	95.2% (90.3 to 98.2%)
High-income	706 (618-769)	1.2 (1.1-1.3)	1.5	-44.0% (-52.9 to -35.7%)	-1.0 (-0.9 to -1.1)	1.4 (1.2-1.7)	69.3% (53.4 to 79.0%)	10.6% (6.0 to 16.5%)	19.6% (11.6 to 30.0%)	57.1% (39.6 to 70.2%)	77.0% (67.2 to 83.6%)
High-income Asia Pacific	53 (45-61)	0.7 (0.6-0.8)	1.5	-43.5% (-56.0 to -28.5%)	-0.5 (-0.5 to -0.7)	0.9 (0.8-1.0)	72.0% (55.8 to 81.0%)	16.1% (10.3 to 23.2%)	21.7% (10.3 to 40.3%)	56.6% (38.5 to 69.8%)	81.1% (70.4 to 87.9%)
Brunei	0 (0-0)	1.0 (0.7-1.5)	4.1	-8.3% (-44.8 to 45.8%)	-0.1 (-0.1 to -0.1)	1.2 (1.1-1.3)	71.4% (54.7 to 82.0%)	19.5% (12.2 to 28.5%)	22.8% (10.3 to 42.3%)	56.9% (38.3 to 71.2%)	82.0% (72.2 to 88.8%)
Japan	42 (36-48)	0.8 (0.7-1.0)	2.2	8.0% (-11.3 to 29.8%)	0.1 (0.0 to 0.1)	1.1 (1.0-1.2)	73.7% (57.4 to 82.3%)	16.0% (10.1 to 23.3%)	21.8% (10.2 to 41.4%)	56.6% (38.6 to 69.7%)	82.1% (71.4 to 88.8%)
South Korea	10 (8-14)	0.5 (0.4-0.6)	1.3	-79.5% (-86.3 to -69.6%)	-1.8 (-1.4 to -2.3)	0.5 (0.5-0.5)	65.9% (46.4 to 78.4%)	15.9% (10.3 to 22.7%)	21.2% (10.0 to 39.9%)	56.7% (37.7 to 71.0%)	76.8% (65.6 to 84.6%)
Singapore	0 (0-0)	0.1 (0.1-0.1)	7.3	49.6% (-4.7 to 123.8%)	0.0 (0.0 to 0.0)	0.1 (0.1-0.1)	74.6% (61.2 to 83.8%)	26.7% (17.3 to 38.0%)	21.0% (9.9 to 39.2%)	56.7% (38.5 to 70.9%)	85.2% (76.8 to 90.8%)
Australasia	12 (8-17)	0.7 (0.5-0.9)	4.4	61.1% (-4.1 to 143.1%)	0.3 (0.1 to 0.4)	1.4 (1.3-1.5)	66.8% (47.3 to 78.6%)	4.1% (1.8 to 8.2%)	22.3% (10.3 to 40.8%)	56.9% (39.0 to 70.1%)	75.6% (63.8 to 83.8%)
Australia	10 (6-14)	0.7 (0.4-0.9)	4.9	76.9% (-0.2 to 179.4%)	0.3 (0.1 to 0.5)	1.4 (1.2-1.5)	67.2% (47.5 to 79.4%)	3.3% (1.2 to 7.7%)	20.9% (9.4 to 39.3%)	56.9% (38.9 to 70.5%)	75.5% (63.7 to 83.8%)
New Zealand	2 (2-3)	0.8 (0.6-1.0)	3	22.4% (-26.3 to 78.6%)	0.1 (0.0 to 0.2)	1.4 (1.4-1.4)	64.9% (45.1 to 77.5%)	7.3% (3.2 to 13.7%)	27.9% (13.1 to 49.3%)	56.9% (39.0 to 70.6%)	75.8% (63.4 to 85.0%)
Western Europe	129 (114-146)	0.6 (0.5-0.7)	2.2	-25.0% (-44.2 to -6.8%)	-0.2 (-0.2 to -0.3)	0.9 (0.8-1.0)	69.1% (52.5 to 79.1%)	18.5% (11.9 to 26.8%)	8.9% (5.1 to 15.0%)	56.7% (39.0 to 70.0%)	74.9% (63.5 to 81.6%)
Andorra	0 (0-0)	0.3 (0.2-0.5)	1.5	-16.6% (-53.0 to 52.0%)	-0.1 (0.0 to -0.1)	0.5 (0.4-0.6)	68.7% (45.9 to 82.8%)	27.5% (16.5 to 40.1%)	6.6% (3.5 to 11.3%)	56.7% (38.1 to 70.6%)	73.0% (60.4 to 81.1%)

Location Name	Deaths (95% UI)	Mortality per 100,000 (95% UI)	Ratio of observed mortality rate to expected	Percent change in mortality rate 1990-2017 (95% UI)	Absolute difference in mortality rate 1990-2017 (95% UI)	Deaths per 100,000 episodes (95% UI)	Nutrition-associated risks attributable fraction (95% UI)	Low rotavirus vaccine attributable fraction (95% UI)	Unsafe WASH attributable fraction (95% UI)	Low ORS coverage attributable fraction (95% UI)	Total risk attributable fraction (95% UI)
Austria	2 (2-3)	0.5 (0.4-0.7)	12.4	334.4% (169.9 to 585.4%)	0.4 (0.3 to 0.5)	0.5 (0.5-0.5)	69.3% (49.4 to 82.1%)	6.0% (2.2 to 12.5%)	11.3% (7.2 to 16.9%)	56.7% (38.2 to 71.0%)	76.4% (64.9 to 83.5%)
Belgium	5 (4-7)	0.8 (0.6-1.1)	3.5	32.6% (-21.5 to 107.5%)	0.2 (0.1 to 0.2)	1.0 (1.0-1.1)	69.5% (49.1 to 82.6%)	4.3% (1.7 to 9.1%)	7.4% (4.1 to 12.4%)	56.7% (38.8 to 70.4%)	73.6% (60.4 to 81.5%)
Cyprus	0 (0-0)	0.3 (0.2-0.5)	2	-61.2% (-79.9 to -18.5%)	-0.5 (-0.2 to -0.8)	0.4 (0.4-0.5)	70.5% (51.8 to 83.8%)	26.7% (16.3 to 38.5%)	9.6% (4.8 to 17.6%)	56.8% (38.0 to 70.6%)	79.7% (70.2 to 86.1%)
Denmark	3 (2-4)	0.9 (0.7-1.3)	2.7	17.2% (-27.4 to 79.8%)	0.1 (0.1 to 0.2)	1.1 (1.1-1.2)	68.7% (51.5 to 80.7%)	46.4% (30.1 to 64.4%)	6.5% (3.5 to 11.4%)	56.7% (38.1 to 71.2%)	74.9% (64.2 to 81.8%)
Finland	1 (0-1)	0.2 (0.1-0.3)	0.8	-69.9% (-81.7 to -51.8%)	-0.4 (-0.3 to -0.5)	0.2 (0.2-0.2)	68.7% (50.3 to 81.2%)	10.6% (4.5 to 20.6%)	6.7% (3.7 to 11.3%)	56.6% (37.9 to 70.8%)	75.7% (64.3 to 82.2%)
France	28 (20-37)	0.7 (0.5-1.0)	1.2	-61.0% (-74.2 to -42.4%)	-1.1 (-1.0 to -1.5)	1.7 (1.6-1.7)	64.8% (44.8 to 77.2%)	37.7% (25.2 to 52.9%)	9.8% (5.1 to 17.8%)	56.9% (37.9 to 70.9%)	70.7% (56.7 to 79.2%)
Germany	19 (14-26)	0.5 (0.4-0.7)	2.5	-3.9% (-37.3 to 42.4%)	0.0 (0.0 to 0.0)	0.5 (0.5-0.5)	64.3% (45.2 to 77.0%)	20.5% (12.1 to 31.2%)	7.8% (4.4 to 13.4%)	56.7% (38.8 to 70.7%)	71.1% (59.7 to 79.0%)
Greece	1 (1-2)	0.2 (0.1-0.3)	4.2	30.2% (-23.7 to 125.9%)	0.1 (0.0 to 0.0)	0.4 (0.3-0.4)	74.0% (56.6 to 85.8%)	13.3% (7.6 to 20.0%)	8.7% (5.2 to 13.4%)	56.8% (38.0 to 71.5%)	80.1% (70.6 to 86.1%)
Iceland	0 (0-0)	0.6 (0.4-0.9)	4.4	49.3% (-7.1 to 139.0%)	0.2 (0.1 to 0.3)	0.9 (0.9-1.0)	69.2% (48.9 to 82.2%)	21.2% (13.5 to 30.6%)	6.4% (3.4 to 11.2%)	56.7% (38.1 to 70.9%)	73.9% (61.5 to 81.5%)
Ireland	2 (1-2)	0.5 (0.4-0.7)	8.8	102.6% (23.4 to 213.1%)	0.3 (0.2 to 0.4)	1.0 (1.0-1.1)	71.2% (52.5 to 83.4%)	15.6% (9.6 to 22.6%)	9.2% (5.7 to 14.6%)	56.6% (37.8 to 70.8%)	76.7% (66.1 to 83.5%)
Israel	9 (6-11)	1.0 (0.7-1.3)	2.2	-14.7% (-41.8 to 22.1%)	-0.2 (-0.1 to -0.3)	2.2 (2.1-2.2)	71.4% (53.2 to 82.3%)	7.7% (3.2 to 15.6%)	10.0% (5.8 to 16.8%)	56.7% (37.9 to 70.4%)	77.2% (65.9 to 83.8%)
Italy	13 (9-18)	0.5 (0.4-0.7)	3.7	50.0% (1.3 to 120.5%)	0.2 (0.1 to 0.3)	1.0 (1.0-1.1)	77.6% (61.9 to 86.8%)	18.5% (12.3 to 26.1%)	10.9% (6.2 to 18.9%)	56.7% (38.5 to 70.8%)	81.9% (72.7 to 87.2%)
Luxembourg	0 (0-0)	0.9 (0.6-1.2)	1.4	-37.8% (-67.4 to 2.7%)	-0.5 (-0.5 to -0.7)	1.2 (1.0-1.3)	69.1% (49.8 to 82.0%)	3.4% (1.3 to 7.5%)	6.6% (3.6 to 11.7%)	56.6% (38.3 to 70.7%)	74.3% (62.5 to 81.9%)
Malta	0 (0-0)	0.4 (0.2-0.6)	5	46.0% (-11.2 to 140.3%)	0.1 (0.1 to 0.2)	1.0 (0.8-1.1)	71.0% (52.7 to 83.2%)	26.9% (16.3 to 38.8%)	9.0% (5.1 to 14.8%)	56.8% (37.5 to 70.9%)	77.2% (66.7 to 84.0%)

Location Name	Deaths (95% UI)	Mortality per 100,000 (95% UI)	Ratio of observed mortality rate to expected	Percent change in mortality rate 1990-2017 (95% UI)	Absolute difference in mortality rate 1990-2017 (95% UI)	Deaths per 100,000 episodes (95% UI)	Nutrition-associated risks attributable fraction (95% UI)	Low rotavirus vaccine attributable fraction (95% UI)	Unsafe WASH attributable fraction (95% UI)	Low ORS coverage attributable fraction (95% UI)	Total risk attributable fraction (95% UI)
Netherlands	4 (3-5)	0.4 (0.3-0.6)	6	123.1% (41.0 to 242.7%)	0.2 (0.2 to 0.4)	1.0 (1.0-1.1)	71.1% (51.2 to 83.4%)	37.2% (23.6 to 53.4%)	6.3% (3.5 to 10.8%)	56.7% (38.2 to 70.4%)	75.4% (63.4 to 82.9%)
Norway	1 (1-1)	0.3 (0.3-0.4)	4.6	45.0% (4.4 to 87.2%)	0.1 (0.1 to 0.1)	0.1 (0.1-0.2)	68.3% (51.3 to 79.2%)	24.5% (15.4 to 35.3%)	6.4% (3.4 to 10.7%)	56.6% (39.1 to 69.6%)	73.8% (62.1 to 81.3%)
Portugal	4 (3-6)	1.0 (0.7-1.4)	1.4	-71.7% (-82.0 to -56.6%)	-2.5 (-1.9 to -3.5)	2.2 (2.1-2.4)	74.0% (56.7 to 85.1%)	13.2% (8.5 to 19.1%)	22.1% (13.1 to 35.8%)	56.8% (38.2 to 70.9%)	82.7% (73.7 to 88.1%)
Spain	11 (8-15)	0.5 (0.4-0.7)	2.4	-32.1% (-55.6 to 2.4%)	-0.3 (-0.2 to -0.3)	1.1 (1.1-1.1)	70.3% (52.8 to 81.0%)	25.1% (17.2 to 34.8%)	8.0% (3.8 to 15.2%)	56.8% (38.7 to 70.7%)	76.7% (65.5 to 83.2%)
Sweden	3 (2-3)	0.4 (0.3-0.6)	12.1	280.4% (149.3 to 439.0%)	0.3 (0.2 to 0.4)	0.5 (0.5-0.5)	67.6% (48.0 to 79.9%)	31.9% (19.9 to 46.3%)	7.4% (4.0 to 12.5%)	56.6% (37.8 to 70.5%)	72.5% (60.1 to 80.2%)
Switzerland	4 (3-6)	1.0 (0.7-1.4)	1	-41.4% (-62.5 to -12.2%)	-0.7 (-0.6 to -1.1)	1.2 (1.1-1.2)	70.6% (56.7 to 81.3%)	27.8% (18.0 to 39.7%)	6.9% (4.0 to 11.7%)	56.6% (38.3 to 70.8%)	80.1% (72.2 to 85.0%)
United Kingdom	19 (17-21)	0.5 (0.4-0.5)	8.7	115.1% (65.7 to 159.6%)	0.3 (0.2 to 0.3)	0.7 (0.6-0.8)	70.1% (53.1 to 80.6%)	1.2% (0.4 to 2.8%)	6.3% (2.9 to 12.0%)	56.8% (38.9 to 69.9%)	74.5% (62.4 to 82.1%)
England	16 (14-17)	0.5 (0.4-0.5)	7.8	100.1% (54.1 to 140.1%)	0.2 (0.2 to 0.2)	0.6 (0.5-0.7)	69.3% (52.2 to 79.7%)	0.7% (0.2 to 1.6%)	6.1% (2.8 to 11.7%)	56.8% (39.2 to 69.8%)	73.8% (61.5 to 81.4%)
Northern Ireland	1 (1-1)	0.6 (0.4-1.0)	12.4	189.4% (67.2 to 360.3%)	0.4 (0.2 to 0.7)	1.0 (0.9-1.2)	73.1% (54.1 to 85.4%)	2.6% (0.6 to 7.8%)	6.6% (3.0 to 13.1%)	56.9% (39.1 to 70.4%)	77.4% (66.2 to 84.9%)
Scotland	2 (1-3)	0.6 (0.4-1.0)	14.8	214.1% (69.0 to 461.0%)	0.4 (0.2 to 0.7)	1.0 (0.8-1.2)	73.9% (54.7 to 86.1%)	3.1% (0.7 to 9.0%)	7.4% (3.3 to 14.9%)	56.9% (38.3 to 70.4%)	77.9% (66.5 to 84.9%)
Wales	1 (1-1)	0.6 (0.4-0.8)	23.4	246.7% (111.8 to 477.9%)	0.4 (0.3 to 0.6)	0.9 (0.8-0.9)	74.3% (55.6 to 86.4%)	3.5% (0.8 to 11.0%)	7.4% (3.3 to 15.1%)	56.9% (38.3 to 70.7%)	78.2% (66.8 to 85.4%)
Southern Latin America	141 (114-170)	2.8 (2.2-3.3)	0.7	-83.4% (-87.1 to -79.0%)	-13.9 (-12.6 to -15.3)	0.9 (0.9-0.9)	76.4% (61.2 to 85.2%)	10.1% (5.2 to 16.7%)	49.6% (26.5 to 74.4%)	57.8% (40.2 to 71.6%)	89.6% (82.3 to 95.2%)
Argentina	124 (97-154)	3.4 (2.6-4.2)	0.7	-83.6% (-87.8 to -78.4%)	-17.2 (-15.2 to -19.5)	1.0 (1.0-1.1)	77.3% (62.2 to 86.1%)	8.5% (4.1 to 14.8%)	51.0% (27.3 to 76.2%)	57.9% (40.0 to 71.8%)	90.4% (83.0 to 95.7%)
Chile	9 (6-13)	0.8 (0.5-1.1)	0.6	-89.4% (-93.0 to -84.4%)	-6.5 (-5.6 to -7.5)	0.3 (0.3-0.3)	67.5% (50.5 to 79.0%)	26.5% (17.2 to 37.8%)	39.8% (23.5 to 60.9%)	57.6% (39.1 to 71.6%)	82.7% (73.6 to 89.9%)

Location Name	Deaths (95% UI)	Mortality per 100,000 (95% UI)	Ratio of observed mortality rate to expected	Percent change in mortality rate 1990-2017 (95% UI)	Absolute difference in mortality rate 1990-2017 (95% UI)	Deaths per 100,000 episodes (95% UI)	Nutrition-associated risks attributable fraction (95% UI)	Low rotavirus vaccine attributable fraction (95% UI)	Unsafe WASH attributable fraction (95% UI)	Low ORS coverage attributable fraction (95% UI)	Total risk attributable fraction (95% UI)
Uruguay	8 (6-11)	3.3 (2.4-4.5)	0.8	-79.7% (-85.8 to -71.2%)	-13.1 (-11.0 to -15.2)	1.9 (1.8-1.9)	72.3% (55.6 to 82.7%)	23.9% (15.3 to 34.6%)	40.1% (18.1 to 67.0%)	57.1% (38.1 to 71.3%)	86.2% (77.3 to 92.9%)
High-income North America	371 (314-410)	1.7 (1.5-1.9)	6	125.3% (76.4 to 164.1%)	1.0 (0.8 to 1.0)	3.3 (2.7-3.8)	66.9% (50.4 to 77.7%)	7.9% (3.8 to 13.6%)	11.6% (7.1 to 17.9%)	56.9% (39.1 to 70.2%)	72.4% (61.5 to 79.8%)
Canada	17 (12-24)	0.9 (0.6-1.2)	18.2	616.2% (355.5 to 993.9%)	0.7 (0.5 to 1.0)	1.3 (1.2-1.3)	72.1% (55.7 to 82.4%)	9.3% (5.3 to 14.3%)	9.6% (5.7 to 15.2%)	56.7% (37.5 to 70.3%)	78.2% (68.7 to 84.2%)
Greenland	0 (0-0)	5.1 (3.3-8.0)	3.9	40.1% (-30.3 to 189.4%)	1.5 (1.2 to 1.9)	7.3 (6.5-8.3)	70.0% (50.5 to 81.6%)	16.5% (9.7 to 24.1%)	19.8% (12.7 to 30.0%)	57.3% (38.0 to 71.1%)	77.2% (66.2 to 84.3%)
United States	353 (298-392)	1.8 (1.5-2.0)	5.9	118.8% (69.5 to 157.8%)	1.0 (0.8 to 1.0)	3.6 (2.9-4.1)	66.6% (50.1 to 77.5%)	7.7% (3.7 to 13.6%)	11.7% (7.2 to 18.1%)	57.0% (39.1 to 70.2%)	72.1% (61.2 to 79.7%)
Latin America and Caribbean	9,904 (8,527-11,490)	19.5 (16.7-22.6)	0.5	-90.0% (-91.5 to -88.1%)	-175.6 (-165.7 to -185.4)	10.4 (9.8-10.9)	79.4% (65.6 to 87.3%)	8.0% (4.9 to 12.1%)	82.0% (67.5 to 92.1%)	55.7% (37.6 to 69.1%)	96.2% (92.6 to 98.5%)
Caribbean	3,513 (2,358-5,044)	89.8 (60.3-128.9)	1	-72.4% (-82.1 to -57.9%)	-236.1 (-212.3 to -262.3)	48.1 (40.2-55.6)	83.9% (70.9 to 91.2%)	6.9% (4.4 to 10.0%)	95.8% (88.9 to 98.9%)	53.7% (35.3 to 67.7%)	99.5% (98.6 to 99.9%)
Antigua and Barbuda	0 (0-0)	4.7 (3.1-7.1)	1.7	-50.5% (-70.4 to -16.6%)	-4.8 (-4.1 to -5.3)	4.4 (3.7-5.2)	71.0% (52.0 to 82.0%)	18.3% (11.5 to 26.8%)	83.9% (61.9 to 95.0%)	56.3% (38.7 to 70.7%)	95.9% (89.8 to 98.8%)
The Bahamas	1 (1-1)	3.5 (2.3-5.3)	0.8	-69.5% (-81.7 to -48.7%)	-8.1 (-5.9 to -10.4)	3.1 (2.7-3.5)	72.3% (53.1 to 82.9%)	11.3% (6.1 to 18.3%)	79.3% (54.9 to 93.2%)	56.7% (37.9 to 70.8%)	94.9% (87.7 to 98.4%)
Barbados	0 (0-1)	2.3 (1.4-3.5)	0.9	-68.8% (-82.7 to -48.1%)	-5.0 (-4.1 to -6.2)	1.9 (1.5-2.4)	73.4% (55.7 to 83.3%)	19.3% (12.1 to 28.2%)	75.4% (51.6 to 91.1%)	47.6% (29.3 to 63.4%)	94.5% (88.1 to 98.2%)
Belize	5 (4-8)	14.4 (9.5-21.2)	2.3	-77.2% (-86.0 to -63.8%)	-48.7 (-39.5 to -58.5)	15.6 (13.1-18.3)	77.7% (62.8 to 85.8%)	15.7% (9.5 to 23.3%)	91.5% (78.9 to 97.5%)	53.7% (35.2 to 68.2%)	98.4% (95.8 to 99.6%)
Bermuda	0 (0-0)	0.8 (0.5-1.1)	0.5	-80.7% (-88.4 to -68.5%)	-3.3 (-2.4 to -4.3)	0.6 (0.6-0.7)	69.1% (48.8 to 81.6%)	18.2% (11.2 to 26.8%)	77.3% (51.5 to 92.3%)	56.5% (38.0 to 70.3%)	93.6% (85.1 to 98.0%)
Cuba	9 (7-13)	1.6 (1.1-2.1)	0.5	-85.0% (-90.0 to -78.4%)	-8.9 (-7.3 to -11.0)	2.0 (1.9-2.2)	73.9% (56.0 to 83.6%)	24.4% (15.5 to 35.0%)	84.5% (64.2 to 95.2%)	51.1% (32.0 to 65.6%)	96.4% (91.3 to 99.0%)

Location Name	Deaths (95% UI)	Mortality per 100,000 (95% UI)	Ratio of observed mortality rate to expected	Percent change in mortality rate 1990-2017 (95% UI)	Absolute difference in mortality rate 1990-2017 (95% UI)	Deaths per 100,000 episodes (95% UI)	Nutrition-associated risks attributable fraction (95% UI)	Low rotavirus vaccine attributable fraction (95% UI)	Unsafe WASH attributable fraction (95% UI)	Low ORS coverage attributable fraction (95% UI)	Total risk attributable fraction (95% UI)
Dominica	0 (0-1)	11.0 (6.7-16.9)	4.8	-19.2% (-51.7 to 36.6%)	-2.6 (-3.4 to -0.9)	10.4 (8.1-12.4)	74.1% (56.3 to 84.1%)	19.3% (11.9 to 28.1%)	86.2% (68.2 to 95.7%)	56.4% (37.4 to 70.2%)	96.9% (92.2 to 99.2%)
Dominican Republic	183 (116-279)	18.3 (11.7-28.0)	0.4	-92.7% (-95.6 to -88.2%)	-231.0 (-184.5 to -280.9)	8.2 (6.5-10.3)	78.7% (61.9 to 88.0%)	9.7% (5.2 to 16.4%)	91.6% (78.7 to 97.7%)	54.9% (35.5 to 69.2%)	98.4% (95.9 to 99.6%)
Grenada	0 (0-0)	2.5 (1.5-4.0)	1.4	-83.2% (-91.3 to -69.0%)	-12.2 (-8.9 to -16.1)	1.7 (1.3-2.1)	74.5% (56.2 to 84.9%)	19.1% (11.9 to 28.0%)	86.3% (68.4 to 95.6%)	56.4% (37.1 to 70.2%)	97.0% (92.7 to 99.1%)
Guyana	17 (11-25)	23.5 (15.6-34.0)	0.9	-82.4% (-89.1 to -72.7%)	-110.2 (-91.0 to -130.4)	19.9 (16.6-22.9)	84.3% (72.0 to 90.7%)	5.7% (2.1 to 12.7%)	91.8% (79.1 to 97.7%)	55.4% (36.8 to 69.5%)	99.0% (97.3 to 99.8%)
Haiti	3,138 (2,033-4,608)	211.7 (137.1-310.9)	0.8	-77.6% (-86.2 to -63.7%)	-731.3 (-611.0 to -870.0)	86.0 (70.7-100.5)	84.4% (71.7 to 91.5%)	6.5% (4.2 to 9.3%)	96.3% (90.0 to 99.1%)	53.6% (34.8 to 67.8%)	99.6% (98.9 to 99.9%)
Jamaica	7 (4-12)	3.9 (2.4-6.2)	0.3	-93.8% (-96.4 to -89.7%)	-59.2 (-48.1 to -71.9)	4.7 (3.7-5.8)	71.1% (52.2 to 82.6%)	18.9% (11.9 to 27.7%)	89.4% (75.3 to 96.8%)	53.9% (35.0 to 68.4%)	97.5% (94.0 to 99.3%)
Puerto Rico	4 (3-5)	2.4 (1.7-3.2)	8.2	83.2% (22.8 to 181.7%)	1.1 (0.7 to 1.5)	2.0 (1.9-2.0)	73.5% (56.8 to 83.3%)	4.5% (1.5 to 11.0%)	75.3% (46.4 to 91.7%)	56.4% (37.8 to 71.8%)	94.4% (87.0 to 98.3%)
Saint Lucia	0 (0-1)	4.4 (2.7-6.7)	1.1	-79.8% (-87.9 to -67.4%)	-17.4 (-13.7 to -21.3)	2.9 (2.3-3.6)	75.2% (57.4 to 85.1%)	19.1% (12.0 to 27.9%)	84.6% (64.9 to 95.1%)	66.4% (49.3 to 78.1%)	96.9% (92.3 to 99.1%)
Saint Vincent and the Grenadines	1 (0-1)	9.6 (6.1-14.1)	1.2	-77.6% (-86.3 to -64.5%)	-33.3 (-27.0 to -41.6)	9.0 (7.4-10.3)	75.9% (59.6 to 85.7%)	19.4% (12.0 to 28.3%)	88.9% (73.5 to 96.7%)	56.5% (37.8 to 70.6%)	97.8% (94.6 to 99.4%)
Suriname	13 (10-18)	28.1 (21.0-36.8)	0.6	-82.5% (-87.6 to -76.0%)	-132.3 (-110.3 to -154.1)	31.0 (29.2-31.4)	80.4% (65.2 to 88.4%)	15.9% (9.8 to 23.2%)	86.6% (68.5 to 95.9%)	55.9% (38.3 to 70.1%)	97.9% (94.6 to 99.4%)
Trinidad and Tobago	6 (4-9)	6.4 (4.1-9.8)	1.4	-55.8% (-73.5 to -28.3%)	-8.1 (-7.5 to -8.2)	8.1 (6.6-9.5)	76.7% (59.4 to 86.5%)	19.2% (12.2 to 28.2%)	82.2% (60.7 to 94.1%)	56.7% (38.0 to 71.2%)	96.6% (91.4 to 99.0%)
Virgin Islands, U.S.	0 (0-0)	1.3 (0.8-2.0)	1.2	-74.3% (-85.3 to -55.7%)	-3.9 (-2.9 to -5.0)	1.0 (0.8-1.1)	70.4% (52.0 to 81.7%)	9.0% (3.9 to 16.2%)	78.2% (51.4 to 93.0%)	56.7% (37.8 to 70.6%)	94.5% (87.1 to 98.4%)
Andean Latin America	754 (560-1,003)	11.2 (8.4-15.0)	0.3	-94.5% (-96.0 to -92.4%)	-193.7 (-163.8 to -227.1)	5.6 (4.8-6.5)	70.4% (53.3 to 81.4%)	5.1% (2.7 to 8.7%)	77.0% (54.8 to 91.3%)	60.3% (42.0 to 73.0%)	93.5% (86.7 to 97.7%)

Location Name	Deaths (95% UI)	Mortality per 100,000 (95% UI)	Ratio of observed mortality rate to expected	Percent change in mortality rate 1990-2017 (95% UI)	Absolute difference in mortality rate 1990-2017 (95% UI)	Deaths per 100,000 episodes (95% UI)	Nutrition-associated risks attributable fraction (95% UI)	Low rotavirus vaccine attributable fraction (95% UI)	Unsafe WASH attributable fraction (95% UI)	Low ORS coverage attributable fraction (95% UI)	Total risk attributable fraction (95% UI)
Bolivia	238 (123-397)	16.4 (8.5-27.3)	0.4	-95.4% (-97.7 to -92.1%)	-344.7 (-257.6 to -453.3)	7.6 (5.1-9.8)	70.6% (51.9 to 82.7%)	6.8% (2.7 to 13.3%)	75.0% (50.1 to 90.8%)	59.3% (41.5 to 72.4%)	93.0% (85.3 to 97.6%)
Ecuador	149 (100-210)	9.6 (6.4-13.5)	0.3	-93.6% (-95.9 to -90.7%)	-141.4 (-125.4 to -159.6)	6.0 (4.8-7.2)	74.8% (57.4 to 85.6%)	6.0% (2.6 to 11.2%)	71.5% (45.2 to 88.8%)	59.5% (40.2 to 72.8%)	93.2% (86.0 to 97.7%)
Peru	366 (236-558)	9.9 (6.4-15.1)	0.3	-94.3% (-96.5 to -90.8%)	-165.0 (-123.3 to -208.2)	4.7 (3.4-6.4)	68.3% (50.7 to 80.0%)	1.8% (0.9 to 3.2%)	80.5% (60.4 to 93.0%)	61.3% (43.5 to 73.9%)	94.0% (87.6 to 97.9%)
Central Latin America	3,781 (3,269-4,428)	15.6 (13.5-18.3)	0.5	-89.9% (-91.4 to -88.0%)	-138.4 (-132.7 to -143.5)	7.1 (6.8-7.5)	76.3% (62.5 to 84.9%)	10.5% (6.3 to 16.3%)	74.4% (55.8 to 88.3%)	58.7% (41.1 to 71.5%)	94.6% (89.7 to 97.8%)
Colombia	298 (203-437)	7.0 (4.8-10.3)	0.6	-90.2% (-93.3 to -85.7%)	-64.7 (-56.5 to -73.5)	3.3 (2.8-3.8)	69.7% (51.6 to 81.1%)	6.7% (2.7 to 14.1%)	70.5% (44.4 to 88.7%)	53.2% (35.8 to 66.9%)	91.6% (83.0 to 96.9%)
Costa Rica	10 (7-14)	2.8 (2.0-4.0)	0.8	-83.5% (-88.7 to -76.1%)	-14.3 (-11.6 to -18.1)	1.2 (1.1-1.4)	70.0% (50.2 to 81.7%)	25.0% (16.2 to 36.4%)	49.3% (26.5 to 73.6%)	59.3% (41.3 to 72.1%)	85.5% (75.1 to 92.9%)
El Salvador	61 (34-102)	11.2 (6.2-18.7)	0.4	-95.6% (-97.7 to -92.3%)	-244.3 (-200.1 to -291.6)	4.4 (3.0-6.2)	77.0% (59.2 to 86.7%)	5.8% (2.1 to 13.0%)	83.6% (66.8 to 94.3%)	53.0% (35.3 to 66.8%)	96.5% (92.2 to 98.9%)
Guatemala	1,288 (890-1,797)	64.8 (44.8-90.4)	1.7	-85.5% (-90.2 to -79.6%)	-381.1 (-347.2 to -413.0)	18.1 (14.8-21.6)	72.2% (58.9 to 82.0%)	17.3% (10.3 to 26.5%)	89.5% (75.3 to 96.7%)	56.4% (38.1 to 69.5%)	97.2% (93.2 to 99.2%)
Honduras	271 (132-484)	23.1 (11.3-41.2)	0.6	-91.9% (-95.9 to -85.6%)	-263.6 (-224.9 to -305.0)	7.1 (4.1-10.8)	74.1% (57.7 to 84.6%)	4.9% (1.7 to 11.0%)	82.1% (65.0 to 93.7%)	50.5% (33.0 to 64.5%)	95.9% (91.3 to 98.7%)
Mexico	1,247 (1,123-1,406)	10.3 (9.3-11.7)	0.3	-92.4% (-93.3 to -91.2%)	-125.4 (-116.9 to -133.7)	6.7 (6.0-7.5)	79.7% (65.6 to 87.6%)	6.7% (3.1 to 12.2%)	59.7% (40.3 to 78.1%)	65.9% (48.7 to 77.5%)	92.0% (86.1 to 96.1%)
Nicaragua	86 (57-125)	12.7 (8.4-18.3)	0.2	-96.7% (-97.9 to -95.0%)	-374.7 (-321.0 to -429.5)	4.7 (3.7-5.6)	71.3% (53.8 to 82.3%)	0.1% (0.0 to 0.4%)	86.9% (72.0 to 95.8%)	50.8% (33.3 to 64.7%)	96.5% (92.1 to 98.9%)
Panama	77 (52-110)	21.4 (14.3-30.4)	2.5	-48.3% (-65.6 to -24.0%)	-20.0 (-17.4 to -23.5)	7.2 (5.9-8.5)	69.6% (51.2 to 81.0%)	8.0% (3.5 to 15.8%)	75.8% (55.1 to 90.9%)	52.4% (34.0 to 66.0%)	92.9% (85.6 to 97.5%)
Venezuela	443 (321-597)	15.7 (11.4-21.2)	0.6	-85.6% (-90.0 to -79.9%)	-93.8 (-82.6 to -104.5)	4.7 (4.2-5.1)	84.8% (70.4 to 91.2%)	14.9% (8.0 to 25.0%)	66.8% (40.3 to 86.5%)	57.0% (38.9 to 70.2%)	95.4% (90.0 to 98.4%)
Tropical Latin America	1,856 (1,647-2,087)	11.5 (10.2-13.0)	0.4	-94.6% (-95.4 to -93.6%)	-202.7 (-178.9 to -225.0)	8.7 (7.7-9.8)	79.8% (64.7 to 88.2%)	2.2% (0.8 to 4.9%)	73.6% (56.7 to 87.7%)	51.7% (33.6 to 66.5%)	94.5% (89.9 to 97.6%)

Location Name	Deaths (95% UI)	Mortality per 100,000 (95% UI)	Ratio of observed mortality rate to expected	Percent change in mortality rate 1990-2017 (95% UI)	Absolute difference in mortality rate 1990-2017 (95% UI)	Deaths per 100,000 episodes (95% UI)	Nutrition-associated risks attributable fraction (95% UI)	Low rotavirus vaccine attributable fraction (95% UI)	Unsafe WASH attributable fraction (95% UI)	Low ORS coverage attributable fraction (95% UI)	Total risk attributable fraction (95% UI)
Brazil	1,795 (1,586-2,025)	11.7 (10.3-13.2)	0.4	-94.6% (-95.4 to -93.6%)	-206.7 (-181.8 to -229.6)	9.3 (8.3-10.4)	79.8% (64.7 to 88.2%)	2.2% (0.8 to 5.0%)	73.5% (56.6 to 87.6%)	51.3% (33.0 to 66.4%)	94.5% (89.9 to 97.6%)
Paraguay	61 (36-101)	8.2 (4.8-13.5)	0.5	-91.3% (-95.0 to -85.2%)	-85.7 (-70.8 to -100.9)	3.0 (2.3-3.6)	78.9% (61.6 to 88.2%)	1.1% (0.4 to 3.0%)	76.0% (54.8 to 91.3%)	64.2% (43.0 to 77.5%)	95.1% (89.9 to 98.4%)
North Africa and Middle East	28,962 (23,106-35,611)	45.0 (35.9-55.3)	1.6	-80.7% (-84.8 to -75.3%)	-188.5 (-149.5 to -227.8)	17.9 (16.7-18.7)	88.0% (78.6 to 92.8%)	17.2% (11.0 to 25.1%)	87.0% (74.9 to 94.9%)	61.2% (43.6 to 73.6%)	98.3% (96.5 to 99.4%)
Afghanistan	5,911 (3,994-8,431)	100.4 (67.8-143.2)	1	-81.1% (-89.8 to -62.3%)	-430.1 (-211.1 to -674.5)	25.2 (19.7-31.5)	87.4% (77.5 to 93.1%)	27.1% (17.1 to 39.4%)	96.1% (89.7 to 99.0%)	61.1% (43.2 to 73.7%)	99.6% (99.0 to 99.9%)
Algeria	166 (79-280)	3.7 (1.8-6.3)	1	-90.1% (-94.7 to -82.0%)	-33.7 (-19.9 to -52.3)	2.0 (1.1-2.9)	82.9% (69.2 to 90.6%)	26.5% (16.6 to 38.1%)	63.1% (39.3 to 83.6%)	59.3% (41.7 to 72.1%)	94.7% (89.7 to 97.9%)
Bahrain	1 (1-1)	0.8 (0.5-1.1)	0.8	-73.7% (-85.0 to -55.5%)	-2.1 (-1.3 to -3.2)	0.4 (0.4-0.5)	77.8% (59.1 to 87.2%)	1.3% (0.3 to 4.1%)	63.2% (38.7 to 83.6%)	59.0% (41.3 to 72.0%)	92.3% (84.4 to 97.0%)
Egypt	6,457 (3,827-9,531)	54.7 (32.4-80.8)	0.7	-89.1% (-93.4 to -82.8%)	-445.1 (-378.8 to -514.7)	21.3 (15.3-26.5)	79.2% (63.4 to 88.1%)	23.8% (16.6 to 32.4%)	66.4% (48.0 to 83.7%)	58.5% (41.0 to 71.4%)	94.7% (90.0 to 97.7%)
Iran	191 (168-212)	2.8 (2.5-3.1)	0.5	-94.6% (-96.0 to -92.5%)	-49.2 (-36.1 to -62.9)	1.9 (1.6-2.1)	78.8% (63.8 to 86.1%)	48.8% (31.9 to 67.7%)	67.8% (43.6 to 86.3%)	59.1% (41.3 to 71.8%)	94.4% (88.9 to 97.8%)
Iraq	561 (401-780)	9.5 (6.8-13.1)	0.9	-84.9% (-90.6 to -75.2%)	-53.3 (-37.0 to -72.7)	6.2 (5.6-6.7)	86.8% (75.2 to 92.7%)	16.2% (9.1 to 25.5%)	75.9% (54.4 to 91.0%)	58.4% (40.3 to 71.4%)	97.7% (94.9 to 99.2%)
Jordan	35 (23-52)	3.0 (1.9-4.5)	0.7	-87.3% (-92.8 to -76.3%)	-20.4 (-12.4 to -30.0)	1.6 (1.3-1.9)	77.4% (60.9 to 87.2%)	3.5% (1.3 to 7.8%)	56.8% (32.8 to 78.8%)	62.1% (44.7 to 74.5%)	91.6% (84.7 to 96.3%)
Kuwait	3 (2-4)	1.1 (0.7-1.5)	1	-76.9% (-84.7 to -64.3%)	-3.5 (-2.7 to -4.4)	0.6 (0.5-0.6)	73.4% (55.7 to 84.0%)	26.8% (17.1 to 38.7%)	52.8% (28.5 to 76.7%)	60.7% (43.1 to 73.4%)	89.6% (81.4 to 95.3%)
Lebanon	32 (11-64)	3.6 (1.3-7.1)	2	-82.7% (-92.1 to -66.6%)	-17.2 (-7.5 to -29.3)	1.7 (0.8-2.6)	78.3% (59.7 to 88.1%)	26.9% (16.9 to 38.9%)	73.0% (45.5 to 90.4%)	56.6% (38.9 to 69.9%)	95.0% (88.9 to 98.4%)
Libya	21 (12-34)	3.3 (1.8-5.3)	0.4	-90.2% (-94.6 to -82.1%)	-30.7 (-15.5 to -50.6)	1.5 (1.0-1.9)	79.8% (61.4 to 89.3%)	6.5% (2.5 to 13.2%)	71.5% (49.0 to 88.1%)	59.1% (41.1 to 71.9%)	95.0% (89.6 to 98.2%)
Morocco	687 (372-1,144)	22.4 (12.1-37.3)	0.7	-92.2% (-96.0 to -86.5%)	-266.7 (-214.8 to -321.0)	11.3 (7.8-14.8)	81.2% (65.9 to 89.8%)	4.1% (1.3 to 10.5%)	79.6% (57.7 to 93.3%)	60.4% (42.7 to 72.9%)	96.7% (92.5 to 99.0%)

Location Name	Deaths (95% UI)	Mortality per 100,000 (95% UI)	Ratio of observed mortality rate to expected	Percent change in mortality rate 1990-2017 (95% UI)	Absolute difference in mortality rate 1990-2017 (95% UI)	Deaths per 100,000 episodes (95% UI)	Nutrition-associated risks attributable fraction (95% UI)	Low rotavirus vaccine attributable fraction (95% UI)	Unsafe WASH attributable fraction (95% UI)	Low ORS coverage attributable fraction (95% UI)	Total risk attributable fraction (95% UI)
Palestine	17 (12-25)	2.6 (1.8-3.8)	2	-75.7% (-86.4 to -55.0%)	-8.2 (-4.7 to -12.5)	1.3 (1.1-1.6)	78.3% (61.6 to 87.8%)	7.5% (2.9 to 15.1%)	81.7% (59.0 to 94.3%)	59.9% (42.2 to 72.7%)	96.7% (92.3 to 99.1%)
Oman	4 (2-6)	1.0 (0.6-1.5)	1.1	-95.4% (-97.8 to -88.5%)	-19.7 (-10.7 to -30.7)	0.5 (0.4-0.6)	82.7% (70.0 to 90.1%)	46.2% (30.2 to 65.1%)	65.1% (37.9 to 85.5%)	59.1% (41.3 to 72.1%)	96.3% (92.1 to 98.6%)
Qatar	1 (1-2)	0.7 (0.5-1.1)	2.2	-66.3% (-80.6 to -40.0%)	-1.4 (-0.9 to -2.2)	0.6 (0.5-0.7)	77.2% (58.6 to 87.1%)	4.0% (1.4 to 9.3%)	55.9% (31.1 to 79.8%)	59.0% (41.3 to 71.8%)	90.9% (83.0 to 96.4%)
Saudi Arabia	36 (20-64)	1.5 (0.8-2.6)	1	-98.1% (-99.1 to -95.9%)	-75.6 (-46.3 to -119.5)	0.8 (0.5-1.0)	73.5% (53.0 to 84.7%)	3.7% (1.3 to 8.7%)	54.6% (29.5 to 78.2%)	59.2% (41.4 to 72.1%)	88.8% (79.7 to 95.3%)
Sudan	7,215 (3,821-11,469)	118.9 (63.0-189.1)	4.7	-76.2% (-86.2 to -58.2%)	-380.7 (-220.5 to -548.6)	28.6 (17.0-40.3)	90.6% (82.4 to 95.2%)	7.2% (4.0 to 11.6%)	95.7% (89.2 to 98.9%)	62.8% (45.1 to 75.1%)	99.7% (99.3 to 99.9%)
Syria	18 (10-27)	1.2 (0.7-1.9)	0.4	-97.0% (-98.5 to -94.5%)	-39.8 (-26.1 to -55.3)	0.8 (0.6-0.9)	85.9% (73.4 to 92.8%)	26.9% (17.3 to 38.6%)	66.2% (42.3 to 85.9%)	64.1% (46.6 to 76.1%)	96.7% (93.0 to 98.8%)
Tunisia	21 (9-40)	2.3 (1.0-4.4)	1	-92.6% (-96.5 to -85.5%)	-28.7 (-13.5 to -47.7)	1.3 (0.7-2.1)	73.1% (53.6 to 85.2%)	19.0% (14.0 to 24.9%)	74.7% (52.3 to 90.4%)	48.7% (31.2 to 62.5%)	94.2% (88.3 to 98.0%)
Turkey	151 (100-220)	2.6 (1.7-3.7)	0.3	-98.0% (-98.8 to -96.5%)	-124.9 (-86.4 to -177.7)	1.8 (1.5-2.1)	74.0% (55.6 to 85.1%)	16.9% (13.1 to 21.4%)	73.5% (48.8 to 90.1%)	63.7% (46.2 to 75.8%)	93.3% (86.4 to 97.7%)
United Arab Emirates	6 (4-9)	1.5 (0.9-2.3)	2.2	-70.6% (-84.5 to -40.0%)	-3.5 (-2.1 to -5.6)	0.7 (0.6-0.9)	79.9% (65.2 to 89.0%)	5.7% (2.2 to 12.0%)	55.8% (29.1 to 79.5%)	59.0% (41.1 to 72.0%)	95.0% (90.2 to 98.1%)
Yemen	7,400 (4,726-11,260)	154.0 (98.4-234.4)	2.8	-80.4% (-87.6 to -66.7%)	-630.1 (-334.1 to -887.5)	32.4 (25.4-41.5)	94.3% (89.3 to 97.0%)	16.7% (9.6 to 26.3%)	92.7% (82.3 to 98.0%)	62.5% (45.0 to 74.7%)	99.8% (99.4 to 99.9%)
South Asia	135,390 (115,688-156,734)	77.6 (66.3-89.9)	2.5	-81.4% (-85.8 to -76.1%)	-339.4 (-282.5 to -397.7)	74.8 (71.6-77.1)	89.6% (84.1 to 92.9%)	16.9% (10.9 to 24.5%)	92.9% (82.9 to 97.9%)	53.5% (36.1 to 67.7%)	99.6% (99.1 to 99.9%)
Bangladesh	3,062 (1,743-5,087)	21.0 (11.9-34.9)	0.5	-95.1% (-97.4 to -91.4%)	-407.1 (-329.1 to -479.9)	24.7 (16.6-35.3)	85.8% (74.8 to 92.4%)	32.4% (27.8 to 37.0%)	96.0% (89.9 to 98.9%)	34.6% (19.7 to 49.2%)	99.7% (99.2 to 99.9%)
Bhutan	7 (3-15)	8.4 (3.5-17.0)	0.7	-95.9% (-98.1 to -91.7%)	-193.9 (-97.1 to -339.1)	6.4 (3.4-10.3)	77.9% (62.5 to 87.8%)	18.9% (12.1 to 27.4%)	83.1% (66.9 to 94.2%)	45.7% (24.8 to 65.1%)	97.1% (93.8 to 99.1%)
India	102,678 (87,608-118,510)	79.4 (67.7-91.6)	3	-79.7% (-84.4 to -73.3%)	-311.4 (-249.2 to -370.1)	91.0 (85.0-97.0)	89.9% (84.9 to 93.1%)	19.7% (12.4 to 28.4%)	92.8% (82.5 to 97.8%)	53.3% (35.9 to 68.1%)	99.7% (99.2 to 99.9%)

Location Name	Deaths (95% UI)	Mortality per 100,000 (95% UI)	Ratio of observed mortality rate to expected	Percent change in mortality rate 1990-2017 (95% UI)	Absolute difference in mortality rate 1990-2017 (95% UI)	Deaths per 100,000 episodes (95% UI)	Nutrition-associated risks attributable fraction (95% UI)	Low rotavirus vaccine attributable fraction (95% UI)	Unsafe WASH attributable fraction (95% UI)	Low ORS coverage attributable fraction (95% UI)	Total risk attributable fraction (95% UI)
Nepal	753 (445-1,191)	24.6 (14.5-38.9)	0.6	-94.9% (-97.3 to -89.6%)	-455.6 (-311.6 to -633.6)	22.8 (16.6-29.5)	76.9% (60.6 to 87.0%)	9.8% (7.3 to 13.0%)	93.5% (83.8 to 98.1%)	50.6% (32.2 to 67.1%)	99.0% (97.3 to 99.7%)
Pakistan	28,890 (18,641-43,911)	105.7 (68.2-160.7)	1.6	-81.4% (-89.2 to -68.5%)	-462.7 (-355.5 to -578.4)	55.1 (42.1-69.7)	89.4% (81.0 to 94.3%)	5.8% (4.2 to 7.7%)	93.2% (83.0 to 98.1%)	56.1% (35.9 to 70.3%)	99.6% (98.8 to 99.9%)
Sub-Saharan Africa	334,306 (285,351-388,790)	204.6 (174.7-238.0)	1.7	-68.4% (-74.2 to -60.3%)	-443.0 (-352.9 to -523.8)	91.3 (91.0-91.4)	88.5% (80.6 to 92.6%)	24.4% (19.2 to 30.0%)	96.2% (90.1 to 99.0%)	59.5% (41.7 to 72.4%)	99.7% (99.1 to 99.9%)
Central Sub-Saharan Africa	34,800 (25,798-46,206)	176.1 (130.6-233.9)	2.3	-63.5% (-73.9 to -49.3%)	-306.9 (-246.9 to -364.9)	59.5 (52.6-67.7)	88.6% (79.2 to 93.4%)	39.8% (32.0 to 47.2%)	96.5% (91.2 to 99.1%)	60.5% (42.2 to 73.4%)	99.7% (99.2 to 99.9%)
Angola	10,236 (7,080-14,044)	206.3 (142.7-283.0)	2.6	-81.2% (-87.8 to -70.6%)	-891.4 (-616.5 to -1,176.0)	70.7 (58.8-81.6)	86.8% (76.5 to 92.4%)	21.7% (15.5 to 29.5%)	96.0% (89.6 to 99.0%)	58.1% (39.8 to 72.1%)	99.6% (98.8 to 99.9%)
Central African Republic	4,156 (2,338-6,558)	685.8 (385.7-1,082.0)	5.8	54.3% (-12.7 to 167.5%)	241.4 (114.6 to 430.5)	203.9 (149.1-248.7)	89.3% (81.0 to 94.1%)	31.1% (24.7 to 37.6%)	97.2% (92.5 to 99.3%)	65.2% (47.7 to 77.1%)	99.8% (99.4 to 99.9%)
Congo	710 (410-1,098)	112.5 (64.9-174.0)	4.7	-49.9% (-71.3 to -17.8%)	-111.9 (-81.8 to -157.8)	37.2 (26.4-47.9)	89.9% (80.2 to 94.6%)	23.3% (17.0 to 30.1%)	95.6% (89.2 to 98.8%)	60.6% (42.2 to 73.8%)	99.7% (99.1 to 99.9%)
Democratic Republic of the Congo	19,514 (12,250-29,439)	148.2 (93.0-223.5)	1	-56.5% (-73.6 to -29.1%)	-192.6 (-153.7 to -221.3)	50.1 (38.2-64.1)	89.4% (80.0 to 94.7%)	53.4% (46.2 to 60.2%)	96.7% (91.6 to 99.1%)	60.8% (42.2 to 73.6%)	99.7% (99.3 to 99.9%)
Equatorial Guinea	89 (41-159)	47.0 (21.9-83.8)	11	-92.2% (-96.5 to -84.5%)	-555.9 (-343.7 to -793.6)	18.5 (11.4-25.1)	81.8% (67.6 to 90.0%)	42.4% (28.1 to 60.1%)	92.3% (81.6 to 97.8%)	59.9% (41.2 to 73.0%)	98.8% (97.1 to 99.7%)
Gabon	95 (43-173)	47.5 (21.8-86.8)	3.6	-71.6% (-85.0 to -49.9%)	-119.5 (-76.4 to -156.6)	16.1 (9.0-24.6)	79.1% (62.9 to 88.7%)	40.4% (26.9 to 56.8%)	91.3% (81.3 to 97.3%)	59.5% (41.9 to 72.7%)	98.5% (96.6 to 99.6%)
Eastern Sub-Saharan Africa	98,175 (84,620-114,013)	155.1 (133.7-180.1)	1.9	-70.3% (-76.4 to -62.3%)	-367.1 (-287.5 to -446.7)	72.2 (69.7-75.4)	86.7% (78.5 to 91.2%)	13.4% (8.7 to 19.0%)	96.7% (91.2 to 99.2%)	58.9% (41.1 to 71.5%)	99.7% (99.1 to 99.9%)
Burundi	3,045 (1,829-4,944)	166.3 (99.9-270.0)	0.7	-64.0% (-78.5 to -40.3%)	-295.1 (-232.7 to -344.0)	77.2 (60.2-96.8)	88.1% (79.0 to 93.5%)	3.6% (1.1 to 9.0%)	97.1% (92.3 to 99.3%)	59.9% (41.2 to 74.0%)	99.8% (99.3 to 99.9%)
Comoros	69 (42-108)	80.5 (49.0-126.4)	1.1	-83.3% (-90.2 to -72.5%)	-401.3 (-306.5 to -505.7)	36.1 (27.3-46.2)	88.4% (78.5 to 94.0%)	27.8% (17.9 to 39.1%)	95.8% (89.4 to 98.9%)	51.9% (32.7 to 68.1%)	99.7% (99.2 to 99.9%)

Location Name	Deaths (95% UI)	Mortality per 100,000 (95% UI)	Ratio of observed mortality rate to expected	Percent change in mortality rate 1990-2017 (95% UI)	Absolute difference in mortality rate 1990-2017 (95% UI)	Deaths per 100,000 episodes (95% UI)	Nutrition-associated risks attributable fraction (95% UI)	Low rotavirus vaccine attributable fraction (95% UI)	Unsafe WASH attributable fraction (95% UI)	Low ORS coverage attributable fraction (95% UI)	Total risk attributable fraction (95% UI)
Djibouti	102 (48-182)	67.6 (32.0-120.4)	1.3	-82.2% (-90.5 to -69.5%)	-311.8 (-208.7 to -419.4)	46.7 (28.5-63.5)	89.7% (81.6 to 94.8%)	17.2% (10.0 to 26.1%)	95.8% (89.4 to 98.8%)	61.1% (42.1 to 74.8%)	99.8% (99.4 to 99.9%)
Eritrea	1,585 (945-2,508)	193.4 (115.3-306.1)	2.6	-76.8% (-87.1 to -56.4%)	-639.9 (-394.2 to -900.1)	92.3 (70.9-111.8)	88.8% (79.7 to 94.1%)	0.3% (0.1 to 0.8%)	96.5% (90.9 to 99.1%)	56.3% (36.4 to 71.9%)	99.7% (99.3 to 99.9%)
Ethiopia	31,805 (26,081-38,114)	191.3 (156.9-229.2)	3.5	-64.3% (-74.1 to -49.7%)	-344.9 (-234.5 to -457.2)	96.4 (94.5-97.1)	89.3% (82.1 to 93.1%)	17.8% (11.0 to 25.9%)	96.7% (91.8 to 99.2%)	63.8% (45.8 to 76.0%)	99.7% (99.3 to 99.9%)
Kenya	9,552 (7,681-11,489)	146.7 (118.0-176.5)	3.7	-42.1% (-58.2 to -25.0%)	-106.6 (-80.0 to -143.7)	67.5 (64.2-68.4)	83.0% (74.0 to 88.4%)	8.0% (4.1 to 13.6%)	96.0% (89.8 to 99.0%)	57.9% (40.2 to 71.0%)	99.5% (98.7 to 99.9%)
Madagascar	14,675 (9,248-21,904)	337.3 (212.5-503.4)	1	-56.3% (-73.2 to -33.4%)	-434.5 (-439.0 to -401.5)	111.0 (88.5-132.8)	87.2% (78.2 to 92.5%)	6.3% (2.9 to 11.0%)	97.0% (91.8 to 99.3%)	64.0% (46.4 to 76.7%)	99.7% (99.3 to 99.9%)
Malawi	2,538 (1,664-3,688)	92.6 (60.7-134.6)	0.6	-89.4% (-93.8 to -78.7%)	-778.7 (-429.6 to -1,040.3)	27.1 (21.7-33.7)	79.6% (66.0 to 87.9%)	8.0% (3.5 to 15.0%)	96.7% (91.4 to 99.1%)	43.9% (26.9 to 61.1%)	99.5% (98.6 to 99.9%)
Mozambique	4,393 (2,905-6,257)	93.4 (61.8-133.1)	2	-82.5% (-90.3 to -70.3%)	-439.2 (-294.4 to -789.7)	57.0 (48.3-63.7)	83.1% (69.9 to 90.9%)	6.5% (3.4 to 11.0%)	96.7% (91.4 to 99.2%)	50.9% (32.1 to 66.9%)	99.5% (98.8 to 99.9%)
Rwanda	1,887 (1,156-2,890)	98.6 (60.4-151.0)	0.6	-88.4% (-93.9 to -79.4%)	-749.4 (-537.2 to -1,000.8)	49.3 (36.4-64.1)	77.5% (63.5 to 86.9%)	3.0% (1.1 to 7.4%)	96.1% (90.1 to 99.0%)	60.5% (42.8 to 74.0%)	99.3% (98.0 to 99.8%)
Somalia	6,681 (3,765-11,254)	225.2 (126.9-379.4)	0.8	-68.6% (-81.5 to -38.3%)	-492.5 (-245.7 to -778.9)	108.6 (79.5-141.3)	91.3% (83.5 to 95.7%)	27.5% (17.7 to 38.8%)	96.8% (91.5 to 99.2%)	55.2% (35.9 to 70.7%)	99.8% (99.5 to 100.0%)
South Sudan	7,912 (4,971-11,405)	452.8 (284.5-652.7)	1.9	-37.8% (-59.7 to 4.5%)	-275.7 (-100.4 to -504.7)	149.1 (120.1-169.6)	90.8% (83.8 to 95.0%)	27.5% (17.7 to 38.7%)	97.3% (92.7 to 99.4%)	54.6% (35.1 to 69.6%)	99.9% (99.6 to 100.0%)
Tanzania	4,902 (2,939-7,719)	54.4 (32.6-85.7)	0.7	-86.5% (-92.3 to -76.1%)	-349.8 (-246.5 to -443.8)	32.8 (24.3-43.1)	80.8% (67.7 to 88.6%)	3.7% (1.6 to 7.4%)	96.0% (89.5 to 99.0%)	55.0% (36.1 to 70.0%)	99.4% (98.4 to 99.9%)
Uganda	5,261 (3,545-7,453)	75.8 (51.0-107.3)	3.3	-76.3% (-85.6 to -59.6%)	-243.3 (-157.2 to -346.5)	34.7 (28.5-41.4)	77.7% (63.1 to 86.7%)	27.4% (23.5 to 31.2%)	96.2% (90.0 to 99.0%)	54.6% (34.0 to 70.5%)	99.3% (98.2 to 99.8%)
Zambia	3,708 (2,510-5,277)	129.3 (87.5-184.0)	1.4	-78.4% (-86.9 to -62.9%)	-468.2 (-306.3 to -647.3)	53.1 (44.2-62.7)	84.6% (74.2 to 90.9%)	0.8% (0.4 to 1.5%)	95.8% (89.0 to 98.9%)	45.2% (25.9 to 63.1%)	99.5% (98.7 to 99.9%)
Southern Sub-	8,070 (6,972-9,314)	94.5 (81.7-109.1)	1.3	-67.9% (-73.3 to -61.4%)	-200.1 (-173.6 to -223.6)	85.4 (85.4-86.6)	81.5% (68.3 to 88.6%)	7.1% (4.1 to 11.1%)	89.0% (78.3 to 96.0%)	53.2% (35.2 to 66.7%)	98.3% (96.2 to 99.4%)

Location Name	Deaths (95% UI)	Mortality per 100,000 (95% UI)	Ratio of observed mortality rate to expected	Percent change in mortality rate 1990-2017 (95% UI)	Absolute difference in mortality rate 1990-2017 (95% UI)	Deaths per 100,000 episodes (95% UI)	Nutrition-associated risks attributable fraction (95% UI)	Low rotavirus vaccine attributable fraction (95% UI)	Unsafe WASH attributable fraction (95% UI)	Low ORS coverage attributable fraction (95% UI)	Total risk attributable fraction (95% UI)
Saharan Africa											
Botswana	196 (137-278)	82.0 (57.0-116.1)	3.9	-62.1% (-75.1 to -42.9%)	-134.3 (-101.5 to -166.3)	57.2 (50.3-64.0)	85.0% (72.7 to 91.5%)	16.1% (9.9 to 23.3%)	90.7% (79.9 to 97.1%)	51.2% (33.1 to 65.9%)	98.8% (97.2 to 99.7%)
Lesotho	474 (293-738)	209.5 (129.6-326.1)	3.4	-47.0% (-67.8 to -14.4%)	-185.6 (-166.8 to -171.9)	114.4 (87.1-147.7)	80.0% (67.2 to 88.3%)	7.7% (3.3 to 14.7%)	96.7% (91.6 to 99.2%)	49.4% (31.3 to 64.3%)	99.4% (98.5 to 99.9%)
Namibia	321 (194-492)	110.8 (67.2-170.0)	2.2	-67.0% (-80.0 to -50.8%)	-225.3 (-170.3 to -256.2)	49.7 (38.4-61.1)	86.1% (73.4 to 92.2%)	4.0% (1.6 to 8.0%)	92.5% (82.4 to 97.7%)	43.1% (25.8 to 57.5%)	99.1% (97.9 to 99.8%)
South Africa	4,616 (3,941-5,353)	84.9 (72.5-98.5)	0.9	-76.8% (-81.0 to -72.2%)	-281.1 (-243.2 to -317.7)	225.3 (215.7-238.6)	82.9% (70.2 to 89.6%)	5.1% (2.2 to 9.5%)	84.5% (70.9 to 94.0%)	53.3% (35.7 to 67.0%)	97.6% (95.0 to 99.2%)
Swaziland	247 (154-379)	170.3 (106.2-261.2)	2.9	-50.3% (-67.7 to -27.0%)	-172.6 (-101.6 to -210.4)	76.9 (57.2-101.0)	75.7% (61.1 to 85.1%)	11.9% (6.1 to 19.9%)	94.8% (87.3 to 98.6%)	41.3% (24.6 to 56.6%)	98.8% (97.0 to 99.7%)
Zimbabwe	2,216 (1,457-3,112)	100.6 (66.2-141.3)	2	6.4% (-34.7 to 76.6%)	6.1 (1.8 to 11.1)	39.0 (30.5-48.0)	78.7% (64.1 to 87.7%)	10.5% (6.2 to 16.1%)	95.5% (88.5 to 98.8%)	56.7% (38.7 to 70.2%)	99.1% (97.8 to 99.8%)
Western Sub-Saharan Africa	193,260 (157,286-236,075)	269.3 (219.1-328.9)	1.7	-70.1% (-76.9 to -60.2%)	-630.4 (-480.1 to -785.7)	119.0 (111.2-126.9)	89.8% (82.2 to 93.6%)	29.2% (23.7 to 35.0%)	96.1% (89.9 to 99.0%)	59.9% (41.0 to 72.9%)	99.7% (99.2 to 99.9%)
Benin	3,614 (2,232-5,495)	189.4 (116.9-287.9)	2.3	-61.5% (-77.8 to -35.8%)	-302.7 (-241.3 to -355.6)	129.8 (92.8-170.6)	89.1% (79.8 to 94.0%)	31.2% (20.2 to 44.3%)	96.5% (91.1 to 99.1%)	55.4% (35.8 to 70.6%)	99.7% (99.3 to 99.9%)
Burkina Faso	7,517 (4,875-11,033)	201.4 (130.6-295.5)	1.5	-73.0% (-83.8 to -55.3%)	-543.0 (-386.3 to -749.5)	83.8 (68.8-98.2)	91.4% (84.3 to 95.4%)	11.3% (5.6 to 19.7%)	97.1% (92.5 to 99.3%)	63.7% (45.4 to 76.5%)	99.8% (99.6 to 100.0%)
Cameroon	7,944 (4,065-13,144)	193.1 (98.8-319.5)	1.8	-68.9% (-81.8 to -51.6%)	-427.1 (-304.3 to -566.3)	62.7 (36.5-90.5)	84.1% (71.9 to 90.9%)	11.4% (5.7 to 19.4%)	96.1% (90.1 to 99.0%)	64.3% (46.4 to 76.9%)	99.5% (98.7 to 99.9%)
Cape Verde	6 (4-9)	11.5 (7.3-17.8)	0.6	-96.5% (-98.0 to -93.9%)	-314.0 (-217.0 to -430.9)	8.5 (6.8-10.2)	70.9% (51.5 to 82.2%)	31.4% (20.3 to 44.2%)	92.6% (83.0 to 97.8%)	58.8% (39.8 to 73.5%)	98.2% (95.6 to 99.5%)
Chad	20,057 (14,653-26,592)	654.3 (478.0-867.5)	2.5	-46.9% (-62.5 to -22.7%)	-577.1 (-381.3 to -812.6)	161.6 (132.7-189.3)	92.5% (86.5 to 95.8%)	30.9% (20.1 to 44.7%)	97.2% (92.4 to 99.3%)	62.2% (43.3 to 75.3%)	99.9% (99.6 to 100.0%)

Location Name	Deaths (95% UI)	Mortality per 100,000 (95% UI)	Ratio of observed mortality rate to expected	Percent change in mortality rate 1990-2017 (95% UI)	Absolute difference in mortality rate 1990-2017 (95% UI)	Deaths per 100,000 episodes (95% UI)	Nutrition-associated risks attributable fraction (95% UI)	Low rotavirus vaccine attributable fraction (95% UI)	Unsafe WASH attributable fraction (95% UI)	Low ORS coverage attributable fraction (95% UI)	Total risk attributable fraction (95% UI)
Cote d'Ivoire	7,608 (4,839-11,201)	196.5 (125.0-289.4)	2.8	-44.0% (-64.5 to -15.4%)	-154.7 (-135.0 to -165.2)	67.8 (54.3-81.5)	85.8% (73.5 to 92.4%)	19.6% (11.4 to 29.6%)	95.8% (89.6 to 98.8%)	63.3% (46.5 to 76.4%)	99.5% (98.7 to 99.9%)
The Gambia	288 (178-428)	90.3 (56.0-134.2)	2.4	-63.1% (-77.1 to -44.0%)	-154.6 (-132.4 to -173.2)	35.0 (27.3-42.4)	88.8% (78.4 to 94.0%)	2.8% (1.3 to 5.1%)	95.9% (89.8 to 98.9%)	54.5% (35.8 to 69.6%)	99.7% (99.1 to 99.9%)
Ghana	3,628 (2,306-5,447)	89.4 (56.8-134.2)	1.3	-79.0% (-87.7 to -63.5%)	-336.8 (-250.1 to -434.5)	40.7 (32.2-50.1)	85.0% (72.4 to 91.9%)	7.7% (3.7 to 13.0%)	96.3% (90.5 to 99.0%)	58.0% (40.0 to 71.0%)	99.6% (98.9 to 99.9%)
Guinea	2,763 (1,775-4,048)	140.5 (90.3-205.9)	1.8	-72.1% (-83.2 to -53.8%)	-362.6 (-265.3 to -471.9)	55.3 (42.3-69.5)	87.0% (76.3 to 92.9%)	31.1% (20.2 to 44.4%)	96.8% (91.5 to 99.2%)	58.4% (39.0 to 72.6%)	99.7% (99.2 to 99.9%)
Guinea-Bissau	738 (479-1,097)	241.2 (156.5-358.3)	2.4	-64.5% (-78.2 to -39.2%)	-438.3 (-285.2 to -582.9)	119.2 (92.0-150.7)	85.5% (73.3 to 91.8%)	17.6% (9.0 to 29.7%)	96.8% (91.6 to 99.2%)	62.6% (44.9 to 75.6%)	99.6% (99.0 to 99.9%)
Liberia	1,195 (628-1,959)	169.0 (88.7-277.0)	0.6	-85.0% (-92.5 to -72.5%)	-960.4 (-750.5 to -1,158.5)	47.4 (28.1-69.0)	84.8% (71.8 to 91.9%)	18.7% (10.2 to 30.1%)	96.9% (91.8 to 99.2%)	49.4% (30.7 to 66.1%)	99.6% (98.9 to 99.9%)
Mali	8,704 (5,093-13,707)	233.0 (136.4-367.0)	1.1	-77.3% (-86.8 to -62.6%)	-794.2 (-593.7 to -961.6)	115.4 (79.2-152.8)	88.5% (79.1 to 93.7%)	6.6% (4.3 to 9.2%)	96.8% (91.6 to 99.2%)	60.0% (42.2 to 73.9%)	99.8% (99.4 to 100.0%)
Mauritania	628 (357-978)	109.7 (62.4-171.0)	1.2	-83.4% (-90.2 to -73.0%)	-551.9 (-401.8 to -699.0)	50.8 (37.1-61.7)	92.0% (86.3 to 95.8%)	13.3% (6.1 to 22.7%)	95.8% (89.6 to 98.9%)	62.7% (45.5 to 75.8%)	99.9% (99.7 to 100.0%)
Niger	16,939 (11,091-24,281)	387.8 (253.9-555.9)	0.7	-77.6% (-86.6 to -64.3%)	-1,344.2 (-969.9 to -1,735.1)	157.5 (123.5-188.7)	93.5% (88.0 to 96.5%)	16.0% (11.4 to 21.2%)	97.1% (92.2 to 99.3%)	57.8% (40.5 to 72.3%)	99.9% (99.7 to 100.0%)
Nigeria	104,267 (75,975-139,594)	302.7 (220.6-405.3)	1.6	-72.2% (-81.6 to -56.2%)	-785.2 (-517.5 to -1,053.9)	161.4 (135.9-187.7)	89.8% (81.9 to 93.9%)	40.4% (35.3 to 45.1%)	95.7% (88.8 to 98.9%)	59.5% (40.8 to 73.1%)	99.7% (99.1 to 99.9%)
Sao Tome and Principe	8 (4-13)	31.5 (16.6-53.4)	0.8	-92.1% (-96.0 to -85.1%)	-368.5 (-278.1 to -454.5)	13.6 (9.1-18.6)	84.3% (70.5 to 91.6%)	6.6% (2.7 to 13.3%)	92.7% (82.9 to 97.9%)	55.3% (35.9 to 70.6%)	99.1% (97.9 to 99.8%)
Senegal	3,300 (2,109-4,807)	145.6 (93.0-212.1)	0.9	-79.2% (-87.2 to -68.3%)	-554.3 (-454.3 to -645.8)	48.5 (37.2-59.3)	87.0% (75.2 to 92.7%)	6.4% (2.7 to 13.0%)	94.4% (86.5 to 98.4%)	63.4% (45.5 to 76.1%)	99.4% (98.5 to 99.9%)
Sierra Leone	2,430 (1,467-3,705)	203.4 (122.8-310.0)	1.4	-76.9% (-86.6 to -62.3%)	-678.8 (-534.4 to -824.0)	103.0 (72.0-135.5)	88.9% (78.9 to 94.3%)	9.2% (3.8 to 17.7%)	96.9% (91.7 to 99.2%)	39.9% (21.7 to 57.3%)	99.7% (99.3 to 99.9%)
Togo	1,625 (899-2,698)	151.9 (84.0-252.3)	1.4	-75.7% (-84.9 to -62.1%)	-473.9 (-321.0 to -654.4)	54.3 (34.4-78.9)	87.0% (76.3 to 92.9%)	12.6% (7.3 to 20.6%)	96.6% (91.2 to 99.1%)	65.0% (47.0 to 77.1%)	99.7% (99.2 to 99.9%)

Supplementary results table 2. The percent change in deaths due to diarrhea between 1990 and 2017 due to changes in exposure to risk factors is shown for each country. These are the results shown in the Manuscript Figure 4 with 95% uncertainty intervals presented in parentheses. The countries are ordered to match the ordering of the panels in the manuscript.

Location	Hand-washing	Low rotavirus vaccine coverage	Unsafe sanitation	Unsafe water	Zinc deficiency	Stunting	Under-weight	Wasting	Low oral rehydration solution coverage	Low weight and short gestation	Sub-optimal Breast feeding	Vitamin A deficiency
Global	2.0% (1.2 to 3.0%)	-1.4% (0.3 to - 4.7%)	-13.3% (-11.2 to - 15.5%)	-1.6% (0.4 to - 6.0%)	-1.3% (0 to - 3.3%)	-4.4% (-1.9 to - 7.2%)	-5.5% (-4.7 to - 6.7%)	-9.9% (-9.6 to - 10.2%)	-6.9% (-4.8 to - 8.4%)	2.0% (1.8 to 2.0%)	-1.1% (-0.7 to - 1.5%)	-3.9% (-3.5 to - 3.9%)
Ghana	-1.2% (-0.7 to - 1.8%)	-6.6% (-6.0 to - 6.6%)	-10.4% (-9.4 to - 10.7%)	-3.8% (-1.0 to - 8.1%)	-11.0% (0 to - 24.7%)	-5.2% (-2.2 to - 8.7%)	-5.0% (-4.6 to - 6.0%)	-17.0% (-16.5 to - 11.5%)	-5.0% (-4.1 to - 6.0%)	-0.4% (-0.6 to 0.0%)	-4.8% (-3.2 to - 6.3%)	-13.1% (-12.8 to - 12.3%)
Bolivia	-6.1% (-2.9 to - 9.0%)	-5.2% (-3.6 to - 7.4%)	-12.7% (-12.1 to - 12.5%)	-21.0% (-11.8 to - 27.2%)	-0.7% (0 to - 2.3%)	-5.4% (-2.2 to - 9.1%)	-2.0% (-1.7 to - 2.3%)	-13.8% (-9.2 to - 16.0%)	-4.8% (-3.0 to - 5.6%)	0.4% (0.4 to 0.4%)	-1.3% (-0.9 to - 1.8%)	-3.2% (-3.0 to - 3.6%)
Ethiopia	-0.5% (-0.2 to - 0.7%)	-1.2% (-1.0 to - 2.4%)	-2.8% (-1.4 to - 4.2%)	-13.9% (-7.5 to - 23.0%)	0.0% (0 to - 0.4%)	-7.2% (-2.9 to - 12.2%)	-10.6% (-8.7 to - 14.1%)	-21.0% (-18.2 to - 22.8%)	-5.0% (-4.7 to - 6.1%)	3.8% (2.9 to 4.2%)	-1.2% (-0.3 to - 2.7%)	-5.9% (-5.4 to - 6.3%)
Tanzania	-0.7% (-0.5 to - 1.1%)	-15.3% (-9.2 to - 23.5%)	-6.6% (-6.5 to - 7.1%)	-10.0% (-7.1 to - 12.6%)	-0.2% (0 to - 0.8%)	-4.6% (-1.9 to - 7.9%)	-4.9% (-4.1 to - 6.0%)	-21.6% (-17.9 to - 21.4%)	7.5% (4.5 to 8.7%)	0.5% (0.6 to 0.4%)	-5.9% (-4.5 to - 7.4%)	-14.3% (-11.7 to - 16.4%)
Guinea	-0.5% (-0.3 to - 0.9%)	-0.3% (0.0 to - 1.7%)	-8.1% (-7.4 to - 9.0%)	-7.2% (-2.8 to - 12.4%)	-0.8% (0 to - 2.6%)	-3.4% (-1.6 to - 5.7%)	-4.0% (-3.4 to - 5.3%)	-39.4% (-36.2 to - 33.5%)	-3.8% (-5.7 to - 3.7%)	2.9% (2.6 to 3.1%)	-0.9% (0.0 to - 1.8%)	-11.1% (-8.8 to - 11.9%)
Sao Tome and Principe	-5.5% (-2.7 to - 7.8%)	-15.6% (-9.7 to - 23.8%)	-16.8% (-16.0 to - 17.5%)	-19.1% (-13.5 to - 25.4%)	-0.7% (0 to - 3.0%)	-4.7% (-1.7 to - 8.1%)	-3.3% (-2.7 to - 4.5%)	-9.5% (-7.9 to - 6.3%)	-5.4% (-4.5 to - 6.0%)	0.6% (0.6 to 0.4%)	-1.4% (-0.8 to - 2.1%)	-9.5% (-9.4 to - 8.7%)
Nicaragua	-5.5% (-2.6 to - 8.2%)	-9.1% (-6.0 to - 13.2%)	-13.6% (-13.4 to - 13.7%)	-20.1% (-14.5 to - 23.9%)	-0.4% (0 to - 1.3%)	-2.0% (-0.9 to - 3.3%)	-1.4% (-1.2 to - 1.6%)	-18.1% (-13.6 to - 19.4%)	-6.5% (-4.1 to - 8.1%)	0.4% (0.4 to 0.3%)	-1.3% (-1.0 to - 1.8%)	-4.9% (-4.1 to - 5.8%)

Location	Hand-washing	Low rotavirus vaccine coverage	Unsafe sanitation	Unsafe water	Zinc deficiency	Stunting	Under-weight	Wasting	Low oral rehydration solution coverage	Low weight and short gestation	Sub-optimal Breast feeding	Vitamin A deficiency
Sudan	-1.2% (-1.0 to -1.7%)	-8.9% (-5.5 to -13.2%)	-5.4% (-3.8 to -6.0%)	2.6% (2.3 to 0.9%)	-2.6% (0 to -7.8%)	-6.2% (-2.5 to -11.0%)	-6.9% (-5.0 to -10.4%)	-24.7% (-27.0 to -16.7%)	1.5% (1.1 to 1.9%)	2.4% (1.8 to 2.6%)	-6.7% (-4.0 to -9.8%)	-7.1% (-6.7 to -6.6%)
Guatemala	-8.1% (-4.1 to -12.5%)	-0.9% (-2.5 to 2.5%)	-18.6% (-17.1 to -19.8%)	-17.2% (-13.2 to -21.4%)	-0.2% (0 to -0.9%)	-5.7% (-2.6 to -8.4%)	-5.8% (-4.9 to -7.1%)	-40.9% (-30.0 to -45.7%)	-9.0% (-6.7 to -11.2%)	0.2% (0.2 to 0.2%)	-0.6% (0.0 to -1.6%)	-4.8% (-4.3 to -5.6%)
Comoros	-1.8% (-1.1 to -2.6%)	0.7% (0.6 to 0.2%)	-3.7% (-3.5 to -3.0%)	-10.7% (-7.7 to -13.4%)	0.3% (0 to 0.7%)	-4.8% (-1.8 to -8.7%)	-4.8% (-3.5 to -6.7%)	-18.6% (-15.7 to -19.1%)	-11.9% (-9.2 to -12.8%)	1.5% (1.5 to 1.5%)	0.3% (0.6 to -0.4%)	-6.1% (-5.8 to -5.7%)
Bangladesh	-1.3% (-0.7 to -2.1%)	8.0% (6.1 to 10.3%)	-7.8% (-6.6 to -9.0%)	-2.4% (-1.7 to -3.5%)	-1.3% (0 to -3.1%)	-6.1% (-2.4 to -10.2%)	-8.7% (-6.9 to -11.7%)	-18.3% (-16.0 to -19.0%)	-14.2% (-9.4 to -17.2%)	0.2% (0.2 to 0.2%)	-1.3% (-0.6 to -2.2%)	-4.1% (-3.2 to -5.3%)
Cameroon	-1.6% (-1.0 to -2.8%)	-30.0% (-18.4 to -50.7%)	-8.8% (-8.7 to -10.1%)	-11.6% (-7.0 to -18.5%)	-1.1% (0 to -3.8%)	-8.6% (-3.5 to -15.1%)	-4.2% (-3.0 to -5.9%)	-14.5% (-11.3 to -20.2%)	0.0% (-1.1 to 0.9%)	2.6% (2.5 to 2.2%)	-2.9% (-2.4 to -3.3%)	-16.2% (-14.2 to -16.9%)
Afghanistan	-2.5% (-1.1 to -3.6%)	0.1% (0.6 to -1.1%)	-17.4% (-13.5 to -21.5%)	-4.6% (-2.0 to -10.3%)	5.5% (0 to 15.4%)	-7.9% (-3.4 to -12.9%)	-9.4% (-6.8 to -13.1%)	-37.7% (-30.6 to -41.0%)	0.1% (-0.6 to -0.3%)	3.0% (3.6 to 2.7%)	-1.1% (-0.4 to -1.9%)	-3.7% (-4.9 to -2.6%)
Madagascar	0.4% (0.4 to 0.2%)	-7.4% (-7.3 to -5.5%)	1.4% (0.3 to 2.7%)	-2.9% (-1.4 to -5.1%)	0.4% (0 to 1.9%)	-3.5% (-2.0 to -2.7%)	-3.0% (-3.9 to -2.7%)	-28.6% (-26.9 to -12.4%)	-0.3% (0.1 to 0.9%)	0.4% (0.2 to 0.7%)	-3.4% (-2.6 to -3.9%)	-5.6% (-4.5 to -6.6%)
Guinea-Bissau	-0.6% (-0.2 to -0.4%)	-25.3% (-15.8 to -43.6%)	-6.8% (-5.8 to -7.0%)	-5.3% (-2.9 to -10.5%)	-2.1% (0 to -6.8%)	-8.7% (-2.8 to -16.3%)	-4.7% (-2.8 to -6.9%)	-28.0% (-20.4 to -38.7%)	2.2% (3.0 to 3.5%)	1.7% (1.7 to 1.6%)	-3.2% (-2.3 to -4.4%)	-10.3% (-10.6 to -9.6%)
Mozambique	-0.9% (-0.6 to -1.2%)	-21.1% (-12.7 to -34.7%)	-5.8% (-4.9 to -7.1%)	-8.9% (-6.2 to -13.8%)	-7.1% (0 to -14.7%)	-8.7% (-3.6 to -14.2%)	-7.0% (-5.7 to -9.1%)	-47.1% (-37.3 to -48.0%)	-10.5% (-7.5 to -14.6%)	0.8% (0.9 to 0.9%)	-1.3% (-0.8 to -1.9%)	-12.5% (-12.3 to -11.4%)
Laos	-3.2% (-1.5 to -5.5%)	25.9% (22.6 to 27.6%)	-12.3% (-11.2 to -13.5%)	-13.8% (-11.3 to -18.9%)	-1.5% (0 to -4.5%)	-4.4% (-2.0 to -6.7%)	-9.6% (-7.9 to -12.6%)	-55.2% (-50.2 to -52.4%)	-10.7% (-8.9 to -12.4%)	0.9% (0.9 to 0.9%)	-1.9% (-0.6 to -3.6%)	-6.8% (-6.7 to -6.0%)
Egypt	-5.3% (-2.0 to -8.9%)	17.1% (10.2 to 28.5%)	-30.6% (-28.2 to -32.0%)	-28.2% (-15.6 to -40.4%)	0.0% (0 to -0.1%)	-1.1% (-0.6 to -1.8%)	-1.2% (-1.0 to -1.3%)	-13.5% (-12.2 to -9.9%)	-4.9% (-3.1 to -5.9%)	3.7% (3.8 to 3.6%)	0.4% (0.2 to 0.9%)	-4.3% (-3.9 to -5.0%)
Nepal	-5.9% (-3.0 to -8.4%)	-6.4% (-3.3 to -11.3%)	-17.7% (-15.3 to -19.6%)	-7.6% (-5.4 to -10.3%)	-0.5% (0 to -2.0%)	-6.3% (-2.1 to -12.8%)	-6.5% (-3.9 to -12.9%)	-17.3% (-13.2 to -21.1%)	-12.3% (-8.9 to -13.7%)	2.2% (2.3 to 2.2%)	0.0% (0.1 to -0.3%)	-5.5% (-4.0 to -6.9%)
Pakistan	-5.3% (-2.6 to -8.5%)	-3.3% (-2.8 to -4.0%)	-24.5% (-21.3 to -27.7%)	-3.4% (-0.6 to -8.1%)	-1.7% (0 to -3.6%)	-5.9% (-2.7 to -8.4%)	-3.2% (-2.3 to -5.0%)	14.5% (10.2 to 21.9%)	-0.7% (-2.5 to -2.5%)	4.9% (5.8 to 4.4%)	-0.2% (0.2 to -0.6%)	-10.6% (-8.8 to -12.8%)
Zambia	-1.5% (-0.7 to -2.5%)	-27.7% (-16.4 to -45.9%)	-2.1% (-1.1 to -3.4%)	-5.5% (-4.0 to -7.4%)	-0.1% (0 to -0.5%)	-5.0% (-2.1 to -8.8%)	-2.8% (-2.0 to -3.6%)	-1.2% (0.3 to -2.0%)	-10.3% (-7.8 to -9.2%)	0.6% (0.7 to 0.5%)	-5.5% (-4.4 to -6.2%)	-14.5% (-13.2 to -14.1%)

Location	Hand-washing	Low rotavirus vaccine coverage	Unsafe sanitation	Unsafe water	Zinc deficiency	Stunting	Under-weight	Wasting	Low oral rehydration solution coverage	Low weight and short gestation	Sub-optimal Breast feeding	Vitamin A deficiency
Togo	-0.6% (-0.2 to -0.6%)	-34.3% (-19.3 to -63.2%)	-7.8% (-6.6 to -8.3%)	-4.8% (-2.2 to -7.7%)	-1.7% (0 to -5.1%)	-5.1% (-1.9 to -9.2%)	-4.4% (-3.6 to -5.9%)	13.1% (11.2 to 8.4%)	0.9% (0.4 to 0.6%)	1.5% (1.6 to 1.2%)	-4.1% (-3.1 to -5.1%)	-9.7% (-7.4 to -11.0%)
Somalia	-0.8% (-0.3 to -1.7%)	1.2% (1.0 to 0.7%)	-5.3% (-4.7 to -6.9%)	-10.2% (-5.8 to -15.0%)	1.0% (0 to 3.4%)	-4.0% (-2.0 to -6.8%)	-4.5% (-3.2 to -6.8%)	-37.0% (-34.7 to -34.3%)	-18.0% (-14.2 to -21.0%)	1.8% (1.3 to 1.7%)	0.8% (0.9 to 0.8%)	-7.8% (-8.0 to -6.5%)
Burkina Faso	-0.7% (-0.4 to -1.3%)	-39.2% (-22.4 to -73.7%)	-2.7% (-2.1 to -2.9%)	-5.4% (-2.9 to -10.7%)	0.1% (0 to 0.1%)	-9.0% (-3.6 to -16.1%)	-7.5% (-5.5 to -10.2%)	19.9% (20.9 to 16.4%)	-4.1% (-4.4 to -3.6%)	1.8% (2.1 to 1.6%)	-1.8% (-1.2 to -2.4%)	-13.1% (-11.3 to -13.3%)
Mauritania	-3.5% (-2.1 to -5.2%)	-15.2% (-9.9 to -23.9%)	-6.3% (-5.8 to -7.2%)	-8.7% (-6.4 to -11.6%)	0.4% (0 to 1.8%)	-5.1% (-2.1 to -8.7%)	-3.6% (-2.1 to -5.4%)	39.2% (37.4 to 38.4%)	-4.4% (-2.5 to -4.0%)	2.4% (2.7 to 2.2%)	-2.3% (-1.4 to -3.7%)	-8.8% (-7.3 to -10.0%)
Senegal	-1.4% (-0.6 to -1.8%)	-19.4% (-12.1 to -30.0%)	-11.9% (-10.6 to -12.5%)	-19.0% (-13.5 to -24.3%)	-2.4% (0 to -7.7%)	-5.1% (-1.9 to -8.9%)	-3.9% (-3.0 to -5.0%)	-10.0% (-8.1 to -11.5%)	-4.6% (-3.0 to -4.2%)	0.6% (0.6 to 0.6%)	-3.0% (-2.1 to -3.9%)	-13.5% (-11.8 to -14.6%)
Equatorial Guinea	-5.4% (-2.9 to -8.1%)	0.5% (0.8 to -2.8%)	-37.0% (-35.6 to -37.7%)	-24.9% (-18.0 to -33.3%)	-8.8% (0 to -18.2%)	-13.5% (-5.6 to -23.3%)	-10.4% (-7.6 to -14.1%)	-33.2% (-24.2 to -42.1%)	-5.7% (-5.7 to -6.5%)	3.6% (3.8 to 3.1%)	0.7% (1.2 to 0.5%)	-15.3% (-12.7 to -15.4%)
Chad	-1.6% (-0.9 to -1.0%)	-0.6% (0.2 to -3.0%)	-3.6% (-2.7 to -5.5%)	-6.9% (-3.7 to -14.1%)	0.0% (0 to -0.8%)	-5.2% (-2.3 to -9.8%)	-6.6% (-4.3 to -11.2%)	-37.4% (-38.1 to -27.3%)	-4.7% (-8.1 to -3.8%)	1.6% (1.1 to 1.6%)	0.8% (0.9 to 0.6%)	-15.5% (-13.4 to -15.0%)
Yemen	-6.3% (-3.1 to -9.4%)	-9.7% (-5.9 to -15.5%)	-28.2% (-25.2 to -30.7%)	-15.4% (-10.5 to -22.3%)	-0.8% (0 to -1.5%)	-6.7% (-2.9 to -11.3%)	-5.1% (-3.2 to -7.7%)	-10.0% (-9.2 to -10.6%)	-3.5% (-2.2 to -4.2%)	0.3% (0.4 to 0.3%)	0.2% (1.2 to -1.0%)	-8.8% (-8.7 to -8.7%)
Eritrea	-0.8% (-0.6 to -1.2%)	-20.9% (-11.9 to -34.8%)	-6.1% (-5.0 to -7.0%)	-10.2% (-7.0 to -14.2%)	-1.3% (0 to -3.3%)	-6.4% (-3.1 to -9.9%)	-10.1% (-8.9 to -12.6%)	-36.5% (-35.3 to -29.2%)	-9.6% (-8.5 to -8.6%)	1.3% (1.5 to 1.1%)	-0.7% (-0.3 to -1.1%)	-9.8% (-9.3 to -8.7%)
Sierra Leone	-0.5% (-0.4 to -0.4%)	-20.6% (-13.1 to -31.8%)	-4.8% (-4.3 to -6.1%)	-3.7% (-2.2 to -8.9%)	-8.3% (0 to -17.1%)	-4.6% (-2.1 to -7.1%)	-5.4% (-4.5 to -7.2%)	-20.3% (-19.7 to -10.8%)	-24.5% (-16.9 to -29.8%)	1.3% (1.3 to 1.4%)	-1.4% (-0.5 to -2.5%)	-12.3% (-9.5 to -13.5%)
Haiti	0.2% (-0.3 to 0.1%)	-1.9% (-0.6 to -3.7%)	-4.3% (-3.5 to -4.9%)	-3.2% (-2.8 to -3.0%)	-0.2% (0 to -0.2%)	-5.1% (-2.1 to -9.1%)	-5.1% (-4.3 to -6.7%)	-10.9% (-8.1 to -11.6%)	-12.9% (-10.2 to -15.6%)	0.4% (-0.3 to 0.5%)	-4.3% (-3.4 to -5.1%)	-4.2% (-2.9 to -5.4%)
Rwanda	-0.4% (-0.2 to -0.5%)	-30.3% (-17.8 to -51.4%)	-1.1% (-0.7 to -0.9%)	-8.6% (-6.5 to -11.6%)	-0.8% (0 to -2.1%)	-5.2% (-2.4 to -8.2%)	-3.7% (-3.2 to -4.7%)	-10.7% (-7.8 to -9.4%)	-3.8% (-1.8 to -4.7%)	2.3% (2.4 to 1.6%)	-1.0% (-0.8 to -1.3%)	-5.3% (-4.6 to -5.3%)
Malawi	0.3% (0.3 to 0.7%)	-15.3% (-9.6 to -23.2%)	-3.3% (-2.5 to -4.2%)	-6.3% (-3.9 to -9.9%)	-0.2% (0 to -0.8%)	-5.2% (-2.2 to -8.6%)	-4.6% (-3.6 to -6.0%)	-23.5% (-17.7 to -23.7%)	-12.7% (-8.9 to -14.8%)	1.5% (1.7 to 1.3%)	-5.9% (-4.3 to -7.4%)	-8.3% (-7.6 to -8.0%)
Nigeria	-0.8% (-0.7 to -1.4%)	31.0% (28.6 to 30.8%)	-24.3% (-22.5 to -26.8%)	-6.6% (-3.0 to -13.4%)	-3.8% (0 to -11.7%)	-6.5% (-2.5 to -11.0%)	-2.2% (-1.2 to -3.6%)	-13.2% (-13.1 to -6.7%)	-17.2% (-13.2 to -17.7%)	3.5% (2.8 to 3.9%)	-1.5% (-0.4 to -3.1%)	-9.4% (-9.2 to -9.3%)

Location	Hand-washing	Low rotavirus vaccine coverage	Unsafe sanitation	Unsafe water	Zinc deficiency	Stunting	Under-weight	Wasting	Low oral rehydration solution coverage	Low weight and short gestation	Sub-optimal Breast feeding	Vitamin A deficiency
Mali	-0.8% (-0.5 to -1.5%)	-5.1% (-2.5 to -9.1%)	-3.1% (-2.8 to -3.4%)	-9.1% (-6.4 to -12.2%)	0.3% (0 to 0.5%)	-4.3% (-1.4 to -8.7%)	-6.9% (-5.4 to -9.6%)	-22.1% (-19.9 to -21.3%)	-13.9% (-9.8 to -13.9%)	3.0% (2.9 to 3.2%)	-3.0% (-1.0 to -5.3%)	-12.9% (-11.4 to -13.2%)
Angola	-1.1% (-0.5 to -1.6%)	-10.9% (-4.9 to -22.9%)	-19.0% (-18.1 to -21.1%)	-8.8% (-5.4 to -13.3%)	-10.1% (0 to -22.4%)	-9.8% (-4.6 to -13.7%)	-11.6% (-8.5 to -17.2%)	-53.9% (-45.5 to -59.1%)	-6.3% (-4.6 to -5.0%)	2.8% (3.1 to 2.3%)	-3.7% (-2.1 to -5.4%)	-16.1% (-13.8 to -17.2%)
Liberia	0.1% (0.0 to 0.2%)	-12.9% (-8.4 to -20.6%)	-7.9% (-7.0 to -8.9%)	-0.5% (1.2 to -2.6%)	2.3% (0 to 7.9%)	-6.5% (-2.8 to -11.6%)	-3.7% (-2.6 to -5.2%)	-13.1% (-11.0 to -16.7%)	-8.7% (-5.3 to -10.7%)	1.5% (1.6 to 1.2%)	-5.7% (-4.1 to -7.4%)	-10.4% (-8.6 to -12.1%)
Niger	-1.4% (-0.5 to -2.4%)	-23.0% (-12.2 to -42.8%)	-2.3% (-1.5 to -3.6%)	-7.4% (-3.8 to -13.1%)	-1.3% (0 to -4.1%)	-7.4% (-3.5 to -10.5%)	-8.2% (-6.7 to -13.1%)	-9.2% (-9.3 to -7.7%)	-19.1% (-13.1 to -19.0%)	1.4% (1.7 to 1.3%)	-1.0% (-0.7 to -1.4%)	-11.2% (-10.0 to -10.8%)
Uzbekistan	-4.4% (-1.9 to -7.4%)	-6.2% (-3.7 to -9.2%)	-6.6% (-7.3 to -5.7%)	-7.9% (-1.9 to -13.5%)	-0.2% (0 to -0.7%)	-4.7% (-1.8 to -8.3%)	-2.5% (-1.8 to -3.5%)	-20.4% (-16.9 to -22.4%)	-1.4% (-0.4 to -2.4%)	0.6% (0.6 to 0.4%)	-1.0% (-0.6 to -1.5%)	-2.8% (-2.6 to -3.6%)
Kenya	-1.1% (-0.6 to -1.9%)	-13.7% (-8.6 to -20.2%)	-7.6% (-7.8 to -8.1%)	-11.6% (-7.6 to -18.8%)	-0.2% (0 to -0.7%)	-6.8% (-2.9 to -11.5%)	-7.0% (-5.7 to -8.9%)	-26.5% (-20.8 to -31.4%)	-4.6% (-2.5 to -5.5%)	3.1% (3.0 to 3.2%)	-5.6% (-4.0 to -7.4%)	-2.9% (-2.1 to -3.2%)
Guyana	3.3% (1.6 to 4.6%)	-17.4% (-10.9 to -27.6%)	-18.4% (-17.0 to -19.3%)	1.7% (1.2 to 2.3%)	-0.7% (0 to -2.0%)	-0.9% (-0.3 to -1.8%)	-2.8% (-2.5 to -3.5%)	-24.0% (-20.9 to -22.0%)	-4.5% (-3.3 to -5.8%)	0.7% (0.4 to 0.7%)	-1.2% (-1.1 to -1.5%)	-2.8% (-2.3 to -3.7%)
Congo	-3.3% (-1.6 to -5.5%)	-27.4% (-11.8 to -63.7%)	-11.4% (-10.8 to -12.3%)	-19.4% (-13.7 to -28.2%)	-1.5% (0 to -5.2%)	-4.0% (-1.4 to -8.1%)	-5.2% (-4.1 to -6.7%)	21.2% (17.4 to 21.4%)	-6.7% (-6.0 to -7.3%)	1.1% (1.2 to 0.7%)	0.0% (0.3 to -0.6%)	-11.6% (-13.0 to -10.7%)
Gabon	-5.3% (-3.0 to -7.4%)	-0.1% (0.5 to -1.9%)	-19.1% (-18.0 to -19.4%)	-32.4% (-21.4 to -41.2%)	-0.3% (0 to -1.3%)	-5.4% (-1.9 to -10.5%)	-2.7% (-1.9 to -3.7%)	-8.5% (-4.4 to -12.6%)	-3.7% (-2.4 to -4.9%)	1.3% (1.9 to 0.8%)	1.0% (1.1 to 0.6%)	-8.9% (-5.8 to -11.8%)
Turkey	-4.2% (-1.7 to -7.4%)	-4.5% (-1.4 to -9.4%)	-18.0% (-15.9 to -19.8%)	-13.9% (-9.1 to -19.5%)	-0.1% (0 to -0.2%)	-2.1% (-0.7 to -4.2%)	-1.6% (-1.3 to -2.0%)	-6.3% (-3.1 to -8.8%)	-2.3% (-1.7 to -2.7%)	3.8% (3.6 to 3.1%)	-1.8% (-1.0 to -2.6%)	-5.9% (-5.4 to -5.9%)
Mexico	-3.1% (-1.2 to -5.0%)	-12.7% (-8.0 to -19.7%)	-14.0% (-11.9 to -16.2%)	-16.7% (-8.5 to -25.9%)	0.1% (0 to 0.0%)	-2.0% (-0.8 to -3.6%)	-1.3% (-1.1 to -1.5%)	-17.2% (-13.1 to -18.7%)	-1.3% (-0.9 to -1.5%)	0.8% (0.8 to 0.7%)	1.0% (0.8 to 1.2%)	-0.6% (-0.5 to -0.8%)
Suriname	-3.4% (-1.4 to -5.6%)	-0.1% (-0.2 to 0.1%)	-16.5% (-14.0 to -19.0%)	-7.2% (-5.1 to -9.8%)	-0.1% (0 to -0.3%)	-1.1% (-0.3 to -2.4%)	-1.2% (-0.9 to -1.7%)	-9.0% (-7.0 to -10.7%)	-3.9% (-2.1 to -4.0%)	1.1% (0.8 to 1.0%)	0.4% (0.1 to 0.6%)	-1.8% (-1.4 to -2.5%)
Botswana	-7.5% (-3.8 to -10.9%)	-5.8% (-3.4 to -10.6%)	-21.8% (-20.8 to -21.3%)	-39.2% (-27.8 to -50.2%)	-0.3% (0 to -1.0%)	-2.8% (-1.0 to -6.2%)	-3.0% (-2.2 to -4.0%)	-30.1% (-27.0 to -29.8%)	-2.6% (-1.3 to -2.0%)	1.7% (2.0 to 1.4%)	1.5% (1.7 to 1.2%)	-10.1% (-8.2 to -12.1%)
Azerbaijan	-5.1% (-2.1 to -8.2%)	2.5% (3.0 to 1.5%)	-10.8% (-10.4 to -10.8%)	-18.0% (-7.8 to -27.5%)	-1.1% (0 to -2.8%)	-1.8% (-0.7 to -3.6%)	-1.3% (-1.0 to -1.7%)	-11.9% (-7.8 to -12.7%)	-4.6% (-3.2 to -4.1%)	-0.2% (0.0 to -0.3%)	-0.1% (0.4 to -0.2%)	-2.1% (-1.7 to -2.6%)

Location	Hand-washing	Low rotavirus vaccine coverage	Unsafe sanitation	Unsafe water	Zinc deficiency	Stunting	Under-weight	Wasting	Low oral rehydration solution coverage	Low weight and short gestation	Sub-optimal Breast feeding	Vitamin A deficiency
Ecuador	-4.8% (-2.0 to -7.3%)	-5.3% (-3.5 to -7.9%)	-19.2% (-17.6 to -21.0%)	-16.8% (-9.2 to -24.4%)	-1.4% (0 to -4.2%)	-3.1% (-1.3 to -5.1%)	-2.5% (-2.2 to -3.0%)	-12.1% (-9.7 to -11.7%)	-3.9% (-3.7 to -4.3%)	0.5% (0.4 to 0.5%)	-1.6% (-1.5 to -1.5%)	-2.8% (-2.5 to -3.2%)
The Gambia	-1.1% (-0.7 to -1.6%)	-7.1% (-4.2 to -11.0%)	-12.7% (-12.0 to -14.1%)	-14.4% (-10.9 to -21.2%)	-5.6% (0 to -17.8%)	-6.4% (-2.4 to -11.9%)	-6.2% (-4.7 to -8.6%)	-6.4% (-1.1 to -15.2%)	-10.2% (-8.8 to -11.5%)	1.8% (2.0 to 1.7%)	-1.6% (-0.7 to -2.5%)	-14.0% (-12.5 to -14.5%)
Cote d'Ivoire	-1.5% (-0.7 to -2.1%)	-10.3% (-6.6 to -15.4%)	-9.2% (-8.5 to -9.9%)	-10.7% (-7.1 to -15.5%)	-1.0% (0 to -3.7%)	-6.1% (-2.7 to -11.7%)	-3.1% (-2.4 to -4.0%)	-22.1% (-18.7 to -21.5%)	-2.3% (2.8 to 0.9%)	1.3% (1.5 to 1.2%)	-3.2% (-2.3 to -4.5%)	-18.6% (-16.0 to -20.4%)
Turkmenistan	-2.9% (-1.3 to -4.8%)	0.0% (0.0 to 0.0%)	-9.1% (-9.6 to -7.9%)	-8.7% (-4.8 to -12.6%)	-0.8% (0 to -2.5%)	-3.1% (-1.2 to -5.6%)	-2.4% (-1.9 to -3.2%)	-18.5% (-15.0 to -20.5%)	-2.7% (-1.3 to -4.5%)	0.7% (0.7 to 0.8%)	-3.9% (-2.5 to -4.9%)	-2.0% (-1.8 to -2.4%)
Peru	-4.7% (-1.9 to -7.6%)	-14.5% (-11.1 to -18.4%)	-8.4% (-7.6 to -7.9%)	-17.3% (-9.5 to -25.2%)	-3.2% (0 to -8.8%)	-6.4% (-2.5 to -11.3%)	-1.5% (-1.1 to -2.0%)	-13.2% (-7.6 to -18.2%)	-5.2% (-3.7 to -6.2%)	0.4% (0.4 to 0.3%)	-4.6% (-3.5 to -5.9%)	-4.7% (-4.6 to -4.7%)
Swaziland	-6.7% (-3.2 to -10.1%)	-21.3% (-13.1 to -37.4%)	-5.6% (-4.1 to -6.9%)	-15.6% (-10.5 to -19.6%)	0.6% (0 to 1.7%)	-4.8% (-1.7 to -8.8%)	-1.7% (-1.2 to -2.4%)	-6.9% (-3.6 to -10.3%)	-4.4% (-2.8 to -6.0%)	0.7% (1.2 to 0.6%)	-1.9% (-1.4 to -2.2%)	-6.2% (-4.6 to -8.5%)
Lesotho	-0.5% (-0.4 to -0.7%)	-12.1% (-7.7 to -17.2%)	-4.8% (-3.9 to -6.9%)	-7.3% (-4.9 to -12.3%)	-0.6% (0 to -1.4%)	-3.5% (-1.6 to -4.9%)	-3.4% (-3.0 to -4.2%)	-82.3% (-63.7 to -86.9%)	-5.5% (-3.9 to -6.4%)	1.2% (1.4 to 1.0%)	-5.4% (-3.7 to -7.2%)	-10.9% (-8.8 to -11.7%)
Indonesia	-0.8% (-0.4 to -1.6%)	-1.3% (-0.5 to -3.2%)	-28.0% (-25.7 to -29.7%)	-7.6% (-4.7 to -11.8%)	-0.7% (0 to -2.0%)	-3.7% (-1.5 to -6.4%)	-3.9% (-3.3 to -4.7%)	-15.1% (-13.2 to -16.4%)	-2.7% (-1.9 to -3.8%)	1.0% (1.0 to 0.9%)	-0.3% (0.0 to -0.7%)	-5.5% (-4.8 to -5.9%)
Democratic Republic of the Congo	0.0% (-0.1 to 0.3%)	-8.0% (13.1 to -42.6%)	-6.6% (-6.2 to -6.9%)	-1.5% (-1.1 to -3.8%)	-1.4% (0 to -3.3%)	-2.0% (-1.0 to -2.0%)	-3.9% (-4.0 to -4.1%)	9.9% (5.5 to 22.0%)	-6.0% (-6.6 to -7.2%)	1.6% (1.5 to 1.3%)	-3.5% (-2.5 to -4.3%)	-4.2% (-3.5 to -5.3%)
Bhutan	-10.7% (-4.8 to -16.2%)	0.4% (0.2 to 1.0%)	-17.6% (-16.5 to -18.5%)	-28.7% (-20.5 to -36.0%)	-0.9% (0 to -2.9%)	-5.9% (-2.6 to -9.4%)	-5.1% (-4.3 to -6.4%)	-12.1% (-10.2 to -9.1%)	-3.1% (0.1 to -5.9%)	1.5% (1.5 to 1.5%)	-0.7% (-0.5 to -0.8%)	-4.0% (-3.6 to -3.6%)
Kiribati	-7.0% (-3.2 to -11.5%)	-8.8% (-5.8 to -13.5%)	-12.0% (-11.8 to -11.6%)	-13.7% (-9.8 to -19.0%)	0.0% (0 to -0.1%)	-2.7% (-1.2 to -4.3%)	-2.1% (-1.6 to -2.7%)	-17.3% (-15.9 to -12.4%)	-1.9% (-2.7 to -2.7%)	0.4% (0.3 to 0.4%)	-0.7% (-0.5 to -1.1%)	-2.8% (-2.5 to -2.8%)
Brazil	-8.4% (-3.8 to -13.1%)	-11.1% (-6.9 to -16.6%)	-11.3% (-10.5 to -12.1%)	-26.1% (-15.7 to -36.3%)	-0.1% (0 to -0.5%)	-1.3% (-0.5 to -2.6%)	-0.8% (-0.7 to -1.0%)	-9.8% (-6.9 to -11.4%)	-12.8% (-10.0 to -14.3%)	1.1% (1.1 to 1.1%)	-2.7% (-2.4 to -2.9%)	0.5% (0.4 to 0.6%)
Cambodia	-7.1% (-3.3 to -10.1%)	1.7% (8.0 to -10.2%)	-18.9% (-17.4 to -19.8%)	-13.6% (-9.8 to -17.8%)	-1.1% (0 to -2.7%)	-6.4% (-3.0 to -10.3%)	-9.5% (-7.2 to -14.4%)	-22.4% (-20.2 to -23.1%)	-7.4% (-4.9 to -8.2%)	0.8% (0.7 to 0.9%)	-4.0% (-2.3 to -5.7%)	-5.6% (-4.2 to -7.4%)
Namibia	-9.0% (-4.6 to -13.5%)	-10.5% (-6.5 to -15.6%)	-14.0% (-11.5 to -16.1%)	-22.3% (-14.9 to -31.3%)	0.0% (0 to 0.2%)	-4.2% (-1.5 to -8.3%)	-4.6% (-3.6 to -6.1%)	-14.4% (-13.2 to -16.0%)	-7.9% (-5.2 to -11.0%)	1.6% (1.9 to 1.2%)	-3.3% (-2.0 to -4.7%)	-3.1% (-2.5 to -4.0%)

Location	Hand-washing	Low rotavirus vaccine coverage	Unsafe sanitation	Unsafe water	Zinc deficiency	Stunting	Under-weight	Wasting	Low oral rehydration solution coverage	Low weight and short gestation	Sub-optimal Breast feeding	Vitamin A deficiency
Dominican Republic	-2.1% (-1.1 to -3.5%)	-5.4% (-3.6 to -7.3%)	-18.8% (-16.7 to -20.2%)	-2.4% (-2.4 to -4.8%)	-1.1% (0 to -2.6%)	-1.4% (-0.5 to -2.6%)	-1.1% (-0.9 to -1.5%)	-3.2% (-2.0 to -3.5%)	-8.4% (-6.8 to -9.3%)	0.8% (0.6 to 0.9%)	0.4% (0.4 to 0.3%)	-3.1% (-2.8 to -3.7%)
Myanmar	-2.1% (-0.8 to -3.1%)	0.9% (7.7 to -12.6%)	-17.7% (-15.8 to -20.3%)	-9.4% (-6.7 to -13.7%)	-3.8% (0 to -8.2%)	-4.8% (-1.7 to -9.2%)	-6.2% (-4.9 to -8.1%)	-22.0% (-17.1 to -24.5%)	-8.6% (-6.4 to -10.2%)	0.7% (0.8 to 0.8%)	-3.3% (-1.8 to -5.1%)	-7.1% (-5.9 to -6.9%)
Uganda	-1.6% (-0.9 to -2.4%)	-6.7% (2.7 to -24.7%)	-3.4% (-3.2 to -3.4%)	-14.4% (-10.4 to -19.6%)	-0.8% (0 to -2.7%)	-8.5% (-3.1 to -15.0%)	-5.6% (-3.9 to -7.8%)	-18.6% (-14.2 to -22.8%)	-9.3% (-9.4 to -8.5%)	1.1% (1.1 to 0.9%)	-1.1% (0.0 to -2.3%)	-10.4% (-8.2 to -12.8%)
El Salvador	-6.4% (-3.0 to -9.9%)	-12.4% (-8.0 to -18.3%)	-10.7% (-9.5 to -11.9%)	-19.1% (-13.0 to -24.4%)	-0.2% (0 to -0.7%)	-2.8% (-1.1 to -4.8%)	-1.8% (-1.5 to -2.2%)	-17.7% (-14.5 to -18.9%)	-5.2% (-3.6 to -6.5%)	0.6% (0.7 to 0.6%)	-3.5% (-2.6 to -4.5%)	-7.4% (-5.5 to -8.9%)
Honduras	-3.1% (-1.2 to -5.2%)	-13.3% (-8.6 to -19.6%)	-17.8% (-16.7 to -18.7%)	-14.9% (-8.0 to -22.7%)	-0.3% (0 to -1.1%)	-5.4% (-2.2 to -8.7%)	-3.8% (-3.4 to -4.6%)	-16.8% (-12.3 to -17.8%)	-7.4% (-5.2 to -8.7%)	0.7% (0.8 to 0.6%)	-0.5% (-0.2 to -1.3%)	-4.0% (-3.5 to -4.6%)
Morocco	-7.2% (-3.5 to -11.0%)	-16.6% (-10.3 to -24.8%)	-18.3% (-16.3 to -20.1%)	-20.4% (-14.0 to -27.1%)	-0.1% (0 to -0.4%)	-3.2% (-1.3 to -6.1%)	-1.9% (-1.4 to -2.6%)	-11.2% (-8.4 to -13.5%)	-6.3% (-4.8 to -7.7%)	2.1% (2.5 to 1.7%)	1.3% (1.2 to 1.5%)	-3.1% (-3.4 to -2.7%)
Tajikistan	-0.4% (-0.1 to 0.2%)	-12.9% (-8.0 to -19.8%)	-1.7% (-2.4 to -1.3%)	-4.1% (-1.3 to -5.4%)	0.1% (0 to 0.3%)	-3.2% (-1.3 to -5.8%)	-2.4% (-1.8 to -3.4%)	177.8% (119.6 to 218.7%)	-14.0% (-8.4 to -18.9%)	-0.1% (-0.2 to -0.1%)	-1.2% (-0.5 to -2.4%)	-2.0% (-1.3 to -3.0%)
South Sudan	-0.2% (0.1 to -0.2%)	1.3% (1.0 to 0.5%)	0.4% (0.9 to 0.2%)	-3.1% (-0.6 to -6.3%)	0.0% (0 to -0.2%)	-6.4% (-2.9 to -10.7%)	-9.7% (-7.3 to -13.7%)	-23.5% (-22.2 to -27.2%)	-2.9% (-2.3 to -6.6%)	0.9% (1.0 to 1.1%)	-5.0% (-3.2 to -6.9%)	-10.0% (-10.2 to -8.6%)
South Africa	-5.5% (-2.8 to -8.9%)	-10.2% (-6.1 to -15.1%)	-15.6% (-13.7 to -17.2%)	-17.7% (-10.2 to -27.5%)	-0.1% (0 to -0.4%)	-2.8% (-1.0 to -5.3%)	-2.2% (-1.8 to -2.8%)	-14.4% (-10.6 to -16.1%)	-4.2% (-2.5 to -5.1%)	1.2% (1.5 to 1.1%)	-0.5% (-0.7 to -0.1%)	-2.3% (-1.8 to -3.0%)
Burundi	-0.3% (-0.2 to -0.2%)	-23.2% (-13.7 to -36.7%)	-2.6% (-2.3 to -3.3%)	-4.0% (-1.5 to -8.2%)	7.5% (0 to 20.3%)	0.8% (0.0 to 3.9%)	-2.3% (-2.6 to -2.6%)	-11.8% (-11.1 to -5.0%)	-5.1% (-3.9 to -2.8%)	1.2% (1.5 to 1.0%)	-1.6% (-0.8 to -2.6%)	-4.3% (-3.4 to -5.4%)
Benin	-2.0% (-0.9 to -2.7%)	-0.5% (0.2 to -2.4%)	-6.2% (-5.2 to -7.6%)	-15.5% (-11.4 to -23.7%)	-1.5% (0 to -4.7%)	-3.3% (-1.6 to -4.6%)	-4.6% (-3.9 to -6.3%)	-15.1% (-17.5 to -6.1%)	-16.0% (-12.0 to -17.4%)	1.2% (0.9 to 1.2%)	-2.3% (-1.7 to -2.8%)	-15.5% (-15.0 to -14.1%)
Timor-Leste	-5.2% (-2.4 to -8.5%)	0.3% (0.2 to 1.1%)	-9.4% (-8.6 to -9.8%)	-16.1% (-12.2 to -20.9%)	-0.8% (0 to -2.8%)	-6.7% (-3.0 to -11.5%)	-11.5% (-7.6 to -18.7%)	-22.2% (-22.0 to -23.9%)	-13.2% (-9.7 to -16.4%)	0.2% (0.3 to 0.2%)	-0.6% (-0.5 to -1.2%)	-5.9% (-5.3 to -5.3%)
India	-3.2% (-1.6 to -4.5%)	-1.0% (-0.8 to -1.0%)	-21.8% (-18.9 to -23.8%)	-6.2% (-2.9 to -11.8%)	-0.8% (0 to -1.9%)	-6.7% (-2.9 to -11.0%)	-9.9% (-8.3 to -12.0%)	-17.3% (-15.6 to -18.4%)	-15.3% (-11.1 to -16.9%)	2.3% (2.4 to 2.2%)	-0.8% (-0.2 to -1.1%)	-1.4% (-1.1 to -1.5%)
Djibouti	-1.8% (-1.1 to -2.5%)	-8.5% (-5.2 to -13.5%)	-9.2% (-8.4 to -9.7%)	-13.6% (-9.3 to -19.0%)	-1.7% (0 to -4.3%)	-2.8% (-0.9 to -4.7%)	-6.3% (-4.5 to -9.0%)	-21.0% (-19.4 to -23.9%)	-0.1% (0.0 to 0.3%)	1.3% (1.6 to 1.1%)	0.2% (0.6 to 0.0%)	-5.2% (-4.3 to -6.4%)

Location	Hand-washing	Low rotavirus vaccine coverage	Unsafe sanitation	Unsafe water	Zinc deficiency	Stunting	Under-weight	Wasting	Low oral rehydration solution coverage	Low weight and short gestation	Sub-optimal Breast feeding	Vitamin A deficiency
Cape Verde	-3.2% (-1.5 to -4.7%)	-0.2% (0.0 to -1.0%)	-15.6% (-15.1 to -16.1%)	-17.3% (-12.1 to -23.4%)	-0.4% (0 to -1.4%)	-3.5% (-1.4 to -6.2%)	-2.9% (-2.4 to -3.8%)	-16.4% (-13.9 to -14.6%)	-2.8% (-2.8 to -3.2%)	0.9% (1.2 to 0.8%)	-1.6% (-1.3 to -1.9%)	-0.2% (-0.2 to -0.3%)
Albania	-1.5% (-0.5 to -2.4%)	0.0% (-0.3 to 0.1%)	-11.6% (-9.3 to -13.6%)	-12.1% (-7.0 to -16.7%)	-0.2% (0 to -0.7%)	-2.9% (-1.1 to -5.2%)	-1.7% (-1.2 to -2.4%)	-12.1% (-10.7 to -13.8%)	-8.5% (-6.7 to -8.8%)	0.7% (0.7 to 0.7%)	-0.6% (-0.1 to -1.2%)	-5.0% (-5.0 to -4.4%)
Marshall Islands	-8.6% (-3.8 to -13.7%)	-5.0% (-3.3 to -7.9%)	-22.6% (-20.7 to -23.9%)	-21.2% (-14.6 to -28.1%)	-0.2% (0 to -1.0%)	-1.9% (-0.6 to -3.7%)	-1.9% (-1.3 to -2.6%)	-17.5% (-14.7 to -20.1%)	-4.6% (-3.2 to -4.7%)	0.4% (0.4 to 0.3%)	-0.3% (-0.6 to -0.4%)	-9.1% (-9.5 to -8.7%)
Argentina	-8.3% (-3.6 to -13.3%)	-6.1% (-5.2 to -6.4%)	-26.6% (-22.1 to -30.5%)	-39.7% (-21.5 to -58.1%)	0.0% (0 to -0.1%)	-0.7% (-0.3 to -1.1%)	-0.9% (-0.8 to -1.1%)	17.7% (10.5 to 32.6%)	-4.6% (-3.8 to -5.6%)	1.5% (1.4 to 1.5%)	0.2% (0.0 to 0.4%)	-4.9% (-4.1 to -6.4%)
Lebanon	-2.9% (-1.2 to -5.0%)	0.1% (0.4 to -0.3%)	-34.8% (-27.5 to -41.5%)	-4.7% (-2.2 to -8.2%)	0.1% (0 to 0.2%)	-2.5% (-0.7 to -4.6%)	-1.0% (-0.7 to -1.6%)	-16.0% (-10.8 to -21.0%)	-0.3% (-0.4 to -0.1%)	3.3% (3.6 to 3.1%)	-0.1% (0.2 to -1.4%)	-5.7% (-4.1 to -7.5%)
Saint Lucia	-3.5% (-1.6 to -5.8%)	0.1% (0.0 to 0.5%)	-21.6% (-19.7 to -23.0%)	-9.2% (-6.7 to -12.3%)	-0.2% (0 to -0.7%)	-1.1% (-0.3 to -2.3%)	-1.0% (-0.6 to -1.5%)	-10.6% (-6.6 to -15.8%)	-0.5% (-0.2 to -0.6%)	0.5% (0.5 to 0.5%)	0.2% (0.2 to -0.1%)	-2.2% (-1.9 to -2.5%)
Oman	-7.5% (-3.1 to -11.4%)	11.3% (2.9 to 36.8%)	-19.5% (-14.1 to -24.6%)	-29.6% (-19.1 to -39.4%)	-0.8% (0 to -2.6%)	-3.0% (-1.1 to -5.8%)	-3.7% (-3.0 to -4.9%)	-19.7% (-17.3 to -21.0%)	-0.5% (-0.5 to -0.5%)	3.4% (3.8 to 2.7%)	0.1% (0.4 to -0.6%)	-6.5% (-4.9 to -8.2%)
Moldova	-3.8% (-1.6 to -6.2%)	-4.7% (-3.1 to -7.2%)	-4.0% (-4.0 to -3.1%)	-11.4% (-7.3 to -14.6%)	0.0% (0 to 0.0%)	-1.0% (-0.3 to -2.4%)	-0.5% (-0.3 to -0.8%)	-11.2% (-6.4 to -15.6%)	-5.8% (-4.9 to -6.6%)	1.0% (0.9 to 0.9%)	0.1% (0.1 to 0.0%)	-0.1% (-0.1 to -0.3%)
Panama	-11.2% (-4.8 to -18.2%)	-16.1% (-10.4 to -23.9%)	-28.0% (-25.4 to -31.4%)	-36.8% (-22.0 to -52.9%)	-1.0% (0 to -4.0%)	-0.7% (-0.4 to -1.0%)	-1.0% (-0.8 to -1.2%)	-27.5% (-19.0 to -30.6%)	-3.5% (-2.9 to -4.8%)	0.3% (0.4 to 0.2%)	0.4% (0.8 to -0.1%)	-0.5% (-0.8 to -0.2%)
Jordan	-3.3% (-1.2 to -5.1%)	-30.5% (-18.2 to -51.0%)	-12.2% (-8.9 to -15.7%)	-20.1% (-8.4 to -36.8%)	-0.6% (0 to -1.6%)	-2.1% (-0.7 to -4.7%)	-0.8% (-0.5 to -1.2%)	-9.2% (-5.2 to -15.3%)	0.9% (0.9 to 0.7%)	5.2% (5.5 to 5.6%)	-1.3% (-0.8 to -1.6%)	-6.9% (-6.2 to -6.7%)
Thailand	-2.2% (-0.8 to -3.5%)	26.6% (17.7 to 40.8%)	-16.8% (-12.2 to -21.6%)	-9.4% (-5.5 to -13.6%)	-0.3% (0 to -1.1%)	-2.3% (-1.0 to -3.9%)	-2.7% (-2.4 to -3.1%)	-11.0% (-10.8 to -6.9%)	-8.1% (-6.0 to -9.6%)	1.4% (1.5 to 1.1%)	0.6% (0.7 to 0.8%)	-6.1% (-4.7 to -7.6%)
Sri Lanka	-6.7% (-2.9 to -10.7%)	-0.1% (0.0 to -0.2%)	-21.7% (-20.6 to -22.3%)	-16.9% (-12.4 to -22.2%)	-0.8% (0 to -2.6%)	-3.6% (-1.5 to -6.0%)	-4.8% (-4.2 to -5.9%)	-17.7% (-17.1 to -15.9%)	-1.3% (-1.1 to -1.8%)	0.8% (0.8 to 0.6%)	-2.8% (-2.2 to -3.3%)	-7.6% (-6.3 to -8.2%)
Mauritius	-1.0% (-0.3 to -1.9%)	-12.5% (-7.8 to -19.0%)	-20.5% (-16.9 to -23.5%)	-15.6% (-4.4 to -31.9%)	-0.5% (0 to -1.8%)	-1.5% (-0.7 to -2.7%)	-2.2% (-2.0 to -2.5%)	-14.4% (-15.4 to -6.9%)	-2.3% (-2.0 to -3.7%)	0.4% (0.4 to 0.2%)	-0.7% (-0.5 to -0.7%)	-2.6% (-2.0 to -3.1%)
Georgia	-1.3% (-0.5 to -2.3%)	-4.1% (-2.6 to -6.0%)	-4.5% (-3.6 to -5.2%)	-1.9% (-0.2 to -3.8%)	-0.1% (0 to -0.3%)	-0.4% (-0.2 to -0.6%)	-0.2% (-0.1 to -0.2%)	-6.2% (-3.4 to -8.1%)	-2.3% (-2.6 to -2.5%)	1.2% (1.2 to 1.2%)	-1.3% (-1.0 to -1.4%)	-1.1% (-0.9 to -1.3%)

Location	Hand-washing	Low rotavirus vaccine coverage	Unsafe sanitation	Unsafe water	Zinc deficiency	Stunting	Under-weight	Wasting	Low oral rehydration solution coverage	Low weight and short gestation	Sub-optimal Breast feeding	Vitamin A deficiency
Tunisia	-3.8% (-1.6 to -6.3%)	3.3% (2.0 to 5.2%)	-15.0% (-11.6 to -17.9%)	-13.1% (-7.6 to -17.4%)	-0.3% (0 to -0.9%)	-1.8% (-0.7 to -3.3%)	-0.9% (-0.7 to -1.1%)	-32.4% (-27.3 to -34.0%)	-0.3% (0.0 to -0.3%)	1.6% (1.8 to 1.4%)	0.7% (0.7 to 0.9%)	-2.5% (-1.9 to -3.3%)
Mongolia	-0.5% (-0.3 to -0.3%)	15.9% (16.4 to 12.2%)	-6.9% (-6.5 to -7.7%)	-0.8% (-0.2 to -1.4%)	-0.2% (0 to 0.0%)	-5.8% (-2.0 to -11.6%)	-3.4% (-2.5 to -4.7%)	-17.1% (-11.0 to -23.9%)	1.2% (1.2 to 0.9%)	0.9% (0.7 to 1.0%)	0.1% (0.1 to 0.0%)	-4.7% (-3.8 to -5.4%)
Libya	0.2% (0.0 to 0.5%)	-10.2% (-6.5 to -14.7%)	-28.1% (-25.2 to -30.8%)	1.5% (1.0 to -0.6%)	0.3% (0 to 0.9%)	-1.4% (-0.5 to -2.1%)	-0.8% (-0.6 to -0.9%)	-5.0% (-5.8 to 0.2%)	-0.3% (0.0 to -0.1%)	1.6% (1.8 to 1.2%)	-0.9% (-0.3 to -1.4%)	-4.0% (-3.1 to -5.1%)
Vanuatu	-5.1% (-2.0 to -9.9%)	0.1% (-0.1 to 0.9%)	-9.8% (-9.4 to -10.3%)	-11.7% (-7.6 to -19.4%)	-0.4% (0 to -1.2%)	-1.4% (-0.6 to -3.5%)	-1.8% (-1.3 to -2.7%)	-24.1% (-18.8 to -25.8%)	-3.2% (-2.7 to -4.2%)	0.2% (0.2 to 0.2%)	-0.2% (0.0 to -0.3%)	-5.1% (-4.5 to -4.8%)
Saint Vincent and the Grenadines	-3.9% (-1.7 to -6.0%)	-0.1% (-0.1 to -0.1%)	-18.2% (-17.6 to -18.8%)	-8.1% (-4.9 to -12.3%)	-0.3% (0 to -1.3%)	-1.3% (-0.4 to -2.8%)	-1.3% (-0.8 to -1.8%)	-9.0% (-5.5 to -12.1%)	-3.9% (-2.6 to -5.0%)	0.5% (0.4 to 0.4%)	-0.4% (-0.3 to -0.6%)	-2.5% (-2.0 to -3.2%)
Algeria	-3.0% (-1.2 to -5.3%)	0.1% (0.3 to -0.5%)	-18.8% (-16.3 to -21.6%)	-8.9% (-3.7 to -16.5%)	-1.7% (0 to -4.5%)	-5.1% (-1.8 to -9.8%)	-5.2% (-4.4 to -6.6%)	-10.0% (-8.8 to -7.0%)	-0.4% (-0.3 to -0.6%)	0.9% (0.9 to 0.9%)	0.8% (0.9 to 0.7%)	-6.3% (-5.0 to -7.9%)
Federated States of Micronesia	-5.3% (-2.2 to -8.8%)	-4.3% (-2.9 to -6.6%)	-16.3% (-15.0 to -17.4%)	-15.9% (-10.8 to -20.2%)	-0.1% (0 to -0.2%)	-1.3% (-0.5 to -2.5%)	-1.4% (-1.0 to -2.0%)	-13.2% (-11.0 to -14.8%)	-3.4% (-2.4 to -4.4%)	0.6% (0.5 to 0.4%)	-0.5% (-0.3 to -0.7%)	-4.0% (-4.1 to -3.9%)
Vietnam	-1.8% (-0.9 to -2.9%)	-0.2% (0.3 to 2.7%)	-22.3% (-20.5 to -23.9%)	-11.7% (-8.2 to -16.1%)	-1.6% (0 to -4.3%)	-5.9% (-2.5 to -9.5%)	-6.2% (-5.3 to -7.6%)	-20.2% (-18.0 to -20.2%)	-5.8% (-4.0 to -6.7%)	0.7% (0.7 to 1.1%)	-0.4% (-0.2 to -0.4%)	-3.2% (-2.6 to -4.0%)
Maldives	-5.8% (-2.6 to -8.5%)	0.2% (0.2 to 0.8%)	-24.6% (-22.2 to -27.0%)	-11.1% (-8.1 to -15.0%)	-1.7% (0 to -3.7%)	-6.0% (-2.5 to -9.8%)	-10.4% (-8.6 to -13.3%)	-28.9% (-27.3 to -27.0%)	-6.6% (-4.4 to -7.1%)	0.7% (0.7 to 0.6%)	-0.7% (-0.3 to -1.0%)	-5.3% (-4.3 to -6.6%)
Syria	-2.6% (-1.1 to -4.3%)	0.0% (0.2 to -0.4%)	-14.2% (-12.2 to -15.8%)	-12.9% (-6.6 to -17.9%)	-0.1% (0 to -0.2%)	-2.7% (-1.1 to -4.8%)	-2.6% (-2.1 to -3.3%)	-24.2% (-22.4 to -23.4%)	-3.0% (-2.3 to -3.6%)	0.9% (1.0 to 0.7%)	-0.5% (-0.1 to -1.2%)	-4.9% (-3.8 to -5.6%)
Solomon Islands	-8.2% (-3.3 to -13.6%)	0.1% (-0.1 to 0.8%)	-9.0% (-8.8 to -8.2%)	-22.9% (-15.5 to -31.3%)	-2.4% (0 to -5.4%)	-2.6% (-0.8 to -5.2%)	-3.2% (-2.4 to -4.2%)	-24.8% (-21.7 to -25.0%)	-6.4% (-5.0 to -6.8%)	0.4% (0.4 to 0.3%)	-0.1% (-0.2 to 0.1%)	-4.8% (-3.8 to -5.2%)
Belize	0.3% (0.0 to 0.6%)	-2.7% (-1.8 to -3.6%)	-24.3% (-23.3 to -24.3%)	-1.9% (-1.1 to -3.7%)	-0.5% (0 to -1.6%)	-3.5% (-1.0 to -7.5%)	-0.9% (-0.7 to -1.1%)	-4.3% (-0.7 to -15.4%)	-4.2% (-3.5 to -5.5%)	0.7% (0.7 to 0.7%)	-3.2% (-3.4 to -3.0%)	-3.0% (-2.3 to -3.7%)
Iran	-3.1% (-1.3 to -5.1%)	3.1% (1.6 to 5.2%)	-21.2% (-17.9 to -23.8%)	-11.7% (-6.5 to -17.8%)	-0.3% (0 to -1.1%)	-3.3% (-1.3 to -6.0%)	-4.1% (-3.4 to -5.0%)	-24.7% (-22.1 to -24.1%)	-0.7% (-0.5 to -0.9%)	1.7% (1.7 to 1.7%)	-0.4% (-0.1 to -0.7%)	-1.6% (-1.1 to -2.1%)
Iraq	-2.1% (-0.8 to -3.3%)	-10.2% (-6.3 to -16.1%)	-20.5% (-16.1 to -23.8%)	-17.4% (-9.5 to -31.1%)	1.3% (0 to 4.5%)	-3.8% (-1.3 to -6.4%)	-4.3% (-3.6 to -5.3%)	-14.8% (-14.1 to -11.0%)	-4.0% (-3.0 to -4.4%)	1.7% (2.0 to 1.5%)	-0.6% (-0.1 to -0.7%)	-5.0% (-4.0 to -5.3%)

Location	Hand-washing	Low rotavirus vaccine coverage	Unsafe sanitation	Unsafe water	Zinc deficiency	Stunting	Under-weight	Wasting	Low oral rehydration solution coverage	Low weight and short gestation	Sub-optimal Breast feeding	Vitamin A deficiency
Philippines	-3.4% (-1.2 to -5.6%)	-2.6% (-1.6 to -4.3%)	-30.3% (-25.7 to -34.5%)	-10.3% (-7.0 to -16.9%)	-1.8% (0 to -4.7%)	-3.1% (-1.3 to -5.1%)	-3.8% (-3.7 to -4.4%)	-12.9% (-13.9 to -6.1%)	-8.1% (-5.8 to -11.2%)	0.6% (0.9 to 0.5%)	-0.3% (-0.3 to -0.3%)	-11.6% (-11.0 to -10.4%)
Jamaica	-2.6% (-1.3 to -4.0%)	0.1% (0.0 to 0.5%)	-10.2% (-10.0 to -10.1%)	-6.7% (-5.3 to -8.0%)	-0.2% (0 to -0.8%)	-0.8% (-0.3 to -1.3%)	-0.8% (-0.6 to -1.0%)	-16.1% (-12.4 to -16.2%)	-6.2% (-5.1 to -7.0%)	0.1% (0.1 to 0.1%)	-0.9% (-0.8 to -1.2%)	-1.4% (-1.2 to -1.9%)
China	-6.8% (-3.0 to -10.9%)	-13.7% (-8.3 to -20.0%)	-20.6% (-19.4 to -21.0%)	-22.4% (-14.2 to -29.8%)	-0.5% (0 to -1.7%)	-2.8% (-1.0 to -5.8%)	-2.7% (-1.9 to -3.8%)	-16.3% (-11.6 to -21.7%)	-6.1% (-4.1 to -7.5%)	0.8% (0.8 to 0.8%)	-1.1% (-0.8 to -1.5%)	-1.4% (-1.1 to -1.8%)
Colombia	-3.7% (-1.6 to -6.4%)	-13.5% (-8.6 to -20.1%)	-14.6% (-12.2 to -16.8%)	-11.3% (-6.2 to -16.2%)	-0.3% (0 to -0.9%)	-1.4% (-0.6 to -2.1%)	-1.3% (-1.2 to -1.6%)	-21.9% (-16.2 to -23.7%)	-5.9% (-3.9 to -7.1%)	1.4% (1.4 to 1.3%)	-4.2% (-3.3 to -5.0%)	-2.7% (-2.1 to -3.2%)
Kazakhstan	-4.5% (-1.8 to -7.6%)	0.1% (0.1 to 0.2%)	-12.1% (-11.4 to -12.7%)	-12.3% (-3.7 to -19.8%)	0.2% (0 to 0.9%)	-1.9% (-0.6 to -3.7%)	-1.2% (-0.8 to -1.8%)	-8.5% (-4.5 to -11.6%)	-10.8% (-8.3 to -12.1%)	0.5% (0.6 to 0.1%)	-2.2% (-1.6 to -2.9%)	-4.2% (-3.1 to -5.4%)
Papua New Guinea	-7.7% (-3.2 to -10.7%)	1.3% (-3.4 to 12.2%)	-9.0% (-9.0 to -9.7%)	-15.3% (-10.1 to -23.0%)	-0.1% (0 to -0.8%)	-6.0% (-2.2 to -11.0%)	-5.6% (-3.4 to -8.5%)	-38.8% (-32.4 to -45.8%)	-3.1% (-2.1 to -2.7%)	0.7% (0.5 to 1.1%)	-0.8% (-0.5 to -1.0%)	-4.1% (-3.7 to -4.7%)
Saudi Arabia	-3.8% (-1.6 to -6.1%)	-11.8% (-7.2 to -17.4%)	-25.4% (-21.6 to -28.7%)	-25.7% (-14.9 to -33.3%)	-0.1% (0 to -0.5%)	-3.5% (-1.4 to -6.5%)	-3.7% (-3.0 to -4.6%)	-18.2% (-14.0 to -20.9%)	-1.4% (-0.9 to -1.6%)	1.0% (1.1 to 0.8%)	-1.1% (-0.6 to -2.0%)	-9.3% (-7.4 to -11.2%)
Armenia	-2.5% (-1.0 to -4.1%)	-4.9% (-3.0 to -7.1%)	-10.2% (-8.5 to -12.0%)	-11.0% (-2.5 to -21.1%)	-0.4% (0 to -1.0%)	-1.4% (-0.4 to -2.9%)	-0.4% (-0.2 to -0.7%)	0.9% (1.5 to 1.6%)	-7.2% (-5.9 to -8.2%)	1.0% (1.2 to 0.8%)	-1.2% (-0.7 to -1.9%)	-0.2% (-0.1 to -0.3%)
Paraguay	-10.5% (-4.7 to -15.8%)	-7.8% (-4.7 to -11.8%)	-26.2% (-24.1 to -27.3%)	-34.0% (-21.6 to -44.2%)	0.0% (0 to 0.0%)	-2.8% (-1.0 to -6.1%)	-0.3% (-0.2 to -0.4%)	-2.1% (-0.8 to -3.2%)	2.5% (-2.3 to 6.3%)	0.8% (0.9 to 0.6%)	-4.2% (-3.0 to -5.4%)	1.5% (1.0 to 2.0%)
Kyrgyzstan	-3.5% (-1.6 to -6.5%)	-0.1% (-0.2 to -0.2%)	6.1% (5.0 to 7.1%)	-6.9% (-1.2 to -10.8%)	-0.3% (0 to -1.1%)	-2.4% (-0.8 to -4.8%)	-0.8% (-0.5 to -1.1%)	-4.4% (-3.3 to -7.4%)	-10.8% (-8.3 to -13.7%)	1.2% (1.1 to 1.3%)	-2.6% (-2.0 to -3.5%)	-1.6% (-1.4 to -2.1%)
Venezuela	-4.6% (-2.0 to -7.8%)	-11.9% (-12.3 to -8.6%)	-11.0% (-9.2 to -12.0%)	-21.9% (-12.4 to -34.2%)	-0.2% (0 to -0.9%)	-1.2% (-0.5 to -1.9%)	-0.7% (-0.7 to -0.8%)	-4.3% (-5.8 to 0.1%)	-1.9% (-1.9 to -2.1%)	0.5% (0.6 to 0.5%)	-0.5% (-0.1 to -1.1%)	-3.1% (-2.4 to -3.9%)
Macedonia	-0.7% (-0.2 to -1.3%)	0.0% (0.1 to -0.2%)	-8.4% (-6.1 to -11.1%)	-11.7% (-5.1 to -19.2%)	-0.1% (0 to -0.3%)	-0.7% (-0.2 to -1.6%)	-0.1% (-0.1 to -0.2%)	-3.1% (-2.0 to -5.0%)	-7.4% (-5.7 to -8.1%)	0.9% (1.0 to 0.8%)	0.0% (0.0 to 0.0%)	-6.1% (-4.9 to -7.1%)
France	-0.1% (0.0 to -0.1%)	-4.4% (-1.3 to -10.8%)	-3.4% (-1.9 to -5.6%)	-4.2% (-0.7 to -10.8%)	0.0% (0 to 0.3%)	-0.3% (-0.1 to -0.9%)	-0.1% (-0.1 to -0.2%)	-3.6% (-2.3 to -4.5%)	-3.3% (-2.2 to -3.2%)	0.9% (0.8 to 1.0%)	-0.4% (-0.2 to -0.7%)	-0.2% (-0.1 to -0.3%)
Taiwan	-2.6% (-1.0 to -4.6%)	-0.2% (-0.3 to -0.6%)	-20.2% (-14.5 to -26.6%)	-25.8% (-9.4 to -47.6%)	0.3% (0 to 1.0%)	-0.6% (-0.2 to -1.5%)	-0.2% (-0.2 to -0.3%)	-10.3% (-7.2 to -11.2%)	-3.4% (-3.4 to -4.1%)	-0.2% (-0.3 to -0.1%)	-1.4% (-1.1 to -1.5%)	0.6% (0.6 to 0.6%)

Location	Hand-washing	Low rotavirus vaccine coverage	Unsafe sanitation	Unsafe water	Zinc deficiency	Stunting	Under-weight	Wasting	Low oral rehydration solution coverage	Low weight and short gestation	Sub-optimal Breast feeding	Vitamin A deficiency
Serbia	-1.6% (-0.6 to -2.9%)	0.0% (0.2 to 0.0%)	-11.2% (-7.5 to -16.0%)	-13.9% (-3.6 to -26.9%)	-0.1% (0 to -0.2%)	-1.1% (-0.3 to -2.5%)	-0.3% (-0.2 to -0.5%)	-11.3% (-6.4 to -15.3%)	-2.4% (-0.8 to -2.3%)	2.2% (2.2 to 2.1%)	0.1% (0.1 to 0.2%)	-6.2% (-5.2 to -6.5%)
Qatar	-2.7% (-1.1 to -3.9%)	-32.1% (-18.8 to -50.4%)	-36.2% (-24.3 to -49.1%)	-22.1% (-9.8 to -35.6%)	0.2% (0 to -0.1%)	-2.6% (-0.8 to -4.9%)	-1.9% (-1.6 to -2.2%)	-25.1% (-20.3 to -16.9%)	0.3% (1.0 to 0.0%)	2.3% (2.5 to 2.0%)	-6.8% (-5.7 to -8.1%)	-9.0% (-6.2 to -12.2%)
Montenegro	-0.4% (-0.2 to -0.8%)	0.1% (0.2 to 0.0%)	-4.9% (-2.9 to -7.3%)	-8.5% (-3.6 to -15.3%)	0.0% (0 to 0.0%)	-0.5% (-0.1 to -1.0%)	-0.2% (-0.1 to -0.3%)	-9.1% (-5.7 to -12.9%)	-4.4% (-3.4 to -4.7%)	1.4% (1.6 to 1.3%)	0.0% (0.2 to -0.1%)	-5.0% (-3.9 to -6.3%)
South Korea	-0.8% (-0.2 to -1.6%)	-14.9% (-13.5 to -15.4%)	-7.0% (-4.1 to -10.4%)	-7.2% (-1.6 to -15.3%)	-0.2% (0 to -0.5%)	-0.2% (0.0 to -0.6%)	-0.2% (-0.1 to -0.2%)	-2.0% (-1.3 to -2.7%)	-3.9% (-2.8 to -3.9%)	1.0% (1.1 to 0.9%)	-0.1% (-0.2 to -0.2%)	-1.9% (-1.6 to -2.3%)
Lithuania	-1.7% (-0.6 to -3.0%)	0.0% (0.1 to -0.5%)	-12.7% (-9.9 to -15.4%)	-15.7% (-5.6 to -27.5%)	0.0% (0 to 0.0%)	-1.0% (-0.3 to -2.4%)	-0.3% (-0.2 to -0.5%)	-9.3% (-5.1 to -13.8%)	-6.2% (-5.0 to -5.9%)	0.6% (0.6 to 0.5%)	-0.3% (-0.2 to -0.3%)	-0.4% (-0.2 to -0.5%)
Latvia	-1.5% (-0.6 to -2.7%)	-9.3% (-5.8 to -14.2%)	-12.4% (-10.0 to -15.7%)	-13.8% (-5.5 to -24.7%)	0.0% (0 to 0.0%)	-1.0% (-0.3 to -2.1%)	-0.3% (-0.2 to -0.5%)	-9.1% (-5.6 to -12.4%)	-6.5% (-4.5 to -5.2%)	0.9% (1.0 to 0.8%)	0.1% (0.3 to 0.1%)	-0.2% (-0.1 to -0.2%)
Bahrain	-0.3% (-0.2 to -0.7%)	-22.1% (-12.8 to -35.4%)	-17.0% (-12.2 to -22.2%)	-3.5% (-2.1 to -8.8%)	0.0% (0 to 0.1%)	-2.3% (-0.8 to -4.4%)	-0.6% (-0.7 to -0.4%)	-33.1% (-26.8 to -34.6%)	-0.6% (-0.7 to -0.4%)	1.8% (1.8 to 1.7%)	-2.1% (-1.5 to -2.8%)	-3.9% (-3.1 to -4.7%)
Bulgaria	-0.9% (-0.3 to -1.7%)	0.1% (0.3 to -0.3%)	-2.0% (-1.1 to -3.0%)	-19.3% (-7.3 to -34.5%)	0.1% (0 to 0.5%)	-1.2% (-0.4 to -2.6%)	-0.5% (-0.3 to -0.8%)	-9.2% (-4.8 to -13.0%)	-5.7% (-3.3 to -5.6%)	0.6% (0.5 to 0.7%)	1.1% (0.8 to 1.4%)	-7.3% (-5.8 to -8.1%)
Poland	-0.8% (-0.3 to -1.5%)	-2.6% (-3.8 to 0.9%)	-9.9% (-6.2 to -13.8%)	-17.3% (-7.0 to -28.9%)	-0.1% (0 to -0.3%)	-0.9% (-0.3 to -1.9%)	-0.3% (-0.2 to -0.4%)	-7.8% (-4.4 to -11.5%)	-4.4% (-4.3 to -3.6%)	2.7% (2.9 to 2.5%)	-0.1% (-0.1 to -0.3%)	-5.5% (-4.3 to -6.8%)
Portugal	-0.6% (-0.2 to -1.3%)	0.1% (0.4 to -0.3%)	-13.4% (-9.8 to -16.5%)	-21.9% (-6.3 to -44.8%)	0.0% (0 to -0.1%)	-0.4% (-0.1 to -1.0%)	-0.3% (-0.2 to -0.4%)	-5.1% (-3.1 to -7.7%)	-3.2% (-2.6 to -3.2%)	1.5% (1.5 to 1.5%)	0.3% (0.1 to 0.6%)	-1.8% (-0.9 to -2.5%)
Dominica	-6.8% (-3.0 to -10.6%)	0.1% (0.0 to 0.7%)	-32.7% (-30.5 to -34.2%)	-15.0% (-11.5 to -20.7%)	-0.3% (0 to -0.9%)	-1.5% (-0.4 to -3.0%)	-1.3% (-0.8 to -1.9%)	-12.0% (-5.6 to -19.4%)	-5.2% (-3.9 to -6.3%)	-0.6% (-0.8 to -0.5%)	-0.5% (-0.2 to -0.8%)	-3.7% (-3.3 to -4.5%)
Estonia	-1.3% (-0.5 to -2.7%)	-7.0% (-5.1 to -9.2%)	-11.2% (-8.0 to -14.3%)	-13.8% (-5.1 to -24.2%)	0.3% (0 to 0.8%)	-0.9% (-0.2 to -2.4%)	-0.3% (-0.2 to -0.4%)	-8.0% (-5.2 to -11.0%)	-6.1% (-5.0 to -5.2%)	2.1% (2.2 to 1.9%)	0.1% (0.4 to -0.1%)	-0.3% (-0.2 to -0.4%)
American Samoa	-4.1% (-1.6 to -7.2%)	0.0% (-0.1 to 0.5%)	-16.1% (-11.4 to -22.3%)	-24.9% (-14.5 to -34.4%)	0.5% (0 to 1.8%)	-0.6% (-0.2 to -1.6%)	-0.4% (-0.2 to -0.6%)	-8.5% (-5.2 to -11.2%)	-3.6% (-1.3 to -4.3%)	0.4% (0.4 to 0.2%)	-0.7% (-0.6 to -0.9%)	-3.4% (-2.4 to -4.6%)
Bermuda	-0.3% (-0.2 to -0.5%)	0.1% (0.0 to 0.6%)	-6.0% (-3.8 to -8.1%)	0.7% (0.2 to 0.5%)	0.4% (0 to 1.2%)	-0.4% (-0.1 to -0.9%)	-0.1% (-0.1 to -0.2%)	-2.2% (-0.8 to -3.2%)	-4.2% (-4.3 to -5.7%)	0.3% (0.4 to 0.3%)	-0.4% (-0.6 to -0.5%)	-1.4% (-1.2 to -1.7%)

Location	Hand-washing	Low rotavirus vaccine coverage	Unsafe sanitation	Unsafe water	Zinc deficiency	Stunting	Under-weight	Wasting	Low oral rehydration solution coverage	Low weight and short gestation	Sub-optimal Breast feeding	Vitamin A deficiency
United Arab Emirates	-4.6% (-1.8 to -7.8%)	-20.7% (-12.6 to -32.5%)	-11.3% (-6.7 to -17.0%)	-23.2% (-11.0 to -36.2%)	-1.1% (0 to -2.6%)	-2.3% (-0.9 to -3.8%)	-0.7% (-0.4 to -1.1%)	-30.3% (-29.4 to -21.6%)	-0.2% (-0.5 to -0.2%)	3.5% (3.3 to 2.7%)	-1.8% (-1.5 to -2.3%)	-4.0% (-3.3 to -4.9%)
Kuwait	-0.7% (-0.3 to -1.5%)	0.0% (0.3 to -0.7%)	-14.3% (-9.4 to -20.2%)	-6.6% (-2.4 to -13.8%)	0.4% (0 to 1.1%)	-0.6% (-0.2 to -1.4%)	-0.2% (-0.1 to -0.3%)	-11.8% (-8.4 to -12.9%)	-3.6% (-2.5 to -4.2%)	1.5% (1.6 to 1.5%)	-1.9% (-1.5 to -2.4%)	-4.4% (-3.2 to -5.8%)
Virgin Islands, U.S.	-1.5% (-0.8 to -2.7%)	-5.7% (-4.1 to -7.3%)	-11.5% (-7.9 to -15.6%)	-4.0% (-3.3 to -3.3%)	0.0% (0 to 0.2%)	-0.5% (-0.1 to -1.2%)	-0.2% (-0.1 to -0.3%)	-3.2% (-1.7 to -5.1%)	-7.5% (-5.9 to -8.8%)	0.9% (1.2 to 0.7%)	-0.2% (-0.2 to -0.5%)	-2.1% (-1.5 to -2.7%)
Bosnia and Herzegovina	-1.8% (-0.6 to -3.0%)	-6.3% (-5.9 to -6.0%)	-19.0% (-14.9 to -22.9%)	-21.7% (-10.6 to -34.0%)	0.0% (0 to 0.1%)	-1.6% (-0.5 to -3.3%)	-0.5% (-0.3 to -0.8%)	-10.7% (-6.9 to -14.1%)	-5.2% (-4.3 to -5.3%)	1.1% (1.2 to 0.8%)	-0.1% (-0.2 to -0.1%)	-6.3% (-5.8 to -6.0%)
Samoa	-2.9% (-1.1 to -5.5%)	0.1% (-0.1 to 0.7%)	-13.3% (-8.8 to -18.5%)	-25.7% (-12.8 to -39.3%)	-0.4% (0 to -1.5%)	0.0% (0.0 to 0.0%)	-0.1% (0.0 to -0.3%)	-3.3% (-3.4 to -1.9%)	-3.4% (-3.1 to -4.1%)	0.5% (0.5 to 0.4%)	-0.1% (-0.1 to -0.4%)	-3.8% (-2.6 to -4.8%)
Antigua and Barbuda	-3.4% (-1.6 to -5.3%)	0.1% (0.0 to 0.6%)	-27.3% (-24.9 to -28.1%)	-10.0% (-8.2 to -13.9%)	-0.4% (0 to -1.2%)	-1.0% (-0.2 to -1.9%)	-0.7% (-0.4 to -1.0%)	-7.1% (-3.7 to -10.6%)	-6.8% (-3.3 to -7.5%)	0.6% (0.7 to 0.4%)	-0.5% (-0.3 to -0.7%)	-3.1% (-2.5 to -3.9%)
Fiji	-10.0% (-3.7 to -16.6%)	-24.0% (-14.4 to -39.3%)	-28.6% (-25.0 to -31.2%)	-45.7% (-26.9 to -65.9%)	-0.4% (0 to -1.7%)	-0.5% (-0.1 to -1.3%)	-1.4% (-1.1 to -1.8%)	-30.5% (-28.5 to -17.1%)	-1.3% (-1.5 to -2.8%)	0.3% (0.3 to 0.5%)	-0.7% (-0.5 to -0.6%)	-5.3% (-4.1 to -6.8%)
Ukraine	-0.8% (-0.3 to -1.7%)	-1.6% (-0.8 to -2.4%)	-2.2% (-0.8 to -3.7%)	-4.0% (-2.2 to -5.9%)	0.0% (0 to 0.0%)	-1.0% (-0.3 to -1.8%)	-0.2% (-0.1 to -0.4%)	-8.2% (-5.7 to -8.9%)	-3.4% (-3.5 to -3.3%)	1.3% (1.5 to 1.2%)	-0.6% (-0.4 to -0.7%)	-0.3% (-0.2 to -0.3%)
Barbados	-1.1% (-0.5 to -1.7%)	0.1% (0.0 to 0.6%)	-15.4% (-14.4 to -16.3%)	-3.0% (-0.8 to -6.0%)	0.0% (0 to 0.0%)	-0.6% (-0.2 to -1.2%)	-0.3% (-0.2 to -0.5%)	-4.3% (-3.1 to -6.2%)	-2.9% (-1.3 to -3.7%)	0.6% (0.6 to 0.5%)	-0.3% (-0.1 to -0.4%)	-1.5% (-1.0 to -2.3%)
Belarus	-3.0% (-1.2 to -5.3%)	0.0% (0.0 to -0.5%)	-13.2% (-11.1 to -15.4%)	-20.5% (-9.3 to -30.1%)	0.1% (0 to 0.3%)	-0.9% (-0.2 to -1.9%)	-0.3% (-0.2 to -0.4%)	-8.1% (-4.1 to -11.9%)	-4.1% (-2.7 to -5.7%)	1.0% (1.2 to 0.9%)	-0.4% (-0.3 to -0.5%)	-0.4% (-0.2 to -0.6%)
Chile	-2.0% (-0.8 to -3.6%)	0.1% (0.1 to 0.6%)	-18.2% (-15.8 to -20.0%)	-20.3% (-8.0 to -33.7%)	-0.1% (0 to -0.4%)	-1.0% (-0.4 to -2.0%)	-0.3% (-0.2 to -0.3%)	-8.1% (-5.8 to -8.1%)	-5.8% (-4.4 to -6.7%)	0.9% (0.9 to 0.9%)	-0.9% (-0.8 to -1.2%)	-2.9% (-2.4 to -3.6%)
Tonga	-5.7% (-2.4 to -9.5%)	0.0% (-0.1 to 0.2%)	-19.7% (-16.6 to -22.2%)	-21.9% (-14.9 to -30.2%)	-0.3% (0 to -1.0%)	-1.5% (-0.3 to -3.6%)	-0.8% (-0.4 to -1.2%)	-15.8% (-9.6 to -23.9%)	-1.1% (-0.5 to -1.8%)	0.4% (0.3 to 0.4%)	-0.6% (-0.4 to -0.8%)	-3.4% (-2.3 to -4.7%)
The Bahamas	-0.9% (-0.3 to -1.8%)	-5.6% (-3.8 to -7.6%)	-13.6% (-11.7 to -14.2%)	-2.1% (-0.4 to -0.6%)	-0.1% (0 to 0.1%)	-0.5% (-0.1 to -1.2%)	-0.2% (-0.1 to -0.3%)	-3.4% (-2.3 to -6.2%)	-6.1% (-5.1 to -7.2%)	0.6% (0.8 to 0.6%)	-0.7% (-0.5 to -0.8%)	-2.1% (-1.6 to -2.9%)
Trinidad and Tobago	-2.2% (-1.0 to -4.2%)	0.1% (0.0 to 0.6%)	-20.4% (-19.3 to -20.8%)	-8.4% (-5.6 to -10.5%)	-0.3% (0 to -0.9%)	-0.3% (-0.1 to -0.6%)	-0.5% (-0.3 to -0.8%)	-7.4% (-5.1 to -10.7%)	1.0% (0.2 to 3.1%)	0.5% (0.8 to 0.5%)	-1.1% (-0.9 to -1.1%)	-3.0% (-2.5 to -3.5%)

Location	Hand-washing	Low rotavirus vaccine coverage	Unsafe sanitation	Unsafe water	Zinc deficiency	Stunting	Under-weight	Wasting	Low oral rehydration solution coverage	Low weight and short gestation	Sub-optimal Breast feeding	Vitamin A deficiency
Palestine	-4.7% (-1.9 to -7.6%)	-17.2% (-10.6 to -25.7%)	-40.5% (-31.7 to -48.6%)	-12.5% (-7.9 to -17.9%)	-0.2% (0 to -0.3%)	-2.4% (-0.9 to -4.6%)	-3.0% (-2.4 to -3.7%)	-33.6% (-25.9 to -34.1%)	-1.8% (-1.3 to -2.2%)	0.9% (1.0 to 0.8%)	-0.6% (-0.3 to -1.4%)	-6.7% (-5.2 to -7.7%)
Russian Federation	-1.8% (-0.6 to -3.2%)	0.3% (0.0 to 1.4%)	-15.6% (-13.6 to -17.0%)	-15.0% (-6.5 to -25.5%)	-0.1% (0 to -0.4%)	-2.0% (-0.8 to -3.4%)	-0.5% (-0.4 to -0.7%)	-9.0% (-7.7 to -6.5%)	-7.0% (-4.9 to -8.6%)	1.4% (1.4 to 1.3%)	0.1% (-0.1 to 0.1%)	-0.4% (-0.3 to -0.5%)
Malaysia	-0.9% (-0.3 to -1.8%)	0.7% (0.5 to 1.5%)	-17.1% (-11.4 to -23.1%)	-15.1% (-6.6 to -24.2%)	-0.4% (0 to -1.4%)	-3.4% (-1.3 to -6.4%)	-2.6% (-2.2 to -3.3%)	-4.6% (-4.0 to -5.2%)	-2.9% (-3.1 to -3.2%)	0.5% (0.5 to 0.5%)	-0.9% (-0.6 to -1.2%)	-2.9% (-2.3 to -3.8%)
Cuba	-2.8% (-1.0 to -5.3%)	0.0% (0.1 to -0.2%)	-10.6% (-7.9 to -12.7%)	-7.8% (-5.0 to -11.2%)	0.0% (0 to -0.2%)	0.0% (0.0 to 0.1%)	-0.1% (-0.1 to -0.1%)	1.9% (1.2 to 5.4%)	-3.2% (-2.5 to -4.8%)	0.8% (0.7 to 0.8%)	-0.4% (-0.4 to -0.3%)	-1.1% (-0.8 to -1.5%)
North Korea	-0.9% (-0.3 to -1.8%)	0.4% (0.3 to 0.2%)	-7.7% (-7.8 to -7.9%)	-14.0% (-5.1 to -25.9%)	0.3% (0 to 1.2%)	-4.5% (-1.8 to -8.8%)	-3.9% (-2.7 to -7.0%)	-51.3% (-45.1 to -51.9%)	-5.0% (-3.5 to -4.1%)	0.0% (0.1 to 0.1%)	-0.5% (-0.5 to -0.8%)	5.3% (4.4 to 6.9%)
Grenada	-4.5% (-2.1 to -7.6%)	0.1% (0.0 to 0.6%)	-19.0% (-18.1 to -19.6%)	-11.3% (-7.9 to -15.8%)	-0.3% (0 to -1.1%)	-1.5% (-0.4 to -3.1%)	-1.4% (-1.0 to -2.1%)	-10.4% (-6.2 to -15.5%)	-4.2% (-3.9 to -6.2%)	0.3% (0.3 to 0.3%)	-0.3% (0.0 to -0.8%)	-2.8% (-2.2 to -3.6%)
Romania	-2.2% (-0.9 to -3.8%)	-5.7% (-2.1 to -12.8%)	-16.1% (-14.7 to -17.2%)	-27.1% (-14.8 to -38.4%)	0.0% (0 to 0.0%)	-0.7% (-0.4 to -1.4%)	-0.8% (-0.6 to -1.0%)	-14.1% (-12.0 to -10.2%)	-5.2% (-4.7 to -4.6%)	0.4% (0.5 to 0.5%)	-0.1% (-0.2 to -0.2%)	-6.7% (-5.4 to -7.8%)
Uruguay	-1.8% (-0.7 to -3.3%)	-0.1% (0.1 to 0.3%)	-18.9% (-14.6 to -23.4%)	-18.7% (-6.8 to -31.8%)	0.1% (0 to 0.3%)	-1.1% (-0.5 to -1.7%)	-0.6% (-0.5 to -0.8%)	-13.1% (-10.1 to -11.6%)	-3.1% (-2.7 to -3.6%)	1.6% (1.6 to 1.7%)	-1.5% (-1.4 to -2.2%)	-3.1% (-2.4 to -4.0%)
Costa Rica	-3.7% (-1.6 to -6.2%)	0.1% (0.0 to 0.3%)	-15.6% (-12.2 to -19.1%)	-18.5% (-8.1 to -29.3%)	-0.1% (0 to -0.5%)	-1.3% (-0.4 to -3.0%)	-0.3% (-0.2 to -0.4%)	-9.1% (-5.9 to -14.2%)	-1.5% (-1.3 to -1.7%)	0.2% (0.2 to 0.2%)	-0.6% (-0.4 to -1.0%)	-2.7% (-2.1 to -3.1%)
Central African Republic	-0.6% (0.2 to -1.9%)	-26.8% (-3.8 to -92.4%)	-0.5% (0.2 to -1.7%)	-3.7% (-1.0 to -8.2%)	-6.3% (0 to -19.4%)	-3.2% (-1.8 to -4.0%)	-2.0% (-2.2 to -2.7%)	-20.9% (-18.4 to -5.9%)	6.3% (4.1 to 8.0%)	0.6% (0.4 to 0.7%)	-7.4% (-6.1 to -7.9%)	-15.1% (-13.4 to -14.6%)
Zimbabwe	0.5% (0.0 to 0.5%)	-13.4% (-11.1 to -14.5%)	-0.2% (0.8 to -0.9%)	1.1% (-0.2 to 0.0%)	0.2% (0 to 0.6%)	-3.1% (-0.9 to -7.8%)	-0.5% (-0.8 to 0.0%)	-4.3% (-3.7 to -5.4%)	-13.2% (-8.9 to -15.8%)	0.9% (0.7 to 0.8%)	-7.1% (-5.4 to -8.7%)	-8.7% (-6.4 to -10.9%)
Greenland	-0.5% (-0.2 to -0.7%)	1.1% (0.1 to 0.4%)	-20.6% (-14.0 to -28.8%)	-20.0% (-4.8 to -48.9%)	-0.2% (0 to -0.1%)	-0.7% (-0.1 to -1.4%)	-0.3% (-0.2 to -0.5%)	-6.8% (-4.3 to -8.6%)	-8.0% (-7.0 to -9.5%)	1.8% (1.8 to 1.7%)	-0.7% (0.0 to -0.9%)	-1.8% (-1.8 to -2.7%)
Puerto Rico	-2.6% (-1.1 to -4.6%)	-18.9% (-12.0 to -25.2%)	-0.2% (0.0 to -1.2%)	-8.9% (-6.4 to -14.3%)	-0.1% (0 to -0.2%)	-1.0% (-0.2 to -2.3%)	-0.3% (-0.2 to -0.5%)	-6.5% (-3.2 to -11.7%)	-8.5% (-5.2 to -6.8%)	2.0% (2.1 to 1.4%)	-0.6% (0.6 to -0.6%)	-4.0% (-2.8 to -5.7%)
United States	-0.6% (-0.2 to -1.1%)	-13.2% (-8.5 to -18.4%)	-12.6% (-9.1 to -16.1%)	-16.6% (-3.3 to -44.0%)	-0.3% (0 to -1.0%)	-0.5% (-0.1 to -1.1%)	-0.2% (-0.2 to -0.4%)	-3.5% (-1.8 to -4.9%)	-8.4% (-6.4 to -10.1%)	1.5% (1.4 to 1.5%)	-5.0% (-5.2 to -5.2%)	0.9% (0.5 to 1.2%)

Location	Hand-washing	Low rotavirus vaccine coverage	Unsafe sanitation	Unsafe water	Zinc deficiency	Stunting	Under-weight	Wasting	Low oral rehydration solution coverage	Low weight and short gestation	Sub-optimal Breast feeding	Vitamin A deficiency
Canada	-1.0% (-0.5 to -2.0%)	-46.5% (-24.4 to -77.3%)	-31.4% (-18.1 to -48.0%)	-38.4% (-7.1 to -105.7%)	2.0% (0 to 5.5%)	-1.6% (-0.3 to -4.4%)	-0.7% (-0.4 to -1.1%)	-18.4% (-7.9 to -30.3%)	-22.2% (-22.6 to -32.4%)	5.6% (5.1 to 6.3%)	-6.5% (-7.1 to -7.6%)	-11.5% (-11.9 to -11.1%)
Hungary	-1.5% (-0.6 to -2.8%)	-1.1% (-7.1 to 9.0%)	-46.3% (-33.9 to -59.8%)	-54.7% (-17.1 to -122.5%)	0.8% (0 to 3.1%)	-2.7% (-0.7 to -6.1%)	-0.9% (-0.6 to -1.3%)	-26.6% (-16.2 to -43.2%)	-15.1% (-7.7 to -14.9%)	4.8% (5.1 to 4.4%)	0.7% (0.7 to 0.5%)	-13.9% (-10.6 to -17.2%)
Austria	-1.0% (-0.3 to -2.3%)	-78.6% (-46.2 to -127.6%)	-15.4% (-10.0 to -22.0%)	-35.9% (-6.6 to -100.4%)	0.3% (0 to 0.3%)	-0.9% (-0.2 to -1.9%)	-0.4% (-0.3 to -0.6%)	-11.7% (-6.0 to -19.9%)	-13.7% (-12.7 to -13.4%)	4.8% (4.9 to 5.0%)	-1.9% (-2.2 to -1.4%)	-4.1% (-3.6 to -8.0%)
Sweden	-0.4% (-0.1 to -0.9%)	13.0% (5.9 to 30.2%)	-11.1% (-6.3 to -19.0%)	-10.4% (-1.9 to -27.0%)	1.7% (0 to 4.2%)	-1.1% (-0.2 to -2.9%)	-0.5% (-0.3 to -0.7%)	-12.1% (-5.9 to -20.3%)	-13.1% (-9.5 to -12.2%)	2.2% (2.4 to 1.8%)	-4.8% (-4.2 to -4.9%)	-3.8% (-4.0 to -4.6%)
Australia	-1.4% (-0.4 to -2.4%)	-57.9% (-48.7 to -63.1%)	-9.1% (-5.2 to -14.6%)	-7.4% (-1.4 to -15.8%)	-0.4% (0 to -1.2%)	-0.8% (-0.3 to -1.1%)	-0.3% (-0.2 to -0.5%)	-8.1% (-3.5 to -10.4%)	-8.4% (-5.1 to -11.5%)	1.0% (1.0 to 0.9%)	1.9% (1.4 to 3.4%)	-0.8% (-0.6 to -0.9%)
Ireland	-0.4% (-0.1 to -0.7%)	6.4% (4.2 to 9.5%)	-23.2% (-13.3 to -36.9%)	-9.1% (-1.6 to -24.4%)	0.0% (0 to -0.2%)	-0.8% (-0.2 to -1.9%)	-0.4% (-0.2 to -0.6%)	-10.3% (-5.0 to -16.1%)	-9.2% (-6.9 to -8.4%)	3.8% (4.1 to 3.7%)	-1.5% (-1.0 to -2.2%)	-4.6% (-3.0 to -8.9%)
United Kingdom	-0.4% (-0.1 to -0.6%)	-17.8% (-9.9 to -27.5%)	-1.2% (-0.7 to -2.2%)	-10.5% (-1.9 to -30.1%)	-0.3% (0 to -1.1%)	-0.8% (-0.3 to -1.8%)	-0.4% (-0.3 to -0.5%)	-9.6% (-5.2 to -12.8%)	-8.6% (-6.6 to -10.6%)	1.7% (1.7 to 1.7%)	-4.1% (-3.5 to -5.5%)	0.9% (0.3 to 1.5%)
Netherlands	-0.2% (0.0 to -0.5%)	-0.8% (-0.4 to 3.4%)	-4.2% (-2.2 to -6.8%)	-4.6% (-0.8 to -13.4%)	1.6% (0 to 4.7%)	-0.5% (-0.1 to -1.5%)	-0.2% (-0.1 to -0.4%)	-6.3% (-3.5 to -9.9%)	-7.8% (-8.1 to -9.9%)	1.3% (1.3 to 1.2%)	1.1% (0.8 to 1.7%)	-4.5% (-3.9 to -6.3%)
Czech Republic	-0.9% (-0.3 to -1.8%)	0.2% (0.5 to 0.2%)	-4.9% (-2.9 to -7.7%)	-28.9% (-8.7 to -67.9%)	0.1% (0 to 0.4%)	-0.6% (-0.2 to -1.1%)	-0.4% (-0.3 to -0.5%)	-27.5% (-21.1 to -21.3%)	-11.5% (-10.7 to -14.1%)	3.5% (3.5 to 3.4%)	-0.2% (-0.5 to -0.3%)	-11.9% (-8.9 to -15.6%)
Iceland	-0.2% (-0.1 to -0.3%)	1.5% (1.0 to 2.4%)	-4.1% (-2.2 to -6.7%)	-4.2% (-0.7 to -12.0%)	1.6% (0 to 4.5%)	-0.4% (-0.1 to -1.0%)	-0.2% (-0.1 to -0.3%)	-5.4% (-2.9 to -8.8%)	-6.4% (-3.5 to -7.5%)	2.3% (2.3 to 2.5%)	0.9% (-0.2 to 1.5%)	-1.5% (-0.7 to -2.8%)
Belgium	-0.2% (0.0 to -0.3%)	-39.9% (-22.6 to -68.1%)	-4.1% (-2.2 to -6.8%)	-4.1% (-0.7 to -11.2%)	-0.4% (0 to -0.5%)	-0.4% (-0.1 to -1.1%)	-0.2% (-0.1 to -0.3%)	-5.5% (-3.4 to -7.7%)	-6.1% (-4.4 to -9.0%)	0.8% (0.9 to 0.9%)	-0.9% (-1.2 to -1.0%)	-2.7% (-2.7 to -5.3%)
Italy	-0.1% (-0.1 to -0.3%)	-13.7% (-9.0 to -22.0%)	-4.7% (-2.5 to -7.3%)	-4.3% (-0.8 to -11.8%)	0.0% (0 to 0.1%)	-0.4% (-0.1 to -1.1%)	-0.2% (-0.1 to -0.3%)	-5.7% (-3.0 to -8.7%)	-6.2% (-5.3 to -8.8%)	1.8% (1.8 to 1.8%)	-0.2% (-0.4 to -0.1%)	-5.0% (-4.1 to -4.2%)
New Zealand	-0.9% (-0.4 to -1.4%)	-10.7% (-7.3 to -13.7%)	-10.0% (-6.0 to -15.3%)	-5.5% (-1.9 to -9.1%)	-0.3% (0 to -0.9%)	-0.6% (-0.2 to -1.5%)	-0.2% (-0.2 to -0.4%)	-5.4% (-3.1 to -7.3%)	-6.2% (-4.3 to -6.3%)	0.7% (0.7 to 0.7%)	-0.5% (-0.5 to -0.1%)	-0.5% (-0.4 to -0.6%)
Denmark	-0.1% (0.0 to -0.3%)	-1.4% (-0.3 to -5.4%)	-2.3% (-1.2 to -3.5%)	-2.7% (-0.4 to -7.0%)	0.9% (0 to 2.5%)	-0.3% (-0.1 to -0.7%)	-0.1% (-0.1 to -0.2%)	-3.6% (-2.2 to -4.5%)	-5.7% (-2.6 to -4.0%)	3.1% (3.3 to 2.8%)	-3.5% (-3.2 to -4.3%)	-1.5% (-0.6 to -4.0%)

Location	Hand-washing	Low rotavirus vaccine coverage	Unsafe sanitation	Unsafe water	Zinc deficiency	Stunting	Under-weight	Wasting	Low oral rehydration solution coverage	Low weight and short gestation	Sub-optimal Breast feeding	Vitamin A deficiency
Malta	-0.2% (-0.1 to -0.5%)	1.7% (0.5 to 1.0%)	-8.0% (-4.6 to -12.5%)	-6.8% (-1.2 to -18.3%)	0.0% (0 to 0.3%)	-0.5% (-0.1 to -1.3%)	-0.3% (-0.1 to -0.4%)	-6.2% (-2.6 to -9.9%)	-5.3% (-5.0 to -6.3%)	2.0% (2.1 to 1.9%)	-0.9% (-0.7 to -1.4%)	-2.7% (-1.1 to -6.9%)
Norway	-0.2% (-0.1 to -0.6%)	-4.0% (-1.7 to -8.2%)	-4.8% (-2.6 to -8.0%)	-4.8% (-0.8 to -13.4%)	0.4% (0 to 1.8%)	-0.4% (-0.1 to -0.9%)	-0.2% (-0.1 to -0.3%)	-5.8% (-3.5 to -7.9%)	-6.8% (-4.2 to -8.8%)	2.5% (2.5 to 2.5%)	0.3% (0.2 to 0.7%)	-3.3% (-2.5 to -4.9%)
Japan	-0.6% (-0.2 to -1.6%)	-1.4% (-1.0 to -2.1%)	-3.9% (-2.2 to -6.2%)	-1.8% (-0.7 to -5.3%)	-0.3% (0 to -1.4%)	-0.6% (-0.2 to -1.3%)	-0.5% (-0.4 to -0.7%)	-4.7% (-2.7 to -5.8%)	-4.8% (-4.0 to -5.6%)	1.8% (1.8 to 1.8%)	-0.8% (-0.6 to -0.8%)	-1.7% (-1.2 to -2.2%)
Greece	-0.1% (0.0 to -0.3%)	-4.9% (-3.1 to -7.2%)	-11.9% (-6.7 to -17.4%)	-0.4% (-0.1 to -0.9%)	0.2% (0 to 0.5%)	-0.5% (-0.1 to -1.4%)	-0.3% (-0.2 to -0.3%)	-6.5% (-3.3 to -9.4%)	-5.2% (-5.3 to -4.5%)	4.7% (5.1 to 4.9%)	1.0% (0.8 to 1.2%)	-2.6% (-1.2 to -6.1%)
Singapore	-1.9% (-0.8 to -3.4%)	0.4% (0.1 to 0.4%)	-15.8% (-9.2 to -24.9%)	-16.5% (-3.4 to -43.4%)	0.1% (0 to 0.4%)	-0.6% (-0.2 to -1.7%)	-0.9% (-0.5 to -1.3%)	-19.1% (-11.3 to -26.0%)	-8.6% (-4.3 to -10.7%)	8.7% (8.3 to 10.3%)	-9.2% (-7.6 to -11.4%)	-4.1% (-3.0 to -5.5%)
Slovakia	-0.9% (-0.3 to -1.6%)	0.1% (0.3 to -0.1%)	-10.7% (-6.7 to -16.6%)	-24.6% (-7.8 to -52.6%)	0.2% (0 to 0.7%)	-1.4% (-0.5 to -2.8%)	-0.4% (-0.3 to -0.6%)	-12.4% (-7.2 to -17.9%)	-8.1% (-5.2 to -9.6%)	1.4% (1.4 to 1.6%)	-0.2% (-0.1 to -0.3%)	-9.8% (-7.4 to -12.1%)
Germany	-0.1% (0.0 to 0.0%)	-10.8% (-7.1 to -17.2%)	-3.5% (-2.0 to -6.0%)	-3.5% (-0.7 to -9.8%)	0.2% (0 to 0.8%)	-0.2% (0.0 to -0.4%)	-0.1% (-0.1 to -0.2%)	-2.8% (-1.6 to -3.8%)	-4.6% (-1.5 to -4.8%)	1.3% (1.3 to 1.5%)	-0.2% (0.2 to -0.3%)	-1.1% (-0.6 to -1.4%)
Andorra	-0.1% (0.0 to 0.0%)	1.4% (0.4 to 0.8%)	-1.6% (-0.9 to -2.7%)	-2.0% (-0.3 to -5.5%)	0.0% (0 to 0.2%)	-0.2% (-0.1 to -0.6%)	-0.1% (-0.1 to -0.1%)	-3.2% (-1.5 to -4.7%)	-4.6% (-4.0 to -5.7%)	1.0% (1.0 to 1.0%)	-1.1% (-1.1 to -1.2%)	-1.7% (-0.7 to -3.4%)
Brunei	-1.0% (-0.4 to -1.7%)	0.1% (-0.3 to 0.4%)	-10.3% (-6.4 to -15.4%)	-9.8% (-2.6 to -21.1%)	0.0% (0 to 0.1%)	-0.1% (0.0 to -0.3%)	-0.4% (-0.3 to -0.6%)	-3.5% (-2.2 to -4.7%)	-4.9% (-3.7 to -4.8%)	1.3% (1.6 to 1.3%)	-0.9% (-0.7 to -1.7%)	-2.7% (-1.7 to -3.8%)
Israel	-0.2% (0.0 to -0.3%)	-48.9% (-27.7 to -87.8%)	-6.7% (-3.8 to -9.7%)	-6.8% (-1.3 to -18.5%)	0.7% (0 to 1.7%)	-0.6% (-0.1 to -1.4%)	-0.3% (-0.2 to -0.4%)	-7.0% (-3.7 to -11.7%)	-6.7% (-4.9 to -9.4%)	2.5% (2.5 to 2.5%)	-1.2% (-1.0 to -1.0%)	-2.6% (-3.5 to -1.3%)
Spain	-0.1% (-0.1 to -0.4%)	1.5% (2.1 to 0.9%)	-3.7% (-2.1 to -6.2%)	-1.6% (-0.2 to -4.1%)	-0.1% (0 to -0.3%)	-0.2% (0.0 to -0.5%)	-0.1% (-0.1 to -0.2%)	-2.8% (-1.7 to -4.9%)	-4.3% (-3.5 to -6.0%)	3.0% (3.0 to 2.9%)	0.6% (0.2 to 1.1%)	-2.5% (-2.0 to -2.9%)
Northern Mariana Islands	-0.9% (-0.2 to -1.7%)	0.0% (-0.1 to 0.1%)	-4.3% (-2.5 to -6.4%)	-11.3% (-5.0 to -19.1%)	-1.2% (0 to -3.8%)	0.0% (0.0 to 0.1%)	-0.1% (0.0 to -0.1%)	-2.7% (-2.3 to -5.5%)	-2.9% (-2.4 to -2.8%)	0.2% (0.2 to 0.1%)	-0.8% (-0.5 to -1.1%)	-1.4% (-1.1 to -1.8%)
Finland	-0.1% (0.0 to -0.1%)	-15.1% (-11.5 to -18.0%)	-1.9% (-1.0 to -3.1%)	-2.0% (-0.4 to -5.5%)	0.0% (0 to 0.0%)	-0.2% (0.0 to -0.4%)	-0.1% (0.0 to -0.1%)	-2.0% (-0.6 to -3.0%)	-3.3% (-2.7 to -2.7%)	2.3% (2.4 to 2.1%)	-0.2% (-0.4 to -0.1%)	-1.1% (-1.0 to -2.0%)
Guam	-2.8% (-1.0 to -5.1%)	0.1% (-0.1 to 1.1%)	-13.2% (-8.3 to -19.8%)	-27.2% (-13.8 to -43.0%)	0.2% (0 to -0.1%)	-0.6% (-0.2 to -1.6%)	-0.3% (-0.2 to -0.4%)	-9.8% (-7.0 to -12.1%)	-1.8% (-0.6 to -1.8%)	0.1% (-0.1 to 0.4%)	-0.9% (-0.7 to -0.8%)	-4.5% (-3.4 to -6.2%)

Location	Hand-washing	Low rotavirus vaccine coverage	Unsafe sanitation	Unsafe water	Zinc deficiency	Stunting	Under-weight	Wasting	Low oral rehydration solution coverage	Low weight and short gestation	Sub-optimal Breast feeding	Vitamin A deficiency
Slovenia	-0.6% (-0.2 to -1.0%)	0.0% (0.2 to 0.2%)	-6.8% (-4.2 to -10.1%)	-10.5% (-3.5 to -21.3%)	0.5% (0 to 1.9%)	-0.8% (-0.2 to -1.5%)	-0.2% (-0.2 to -0.3%)	-7.3% (-4.2 to -11.6%)	-6.0% (-5.5 to -5.0%)	0.8% (0.9 to 0.9%)	0.6% (0.5 to 0.8%)	-5.4% (-4.1 to -7.1%)
Cyprus	-0.3% (-0.1 to -0.6%)	1.2% (0.4 to 0.7%)	-5.9% (-3.4 to -9.3%)	-4.1% (-0.8 to -10.8%)	0.0% (0 to 0.0%)	-0.4% (-0.1 to -1.1%)	-0.2% (-0.1 to -0.3%)	-5.0% (-2.7 to -6.4%)	-3.6% (-2.5 to -4.4%)	2.3% (2.7 to 1.7%)	-0.4% (-0.2 to -0.6%)	-2.6% (-1.3 to -4.3%)
Luxembourg	-0.1% (0.0 to -0.1%)	-24.8% (-14.5 to -41.5%)	-1.9% (-1.0 to -3.2%)	-2.3% (-0.4 to -5.1%)	0.0% (0 to -0.7%)	-0.3% (-0.1 to -0.6%)	-0.1% (-0.1 to -0.2%)	-3.7% (-2.3 to -5.7%)	-5.4% (-2.7 to -5.8%)	2.0% (2.1 to 2.0%)	-0.8% (-0.7 to -0.9%)	-1.9% (-1.2 to -3.8%)
Croatia	-0.5% (-0.2 to -0.7%)	0.1% (0.3 to 0.0%)	-5.6% (-3.4 to -8.1%)	-14.2% (-5.3 to -26.5%)	-0.7% (0 to -2.3%)	-0.7% (-0.2 to -2.0%)	-0.2% (-0.1 to -0.4%)	-7.4% (-3.5 to -12.5%)	-6.5% (-5.5 to -5.8%)	0.7% (0.8 to 0.6%)	0.9% (0.5 to 0.6%)	-6.7% (-5.1 to -8.4%)
Switzerland	-0.1% (-0.1 to -0.1%)	1.8% (0.3 to 5.4%)	-1.4% (-0.8 to -2.5%)	-1.7% (-0.3 to -4.8%)	0.0% (0 to -0.1%)	-0.1% (0.0 to -0.2%)	0.0% (0.0 to -0.1%)	-1.9% (-0.8 to -2.2%)	-4.1% (-3.4 to -3.5%)	2.8% (2.6 to 2.8%)	0.3% (0.4 to 0.2%)	-0.5% (-0.4 to -1.1%)
Seychelles	-1.3% (-0.5 to -1.9%)	-23.2% (-14.2 to -36.4%)	-29.2% (-23.6 to -35.3%)	-3.4% (-2.6 to -9.5%)	-0.6% (0 to -2.1%)	-0.7% (-0.2 to -1.7%)	-1.2% (-0.9 to -1.6%)	0.4% (-0.5 to 0.3%)	-5.3% (-5.4 to -5.2%)	1.4% (1.4 to 0.9%)	-1.4% (-1.0 to -1.6%)	-4.2% (-3.3 to -4.4%)

Supplementary Figure. Maps of the Socio-demographic Index by country in 1990, 2017, and the difference between 1990-2017. The difference shown is in absolute terms. The SDI is scaled between 0 and 1 so the difference is simply the difference in the value in 2017 and 1990. A) The SDI value in 1990; B) The SDI value in 2017; and C) The difference in SDI between 1990 and 2017.

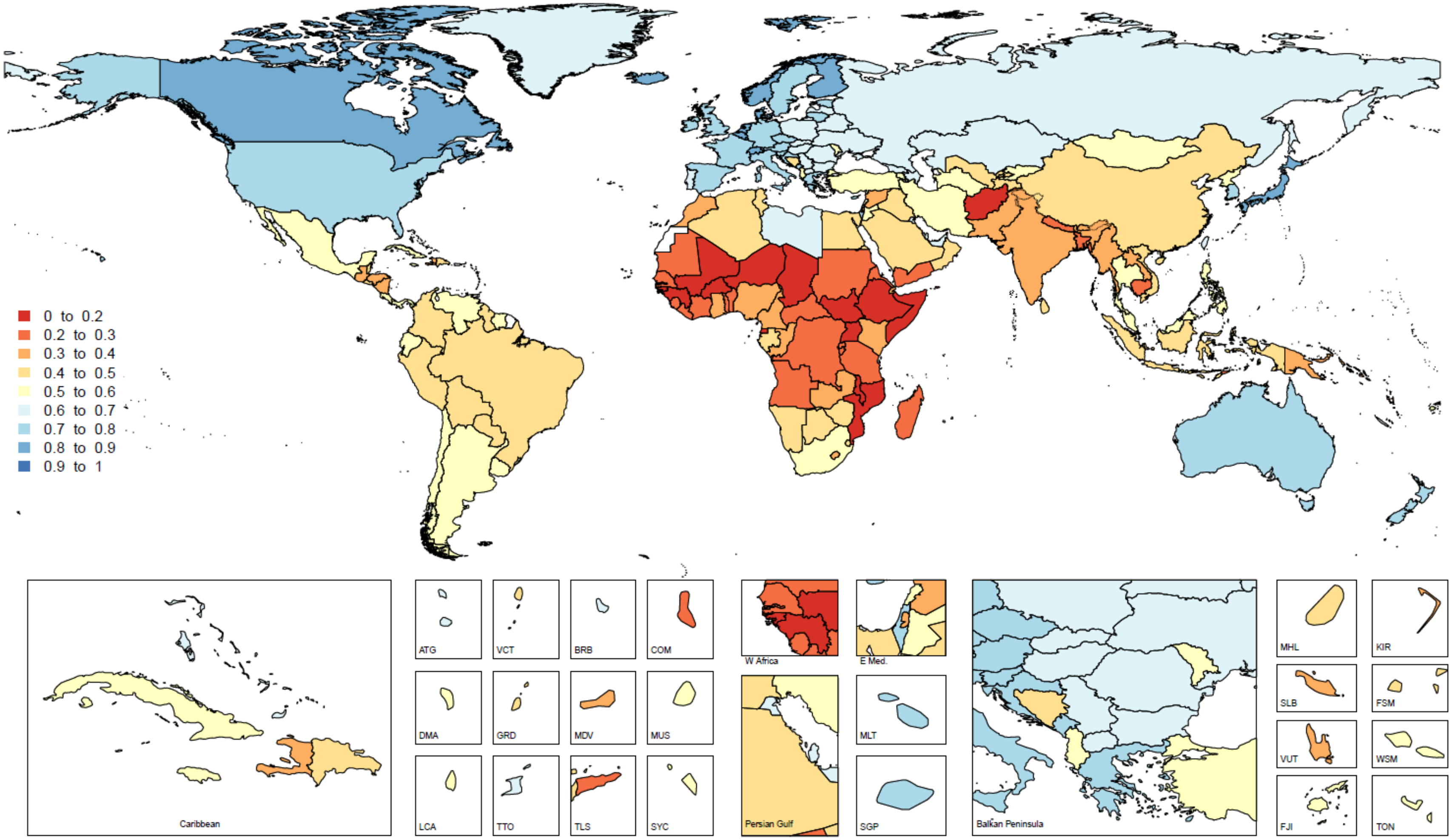
0 to 0.2
0.2 to 0.3
0.3 to 0.4
0.4 to 0.5
0.5 to 0.6
0.6 to 0.7
0.7 to 0.8
0.8 to 0.9
0.9 to 1
Caribbean
ATG
VCT
BRB
COM
DMA
GRD
MDV
MUS
LCA
TTO
TLS
SYC
W Africa
E Med.
Persian Gulf
MLT
SGP
Balkan Peninsula
MHL
KIR
SLB
FSM
VUT
WSM
FJI
TON

A)

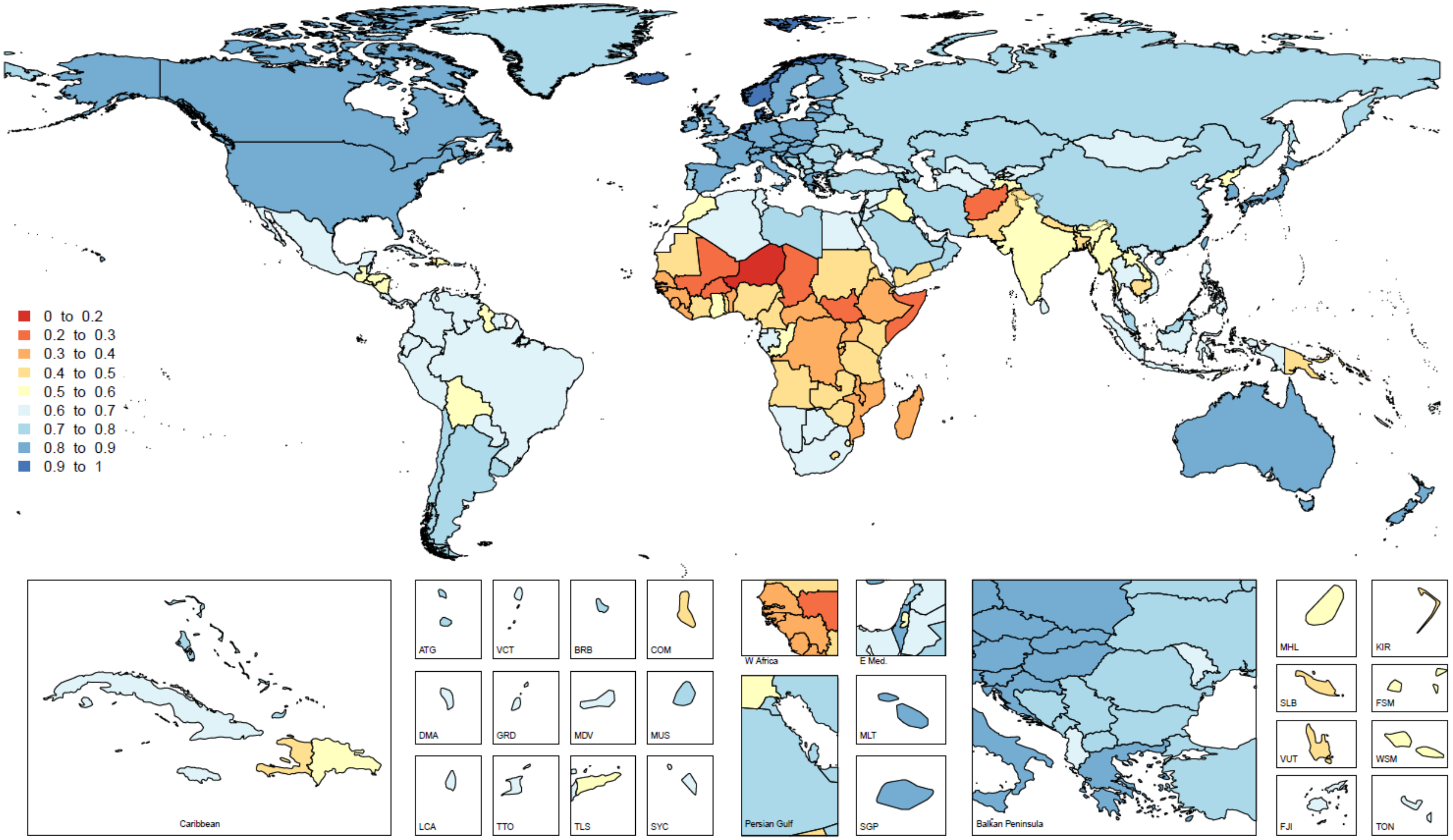
0 to 0.2
0.2 to 0.3
0.3 to 0.4
0.4 to 0.5
0.5 to 0.6
0.6 to 0.7
0.7 to 0.8
0.8 to 0.9
0.9 to 1
Caribbean
ATG
VCT
BRB
COM
DMA
GRD
MDV
MUS
LCA
TTO
TLS
SYC
W Africa
E Med.
Persian Gulf
MLT
SGP
Balkan Peninsula
MHL
KIR
SLB
FSM
VUT
WSM
FJI
TON

B)

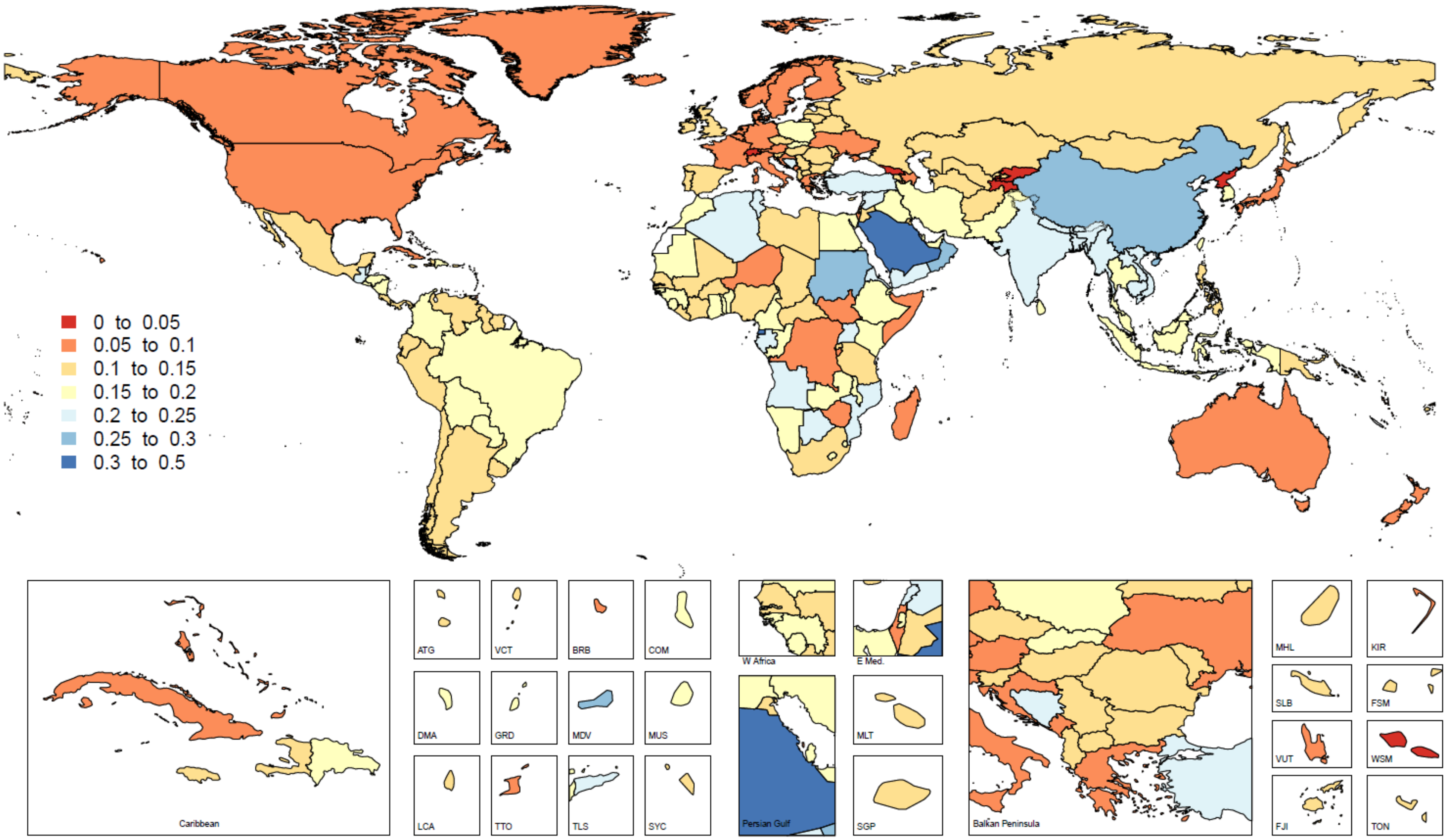
0 to 0.05
0.05 to 0.1
0.1 to 0.15
0.15 to 0.2
0.2 to 0.25
0.25 to 0.3
0.3 to 0.5
Caribbean
ATG
VCT
BRB
COM
DMA
GRD
MDV
MUS
LCA
TTO
TLS
SYC
W Africa
E Med.
Persian Gulf
MLT
SGP
Balkan Peninsula
MHL
KIR
SLB
FSM
VUT
WSM
FJI
TON

C)

Supplementary Figure. Bar charts for the aggregated attributable fractions for diarrheal risk factors among children younger than 5 years by Socio-demographic Index quintile in 2017. This figure is analogous to Figure 3 in the main text but here the grouping is by SDI quintile instead of GBD super region. In the comparative risk factors framework used in GBD 2017, risk factors are counterfactual and can overlap such that a single risk may be sufficient to cause a diarrhea death but is not necessary. Therefore, although the *Total* risk attribution cannot exceed 1, there could be overlap between rotavirus *Vaccine, ORS Treatment, Nutrition* or *WASH* associated risk factors at the population level such that eliminating exposure to one would avert a diarrhea death.

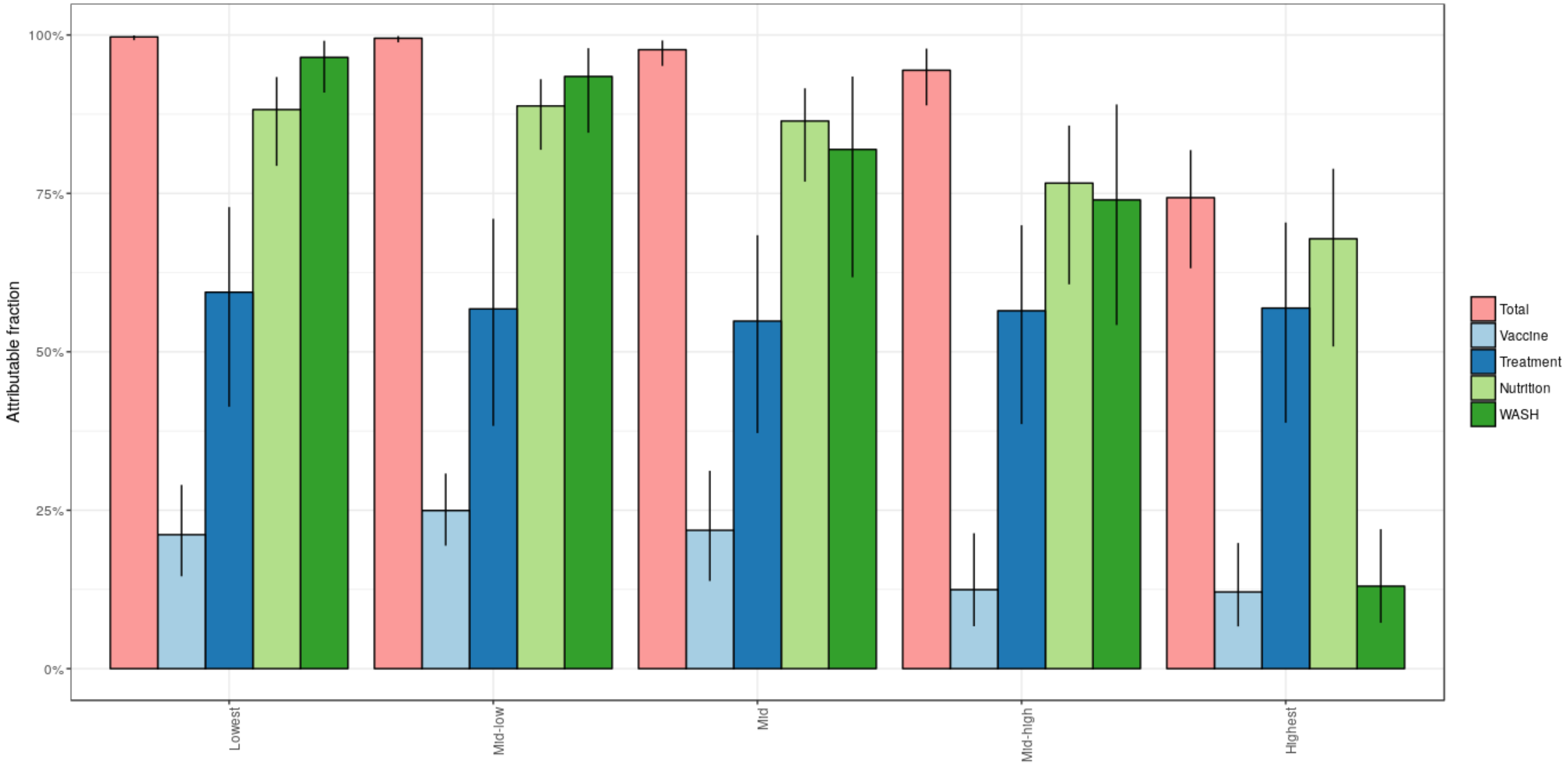

Supplement: Supplementary appendix [file mmc1.pdf]
